# A combined aqueous extract of *Psidium guajava* L. and *Tithonia diversifolia* (Hemsl.) A. gray improves metabolic homeostasis and preserves intestinal integrity in experimental rabbit colibacillosis

**DOI:** 10.1371/journal.pone.0357951

**Published:** 2026-09-15

**Authors:** Mireille Tuedom Chouegouong, Rufin Marie Toghueo Kouipou, Yanick Kevin Melogmo Dongmo, Jaurès Marius Tsakem Nangap, Michele Stella Majoumouo, Brice Pinlap, Kingsley Agbor Etchu, Fabrice Fekam Boyom

**Affiliations:** 1 Antimicrobial and Biocontrol Agents Unit (AmBcAU), Laboratory for Phytobiochemistry and Medicinal Plants Studies, Department of Biochemistry, Faculty of Science, University of Yaoundé I, Messa-Yaoundé, Cameroon; 2 Institute of Agricultural Research and Development (IRAD), Yaoundé, Cameroon; 3 Laboratory for Antibiotic Resistance and Microbial Metabolomics, Institute of Microbiology, Czech Academy of Sciences, Prague, Czech Republic; 4 Laboratory of Animal Physiology, Department of Animal Biology and Physiology, Faculty of Science, University of Yaoundé 1, Yaoundé, Cameroon; 5 Division of Pulmonary, Critical Care and Sleep Medicine, Department of Internal Medicine, University of Cincinnati College of Medicine, Ohio, United States of America; Kwame Nkrumah University of Science and Technology, GHANA

## Abstract

**Background:**

Colibacillosis, caused by pathogenic *Escherichia coli*, remains a significant threat to rabbit production, particularly during the critical weaning period. With the emergence of multidrug-resistant strains limiting conventional antibiotic efficacy, there is an urgent need for sustainable phytotherapeutic alternatives. This study evaluated the therapeutic efficacy of a combined aqueous leaf extract of *Psidium guajava* L. and *Tithonia diversifolia* (Hemsl.) A. Gray on systemic biological markers and organ integrity in a rabbit model of colibacillosis.

**Methods:**

Thirty healthy rabbits were randomly grouped into six cohorts: negative control (infected-untreated), healthy control (uninfected-untreated), positive control (infected-treated with Neomycin 10 mg/kg), immunodepressed (uninfected-cyclophosphamide), and two treatment groups (infected; receiving 400 or 800 mg/kg of the combined *P. guajava* and *T. diversifolia* extract). Colibacillosis was induced via oral administration of a pathogenic *E. coli* isolate (1 mL/100 g body weight; 10⁹ CFU/mL). Systemic recovery was assessed through hematological, biochemical, and antioxidant profiling, alongside histological examination and multivariate Principal Component Analysis (PCA). The data were analysed using OriginLab Pro 2025 and GraphPad Prism version 8.0.1 and all multivariate analyses and visualizations were performed in the R statistical environment (v4.4.3).

**Results:**

*E. coli* infection triggered severe haematological, biochemical, and inflammatory dysregulation, characterized by significant increases in white blood cell counts, aspartate aminotransferase, creatinine, and triglycerides, concomitant with reduced red blood cell counts, haemoglobin, and albumin. The combined extract demonstrated dose-dependent efficacy. Notably, multivariate PCA mapping revealed that the 800 mg/kg dose achieved a superior therapeutic outcome, effectively resolving clinical diarrhoea and restoring biological profiles to states comparable to those of the healthy and Neomycin-treated cohorts. Furthermore, the extract significantly mitigated systemic oxidative stress and protected organ integrity.

**Conclusion:**

These findings demonstrate the therapeutic efficacy and potential of the combined *P. guajava* and *T. diversifolia* extract in managing colibacillosis and associated systemic inflammation in rabbits. This study supports the traditional ethnomedicinal use of these plants and demonstrate that these combined extracts could be develop as natural alternatives to synthetic antibiotics in veterinary medicine for managing colibacillosis in rabbits.

## Introduction

In the face of escalating global climate change and the urgent need for sustainable food systems, rabbit farming (*Oryctolagus cuniculus*) has emerged as an environmentally superior alternative to traditional livestock [1,2]. Characterized by a low carbon footprint, minimal water requirements, and the ability to thrive on non-competitive forage, the domestic rabbit industry is increasingly recognized as a cornerstone of eco-friendly agriculture and a vital contributor to rural economic development and food security worldwide [2]. Global rabbit meat production reached approximately 860,000 tons in 2021, with China leading the sector (53.1%), followed by a growing importance in developing nations [3]. This prominence of farming is driven by the rabbit’s favourable biological characteristics, notably their high fecundity and efficient feed conversion [4]. Furthermore, rabbit meat is a highly nutritious, lean protein source, rich in essential micronutrients such as iron, zinc, and vitamin B12 [5].

Despite this potential, the rabbit farming industry faces substantial health challenges, with enteric diseases representing the most economically devastating hurdle [6]. Infectious diarrhoea remains a primary cause of mortality and reduced growth performance, leading to high veterinary costs [7]. Colibacillosis, caused by enteropathogenic *Escherichia coli* (EPEC), is the most prevalent bacterial threat to young rabbits [6]. The pathogenesis of EPEC is defined by a sophisticated cytokine cascade: bacterial attachment (BFP) and effector protein injection (T3SS) trigger an immediate innate response (interleukin-1β (IL-1β) and interleukin-6 (IL-6)), which subsequently activates a Th1-mediated adaptive immune response [8]. The resulting attaching and effacing (A/E) lesions destroy the intestinal brush border, compromising the epithelial barrier and allowing systemic cytokines to exacerbate fluid loss and diarrhoea [8]. Furthermore, this bacterial colonization triggers an intense oxidative stress response. The host’s ability to survive this oxidative storm depends heavily on the mobilization of its antioxidant defences system, particularly superoxide dismutase (SOD) and glutathione peroxidase (GPX) [9].

The conventional management of colibacillosis has historically relied on a narrow spectrum of antimicrobials, predominantly trimethoprim-sulfamethoxazole and fluoroquinolones [10]. However, the livestock sector is now at a critical juncture due to the global escalation of antimicrobial resistance (AMR). Recent surveillance indicates a dramatic rise in multidrug-resistant (MDR) *E. coli* strains in rabbitries, with some isolates exhibiting total resistance to watch-list antibiotics like tetracyclines and aminoglycosides [11,12]. This phenomenon is not merely a veterinary concern, but a significant public health threat, as the transmission of resistant genes from livestock to the human food chain complicates the treatment of enteric infections in people [13,14]. Furthermore, the increasing regulatory restrictions on antibiotic growth promoters (AGPs) and the growing consumer demand for antibiotic-free animal products have rendered the status quo untenable [15,16]. Consequently, there is an urgent imperative to transition toward sustainable, bio-based therapeutic alternatives that can achieve clinical resolution without contributing to the environmental resistome.

Ethnoveterinary medicine offers a robust reservoir for therapeutic discovery, particularly through species such as *P. guajava*. (Myrtaceae) and *T diversifolia* (Asteraceae). While *P. guajava* is widely used for its tannin-rich antidiarrheal properties [17] and *T. diversifolia* for its anti-inflammatory sesquiterpene lactones [18], their selection for this study was based on the results from our previous investigation. In our preceding ethnopharmacological survey of rabbitries in Cameroon, these two species emerged as the most prominent remedies used by local farmers to manage bacterial enteritis [19]. Subsequent *in vitro* screening of forty-five different preparations revealed that only six extracts, exhibited potent antibacterial activity against pathogenic *E. coli*. Notably, the combination of *T. diversifolia* and *P. guajava* yielded a synergistic interaction, achieving a Fractional Inhibitory Concentration Index (FICI) as low as 0.265 and reducing individual minimum inhibitory concentration (MIC) values by up to 64-fold [19]. This specific formulation demonstrated a high biological potency index, consistently maintaining activity levels that justify further investigation. Consequently, the present study was designed to validate these synergistic findings *in vivo*, utilizing an integrated multivariate approach to determine if the combination of *P. guajava* and *T. diversifolia* extracts can resolve systemic colibacillosis and restore host homeostasis as effectively as conventional antibiotherapy.

## Materials and methods

### Ethical approval, ethical statement, and animal welfare

All experimental protocols were reviewed and approved by the Cameroon National Ethical Committee (Ref No. FWIRB00001954). The study was conducted in strict accordance with the ARRIVE guidelines (Animal Research: Reporting of In Vivo Experiments).

### Ethnobotanical selection and extract preparation

The selection of plant materials was based on our previous ethnopharmacological survey, identifying active remedies against rabbit bacterial enteritis [19].

Fresh leaves of *P. guajava* (Myrtaceae) and *T. diversifolia* (Hemsl.) A. Gray (Asteraceae) were collected in April 2024 (corresponding to the onset of the rainy season) at approximately 07:00 h (local time) in Yaoundé, Cameroon. The botanical samples were authenticated at the National Herbarium of Cameroon (Voucher Nos. 2884 SRFK and 57410 HNC, respectively). The fresh leaves were air-dried at ambient temperature (25°C) for 14 days until a constant weight was achieved, followed by mechanical pulverization into a fine powder. Fresh leaves of *Psidium guajava* weighing 2,204 g produced 500 g of dry powder, corresponding to an approximate fresh-to-dry ratio of 4.4:1. Similarly, 3,774 g of fresh *Tithonia diversifolia* leaves yielded 500 g of dry powder, equivalent to an approximate fresh-to-dry ratio of 7.5:1. The extracts were prepared using cold extraction with distilled water at room temperature to mimic the traditional preparation, ensuring high extraction yield. 500 g of powder from each plant species was macerated in 2 L of distilled water for 72 h under continuous agitation. The mixtures were filtered through hydrophilic cotton, and the filtrates were dehydrated under forced ventilation at 25°C to remove the residual solvent. The resulting crude extracts were stored at −20°C.

### Preparation of the combined extract solutions

Combined extract formulations of *P. guajava* and *T. diversifolia* were freshly prepared in distilled water daily. The ratio of the two extracts within the combination (96.975% *P. guajava* and 3.025% *T. diversifolia*) was determined based on the synergistic Fractional Inhibitory Concentration Index (FICI = 0.265) identified in our prior *in vitro* assays [19]. Two distinct stock solutions were prepared to deliver target doses of 400 and 800 mg/kg body weight using a standardized oral gavage volume of 1 mL per 100 g body weight, equivalent to 10 mL/kg body weight. The stock solution of 40 mg/mL (for the 400 mg/kg group) was prepared by dissolving 310.32 mg of *P. guajava* extract and 9.68 mg of *T. diversifolia* extract in 8 mL of sterile distilled water. This delivered 387.9 mg/kg of *P. guajava* and 12.1 mg/kg of *T. diversifolia in vivo*. The second stock solution of 80 mg/mL (for the 800 mg/kg group) was prepared by dissolving 620.64 mg of *P. guajava* extract and 19.36 mg of *T. diversifolia* extract in 8 mL of sterile distilled water. This delivered 775.8 mg/kg of *P. guajava* and 24.2 mg/kg of *T. diversifolia in vivo*.

### Characterization and virulence of the clinical isolate

A clinical isolate of *E. coli* was recovered from a rabbit presenting acute colibacillosis symptoms. Identification was performed using the API 20E analytical profile index (bioMérieux, France). Virulence potential was assessed via the Congo red binding assay [20] on tryptic soy agar supplemented with 0.003% Congo red and 1.5% bile salts. Red-pigmented colonies, indicative of virulent plasmid expression, were selected and cultured in nutrient broth supplemented with bile salts (0.4%). Inoculum turbidity from a 24-hour culture was adjusted to 4 McFarland, corresponding to a concentration of approximately 10^9^ CFU/mL in sterile 0.9% saline [21].

### Experimental animals

Thirty hybrid rabbits (mixed sex: *three females and two males*, 6–7 weeks old, 800–900 g) were acclimatized for 7 days at the Laboratory of Phytobiochemistry and Medicinal Plant Studies, University of Yaoundé I. Animals were housed in pairs in galvanized cages (35 cm × 40 cm × 60 cm) under a 12-hour light/dark cycle. They were provided ad libitum access to water and a standard pelleted diet (composition: 21 kg corn flour, 4 kg cassava flour, 20 kg *Pennisetum* sp., 31 kg wheat bran, 8 kg palm kernel cake, 6 kg soybean cake, 1 kg bone meal, 4 kg palm oil, and 5 kg dried fish). Following acclimatization, rabbits were weighed to establish a baseline, ensuring comparable mean body weights across all experimental groups.

### Experimental design and induction of colibacillosis

Animals were randomly assigned to one of six experimental cohorts (n = 5 per group) using a computer-generated randomization sequence (www.random.org), to ensure unbiased group allocation. Throughout the study, the investigators adhered to a strict blinding protocol; all samples and histological slides were labelled with anonymous codes, which were unblinded only after the completion of the final statistical analysis. The experimental groups were designated as follows: T0 (normal control, non-infected, receiving sterile water), T1 (negative control, infected, untreated), T2 (positive control, infected, treated with Neomycin 10 mg/kg p.o. for 5 days), T3 (low dose, infected, 400 mg/kg combined extract p.o.), T4 (high dose, infected, 800 mg/kg combined extract p.o.), and T5 (immunosuppression control, non-infected, receiving 30 mg/kg cyclophosphamide i.p.). The rabbits in groups T1 to T4 were pretreated 48 hours before *E. coli* inoculation with a combination of cyclophosphamide (30 mg/kg body weight, administered intraperitoneally) and streptomycin (150 mg/kg bw, orally) to establish an experimental model of acute colibacillosis. This dual-pretreatment protocol is essential in adult lagomorphs, as streptomycin disrupts resident microflora to overcome colonization resistance, while cyclophosphamide induces a transient state of immunosuppression, effectively mimicking the susceptibility of weanling or immunologically stressed rabbits to enteropathogenic *E. coli* colonization. Following these physiological disruptions, colibacillosis was induced in groups T1 through T4 via oral gavage of 1 mL of an *E. coli* suspension (10^9^ CFU/mL in 0.9% saline). Therapeutic interventions were administered daily (10 mL/kg bw) for 7 consecutive days, commencing 24 hours post-infection. To ensure uniform experimental conditions and control for handling stress, animals in the T5 group, pretreated with cyclophosphamide (30 mg/kg bw, intraperitoneally) received a daily oral gavage of the vehicle (10 mL/kg bw of sterile distilled water) for the same 7-day duration.

### Clinical and microbiological monitoring

Daily faecal samples were collected to quantify bacterial shedding. Stool samples (10 mg) were serially diluted (10^−5^) in sterile saline and plated on MacConkey agar. After 24 h incubation at 37°C, typical *E. coli* colonies were counted and expressed as Log_10_ CFU/g of stool. Growth performance was monitored by weighing rabbits every two days.

### Ethical considerations and humane endpoints

A strict clinical monitoring protocol was implemented due to the risk of severe morbidity associated with inducing acute colibacillosis. Rabbits were monitored three times daily (every 8 hours) by for clinical signs of distress. Humane endpoints were strictly defined as: (i) loss of >20% of initial body weight; (ii) severe lethargy or inability to access food/water; (iii) persistent recumbency or lack of righting reflex; (iv) laboured or agonal breathing; and (v) hypothermia or severe dehydration (skin tent >2 seconds). To avoid needless suffering, animals that satisfied one or more of these criteria were immediately subjected to humane euthanasia. In this study, only one animal in the T1 (Negative Control) group reached humane endpoints on Day 3 post-infection due to severe dehydration and was euthanized immediately. No animals died spontaneously during the experimental period.

### Anaesthesia and methods of sacrifice

On Day 7 post-infection (PI), a total of 24 surviving rabbits (n = 4 per group) were humanely sacrificed for sample collection. The remaining five healthy animals from the treatment groups were excluded from terminal sampling and were maintained under standard husbandry conditions for continued post-experimental monitoring. Rabbits were transferred to a separate, quiet procedure room to promote animal welfare and preserve the integrity of the experimental data. Animals were fasted for 12 hours before euthanasia to reduce anaesthesia-related complications. Deep surgical anaesthesia was induced via intramuscular injection of ketamine (30 mg/kg) combined with diazepam (10 mg/kg). The depth of anaesthesia was strictly verified by the complete abolition of the pedal withdrawal and corneal reflexes before any procedures commenced. Euthanasia was completed under deep anaesthesia via cervical dislocation (for rabbits under 1 kg) or exsanguination via cardiac puncture, ensuring a rapid and painless death in accordance with AVMA Guidelines for the Euthanasia of Animals.

### Sample collection, systemic antioxidant and histopathological analysis

Blood was collected for haematology (EDTA tubes) and serum biochemistry (dry tubes). Haematological parameters included white blood cells (WBC), red blood cells (RBC), Haemoglobin (HGB), Haematocrit (HCT), and leukocyte differentials, analysed using a Mindray BC-5300 automated analyser. Serum biochemistry included Alanine aminotransferase (ALT), aspartate aminotransferase (AST), Gamma-Glutamyl Transferase (GGT), Creatinine, Uric Acid, Total Protein, and lipid profile, including Total Cholesterol (TC), Triglycerides (TG), and High-Density Lipoprotein cholesterol (HDL), analysed using commercial kits (LABKIT, France). Cytokine levels [Tumor Necrosis Factor alpha (TNF-α), interleukin-1beta (IL-1β), interleukin-6 (IL-6), interleukin-10 (IL-10), and C-reactive protein (CRP)] were quantified via sandwich ELISA (Quantikine® Rabbit ELISA kits, Sigma-Aldrich).

Tissue samples from the ileum and caecum, liver, and kidney were collected post-mortem for antioxidant assays and histopathological analysis. Ileum and caecum segments were homogenized in 50 mM Tris-HCl buffer (pH 7.4) and centrifuged at 1500 g for 25 min. Supernatants were analysed for Malondialdehyde (MDA) [22], reduced glutathione (GSH) [23], Superoxide dismutase (SOD) [24] and Catalase (CAT) [25].

For histopathological evaluation, liver, kidney, and caecum sections were fixed in 10% buffered formalin, embedded in paraffin wax. Sections (5 μm thick) were cut using a rotary microtome and stained with haematoxylin and eosin (H&E). Photomicrographs were captured using a digital camera attached to a light microscope. Tissue sections were subjected to a rigorous, independent qualitative descriptive analysis focusing on the presence, distribution, and resolution of classical pathological signs associated with enteric *E. coli* infection, including epithelial desquamation, villous blunting, inflammatory cell infiltration, and vascular congestion. After all sample collection, rabbit carcasses were autoclaved before disposal in compliance with biosafety regulations.

### Advanced statistical and multivariate analysis

The data were analysed using OriginLab Pro 2025 and GraphPad Prism version 8.0.1 (GraphPad Software, San Diego, CA, USA). All quantitative data are expressed as mean ±standard deviation (SD). Before hypothesis testing, the normality of data distribution was verified using the Shapiro–Wilk test, and homogeneity of variances was confirmed via Levene’s test. Univariate comparisons across the six experimental groups were performed using one-way Analysis of Variance (ANOVA), followed by Tukey’s Honest Significant Difference (HSD) post-hoc test to control for multiple comparison inflation and Type I error accumulation. Statistical significance was defined at P < 0.05.

An integrated multivariate approach was performed in the R Studio environment (v4.4.3) to evaluate the holistic therapeutic trajectory. Principal Component Analysis (PCA) was utilized to reduce dataset dimensionality, with variables auto-scaled (mean-centred and standardized) to account for differing units. We further generated a global biological fingerprint using hierarchical clustering, where heatmaps were constructed from Z-score-normalized data using Euclidean distance and Ward’s linkage method to visualize metabolic and inflammatory shifts across cohorts. A direct comparison between the 800 mg/kg dose and Neomycin (10 mg/kg) was conducted to assess clinical non-inferiority, defined by the absence of significant divergence (P > 0.05) in recovery endpoints.

The study sample size (n = 4 per group) was justified by an *a priori* power analysis. Based on historical indices of acute enteric inflammation (anticipating a Cohen’s f ≥ 0.70), this sample size was sufficient to achieve a statistical power (1 – β) of 0.80 at α = 0.05, consistent with the 3Rs (Reduction) ethical framework. All multivariate analyses and visualizations were performed in the R statistical environment (v4.4.3) using the tidyverse for data manipulation, FactoMineR and factoextra for multivariate modelling, and ggplot2 (ggrepel) for high-resolution, colour-blind friendly graphical representation.

## Results

### Biochemical identification of *E. coli* isolates and virulence testing

**Fig 1:**
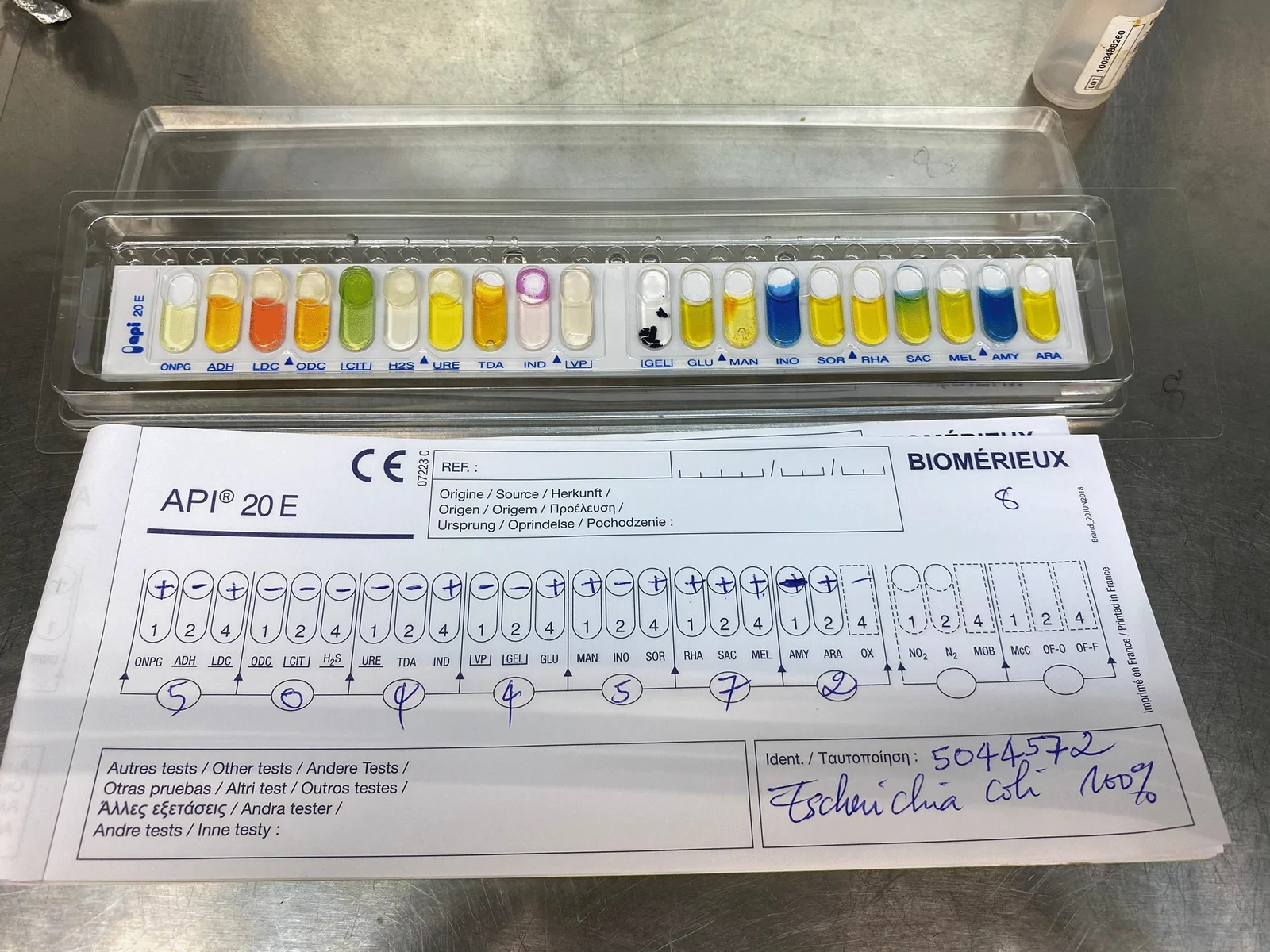
Phenotypic characterization of the pathogenic *E. coli* isolate using the API 20E analytical profile index system. Photograph of an API 20E strip (bioMérieux, Marcy-l’Étoile, France) showing the colorimetric reaction pattern generated by the clinical isolate across the 20 miniaturised biochemical test compartments after 24 h of incubation at 37 °C. The resulting 7-digit numerical profile (5044572) identified the isolate as Escherichia coli with an excellent identification percentage (>99.9%), determined using the apiweb identification software (bioMérieux). The isolate was subsequently confirmed as pathogenic using the Congo red binding assay (see Methods) prior to use in the experimental challenge described in Figs 2–14.

The clinical isolate recovered from symptomatic rabbits was subjected to standardized biochemical characterization using the API 20E system (bioMérieux, Marcy l’Etoile, France). The isolate exhibited characteristic carbohydrate fermentation patterns, including positive reactions for glucose, sorbitol, and arabinose, while remaining negative for urease and H_2_S production. The resulting biochemical profile confirmed the identity of the pathogen as *E. coli* with an identification probability of 100% (API 20E profile 5044572; identification quality: very good; probability: 100% for *E. coli* 1), validating its use for the subsequent experimental infection model (Fig 1, S1 Table in S1 File). The virulence potential of the *E. coli* isolate was phenotypically confirmed via the Congo red binding assay. The isolate exhibited strong dye uptake, resulting in distinct, red-pigmented colonies (CR+) after 24 hours of incubation (S1 Fig in S1 File). This positive phenotype indicates the presence of virulence-associated surface proteins and curli fimbriae, validating the isolate’s capacity for host colonization and its suitability for inducing experimental colibacillosis.

### Effects of the combination of *Psidium guava* and *Tithonia diversifolia* extracts on the body weight of experimentally infected rabbits

**Fig 2:**
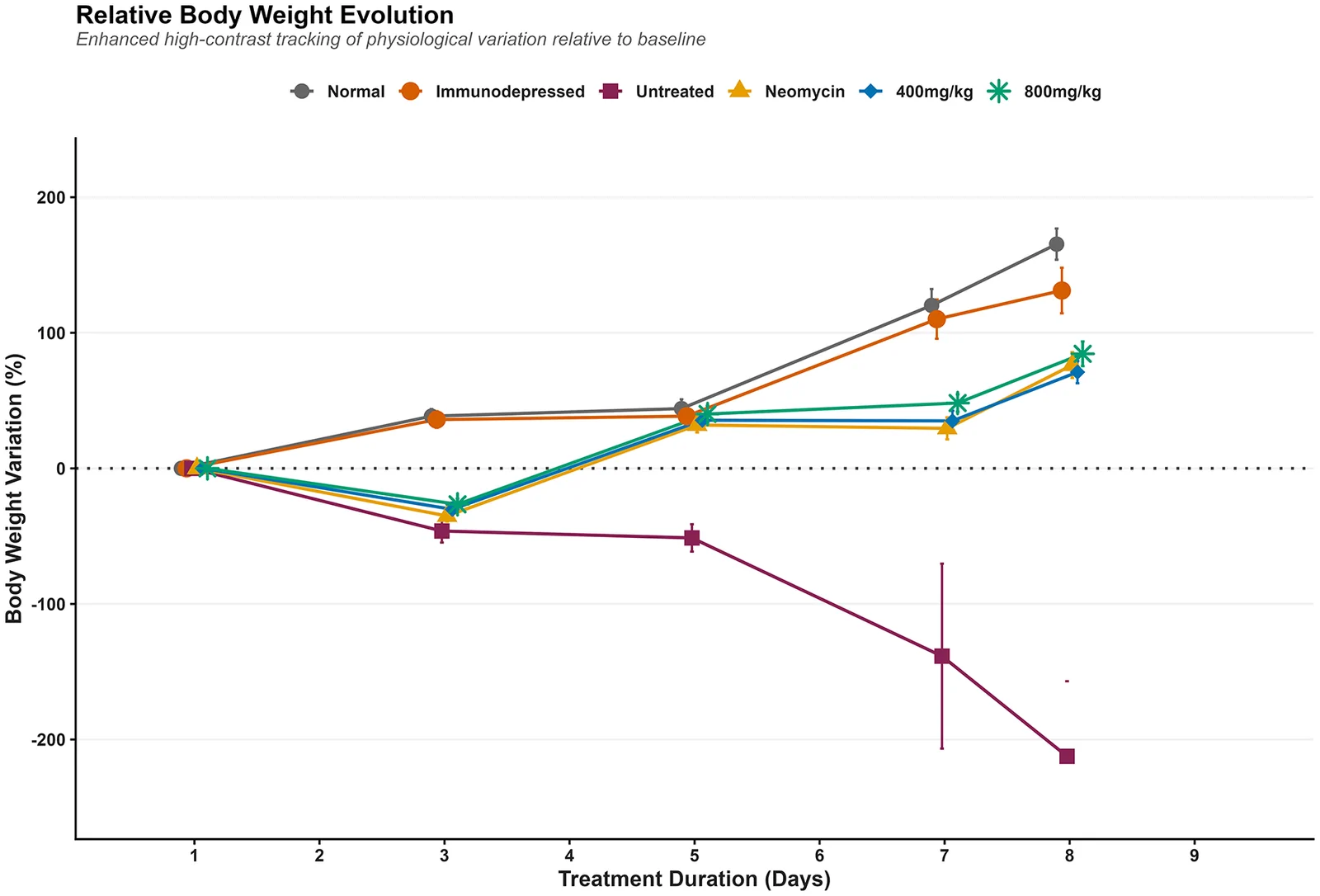
Effect of the combined aqueous extracts of *Psidium guajava* and *Tithonia diversifolia* on the body weight variation in colibacillosis-infected rabbits. Longitudinal relative body weight variation (%, expressed relative to the Day 0 pre-infection baseline) monitored over 8 days following Escherichia coli challenge in six experimental groups: Normal (uninfected, untreated control); Immunosuppressed (uninfected, cyclophosphamide-only control); Untreated (infected, untreated); Neomycin (infected, treated with neomycin, 10 mg/kg body weight (bw), reference antibiotic); 400 mg/kg bw (infected, treated with the combined extract at the low dose); and 800 mg/kg bw (infected, treated with the combined extract at the high dose). Data are mean ± standard deviation (SD), n = 4 animals per group. Group differences at each time point were assessed by two-way repeated-measures ANOVA followed by Tukey’s honestly significant difference (HSD) post-hoc test (GraphPad Prism version 8.0.1; GraphPad Software, San Diego, CA, USA); P < 0.05 was considered significant.

The effect of *E. coli* infection and subsequent treatment with the combination of *T. diversifolia* and *P. guajava* extracts on the growth trajectory of rabbits is presented in Fig 2 (S2 Table in S1 File). Following the experimental challenge, a significant divergence in body weight was observed between the study cohorts. The infected, untreated group (T1) exhibited a sharp and persistent decline in body weight throughout the 7-day post-infection (PI) period. This pathological weight loss is associated with severe malabsorption of nutrient and dehydration from infectious diarrhoea.

In contrast, rabbits treated with the combined *P. guajava* and *T. diversifolia* extracts exhibited a reversal of the infection-induced weight loss. From Day 3 post-infection (PI) onward, the 800 mg/kg dose (T4) promoted body weight gain following a trajectory like that of the healthy control (T0) and Neomycin-treated (T2) groups (P < 0.05). However, the 400 mg/kg dose (T3) resulted in moderate weight recovery, indicating a clear dose-dependent therapeutic response. Notably, the 800 mg/kg dose demonstrated efficacy in restoring rabbits to their pre-infection growth curves. These results suggest that the combined extract could be facilitating nutrient assimilation and helping restore osmotic and fluid-solute equilibrium.

### Effect of the combination of *Psidium guava* and *Tithonia diversifolia* extracts on the relative weight of some vital organs

**Fig 3:**
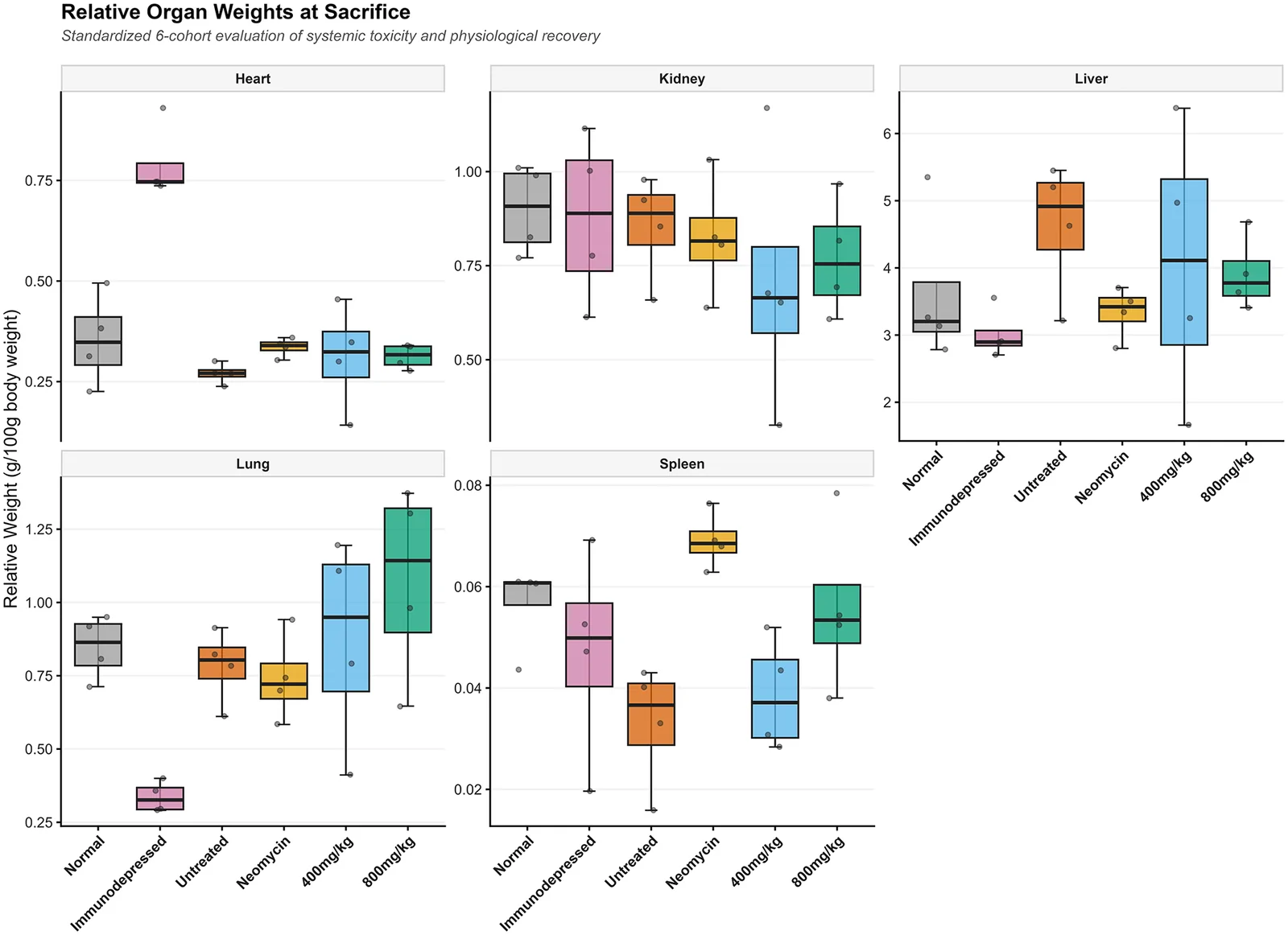
Relative organ weights at sacrifice (Day 7 post-infection) in rabbits challenged with *Escherichia coli* and treated with the combined *Psidium guajava* and *Tithonia diversifolia* extracts. Boxplots of relative organ weights (g organ weight per 100 g body weight) for the heart, kidney, liver, lung and spleen, recorded at necropsy on Day 7 post-infection. Boxes show the median and interquartile range (IQR); whiskers extend to the most extreme values within 1.5 × IQR; individual points represent single animals (n = 4 per group). Experimental groups (Normal, Immunosuppressed, Untreated, Neomycin, 400 mg/kg, 800 mg/kg) are as defined in Fig 2. Between-group differences were assessed by one-way ANOVA followed by Tukey’s HSD post-hoc test (GraphPad Prism v8.0.1); P < 0.05 was considered significant.

The macroscopic effect of *E. coli* infection and the safety profile of the combined *P. guajava* and *T. diversifolia* extracts treatment were evaluated through relative organ weight analysis and visual inspection (Fig 3). Upon necropsy, all treatment groups exhibited normal organ coloration and morphology, with no visible lesions or congestion in the liver, kidneys, or heart. Statistical analysis revealed that the relative weights of the kidneys and liver in both the 400 and 800 mg/kg extract groups showed no significant variation (P > 0.05) when compared to the healthy control (T0) and the Neomycin-treated (T2) groups.

Notably, the 800 mg/kg cohort (T4) maintained liver and spleen weights comparable to healthy controls, contrasting with the inflammatory oedema and reactive splenomegaly typically observed in untreated colibacillosis. In the infected-untreated group (T1), increased relative spleen weight suggested a heightened systemic immune response to the bacterial load. Conversely, treatment with the *P. guajava* and *T. diversifolia* combination effectively preserved organ homeostasis. These results suggest that the extract could be providing systemic protection against *E. coli*-induced pathology while maintaining the structural integrity of vital metabolic organs, without inducing adverse toxic effects.

### Therapeutic efficacy and *in vivo* bacterial clearance of the combination of *P. guajava* and *T diversifolia*

**Fig 4:**
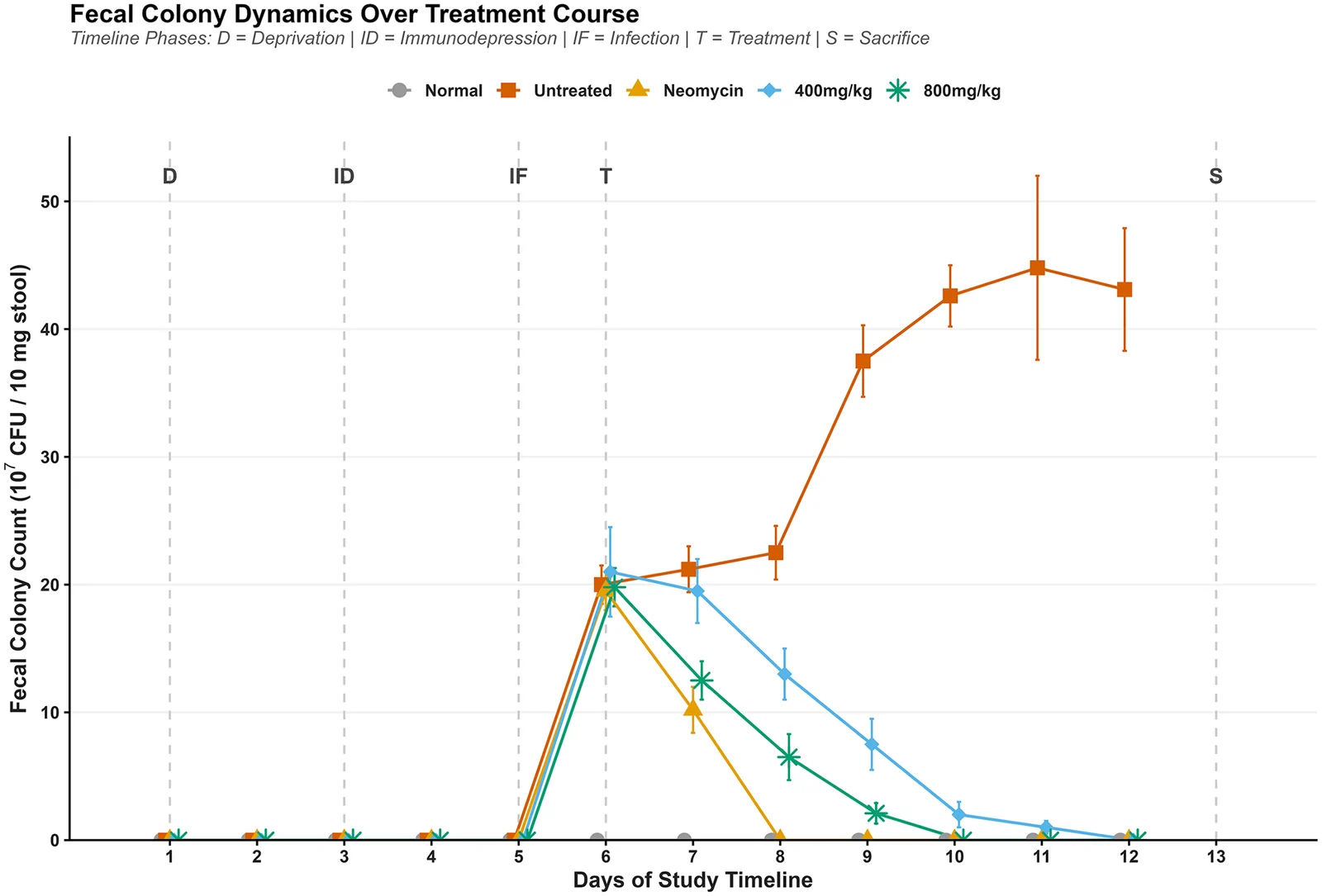
Fecal *Escherichia coli* shedding kinetics in rabbits treated with the combined *Psidium guajava* and *Tithonia diversifolia* extracts. Longitudinal fecal bacterial load, expressed as colony-forming units (CFU) × 10⁷ per 10 mg of stool, monitored across the study timeline in five experimental groups: Normal, Untreated, Neomycin (10 mg/kg bw), 400 mg/kg bw and 800 mg/kg bw (group definitions as in Fig 2). Timeline phases are indicated above the plot: D, dietary deprivation/acclimatisation period; ID, immunosuppression (cyclophosphamide administration, Day 3); IF, bacterial infection/challenge (Day 5); T, treatment period; S, sacrifice (Day 13). Data are mean ± SD, n = 4 animals per group.

The *in vivo* antibacterial potency of the *P. guajava* and *T. diversifolia* combination was assessed by monitoring the temporal kinetic of *E. coli* shedding in the faeces (Fig 4, S3 Table in S1 File). Bacterial load is a critical indicator of the extract’s ability to disrupt intestinal colonization and prevent further mucosal invasion. Following the experimental challenge, all infected groups exhibited peak bacterial shedding (10^9^ CFU/10 mg stool) before the initiation of therapy.

Treatment led to a progressive, time-dependent reduction in bacterial shedding across all cohorts. While the reference antibiotic (Neomycin, T2) achieved complete bacterial clearance within 72 hours (Day 3), the combined *P. guajava* and *T. diversifolia* extracts also demonstrated potent curative efficacy. Specifically, both the 400 mg/kg (T3) and 800 mg/kg (T4) doses resulted in no quantifiable bacterial load in faeces by Day 5 of treatment. This 5-day clearance window is clinically significant, as it correlates with the rapid resolution of diarrheic symptoms and the reversal of infection-induced weight loss observed (Fig 3). Collectively, these results indicate that the combined extract exhibits effective, dose-dependent curative activity against *E. coli*, achieving clinical recovery comparable to the reference antibiotic. These findings demonstrate that the *P. guajava* and *T. diversifolia* formulation particularly the 800 mg/kg dose could be a promising biological alternative for the management of clinical colibacillosis.

### The effect of combined aqueous extract treatment on the Antioxidant defense

**Fig 5:**
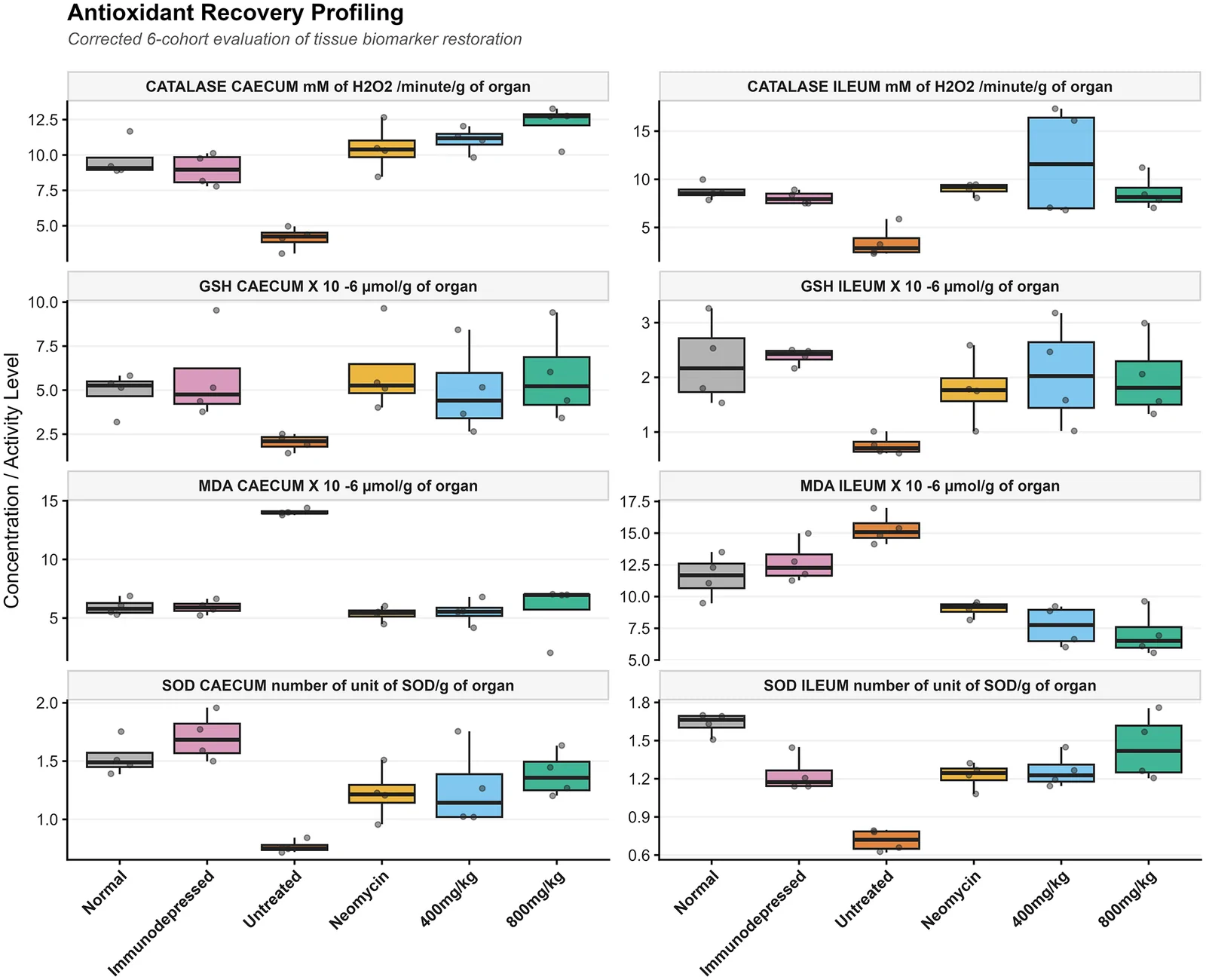
Effect of the combined *Psidium guajava* and *Tithonia diversifolia* extracts on intestinal oxidative stress markers on Day 7 post-infection. Boxplots comparing oxidative stress and antioxidant biomarkers in the caecum (left column) and ileum (right column): catalase (CAT) activity, expressed in millimoles of hydrogen peroxide (H_2_O_2_) consumed per minute per gram of tissue; reduced glutathione (GSH) concentration, expressed as × 10^-6^ µmol per gram of tissue; malondialdehyde (MDA) concentration, an indicator of lipid peroxidation, expressed as × 10^-6^ µmol per gram of tissue; and superoxide dismutase (SOD) activity, expressed in units per gram of tissue. Experimental groups (Normal, Untreated, Neomycin, 400 mg/kg, 800 mg/kg, Immunosuppressed) are as defined in Fig 2; n = 4 animals per group. Boxes show median and IQR; whiskers extend to 1.5 × IQR. Between-group comparisons: one-way ANOVA with Tukey’s HSD post-hoc test (GraphPad Prism v8.0.1); P < 0.05 was considered significant.

The impact of *E. coli* infection and subsequent treatment with combined *P. guajava* and *T. diversifolia* extracts on the intestinal redox landscape was evaluated (Fig 5; S4 Table in S1 File). In the infected-untreated cohort (T1), experimental colibacillosis induced a state of biochemical collapse within both the caecum and ileum. This pathological state was characterized by a significant increase (P < 0.05) in MDA levels linked to extensive lipid peroxidation, suggesting the depletion of the primary antioxidant defences, including SOD, CAT, and GSH. These findings demonstrated that *E. coli* colonization rapidly exhausts the host’s local antioxidant protection, leading to oxidative tissue damage.

Therapeutic administration of the *P. guajava* and *T. diversifolia* extracts effectively reversed this oxidative trend in a dose-dependent manner. While the 400 mg/kg dose (T3) provided moderate protection, the 800 mg/kg dose (T4) achieved a superior restorative profile, returning MDA, CAT, and SOD levels to values statistically similar from those of the healthy control (T0).

Interestingly, in specific parameters such as caecal GSH and ileal SOD, the 800 mg/kg dose of combined *P. guajava* and *T. diversifolia* extracts treatment maintained the antioxidant levels that were numerically superior to the Neomycin standard (T2). This could indicate that while Neomycin serves as a potent bactericidal agent, the combined *P. guajava* and *T.diversifolia* extracts could be providing a dual-action benefit by simultaneously preventing the *E. coli* colonization but also restoring the intestinal antioxidant framework.

### The effect of combined aqueous extract treatment on haematological stabilization and resolution of systemic inflammation

**Fig 6:**
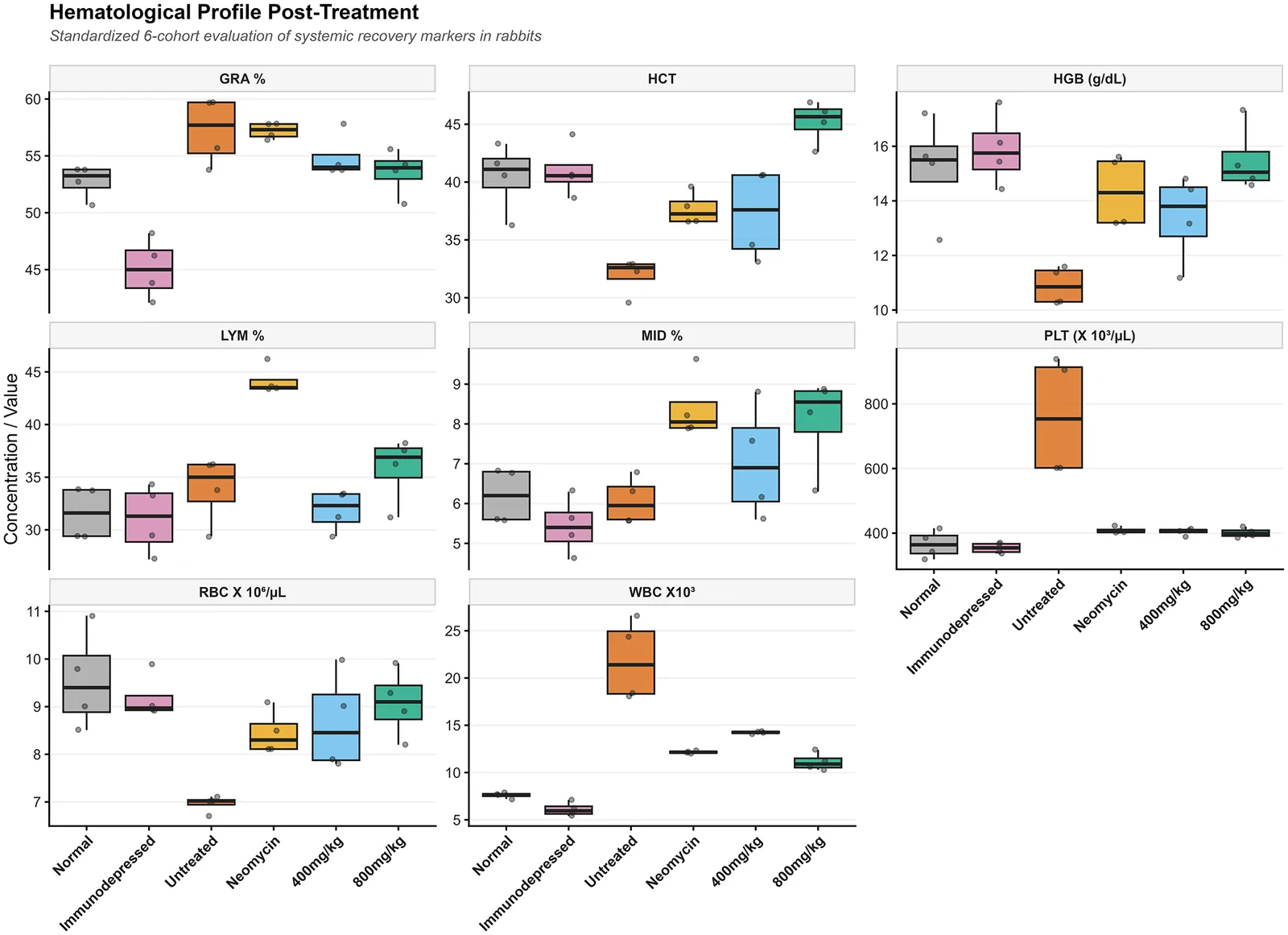
Hematological profile of rabbits on Day 7 post-infection following treatment with the combined *Psidium guajava* and *Tithonia diversifolia* extracts. Boxplots of hematological parameters: granulocyte percentage (GRA %); hematocrit (HCT, %); hemoglobin concentration (HGB, g/dL); lymphocyte percentage (LYM %); percentage of mid-sized cells (MID %; combined monocyte, eosinophil and basophil fraction); platelet count (PLT, × 10³/µL); red blood cell count (RBC, × 10⁶/µL); and white blood cell count (WBC, × 10³/µL). Six experimental groups as defined in Fig 2; n = 4 animals per group. Boxes show median and IQR; whiskers extend to 1.5 × IQR; individual points represent single animals. Between-group comparisons: one-way ANOVA with Tukey’s HSD post-hoc test (GraphPad Prism v8.0.1); P < 0.05 was considered significant.

The systemic impact of the *E. coli* challenge and the subsequent restorative effects of the *P. guajava* and *T. diversifolia* combination are summarized in the haematological profiles (Fig 6; S5 Table in S1 File). The infected-untreated group (T1) exhibited a classic septic hemogram, characterized by profound leucocytosis and thrombocytosis. Specifically, WBC counts nearly doubled and Platelet (PLT) counts nearly tripled compared to the healthy control (T0), signalling a severe systemic inflammatory response and the activation of the host’s emergency myeloid defences against *E. coli* septicaemia.

Simultaneously, the T1 cohort suffered from anaemia of infection, evidenced by significant declines (P < 0.05) in RBC counts, HGB levels, and HCT compared to all other groups. This erythrocytic collapse likely reflects the combined effects of bacterial hemolysins, iron sequestration, and the haemorrhagic nature of the colibacillosis-induced intestinal lesions.

Treatment with the combined *P. guajava* and *T. diversifolia* extracts achieved near-complete normalization of the hemogram in a dose-dependent manner. The 800 mg/kg dose (T4) proved to be particularly effective, successfully restoring the systemic inflammatory response by returning WBC and PLT counts to baseline levels. Furthermore, the bioactive extract reversed the erythrocytic decline, restoring haemoglobin concentrations from a pathological low of approximately 11 g/dL to a healthy 15 g/dL. Notably, the leukocyte differential (GRA%, LYM%, and MID %) was rebalanced in the T4 group, indicating a restored and functional immune landscape comparable to the neomycin-treated (T2) and healthy (T0) cohorts. These findings underscore the extract’s potent haematoprotective efficacy and its ability to halt the progression of the infection from a local gut disturbance to a systemic crisis.

### Immunomodulatory effects of combined aqueous extract treatment on *E. coli*-infected rabbits

**Fig 7:**
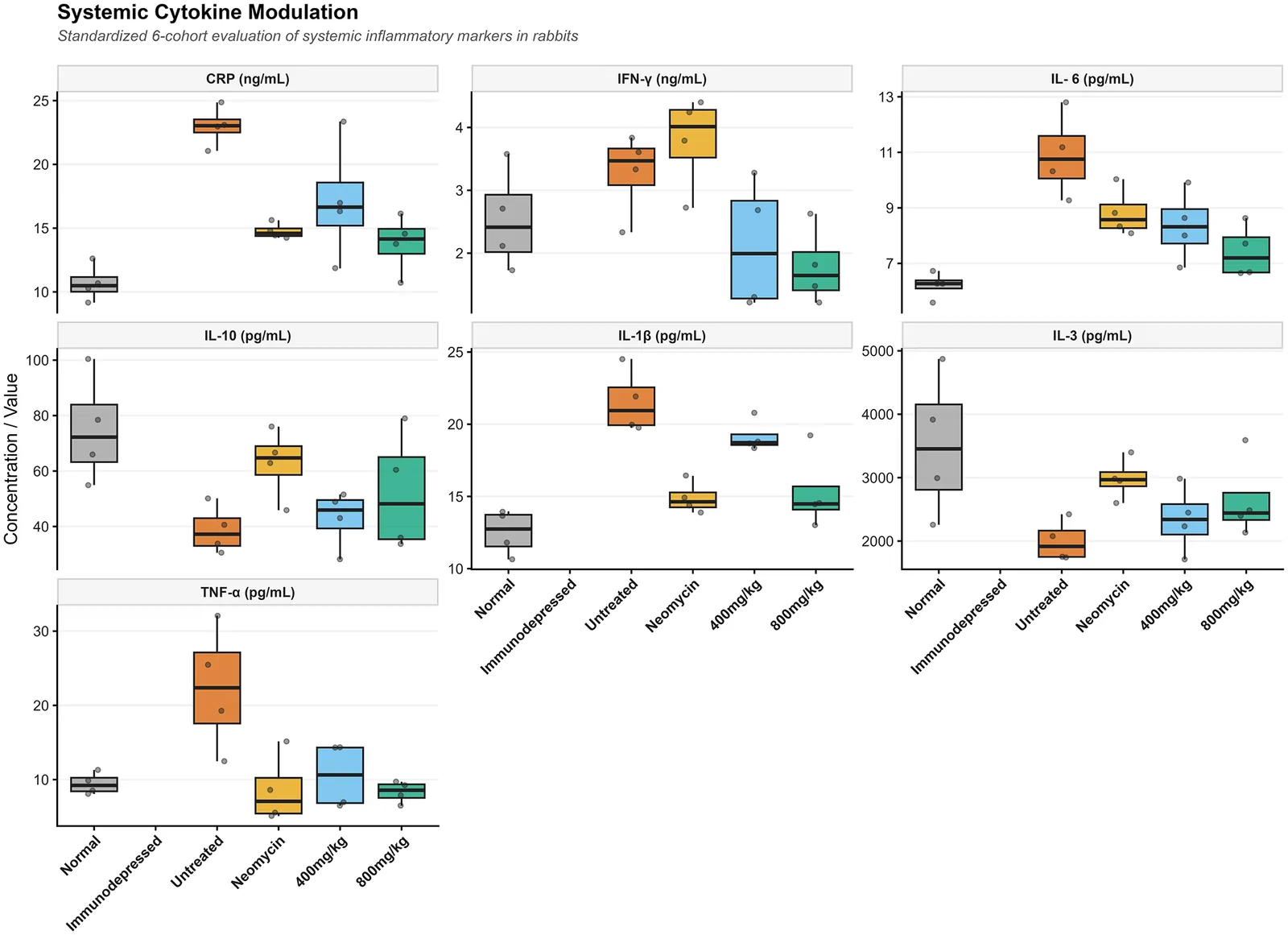
Systemic cytokine and acute-phase protein modulation by the combined *Psidium guajava* and *Tithonia diversifolia* extracts on Day 7 post-infection. Boxplots of serum concentrations of C-reactive protein (CRP, ng/mL), interferon-gamma (IFN-γ, ng/mL), interleukin-6 (IL-6, pg/mL), interleukin-10 (IL-10, pg/mL), interleukin-1β (IL-1β, pg/mL), interleukin-3 (IL-3, pg/mL) and tumor necrosis factor-alpha (TNF-α, pg/mL). Six experimental groups as defined in Fig 2; n = 4 animals per group, except where individual values fell below the analytical lower limit of detection, in which case the corresponding data point is omitted from the panel. Between-group comparisons: one-way ANOVA with Tukey’s HSD post-hoc test (GraphPad Prism v8.0.1); P < 0.05 was considered significant.

The immunological response to *E. coli* infection and the restorative capacity of the combined *P. guajava* and *T. diversifolia* extracts intervention were evaluated through key pro-inflammatory and anti-inflammatory biomarkers (Fig 7; S6 Table in S1 File). In the infected-untreated cohort (T1), experimental colibacillosis triggered a massive cytokine production, evidenced by significant elevations (P < 0.05) in CRP, TNF-α, IL-1β, and IL-6. This excessive release of innate first-responder cytokines drives the systemic inflammatory response, leading to tissue damage and metabolic exhaustion observed in the T1 group.

Oral administration of the *P. guajava* and *T. diversifolia* combination at 400 and 800 mg/kg induced a significant reduction in these pro-inflammatory markers. Notably, for critical indicators like TNF-α and IL-6, the 800 mg/kg dose (T4) was more potent, suppressing cytokine levels as effectively as the Neomycin standard (T2). This suggests that the bioactive metabolites in the extract, such as flavonoids and sesquiterpene lactones, likely modulate the underlying signalling pathways (such as NF-kB) responsible for acute inflammatory cascades.

Conversely, the anti-inflammatory cytokine IL-10 showed a distinct and clinically relevant pattern. While IL-10 levels were significantly lower in the untreated group (T1), all treated cohorts (T2, T3, T4) maintained significantly higher concentrations of this cytokine (P < 0.05). This could be an indication that the combined *P. guajava* and *T*. *diversifolia* extract does not merely suppress the immune system; rather, it facilitates immunological rebalancing by promoting an anti-inflammatory environment that accelerates tissue healing and the resolution of diarrhoea. The normalization of CRP, a standard marker of acute systemic inflammation in rabbits, further validates that the T4 dose achieved complete systemic resolution of the infection**.**

### Effects of combined aqueous extract treatment on Hepato-Renal Protection and Metabolic Homeostasis in *E. coli*-infected rabbits

**Fig 8:**
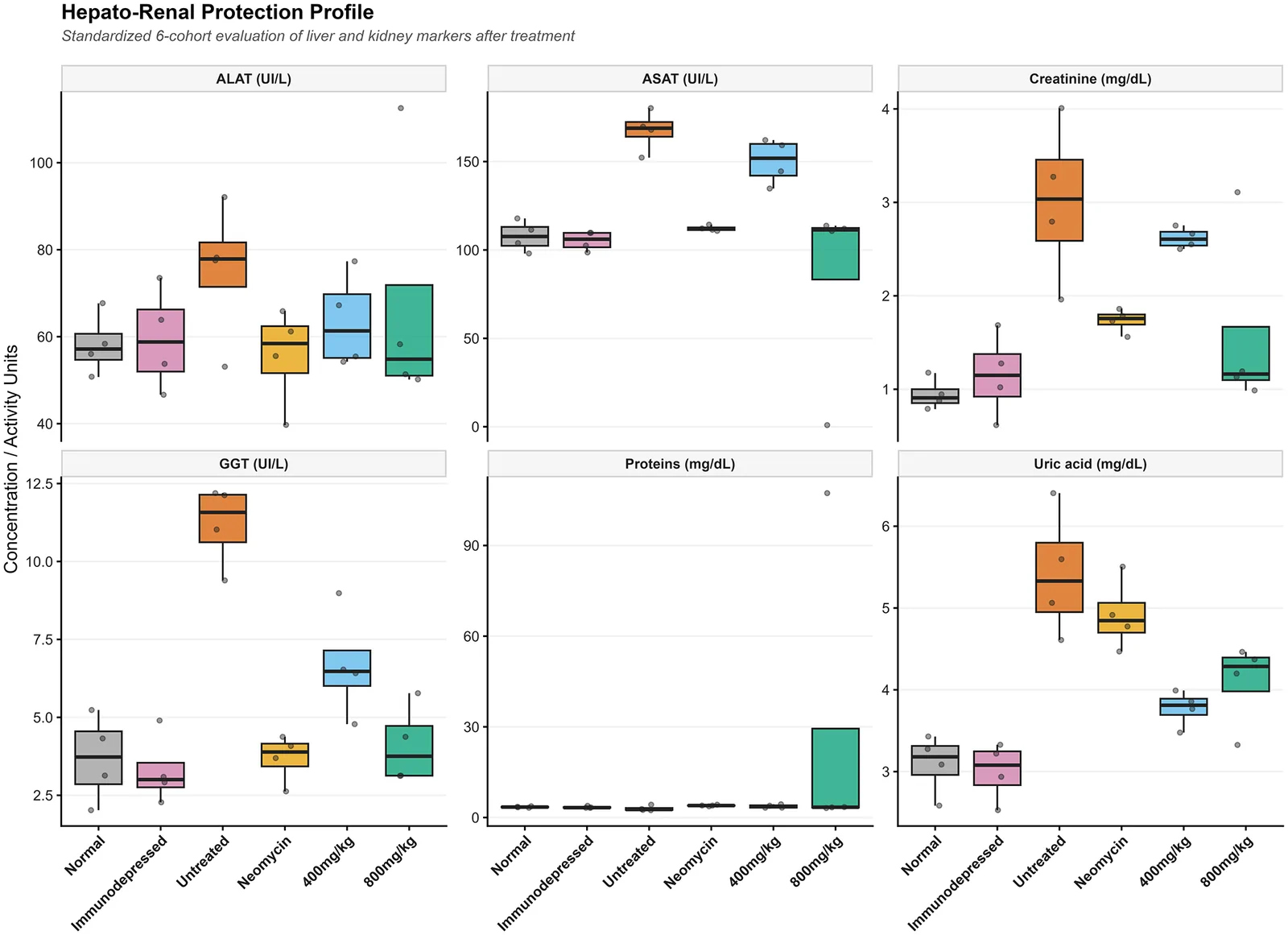
Hepatic and renal function markers in rabbits treated with the combined *Psidium guajava* and *Tithonia diversifolia* extracts on Day 7 post-infection. Boxplots of serum alanine aminotransferase (ALAT, IU/L), aspartate aminotransferase (ASAT, IU/L), creatinine (mg/dL), gamma-glutamyl transferase (GGT, IU/L), total protein (mg/dL) and uric acid (mg/dL) concentrations. Six experimental groups as defined in Fig 2; n = 4 animals per group. Between-group comparisons: one-way ANOVA with Tukey’s HSD post-hoc test (GraphPad Prism v8.0.1); P < 0.05 was considered significant.

The systemic metabolic profile and organ-functional integrity of the experimental rabbits are summarized in Fig 8 (S7 Table in S1 File). Induction of colibacillosis in the untreated cohort (T1) resulted in significant biochemical derangement (P < 0.05), characterized by a dyslipidaemia of infection and acute hepato-renal distress. Specifically, T1 rabbits exhibited an increase in serum transaminases (AST, ALT) and GGT, signalling acute hepatocellular injury and membrane leakage induced by bacterial endotoxins.

Renal function was similarly compromised in the untreated group, with Creatinine and Urate levels nearly doubling. This elevation may indicate a reduced Glomerular Filtration Rate (GFR), likely secondary to infection-induced dehydration and septic renal injury. Furthermore, a state of hypoproteinaemia and hypoalbuminemia was observed in T1, reflecting the systemic inflammatory response and potential protein-losing enteropathy associated with severe diarrhoea.

Therapeutic intervention with the *P. guajava* and *T. diversifolia* extracts effectively shielded the primary metabolic organs from this endotoxicity. The 800 mg/kg dose (T4) achieved a metabolic reset, returning AST, GGT, Creatinine, and Urate to baseline levels that were statistically indistinguishable from those of the healthy controls (T0). Notably, the extract restored the lipid profile by lowering triglycerides and LDL-cholesterol while significantly increasing HDL-cholesterol (P < 0.05) compared to the untreated group. By stabilizing hepatocellular membranes and maintaining renal blood flow, the 800 mg/kg dose showed organ-protective effects. This helped restore systemic balance comparable to the neomycin-treated group (T2).

### Integrated hierarchical clustering: The global biological fingerprint

**Fig 9:**
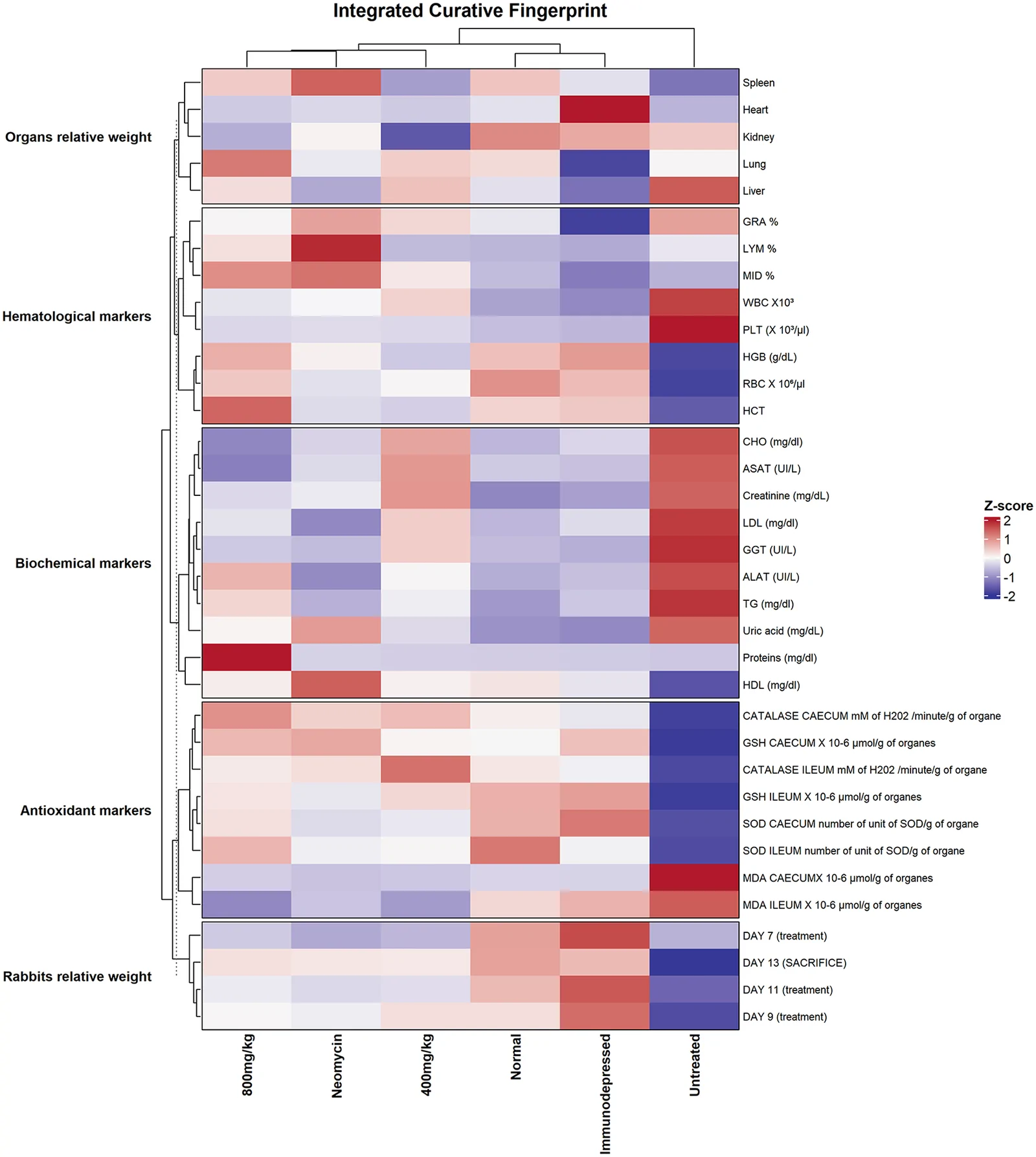
Two-way hierarchical clustering heatmap of the integrated physiological profile of rabbits at the study endpoint. Row-standardised (Z-score) heatmap of measured parameters (rows) grouped into five physiological categories: relative organ weights, hematological markers, serum biochemical markers, intestinal antioxidant markers, and longitudinal rabbit body weight (Days 7, 9, 11 and 13 post-infection). Columns represent the six experimental groups (800 mg/kg, Neomycin, 400 mg/kg, Normal, Immunosuppressed, Untreated), each summarising n = 4 animals. Red indicates values above the overall parameter mean (Z-score up to +2), blue indicates values below the mean (Z-score down to −2), and white/pale pink indicates values near the mean. Row and column dendrograms represent unsupervised hierarchical clustering based on Euclidean distance with complete linkage. Parameter abbreviations: GRA %, granulocyte percentage; LYM %, lymphocyte percentage; MID %, mid-sized cell percentage; WBC, white blood cells; PLT, platelets; HGB, hemoglobin; RBC, red blood cells; HCT, hematocrit; CHO, total cholesterol; ASAT, aspartate aminotransferase; LDL, low-density lipoprotein; GGT, gamma-glutamyl transferase; ALAT, alanine aminotransferase; TG, triglycerides; HDL, high-density lipoprotein; SOD, superoxide dismutase; GSH, reduced glutathione; MDA, malondialdehyde.

Hierarchical clustering analysis was performed on the integrated biological dataset to visualizes the holistic therapeutic trajectory of the rabbits (Fig 9). The resulting heatmap successfully discriminated between the various experimental cohorts, establishing a clear molecular signature for both the pathological state and the subsequent recovery. The infected-untreated cohort (T1) formed a distinct cluster characterized by a massive upregulation of pro-inflammatory and damage-associated markers, specifically manifesting as elevated AST, creatinine, MDA, and pro-inflammatory cytokines (TNF-α, IL-6).

In stark contrast, the cohort treated with 800 mg/kg of combined *P.guajava* and *T. diversifolia* extracts (T4) exhibited a reversal of this fingerprint. The T4 group clustered closely with the healthy control (T0) and the neomycin-treated (T2) groups, displaying a synchronized restoration of antioxidant enzymes (SOD, CAT, GSH) and a normalization of systemic biochemical indices. This global transition from the oxidative-inflammatory cluster to the homeostatic cluster underscores the multifaceted curative potential of the *P. guajava* and *T. diversifolia* combination. These results could be suggesting that the bioactive extract does not merely target isolated symptoms but induces a systemic homeostatic restoration, restoring the animal’s physiological state to its pre-infection baseline.

### Multivariate trajectory and key biological drivers

**Fig 10:**
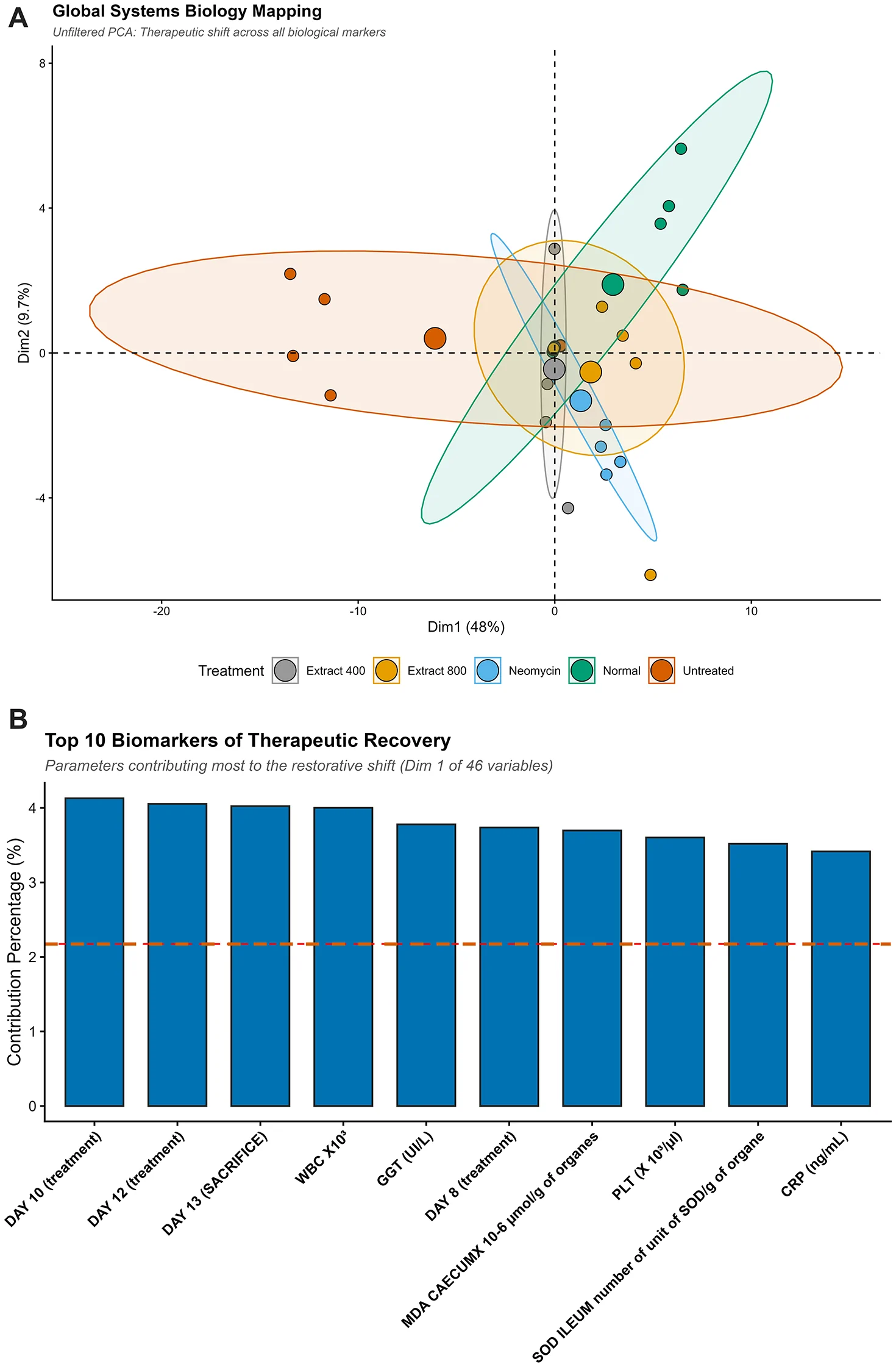
Multivariate trajectory and key biological drivers of the therapeutic response. **(A)** Unsupervised principal component analysis (PCA) score plot of individual rabbit multi-parametric profiles. Dimension 1 (Dim1) accounts for 48% and Dimension 2 (Dim2) for 9.7% of total variance. Small circles represent individual animals; large filled circles represent group centroids, each surrounded by its 95% confidence ellipse (Extract 400, grey; Extract 800, yellow; Neomycin, blue; Normal, green; Untreated, orange). **(B)** Bar plot of the top 10 parameters contributing to Dim1, expressed as percentage contribution; the dashed red line indicates the theoretical uniform contribution threshold (≈ 2.17%).

Principal Component Analysis (PCA) was applied to the combined dataset as an exploratory tool to visualize the overall multidimensional relationships and clustering trends across the experimental cohorts (Fig 10A). The unsupervised PCA model revealed a visible separation among groups, with the first two dimensions capturing a cumulative total of 57.7% of the total variance. Principal Component 1 (Dim1), which accounted for 48.0% of the variance, served as the primary axis of separation, segregating the coordinates of the infected-untreated cohort (T1) from the clustering space of the healthy controls (T0). Notably, the 800 mg/kg cohort (T4) exhibited a spatial shift along Dim1, positioning itself in closer proximity to the multivariate space occupied by the T0 and Neomycin (T2) groups. This spatial alignment suggests an exploratory trend wherein the global physiological profile of the high-dose extract cohort reflects an orientation toward the healthy and reference treatment spaces. However, given the exploratory nature of this analysis and the lack of an independent validation dataset, these spatial configurations represent descriptive clustering behaviours rather than definitive proof of systemic homeostatic equivalence.

The Variable Contribution analysis (Fig 10B) further highlighted the primary variables associated with the spatial distribution across the dimensions. The clustering trends observed in the PCA map were heavily influenced by coordinates related to bacterial clearance kinetics (load at Days 10, 12, and 13) and systemic inflammatory markers (WBC and CRP). While these variables exhibited the highest contribution scores along Dim1, these mathematical relationships remain exploratory in nature. In small-cohort designs, individual biological variation can heavily influence variable contribution weights. Thus, these parameters should be viewed as hypothesis-generating indicators, pointing to pathogen eradication and the subsequent quenching of the systemic leukocyte response as key therapeutic pathways of the extract rather than validated predictive biomarkers.

### Comparative functional efficacy and therapeutic parity

**Fig 11:**
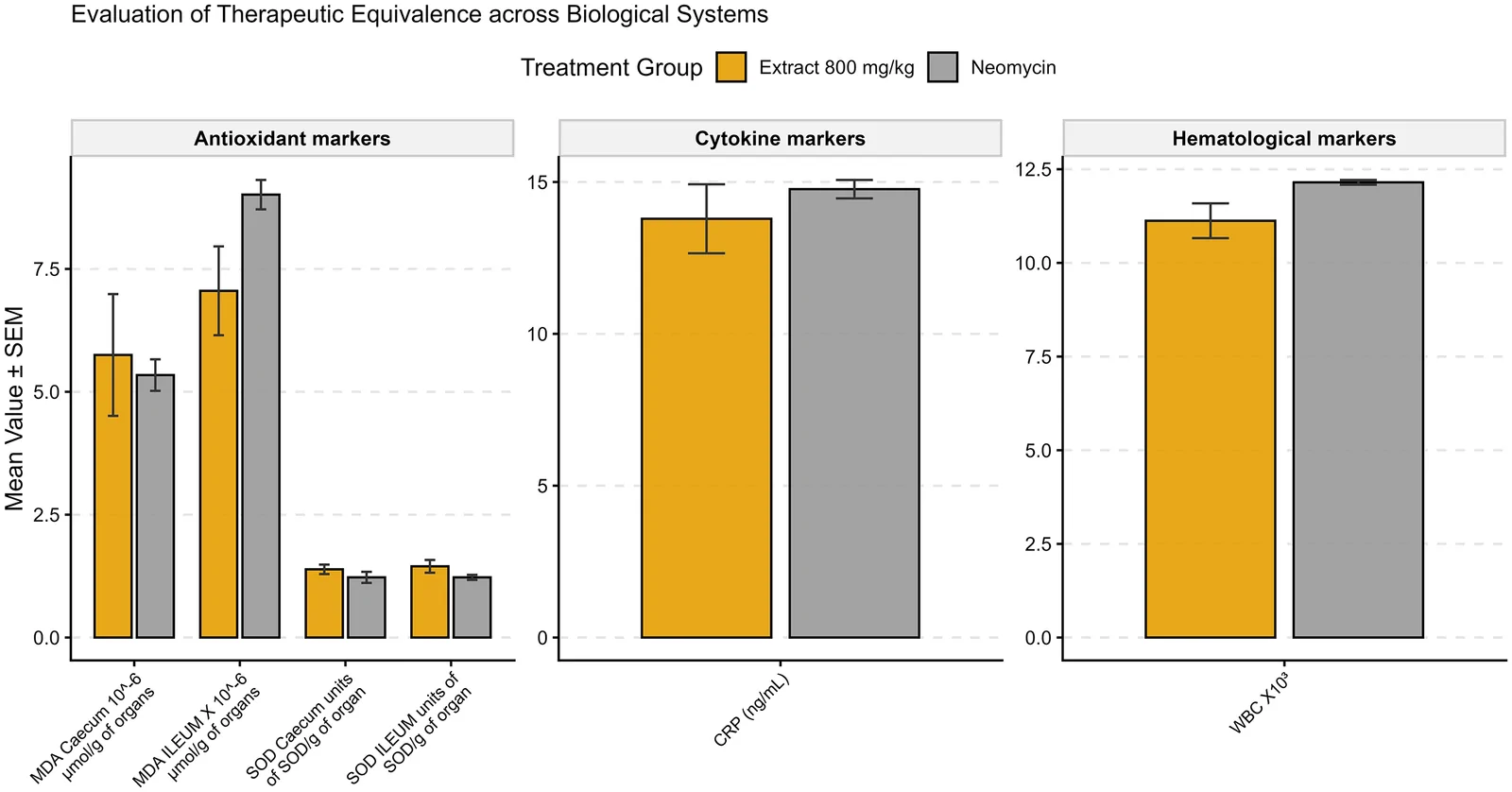
Comparative efficacy of the 800 mg/kg combined extract dose versus the reference antibiotic neomycin on Day 7 post-infection. Bar charts comparing mean ± standard error of the mean (SEM) values for the 800 mg/kg combined-extract group (yellow, n = 4) and the neomycin, 10 mg/kg group (grey, n = 4) across three physiological domains: antioxidant markers (caecal and ileal catalase, malondialdehyde (MDA) and superoxide dismutase (SOD)); the acute-phase marker C-reactive protein (CRP, ng/mL); and the hematological marker white blood cell count (WBC, × 10^3^/µL). Groups were compared for each parameter using an unpaired two-tailed t-test (or the non-parametric equivalent where normality assumptions were not met; GraphPad Prism v8.0.1). Note: No statistically significant differences (P > 0.05) were observed between the two groups for any parameter shown, indicating comparable recovery at these specific endpoints. This finding is limited to the parameters tested and should not be interpreted as evidence of overall clinical, pharmacokinetic, or safety equivalence (see Discussion, Limitations of the Study).

A comparative functional analysis was conducted between the high-dose extract (800 mg/kg) and the Neomycin standard to evaluate the clinical viability of the combination of the *T. diversifolia and P. guajava* intervention as a sustainable alternative to conventional therapy (Fig 11).

The restorative capacity of the *P. guajava* and *T. diversifolia* combination was found to be functionally equivalent to Neomycin across all oxidative stress indices. In both the caecum and ileum, the 800 mg/kg cohort exhibited a robust recovery of SOD and CAT activities, with mean values statistically indistinguishable from the antibiotic-treated group (P > 0.05).

In terms of immunomodulation, the extract achieved complete systemic stabilization. The suppression of CRP, a sensitive marker of acute-phase inflammation, in rabbits, and the normalization of WBC counts showed no significant divergence between the combined extract of *P*. *guajava and T. diversifolia* and Neomycin cohorts. This suggests that the extract possesses a systemic anti-inflammatory mechanism comparable to the resolution achieved through bacterial clearance by synthetic antimicrobials.

By resolving the *E. coli* challenge through both direct antimicrobial action and active tissue repair, the 800 mg/kg dose demonstrated therapeutic efficacy comparable to that of Neomycin. These findings suggest that this combined *P. guajava* and *T. diversifolia* extracts could be a potent bioactive agent, an alternative to conventional antibiotic that can maintain rabbit health and productivity.

## Histopathology results

### Effect of combined extract on the renal architecture

**Fig 12:**
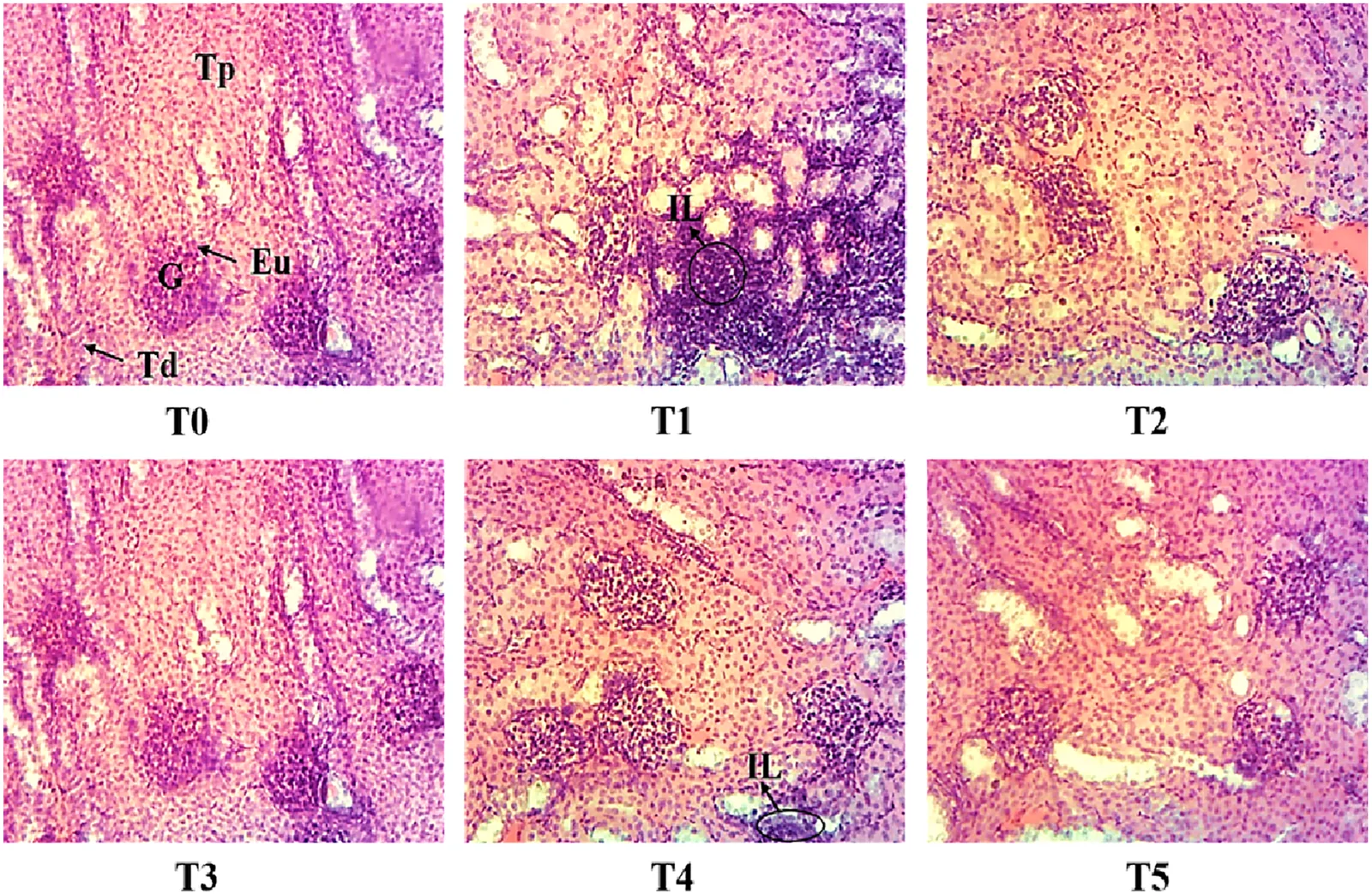
Photomicrographs of rabbit kidney sections following *Escherichia coli* challenge and treatment with the combined *Psidium guajava* and *Tithonia diversifolia* extracts (hematoxylin and eosin (H&E) stain; original magnification ×100; scale bar = 10µm). T0, uninfected control receiving distilled water only (normal control); T1, infected, untreated (vehicle control); T2, infected, treated with neomycin (10 mg/kg bw); T3 and T4, infected, treated with the combined extract at 400 and 800 mg/kg bw, respectively; T5, uninfected, cyclophosphamide-only (immunosuppressed) control. n = 4 kidneys examined per group; one representative field is shown per group. Abbreviations: Cd, cell degeneration; US, urinary space; G, glomerulus; IL, leukocyte infiltration; DT, distal convoluted tubule; PT, proximal convoluted tubule. Note: Histopathological evaluation was descriptive/qualitative, performed by a blinded pathologist; semi-quantitative lesion scoring was not performed in the present study (see Discussion, Limitations of the Study).

The protective effect of the *P. guajava* and *T. diversifolia* combination on the renal microarchitecture of *E. coli*-infected rabbits is presented in Fig 12. Microscopic examination of the kidney sections from the healthy control group (T0) revealed a well-preserved renal parenchyma. This was characterized by a distinct glomerular architecture (G) with a clear Bowman’s space (US) and clearly differentiated proximal (PT) and distal (DT) convoluted tubules, indicating normal physiological filtration and reabsorption functions.

In contrast, the infected-untreated cohort (T1) exhibited significant histopathological derangement. The infection induced a localized inflammatory response marked by conspicuous leukocyte infiltration (IL) and mesangial expansion within the glomerulus. These alterations are indicative of acute renal distress and the systemic inflammatory burden caused by the pathogenic *E. coli* strain. However, therapeutic intervention with the combined *P. guajava* and *T. diversifolia* extracts effectively mitigated these structural damages. The renal sections from the T3 (400 mg/kg) and T4 (800 mg/kg) groups, alongside the Neomycin-treated cohort (T2), demonstrated a restoration of the renal tissue architecture. While the 800 mg/kg dose (T4) showed minor residual leukocyte infiltrates, the overall prevention of mesangial expansion and tubular disruption confirms the extract’s nephroprotective potential. This structural preservation is consistent with the biochemical results, where the extract successfully normalized serum creatinine and uric acid levels, confirming that the formulation prevents the acute kidney injury (AKI) typically associated with severe colibacillosis.

### Protective effect of extracts on hepatic microarchitecture

**Fig 13:**
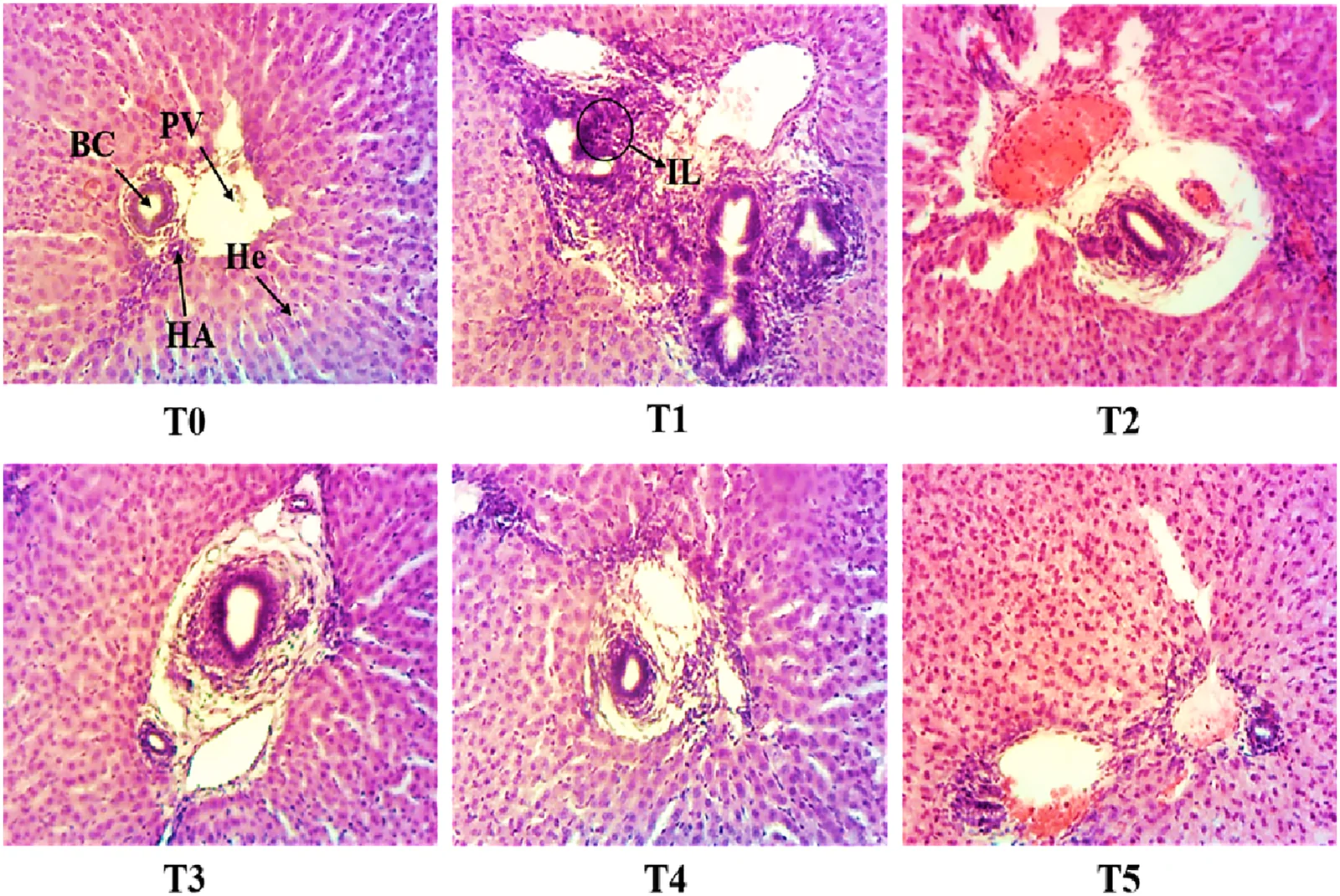
Photomicrographs of rabbit liver sections following *Escherichia coli* challenge and treatment with the combined *Psidium guajava* and *Tithonia diversifolia* extracts (hematoxylin and eosin (H&E) stain; original magnification ×100; scale bar = 10 µm). Group designations (T0–T5) are as defined in the Fig 12 legend; n = 4 livers examined per group; one representative field is shown per group. Abbreviations: Cd, cell degeneration; He, hepatocytes; HA, hepatic artery; PV, portal vein; BC, bile canaliculus; IL, leukocyte infiltration. *Note: Histopathological evaluation was descriptive/qualitative (see* Fig 12 *legend and Discussion, Limitations of the Study).*

The histological evaluation of the liver (Fig 13) confirms the organoprotective properties of the combination of *P. guajava* and *T. diversifolia* during acute *E. coli* infection. Microscopic analysis of the liver from the healthy control group (T0) showed a classical hexagonal lobular arrangement with a well-preserved hepatic parenchyma. The sections exhibited a distinct portal triad comprising the hepatic portal vein (PV), hepatic artery (HA), and bile canaliculus (BC) surrounded by radiating cords of well-defined, polyhedral hepatocytes (He) with prominent nuclei.

In the infected-untreated cohort (T1), the hepatic architecture was compromised by a localized inflammatory response, characterized by significant leukocyte infiltration (IL). This infiltration suggests that the systemic *E. coli* infection induced a degree of hepatitis or endotoxin-mediated tissue stress. However, administration of the combined *P. guajava* and *T. diversifolia* extracts formulation at both 400 mg/kg (T3) and 800 mg/kg (T4) successfully mitigated these inflammatory changes. The hepatic sections of the treated groups showed a restoration of the hepatocellular cords and a marked absence of inflammatory clusters, appearing histologically comparable to the Neomycin-treated (T2) and healthy control (T0) groups.

This structural preservation directly correlates with the enzymatic profiles discussed earlier; by preventing the infiltration of inflammatory cells and maintaining the integrity of the hepatocellular membranes, the extract effectively prevented the leakage of transaminases (AST and ALT) into the systemic circulation. These findings reinforce the dual role of the combination of *P. guajava* and *T. diversifolia* as both an antimicrobial and a potent hepatoprotective agent.

### Restorative effects of extracts on intestinal (Caecal) microarchitecture

**Fig 14:**
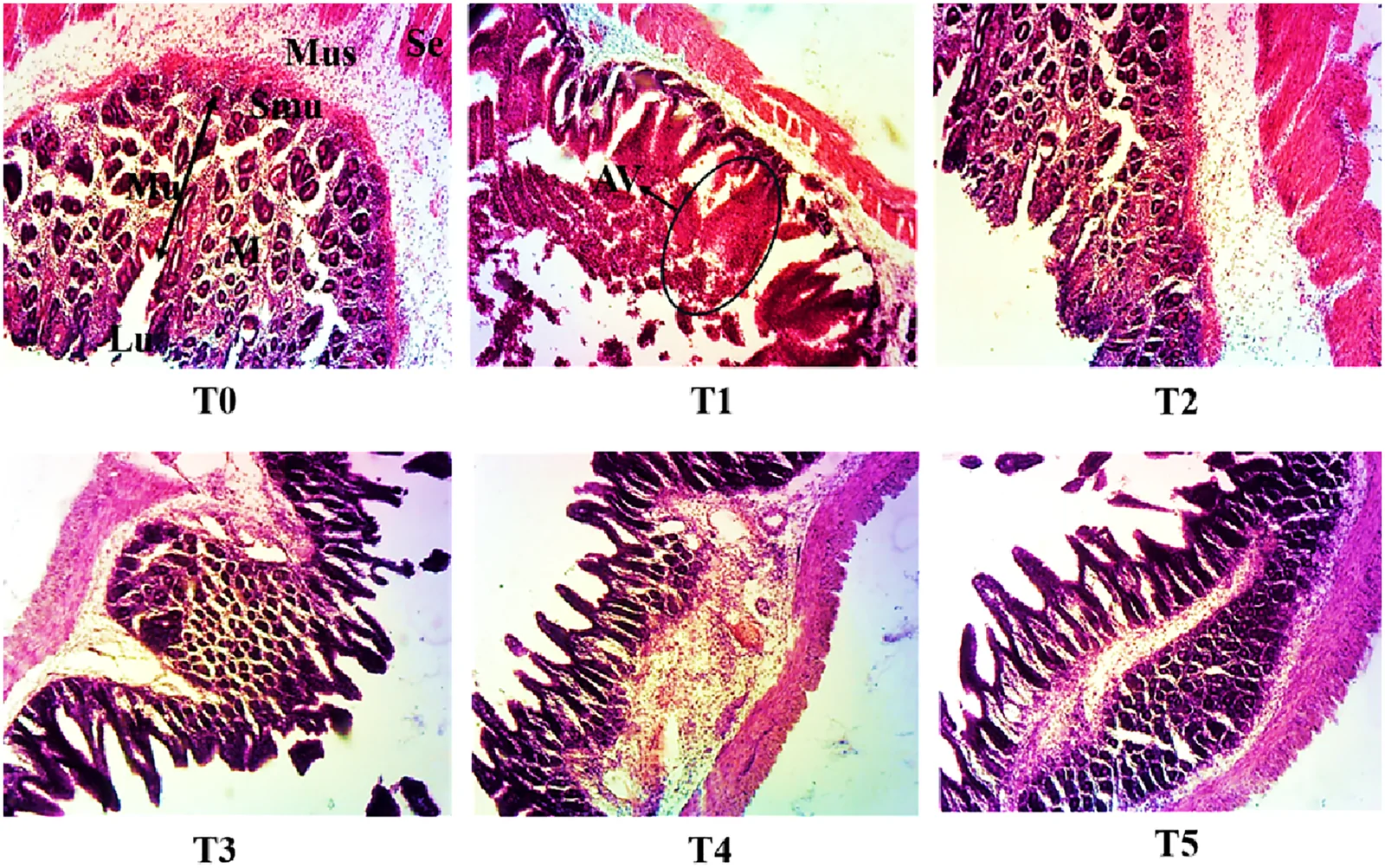
Photomicrographs of rabbit small intestine sections following *Escherichia* *coli* challenge and treatment with the combined *Psidium guajava* and *Tithonia diversifolia* extracts (hematoxylin and eosin (H&E) stain; original magnification ×100; scale bar = 10 µm). Group designations (T0–T5) are as defined in the Fig 12 legend; n = 4 intestinal segments examined per group; one representative field is shown per group. Abbreviations: Cd, cell degeneration; Lu, intestinal lumen; Mu, mucosa; Smu, submucosa; Mus, muscularis; Se, serosa. *Note: Villus height and crypt depth were not measured quantitatively; evaluation was descriptive/qualitative (see* Fig 12 *legend and Discussion, Limitations of the Study).*

The impact of the *E. coli* challenge and the subsequent therapeutic intervention on the caecal microarchitecture is presented in Fig 14. Histological analysis of the caecal sections from healthy control rabbits (T0) revealed a robust and well-organized intestinal wall. The architecture followed a classic structural hierarchy, consisting of the serosa (Se), muscular layer (Mus), submucosa (Smu), and a well-developed mucosa (Mu) lining the intestinal lumen (Lu).

In the infected-untreated cohort (T1), the *E. coli* challenge induced severe mucosal degeneration. The sections exhibited a distinct narrowing of the intestinal mucosa and marked villous atrophy. This loss of structural height and complexity in the mucosal layer indicates a depletion of goblet cell function (leading to decreased mucus secretion) and a reduction in the total absorptive surface area. Such morphological damage provides a structural explanation for the persistent diarrhoea and negative growth performance observed in structurally comparable to the healthy controls and the Neomycin-treated cohort (T2). By maintaining the mucosal barrier and preventing villous blunting, the high-dose extract (800 mg/kg) preserved the intestinal function, facilitating nutrient absorption and preventing the translocation of enteric pathogens. This structural recovery serves as the biological foundation for the observed resolution of clinical diarrhoea and the restoration of the animals’ growth trajectory.

## Discussion

Even though rabbit meat continues to be considered a niche product, demand for it is increasing steadily in various parts of the world, particularly in Asia [26]. Beyond this expanding market, rabbit farming also offers advantages in social equity [27] and environmental sustainability [2]. Despite this potential, the industry, especially in developing nations, is severely constrained by enteric pathologies, most notably colibacillosis caused by pathogenic strains of *E. coli*. This disease compromises animal health and productivity through high morbidity and mortality, and poses a critical One Health risk because the transmission of MDR strains through the food chain represents a growing public health threat [28,29]. As the efficacy of conventional antimicrobials wanes with the global rise of AMR, the exploration of plant-based therapeutic alternatives has become a pharmacological necessity. Our previous ethnopharmacological screening of Cameroonian flora identified *P. guajava* and *T. diversifolia* as potent inhibitors of pathogenic *E. coli*, with their combination exhibiting a synergistic reduction in Minimum Inhibitory Concentration of up to 64-fold [19]. Building on that *in vitro* evidence, the present study evaluated the *in vivo* curative potential and systemic restorative effects of the combined *P. guajava* and *T. diversifolia* extract in *E. coli*-infected rabbits.

The therapeutic efficacy of the combined extract was assessed in colibacillosis-infected rabbits, following the induction protocol of Pouofo et al. [30]. The treatment was conducted for 7 days, during which the bacterial shedding was used to monitor the treatment. By the end of the treatment period, the various parameters evaluated to determine the extract’s therapeutic efficacy, including body weight, relative organ weight, bacterial shedding, haematological, biochemical, inflammatory, and oxidative markers, as well as histopathological findings, showed comparable values between T5 (immunosuppression group) and the healthy control group T0 (uninfected, untreated, and free of cyclophosphamide/streptomycin). This finding indicates that the immunosuppressive effect of cyclophosphamide was reversible and had no measurable lasting impact under our experimental conditions. Therefore, the variation observed in the infected groups could be attributed to the infection and/or extract treatment rather than to pretreatment toxicity.

The experimental induction of colibacillosis produced a profound clinical decline in the untreated cohort, characterized by acute weight loss, dehydration, and high-titer fecal shedding of Congo red–positive *E. coli*. This pathological state is consistent with the capacity of pathogenic *E. coli* to disrupt intestinal water transport and induce catabolic stress [31]. The reversal of these symptoms following administration of the combined extract, particularly at 800 mg/kg demonstrates clear therapeutic efficacy. Bacterial clearance by Day 5 of treatment by combined extract is notable and comparable to the fast-killing activity observed with Neomycin. This bacterial clearance is consistent with previously observed antimicrobial activity of these extracts and their combination [19,32].

Our LC-MS/MS profiling of the two extracts identified pedunculagin, an ellagitannin, in *P. guajava*, together with the flavonoid’s quercetin, quercetin-4′-O-glucoside, myricetin-3-O-pentoside, and 2′-hydroxy-α-naphthoflavone in *T. diversifolia* (S8 Table in S1 File). In fact, tannins and flavonoids of *P. guajava* have been reported to interact with bacterial cell-wall proteins and inhibit *E. coli* growth. Dhiman et al. [33], showed that the methanolic extract *P. guajava* leaf exhibited antibacterial activity against *E. coli* with a minimum inhibitory concentration of 0.78 μg/ml and minimum bactericidal concentration of 50 μg/ml and the phytochemical screening revealed flavonoids, steroids, and tannins as the likely antimicrobial contributors. However, Pereira et al. [34] also found that an *aqueous P. guajava* leaf extract, contain flavonoid and tannin classes (rutin, hesperidin, quercetin). Recently, El-Deeb et al. [32] conducted a comprehensive assessment of *P. cattleianum* aerial-part polyphenols and found that, beyond producing significant antidiarrheal effects in a castor oil-induced diarrhoea model, the extract also showed direct activity against several diarrhoea-associated pathogens, with *in silico* docking implicating the bacterial fatty-acid synthesis enzyme FabH and the kappa opioid receptor as plausible molecular targets. An independent chemical characterization of an aqueous leaf extract of *T. diversifolia* by Dongmo et al. [35] reported tannins and flavonoids, together with phenols, alkaloids, terpenoids, and saponins, thereby supporting the findings of our metabolomics annotation..

The septic hemogram observed in the untreated group, marked by leucocytosis, thrombocytosis, and secondary anaemia, reflects the systemic inflammatory burden of colibacillosis, consistent with findings reported by Petrov et al. [36] in both spontaneous and experimental colibacillosis in weaned rabbits. The normalization of these parameters in all treated groups (T2–T4) indicates resolution of systemic stress. The extract lowered circulating TNF-α, IL-6, and IL-1β and returned CRP to near-normal levels, consistent with an immunomodulatory rather than a purely immunosuppressive action. Phytochemicals from both plant sources may plausibly contribute to this effect.

Recent evidence from a carrageenan-induced inflammation study in Wistar rats provides strong support for the anti-inflammatory potential of *P. guajava*. In that study, an ethanolic bark extract significantly reduced TNF-α, IL-6, and CRP levels compared with those observed in untreated controls. Molecular docking analysis further suggested that constituents of the extract may interact with proteins involved in inflammatory pathways [37]. In carrageenan-induced inflammation models, Broering et al. reported that an extract of *T. diversifolia* reduced the secretion of TNF, IL-1β, IL-6, and nitrite, while also inhibiting neutrophil chemotaxis and CD18 expression [38]. However, specific mechanisms such as NF-κB inhibition, direct cytokine receptor modulation, ROS scavenging, membrane stabilization, or inhibition of bacterial adhesion were not directly evaluated in the present study, therefore remain as plausible literature-supported hypotheses rather than demonstrated mechanisms. Unlike antibiotics, which primarily target the pathogen, this combined extract of *P. guajava* and *T. diversifolia* actively manages the host’s immune response, preventing the tissue-damaging effects of chronic inflammation.

The hypoproteinaemia and hypoalbuminemia observed in Group T1 are consistent with a negative acute-phase response, in which the liver shifts protein synthesis toward inflammatory mediators such as CRP at the expense of albumin [39], a pattern previously reported in *E. coli* infection [40,41]. The concurrent rise in AST and GGT points to hepatocellular leakage, while elevated uric acid suggests impaired renal clearance or accelerated purine catabolism secondary to tissue damage. The extract’s ability to restore these metabolic markers, corroborated by our histological findings of preserved portal triad architecture and tubular integrity, is consistent with a hepatoprotective and nephroprotective effect [42].

At the primary site of infection, colibacillosis-induced oxidative stress (elevated MDA, depleted SOD/CAT) plausibly drove the mucosal damage observed histologically, with villous atrophy and mucosal narrowing providing a physical basis for the malabsorption seen in untreated animals. The tannins and flavonoids identified in both extracts are consistent with a protective, astringent mechanism in which these compounds could coat and stabilize the mucosal surface, limiting further epithelial damage. That could plausibly contribute to the restored villous height and mucosal thickness observed in treated groups [43]. This mechanism was not directly tested in the present study and remains a hypothesis consistent with the compositional and histological findings.

The multivariate analyses (PCA and hierarchical clustering) show that, within this exploratory dataset, the 800 mg/kg dose cohort clusters closer to the healthy and Neomycin-treated groups than to the untreated group. Given the small per-group sample size (n = 4) relative to the number of variables analysed, and the absence of an independent validation cohort, these multivariate results should be interpreted as purely descriptive. Overall, the combined extract shows antimicrobial, immunomodulatory, antioxidant, and organ-protective effects comparable to Neomycin at 800 mg/kg, supporting further mechanistic and pharmacokinetic investigation as a candidate alternative for colibacillosis management.

## Limitations of the study

- The chemically induced infection model (cyclophosphamide + streptomycin) is a manipulated, opportunistic model rather than a natural infection; the T5 control indicates pretreatment effects had resolved by the study endpoint, but early interactions between immunosuppression and infection remain a confounding variable that non-chemical, age-susceptible models could better address.
- The small sample size (n = 4–5/group), while adequately powered for the large primary effects observed (a priori Cohen’s f ≥ 0.70, power = 0.80), limits detection of subtler effects and may not capture the full range of biological variability in outbred rabbits.
- Histopathological evaluation was qualitative, without semi-quantitative lesion scoring or morphometric measurement (villus height, crypt depth), due to equipment constraints; the polarized group differences were visually clear but not numerically graded.
- PCA/heatmap analyses are exploratory given the sample-to-variable ratio and lack an independent validation cohort.
- Mechanistic claims regarding NF-κB inhibition, receptor binding, membrane disruption, or adhesion inhibition are hypotheses drawn from phenotypic and phytochemical data, not directly tested pathways; future work (bioactivity-guided fractionation, adhesion assays, pharmacokinetics, dedicated toxicology, and non-chemical infection models) is needed to confirm them.

## Conclusion

The present study demonstrates the *in vivo* efficacy of the combined *P. guajava* and *T. diversifolia* extracts in mitigating *E. coli* infection in a rabbit model. In infected, immunocompromised rabbits, the combined extract at 800 mg/kg was associated with resolution of the clinical signs of colibacillosis, including restored body-weight gain and a reduction in faecal bacterial shedding to near-undetectable levels, alongside repair of infection-induced tissue damage and no overt signs of toxicity within the parameters assessed in this study. Treated animals showed biochemical, haematological, and antioxidant profiles approaching those of healthy controls, though consistent with the exploratory nature of our multivariate analysis this should be read as a marked improvement toward baseline rather than confirmed physiological equivalence. These findings indicate that the combined *P. guajava*–*T. diversifolia* extract is a promising candidate for managing colibacillosis in rabbits, warranting further development. By addressing both the pathogen and the host’s systemic response, this combination represents a potentially sustainable approach to colibacillosis control amid rising antimicrobial resistance, consistent with One Health-aligned veterinary practice.

Before translational application, several steps remain. Phytochemical fingerprinting and standardization of the formulation are needed to support pharmacological interpretation and reproducibility. Evaluation against a broader panel of multidrug-resistant strains would clarify the extract’s relevance to other enteric infections, and 16S rRNA metagenomic profiling would clarify its impact on commensal intestinal microbiota relative to conventional aminoglycosides. Critically, dedicated pharmacokinetic, dose-optimization, and long-term toxicology studies, together with larger confirmatory trials, are required before the clinical or field-level viability of this formulation can be established.

## Supporting information

S1 File This supporting information provides comprehensive data related to the antibacterial and therapeutic potential of combined aqueous extracts of Psidium guajava (bark) and Tithonia diversifolia (leaves) in a rabbit model of colibacillosis. Fig S1 shows red-pigmented colonies on the Congo Red binding assay (CR+), indicating biofilm formation. Table S1 details the API 20E biochemical profile used to confirm the identity of the isolated E. coli strain. Tables S2 and S3 present the in vivo efficacy data, illustrating the effects of the combined extracts on body weight variation and the dynamics of faecal E. coli shedding over time. Tables S4 and S5 evaluate the physiological impact of treatments by measuring oxidative stress biomarkers and haematological parameters, respectively. Table S6 reports the immunomodulatory effects by quantifying cytokine levels (TNF-α, IFN-γ, IL-10, IL-6, IL-1β, and CRP). Table S7 provides key biochemical parameters (ALAT, ASAT, GGT, triglycerides, cholesterol, HDL, and LDL) to assess hepatic and metabolic function. Finally, Table S8 presents the LC‑MS/MS phytochemical profiling, listing putatively annotated compounds from both plant extracts, including their retention times, molecular formulae, molecular weights, and compound classes. All quantitative data are expressed as mean ± SEM, with statistical significance denoted as *p < 0.05, **p < 0.01, ***p < 0.001, and ****p < 0.0001.
(DOCX)
