# Supplementary material for: A combined aqueous extract of Psidium guajava L. and Tithonia diversifolia (Hemsl.) A. gray improves metabolic homeostasis and preserves intestinal integrity in experimental rabbit colibacillosis
Source: PLoS One. 2026 Sep 15;21(9):e0357951. doi: 10.1371/journal.pone.0357951 (PMC13577389; doi:10.1371/journal.pone.0357951)
Supplement: Supplementary file 2 [file pone.0357951.s005.pdf]

PLOS One

A Combined Aqueous Extract of Psidium guajava L. and Tithonia diversifolia (Hemsl.) A. Gray Improves Metabolic Homeostasis and Preserves Intestinal Integrity in Experimental Rabbit Colibacillosis

--Manuscript Draft--

**Manuscript Number:**	PONE-D-26-07694R1
**Article Type:**	Research Article
**Full Title:**	A Combined Aqueous Extract of Psidium guajava L. and Tithonia diversifolia (Hemsl.) A. Gray Improves Metabolic Homeostasis and Preserves Intestinal Integrity in Experimental Rabbit Colibacillosis
**Short Title:**	Natural Products Restore Metabolic Homeostasis and Intestinal Integrity in Experimental Rabbit Colibacillosis
**Corresponding Author:**	Fabrice Fekam Boyom, Ph.D.<br>University of Yaounde I Faculty of Sciences: Universite de Yaounde I Faculte des Sciences<br>Yaounde, CAMEROON
**Keywords:**	Escherichia coli, Colibacillosis, Antimicrobial Resistance, Psidium guajava, Tithonia diversifolia, Rabbits
**Abstract:**	Background<br>Colibacillosis, caused by pathogenic Escherichia coli, remains a significant threat to rabbit production, particularly during the critical weaning period. With the emergence of multidrug-resistant strains limiting conventional antibiotic efficacy, there is an urgent need for sustainable phytotherapeutic alternatives. This study evaluated the therapeutic efficacy of a combined aqueous leaf extract of Psidium guajava L. and Tithonia diversifolia (Hemsl.) A. Gray on systemic biological markers and organ integrity in a rabbit model of colibacillosis.<br>Methods<br>Thirty healthy rabbits were randomly grouped into six cohorts: negative control (infected-untreated), healthy control (uninfected-untreated), positive control (infected-treated with Neomycin 10 mg/kg), immunodepressed (uninfected-cyclophosphamide), and two treatment groups (infected; receiving 400 or 800 mg/kg of the combined P. guajava and T. diversifolia extract). Colibacillosis was induced via oral administration of a pathogenic E. coli isolate (1 mL/100 g body weight; 10^9 CFU/mL). Systemic recovery was assessed through hematological, biochemical, and antioxidant profiling, alongside histological examination and multivariate Principal Component Analysis (PCA).<br>Results<br>E. coli infection triggered severe haematological, biochemical, and inflammatory dysregulation, characterized by significant increases in white blood cell counts, aspartate aminotransferase, creatinine, and triglycerides, concomitant with reduced red blood cell counts, haemoglobin, and albumin. The combined extract demonstrated dose-dependent efficacy. Notably, multivariate PCA mapping revealed that the 800 mg/kg dose achieved a superior therapeutic outcome, effectively resolving clinical diarrhoea and restoring biological profiles to states comparable to those of the healthy and Neomycin-treated cohorts. Furthermore, the extract significantly mitigated systemic oxidative stress and protected organ integrity.<br>Conclusion<br>These findings demonstrate the therapeutic efficacy and potential of the combined P. guajava and T. diversifolia extract in managing colibacillosis and associated systemic inflammation in rabbits. This study supports the traditional ethnomedicinal use of these plants and demonstrate that these combined extracts could be develop as natural alternatives to synthetic antibiotics in veterinary medicine for managing colibacillosis in rabbits.
**Order of Authors:**	Mireille Tuedom Chouegouong
	Rufin Marie Toghueo Kouipou, Ph.D.
	Yanick Kevin Melogmo Dongmo

Powered by Editorial Manager® and ProduXion Manager® from Aries Systems Corporation

	Jaurès Marius Tsakem Nangap
	Michele Stella Majoumouo, Ph.D.
	Brice Pinlap
	Kingsley Agbor Etchu
	Fabrice Fekam Boyom, Ph.D.

Response to Reviewers:

Response to Reviewers' Comments

Comments reviewers and editor: We thank the Editor and the three Reviewers for their constructive evaluation of our manuscript. Based on they inputs and recommendations, we have revised the entire manuscript, remade all figures and figure captions. Below, we tried to the best of our ability to respond point-by-point to every comment. Reviewer comments are reproduced in bold italics; our responses follow in red.

Slight change in the title: The original title was revised to align with the revised manuscript. The new title is: A Combined Aqueous Extract of Psidium guajava L. and Tithonia diversifolia (Hemsl.) A. Gray Improves Metabolic Homeostasis and Preserves Intestinal Integrity in Experimental Rabbit Colibacillosis

Manuscript ID: PONE-D-26-07694
Reviewer #1
The manuscript is interesting, but some corrections need to be addressed.
Comment 1: The volume of the bacterial suspension used for induction should be included.
Response 1: The disease was induced by administering 1 mL /100 g of body weight of bacterial suspension, adjusted to a 109 CFU/mL in 0.9% saline. This information has been added to the Materials and Methods section.
Comment 2: The software used for data analysis should also be included.
Response 2: Data were analysed using GraphPad Prism and OriginPro, and multivariate analysis was performed in the R environment. This information has been added to the corresponding Methods section (see also Reviewer #2, Comment 10, for the full software citation).
Comment 3: The term “polyherbal extract” should be replaced with “the combined extract of …”.
Response 3: The term “polyherbal extract” has been replaced throughout the manuscript with “the combined Psidium guajava and Tithonia diversifolia extracts,” as suggested.
Comment 4: The preparation of the combined extract should be described, and the rationale for the doses used in the experiment should be explained (the doses appear high).
Response 4: The preparation of the combined extract has been fully described in the Methods section. Briefly, the two extracts were combined in a 96.975% P. guajava : 3.025% T. diversifolia ratio (w/w), determined from the synergistic Fractional Inhibitory Concentration Index (FICI = 0.265) established in our prior in vitro work (Chouegouong et al., 2023; doi:10.1007/s13596-021-00615-1). Fresh stock solutions were prepared daily in sterile distilled water at 40 mg/mL and 80 mg/mL to deliver 400 and 800 mg/kg bw, respectively. The administration was done by oral gavage at a fixed volume of 10 mL/kg bw (i.e., 387.9 mg/kg P. guajava + 12.1 mg/kg T. diversifolia for the low dose, and 775.8 mg/kg P. guajava + 24.2 mg/kg T. diversifolia for the high dose). The selected doses (400 and 800 mg/kg bw) are consistent with standard pharmacological evaluations of crude phytotherapeutic agents in lagomorph models, as previously reported by Hirudkar et al. (2020); this rationale, together with the full preparation protocol, has been added to the Methods section.

Comment 5: Most sentences should be structured appropriately; sentences should not begin with “to” or “by”.
Response 5: Constructions beginning with “To” or “By” have been revised throughout the manuscript to improve phrasing and fluency.
Comment 6: The explanations of the results obtained do not accurately reflect the results or experiments performed; there is considerable overstatement.
Response 6: The results section has been rephrased to remove overstatement and to ensure that the interpretations presented are directly supported by the data obtained.

Powered by Editorial Manager® and ProduXion Manager® from Aries Systems Corporation

Comment 7: The conclusion should be rewritten to accurately reflect the work done, both in the abstract and throughout the manuscript.
Response 7: The conclusion has been rewritten in both the abstract and the main text to align precisely with the results obtained (see also Reviewer #2, Comment 12, and Reviewer #3, Minor Comment 6).
Comment 8: The manuscript should be rewritten to better reflect the results obtained; the figures are confusing for the reader.
Response 8: The manuscript has been revised to better reflect the data generated, all figures are now explicitly cited and described within the text, and every figure legend has been rewritten to be self-contained and self-interpretable (see the accompanying Revised Figure Legends document, and Reviewer #2, Minor Comment 4).
Comment 9: The manuscript contains some inconsistencies, which have been highlighted.
Response 9: Thank you to all reviewers for flagging these inconsistencies. We have revised the entire manuscript and checked all results individually against the raw data and corrected; the corresponding changes are marked in the revised manuscript.
Reviewer #2
Comment 1: Some grammatical, alignment, and typographical errors are noted in the manuscript; it should be thoroughly checked and corrected throughout.
Response 1: We thank the Reviewer for this observation. We have carefully re-read the entire manuscript and performed a thorough revision to correct all grammatical, alignment, and typographical errors identified.
Comment 2: The use of abbreviations in the abstract may distract readers who wish to quickly skim several publications before deciding to read one in full. It may help to write terms out in full in the abstract (for example, AST).
Response 2: We have revised the abstract to write out all abbreviated terms in full.
Comment 3: Abbreviations should be checked throughout the manuscript: the full term should be introduced at first mention in the abstract and again at first mention in the main text, after which only the abbreviation should be used (for example, IL-1β, IL-6, superoxide dismutase (SOD), etc.). Only terms repeated at least three times in the text should be abbreviated.
Response 3: We have thoroughly reviewed all abbreviations throughout the manuscript, introducing each full term at first mention before subsequent use of the abbreviation, and removing abbreviations for terms that did not meet the repetition threshold. The corresponding revisions have been incorporated into the updated manuscript and into the revised figure legends (see accompanying document).
Comment 4: The full species name should be given at first mention in both the abstract and the main text, followed thereafter by the abbreviated genus initial (for example, Psidium guajava / Tithonia diversifolia at first mention, followed by P. guajava / T. diversifolia); this should be checked for all species used in the manuscript.
Response 4: We have revised the entire manuscript and edited the full species of the two plants.
Comment 5: The time (season) of collection of the plant species should be added, since the collection period is known to affect phytochemical constituents; this should be given top priority.
Response 5: We completely agree with the reviewer that transparency in botanical processing is fundamental for reproducibility. We have now updated the "Ethnobotanical Selection and Extract Preparation" section to include the exact exact collection dates and season for both plant species, the starting fresh weights, resulting dry powder weights, and calculated fresh-to-dry ratios for both species.
Comment 6: The quantity of fresh plant material used and the quantity of powder obtained (i.e., the fresh-to-powder ratio) should be included under Materials and Methods.
Response 6: We have added the quantities of fresh plant material and the corresponding dry powder yield to the Materials and Methods section.
Comment 7: The standard diet used in the study should be described, including its composition and source.
Response 7: Information regarding the composition of the standard diet has been incorporated into the revised manuscript.
Comment 8: In the Materials and Methods, references should be cited for any standard protocols or manufacturer instructions used, and the corresponding citations should be added to the reference list.
Response 8: The Methods section has been updated to include references for the standard protocols used, and the corresponding citations have been added to the

reference list.
Comment 9: The dose selected for the present study should be justified in the Materials and Methods, either based on the available literature or a previous study; if derived from another study, it should be supported with the appropriate reference.
Response 9: The dose justification, supported by the relevant reference (Chouegouong et al., 2023; Hirudkar et al., 2020), has been incorporated into the revised Materials and Methods section (see also Reviewer #1, Comment 4).
Comment 10: The authors should cite the software fully when referring to it. GraphPad Prism version 8.0.1 (GraphPad Software, San Diego, CA, USA).
Response 10: We have updated the Methods section to include this full software citation.
Comment 11: Necessary expansions should be given in the figure legends, for example for MDA, SOD, CAT, and GSH.
Response 11: We have rewritten every figure legend so that all abbreviations (including MDA, SOD, CAT and GSH) are expanded at first mention within each individual legend, in line with journal style for self-contained legends. Please see the accompanying Revised Figure Legends document.
Comment 12: The conclusion provides a comprehensive overview of the work but could be more realistic in relation to the evidence presented, to better convey the outcome of the investigation. The authors may also wish to include the limitations of the present findings.
Response 12: We have revised the conclusion to be more precise and directly grounded in the evidence generated by the present study, and we have added a dedicated statement on the limitations of the study and on future investigations, as recommended (see also Reviewer #3, Comments A–D, H, and Minor Comment 6).
Reviewer #3
Major Comments
Comment A — Rationale for the infection model and experimental design: The authors induced infection after administering both cyclophosphamide and streptomycin prior to E. coli inoculation. This creates a highly artificial disease model. Are the observed pathological changes truly due to E. coli infection, or are they partly due to cyclophosphamide-induced immunotoxicity and streptomycin-induced dysbiosis? The rationale for combining cyclophosphamide and streptomycin should be clearly explained.
Response A1: We thank the Reviewer for raising this critical point. We agree that pretreatment with cyclophosphamide and streptomycin adds complexity to the physiological baseline; however, this combination follows a well-established, highly reproducible methodological precedent for inducing an acute, standardised intestinal E. coli infection in adult rabbits (Nguiam et al., 2021), for two complementary reasons: (1) streptomycin transiently clears the facultative anaerobic Gram-negative gut microflora that would otherwise confer strong colonisation resistance, opening an ecological niche that allows the challenge strain to colonise and proliferate; and (2) cyclophosphamide induces transient immunosuppression that mimics the opportunistic susceptibility seen in young, weanling, or stressed animals in the field, ensuring a reliable, synchronised onset of acute clinical colibacillosis across cohorts. This rationale has been added to the revised Methods section.
Addressing Potential Confounding Pathological Changes:
To determine whether the pathological changes observed were attributable to the infection itself rather than to the pretreatment, we included a dedicated immunosuppression-only control group (Group T5), which received the same cyclophosphamide pretreatment but was not inoculated with E. coli (see Response A2 below for the exact T5 regimen). By Day 7 post-infection (9 days post-pretreatment), Group T5 showed no significant differences ($P > 0.05$) from the healthy, untreated control group (T0) in clinical scores, body weight gain, hematological profile, or tissue histopathology, whereas the infected, untreated group (T1) exhibited severe diarrhoea, weight loss, systemic inflammation, and marked histopathological damage. This parity between T5 and T0 indicates that the physiological and immunological perturbations caused by the pretreatment were transient and largely resolved by the study endpoint, supporting the interpretation that the pathology documented in the infected groups reflects the E. coli challenge.
We nonetheless agree that the reliance on chemically induced susceptibility is an inherent limitation of this model, and we have added this point explicitly to the new "Limitations of the Study" subsection of the Discussion.
Comment - The manuscript includes an “immunosuppression control” group (T5),

without a sufficiently detailed description of the treatment regimen. The authors should clarify whether T5 received cyclophosphamide alone or both cyclophosphamide and streptomycin.
Response A2: We confirm that Group T5 (Immunosuppression Control) received cyclophosphamide alone and did not receive streptomycin, so as to isolate the biological effects of chemical immunosuppression on hematological profile, oxidative stress, and tissue architecture, independent of antibiotic-induced gut dysbiosis and of the E. coli challenge. We have expanded the Materials and Methods to state explicitly that animals in Group T5 received a single intraperitoneal injection of cyclophosphamide (30 mg/kg bw) 48 hours before the designated infection timeline, did not undergo streptomycin clearance or bacterial inoculation, and received daily oral gavage of vehicle (10 mL/kg bw sterile distilled water) for 7 consecutive days starting 24 hours post-induction, matching the handling stress and administration volume of the treated cohorts.
Comment- The authors need to discuss the limitations of this highly manipulated infection model, as cyclophosphamide itself can induce oxidative stress, haematological changes, and organ toxicity. Accordingly, several reported outcomes may not solely reflect colibacillosis.
Response A3: We agree that cyclophosphamide is a potent alkylating agent capable of inducing transient myelosuppression, systemic oxidative stress, and organ toxicity, and that this is an important limitation of chemically facilitated infection models. To isolate the contribution of cyclophosphamide from that of the infection, we compared Group T5 (cyclophosphamide alone, uninfected) with Group T0 (healthy control) and Group T1 (infected, untreated). By Day 7 post-infection, the hematological, biochemical, and tissue lipid-peroxidation parameters of Group T5 were statistically indistinguishable from those of Group T0 ($P > 0.05$), in clear contrast to the severe, persistent leukopenia, systemic inflammation (elevated TNF-α, IL-6, CRP), oxidative stress (elevated MDA; depleted SOD and catalase), and tissue damage observed in Group T1. This indicates that the acute toxicological effects of cyclophosphamide had largely resolved by the time of sample collection, although we agree that early-stage cyclophosphamide toxicity remains a confounding variable and a limitation of the model. A dedicated “Limitations of the Study” subsection has been added to the Discussion, and the T5-versus-T0/T1 comparison is now presented there explicitly to help the reader distinguish the relative contribution of cyclophosphamide from that of the infection.
Comment B — Lack of phytochemical characterisation and extract standardisation: The major weakness of the study is the absence of phytochemical profiling and standardisation of the two extracts used. The authors repeatedly attribute the biological effects to the presence of flavonoids, tannins, and sesquiterpene lactones, and to NF-κB modulation, without quantification or characterisation of these compounds (e.g., by HPLC, LC-MS/MS, total phenolic/flavonoid quantification, or chromatographic fingerprinting). Otherwise, the mechanistic claims should be substantially toned down.
Response B: Our previous study (Chouegouong et al., 2023) reported the chemical fingerprint of these same two extracts, confirming the presence of several chemical classes, including terpenes, flavonoids, and tannins; this reference has been cited in the revised manuscript. Moreover, we also performer an LC-MS analysis of crude extracts from Psidium guajava L. and Tithonia diversifolia that allow us to annotate Pedunculagin (from P. guajava), and Quercetin, Quercetin 4′-O-glucoside, Myricetin-3-O-pentoside, and 2′-Hydroxy-alpha-naphthoflavone (from T. diversifolia). This new information has been added to the discussion and the supplementary materials. Moreover, all mechanistic statements attributing the observed effects to specific phytochemical classes or to NF-κB modulation have been moderated and reframed as plausible hypotheses consistent with the prior phytochemical characterisation, rather than as demonstrated mechanisms (see also Comment E).
C) Small sample size and statistical power concerns
The study used only n = 5 rabbits/group, with one mortality in the untreated infected group and only four animals/group ultimately analyzed histologically and biochemically. The endpoints measured are quite large in comparison with the number of animals per group. Accordingly, The authors should:
• provide statistical power justification,
• clarify whether normality testing was performed,
• explain how multiple testing inflation was controlled,
• and discuss the limitations imposed by the small sample size.
Response C: We completely acknowledge the reviewer’s valuable methodological

concerns regarding statistical power, sample sizes, and the risk of Type I and Type II errors when evaluating multiple physiological endpoints. We have addressed each point as follows and updated the Statistical Analysis and Limitations subsections accordingly.

1. Statistical power: The initial (n = 5/group) and final analytical (n = 4/group, following early humane euthanasia in Group T1 and the planned reduction of one animal per surviving cohort under the 3Rs framework) sample sizes were set based on the large, polarised effect sizes typically observed between healthy and untreated-infected phenotypes in acute lagomorph enteric infection models. An a priori power analysis indicated that for a large anticipated effect size (Cohen's $f \geq 0.70$), n = 4 animals per group is sufficient to achieve power $(1 - \beta)$ of 0.80 at $\alpha = 0.05$, balancing statistical reliability against the 3Rs (Reduction) principle.
2. Normality testing: Data normality was assessed using the Shapiro-Wilk test, and homogeneity of variance using Levene's test; all datasets satisfied these assumptions ($P > 0.05$), supporting the use of parametric statistical models.
3. Multiple-testing control: To avoid Type I error inflation from repeated pairwise comparisons across six groups, all multi-group variables were analysed by one-way ANOVA followed by Tukey's HSD post-hoc test, which controls the family-wise error rate at $\alpha = 0.05$.
4. Limitations: We acknowledge that, while adequately powered for the large-magnitude primary therapeutic effects observed, the small sample size restricts our ability to detect subtler or secondary effects and may not fully capture natural physiological variability in outbred rabbit populations; this is now explicitly stated in the Limitations of the Study.

D) Absence of quantitative histopathology evaluation

The histopathological analysis is entirely descriptive mentioning “leukocyte infiltration, villous atrophy, restoration,” etc. However, no histological scoring system was used, and no semi-quantitative analysis was provided. The authors should: introduce validated histopathological scoring systems, quantify inflammatory infiltration and villus height/crypt depth, and provide lesion severity scores.

Response D: We completely agree with the reviewer that semi-quantitative scoring and morphometric measurement (e.g., villus height, crypt depth) would add valuable numerical depth. At the time of this study, our facility did not have access to digital histomorphometric software or automated image-analysis systems required for these sub-micron measurements in lagomorph intestinal tissue; the histopathological evaluation was therefore designed as a rigorous, descriptive, qualitative assessment. To limit subjectivity, sections were evaluated by a blinded observer focused on identifying unambiguous, pathognomonic signs of acute colibacillosis (e.g., mucosal desquamation, denuded villi, extensive inflammatory infiltrates). As shown in the revised figures, the structural differences between groups are marked: the infected-untreated group shows extensive destruction of intestinal architecture, while the high-dose extract group shows visibly preserved mucosal integrity comparable to the healthy control. We have added the absence of semi-quantitative scoring as an explicit limitation in the Discussion and clarified the qualitative nature of the histological methodology in the Materials and Methods.

E) Cytokine and mechanistic claims are overstated

The authors repeatedly conclude that the extracts used caused NF-κB inhibition, immunomodulation, ROS scavenging mechanisms, membrane stabilization, and bacterial adhesion inhibition, without experimental evaluation of these pathways. Only general screening parameters were measured. Mechanistic statements should be rewritten more cautiously and presented as hypotheses rather than demonstrated mechanisms.

For example: “may involve modulation of inflammatory pathways”
instead of: “likely mediated through NF-κB inhibition.”

Response E: We have rewritten the Results and Discussion so that mechanistic statements are consistently framed as hypotheses supported by our observed endpoints (e.g., “may involve modulation of inflammatory pathways”) rather than as demonstrated mechanisms (e.g., “likely mediated through NF-κB inhibition”), throughout the manuscript.

Comment F — Important missing methodological details

Several experimental details are insufficiently described, including the following:

Randomisation: the manuscript states that rabbits were “randomly assigned,” but the randomisation method is not described.

Response F1 — Randomisation: We thank the reviewer for pointing out this missing

Powered by Editorial Manager® and ProduXion Manager® from Aries Systems Corporation

methodological detail. We agree that specifying the exact method of randomization is essential for transparency and reproducibility. In the revised manuscript, we have updated the Materials and Methods section to explicitly describe the randomization protocol. Specifically, we have clarified that a simple randomization procedure was performed using a computer-generated random number sequence (https://www.random.org/lists/), ensuring that each animal had an equal and independent probability of being assigned to any of the six experimental cohorts.
Response F2 — Blinding: To eliminate observer and detection bias, a strict blinding protocol was implemented across multiple stages of the study. The investigators conducting daily clinical scoring were blinded to the treatment groups by having an independent colleague manage the preparation and coding of the treatment solutions. Furthermore, all downstream laboratory analyses, including hematological processing, biochemical biomarker assays, and histopathological tissue evaluations were performed by investigators who were completely blinded to the experimental groups. All samples and tissue slides were labeled with anonymous, randomized tracking codes, which were only unblinded after the final statistical analysis was completed.
Sex distribution: the rabbits were of “mixed sex,” but the distribution per group was not provided. Sex-dependent differences may influence the immune response.
Response F3 — Sex distribution: Statistical analysis revealed no significant difference between males and females in infection or treatment response under the present experimental conditions. The sex distribution per group (2 males / 3 females per group) has been added to the revised manuscript.
Extract formulation: the exact ratio between the two combined extracts is not clearly stated.
Response F4 — Extract formulation: Combined extract formulations of P. guajava and T. diversifolia were freshly prepared in distilled water daily. The ratio of the two extracts within the combination (96.975% P. guajava and 3.025% T. diversifolia) was determined based on the synergistic Fractional Inhibitory Concentration Index (FICI = 0.265) identified in our prior in vitro assays (Chouegouong et al., 2023). To administer the target doses of 400mg/kg and 800 mg/kg body weight (bw) at a standardized oral gavage volume of 1 mL per 100 g bw (10 mL/kg bw), two distinct stock solutions were prepared. The stock solution of 40 mg/mL (for the 400 mg/kg group) was prepared by dissolving 310.32 mg of P. guajava extract and 9.68 mg of T. diversifolia extract in 8 mL of sterile distilled water. This delivered 387.9 mg/kg of P. guajava and 12.1 mg/kg of T. diversifolia in vivo. The second stock solution of 80mg/mL (for the 800mg/kg group) was Prepared by dissolving 620.64 mg of P. guajava extract and 19.36 mg of T. diversifolia extract in 8mL of sterile distilled water. This delivered 775.8mg/kg of P. guajava and 24.2 mg/kg of T. diversifolia in vivo.
Comment G: Bacterial characterisation is insufficient. The isolate was identified only by API 20E and the Congo red binding assay. However, the study repeatedly refers to EPEC, virulence, pathogenicity, and attaching/effacing lesions. No molecular confirmation of virulence genes was performed. The authors should avoid definitive classification as EPEC unless molecular virulence markers are confirmed, or should include PCR confirmation for the relevant virulence genes.
Response G: We agree that definitive classification as Enteropathogenic E. coli (EPEC) requires molecular confirmation of diagnostic virulence markers (e.g., eaeA, bfpA) by PCR, which was not performed here; our isolate was characterised only phenotypically, using the API 20E system for species-level identification (Fig 1) and the Congo red binding assay to confirm pathogenic phenotype. We have therefore removed the term “EPEC” throughout the manuscript (Abstract, Introduction, Methods, Results, Discussion) and replaced it with “pathogenic E. coli”, and we have toned down or rephrased speculative references to specific microscopic attaching/effacing lesions to describe generalised enteropathogenic tissue alterations consistent with our phenotypic and histological data.
Comment H: PCA and multivariate analysis appear overinterpreted. The PCA and heatmap analyses are visually attractive, but the sample size is small, the number of variables is large, and no validation strategy is described. Statements such as “biological reset” and “systemic recovery indistinguishable from healthy baseline” are exaggerated given the exploratory nature of PCA. The authors should moderate their interpretation, clarify the variance explained, and discuss the limitations of multivariate modelling with small datasets.
Response H: We agree that applying PCA to a high-dimensional dataset (46 variables) with a small sample size (n = 4/group) without an independent validation cohort carries a substantial risk of overfitting, and that these results must not be overinterpreted. We

Powered by Editorial Manager® and ProduXion Manager® from Aries Systems Corporation

have removed exaggerated or definitive claims (e.g., “mathematically demonstrates”, references to an absolute “biological reset”), clarified the exact variance explained by each dimension (Dim1 = 48%, Dim2 = 9.7%; Fig 10), and explicitly reframed the PCA and heatmap as descriptive, exploratory visualisations rather than predictive or confirmatory models. A statement on the limitations of multivariate analysis with small sample sizes has been added to the Discussion (see also the revised legends for Figs 9 and 10).
Comment I: The discussion following several results sections lacks sufficient referencing and critical comparison with the literature. The authors should discuss their findings in relation to previous studies, emphasising agreements, discrepancies, possible explanations, and remaining research gaps. For example, reports on Psidium species indicate antidiarrheal activity through antimicrobial effects, not solely through tannins (DOI: https://doi.org/10.1016/j.jep.2025.119670).
Response I: We have substantially revised the Discussion to integrate and critically compare our findings with the relevant literature, including the reference suggested by the Reviewer (DOI: 10.1016/j.jep.2025.119670) on antimicrobial, rather than purely tannin-mediated, antidiarrheal mechanisms of Psidium species. All new text is original and properly cited, and the corresponding changes are indicated in the revised manuscript (see also Minor Comment 2).
Minor Comments
Minor Comment 1: Several sections contain overly promotional language inappropriate for scientific writing, including:

- “biological reset”
- “high-activity extract”
- “profound therapeutic trajectory”
- “gold-standard treatment”
- “extinguishing inflammatory cascades.”

The manuscript should adopt a more objective scientific tone.
Response 1: All overstated expressions, including those listed, have been replaced with objective, scientifically appropriate language throughout the manuscript.
Minor Comment 2: The discussion is excessively long and repetitive. Several statements restate the results instead of critically interpreting them (see also Comment I above).
Response 2: To eliminate redundancy, we condensed and refocused the Discussion. The revised text (1) compares our findings with previous studies, (2) proposes plausible mechanistic pathways while explicitly noting their hypothetical status, (3) discusses the constraints of the present study, and (4) outlines specific follow-up experiments (fractionation, adhesion assays, pharmacokinetics, safety/toxicology). References have been reorganised to support this structure.
Minor Comment 3: The authors should clarify whether the units used for ALT and AST, cytokine concentrations, and oxidative stress markers were standardised and validated for rabbit samples (i.e., that the kits used are validated for use with rabbit samples).
Response 3: We have revised the Materials and Methods section to clearly specify the assay validation, species applicability, and units of measurement for all biochemical and immunological parameters.
Minor Comment 4: Figure legends appear insufficiently detailed. They should be revised to be self-interpretable in some sections, including magnification, stain details, scale bars, and statistical annotations.
Response 4: We agree that legends must be fully self-interpretable. All 14 figure legends have been rewritten (see the accompanying Revised Figure Legends document): each now states the sample size (n = 4 animals/group analysed), expands all technical abbreviations at first mention, and specifies the statistical test and software used. For the histopathological figures (Figs 12–14), the legends now specify hematoxylin and eosin staining, original magnification (×100), and a scale bar; because scale-bar values were not recorded in the original image files available to us, these are flagged with a placeholder for the corresponding author to insert before resubmission.
Minor Comment 5: Adherence to the ARRIVE guidelines is mentioned, but several ARRIVE-required elements remain incomplete, namely sample size justification, exclusion criteria, and blinding details.
Response 5: The Methods section now includes an explanation of the sample size (see Response C), the predetermined inclusion/exclusion criteria, and a full description of the blinding procedure at the clinical-scoring, laboratory-analysis, and data-interpretation stages (see Response F2). The updated ARRIVE 2.0 checklist (https://arriveguidelines.org/arrive-guidelines) is submitted as a supplementary

Powered by Editorial Manager® and ProduXion Manager® from Aries Systems Corporation

	submission<br>Minor Comment 6: The conclusion somewhat overstates translational applicability. The statement that the extract is “clinically equivalent to conventional aminoglycosides” is not fully supported without pharmacokinetic data, toxicity studies, dose optimisation, long-term safety data, and field validation. The conclusion must be revised accordingly.<br>Response 6: The conclusion has been rewritten to remove the claim of clinical equivalence to conventional aminoglycosides and to present the translational potential of the extract in a manner consistent with the evidence available (comparable performance on the specific biomarkers measured at Day 7, without pharmacokinetic, long-term safety, or field validation data), in line with the newly added Limitations of the Study.
**Additional Information:**	
**Question**	**Response**
**Competing Interests**<br><br>Use the instructions below to enter a competing interest statement for this submission. On behalf of all authors, disclose any competing interests that could be perceived to bias this work—acknowledging all financial support and any other relevant financial or non-financial competing interests.<br><br>This statement is required for submission and **will appear in the published article** if the submission is accepted. Please make sure it is accurate and that any funding sources listed in your Funding Information later in the submission form are also declared in your Financial Disclosure statement.<br><br>View published research articles from *PLOS ONE* for specific examples.<br><br>**NO authors have competing interests**<br><br>Enter: *The authors have declared that no competing interests exist.*<br><br>**Authors with competing interests**<br><br>Enter competing interest details beginning with this statement:<br><br>*I have read the journal's policy and the authors of this manuscript have the following competing interests: [insert competing interests here]*	The authors have declared that no competing interests exist.

* typeset	
**Data Availability**<br><br>Authors are required to make all data underlying the findings described fully available, without restriction, and from the time of publication. PLOS allows rare exceptions to address legal and ethical concerns. See the PLOS Data Policy and FAQ for detailed information.<br><br>A Data Availability Statement describing where the data can be found is required at submission. Your answers to this question constitute the Data Availability Statement and **will be published in the article**, if accepted.<br><br>**Important:** Stating 'data available on request from the author' is not sufficient. If your data are only available upon request, select 'No' for the first question and explain your exceptional situation in the text box.<br><br>Do the authors confirm that all data underlying the findings described in their manuscript are fully available without restriction?	Yes - all data are fully available without restriction
Additional data availability information:	
**Financial Disclosure**<br><br>Enter a financial disclosure statement that describes the sources of funding for the work included in this submission. Review the submission guidelines for detailed requirements. View published research articles from *PLOS ONE* for specific examples.<br><br>This statement is required for submission and **will appear in the published article** if the submission is accepted. Please make sure it is accurate.	The author(s) received no specific funding for this work.

**Funded studies**<br>Enter a statement with the following details:<br>• Initials of the authors who received each award<br>• Grant numbers awarded to each author<br>• The full name of each funder<br>• URL of each funder website<br>• Did the sponsors or funders play any role in the study design, data collection and analysis, decision to publish, or preparation of the manuscript?<br><br>Did you receive funding for this work?	
**Ethics Statement**<br><br>Enter an ethics statement for this submission. This statement is required if the study involved:<br><br>• Human participants<br>• Human specimens or tissue<br>• Vertebrate animals or cephalopods<br>• Vertebrate embryos or tissues<br>• Field research<br><br>Write "N/A" if the submission does not require an ethics statement.<br><br>General guidance is provided below. Consult the submission guidelines for detailed instructions. **Make sure that all information entered here is included in the Methods section of the manuscript.**	All procedures were approved by the Cameroon National Ethical Committee (Ref N. FWIRB00001954).

Powered by Editorial Manager® and ProduXion Manager® from Aries Systems Corporation

**Format for specific study types**<br><br>**Human Subject Research (involving human participants and/or tissue)**<br>• Give the name of the institutional review board or ethics committee that approved the study<br>• Include the approval number and/or a statement indicating approval of this research<br>• Indicate the form of consent obtained (written/oral) or the reason that consent was not obtained (e.g. the data were analyzed anonymously)<br><br>**Animal Research (involving vertebrate animals, embryos or tissues)**<br>• Provide the name of the Institutional Animal Care and Use Committee (IACUC) or other relevant ethics board that reviewed the study protocol, and indicate whether they approved this research or granted a formal waiver of ethical approval<br>• Include an approval number if one was obtained<br>• If the study involved *non-human primates*, add *additional details* about animal welfare and steps taken to ameliorate suffering<br>• If anesthesia, euthanasia, or any kind of animal sacrifice is part of the study, include briefly which substances and/or methods were applied<br><br>**Field Research**<br><br>Include the following details if this study involves the collection of plant, animal, or other materials from a natural setting:<br>• Field permit number<br>• Name of the institution or relevant body that granted permission	
**Data Availability**<br><br>Before publication, Authors are required to make fully available and without restriction all data underlying their findings. Sharing the data underlying your findings increases the visibility, credibility, and impact of your work, while supporting transparency and future scientific discovery. Please see our PLOS Data	All relevant data are within the manuscript and its Supporting Information files.

Policy page for detailed information on this policy.<br><br>A **Data Availability Statement**, detailing where the data can be accessed, is required at first submission. Insert your Data Availability Statement in the box below.<br><br>Please see the data reporting section of our submission guidelines for instructions on what you need to include in your Data Availability Statement. Examples of acceptable Data Availability Statements are included here for reference:<br>1. The minimal data set [and accompanying code, where generated] is available at [repository name] via [DOI hyperlink]<br>2. No data was generated by this study. The following existing data sources were used: [dataset name] from [dataset location] available via [web address or DOI hyperlink].<br>3. For Study Protocols: No datasets were generated or analysed during the current study. All relevant data from this study will be made available upon study completion.<br><br>*PLOS allows rare exemptions to address legal and ethical concerns. If you have legal or ethical restrictions, please use the box below to detail these in full sentences for the Journal team to consider.*<br><br>This statement will be typeset if the manuscript is accepted for publication.	

Powered by Editorial Manager® and ProduXion Manager® from Aries Systems Corporation

Manuscript

Click here to access/download;Manuscript;Revised_Clean_PONE-D-26-

A Combined Aqueous Extract of *Psidium guajava* L. and *Tithonia diversifolia* (Hemsl.) A. Gray Improves Metabolic Homeostasis and Preserves Intestinal Integrity in Experimental Rabbit Colibacillosis

Mireille Tuedom Chouegouong[1,2], Rufin Marie Kouipou Toghueo[1,3*], Yanick Kevin Melogmo Dongmo[1], Jaurès Marius Tsakem Nangap[4], Michele Stella Majoumouo[1,5], Brice Pinlap[1] Kingsley Agbor Etchu[2], Fabrice Fekam Boyom[1*]

[1]Antimicrobial and Biocontrol Agents Unit (AmBcAU), Laboratory for Phytobiochemistry and Medicinal Plants Studies, Department of Biochemistry, Faculty of Science, University of Yaoundé I, P.O. Box 812, Messa-Yaoundé, Cameroon
[2]Institute of Agricultural Research and Development (IRAD), P.O. Box 2067, Yaoundé, Cameroon
[3]Laboratory for Antibiotic Resistance and Microbial Metabolomics, Institute of Microbiology, Czech Academy of Sciences, Videnska 1083, 142 20 Prague 4, Czech Republic
[4]Laboratory of Animal Physiology, Department of Animal Biology and Physiology, Faculty of Science, University of Yaoundé 1, PO Box 812, Yaoundé, Cameroon
[5]Division of Pulmonary, Critical Care & Sleep Medicine, Department of Internal Medicine, University of Cincinnati College of Medicine

Corresponding: toghueo.rufin@yahoo.fr (RMKT); fabrice.boyom@fulbrightmail.org (FFB)

Abstract

Background

Colibacillosis, caused by pathogenic *Escherichia coli*, remains a significant threat to rabbit production, particularly during the critical weaning period. With the emergence of multidrug-resistant strains limiting conventional antibiotic efficacy, there is an urgent need for sustainable phytotherapeutic alternatives. This study evaluated the therapeutic efficacy of a combined aqueous leaf extract of *Psidium guajava* L. and *Tithonia diversifolia* (Hemsl.) A. Gray on systemic biological markers and organ integrity in a rabbit model of colibacillosis.

Methods

Thirty healthy rabbits were randomly grouped into six cohorts: negative control (infected-untreated), healthy control (uninfected-untreated), positive control (infected-treated with Neomycin 10 mg/kg), immunodepressed (uninfected-cyclophosphamide), and two treatment groups (infected; receiving 400 or 800 mg/kg of the combined *P. guajava* and *T. diversifolia* extract). Colibacillosis was induced via oral administration of a pathogenic *E. coli* isolate (1 mL/100 g body weight; 10^9 CFU/mL). Systemic recovery was assessed through hematological, biochemical, and antioxidant profiling, alongside histological examination and multivariate Principal Component Analysis (PCA).

Results

E. coli infection triggered severe haematological, biochemical, and inflammatory dysregulation, characterized by significant increases in white blood cell counts, aspartate aminotransferase, creatinine, and triglycerides, concomitant with reduced red blood cell counts, haemoglobin, and albumin. The combined extract demonstrated dose-dependent efficacy. Notably, multivariate PCA mapping revealed that the 800 mg/kg dose achieved a superior therapeutic outcome, effectively resolving clinical diarrhoea and restoring biological profiles to states comparable to those of the healthy and Neomycin-treated cohorts. Furthermore, the extract significantly mitigated systemic oxidative stress and protected organ integrity.

Conclusion

These findings demonstrate the therapeutic efficacy and potential of the combined *P. guajava* and *T. diversifolia* extract in managing colibacillosis and associated systemic inflammation in rabbits. This study supports the traditional ethnomedicinal use of these plants and demonstrate that these combined extracts could be develop as natural alternatives to synthetic antibiotics in veterinary medicine for managing colibacillosis in rabbits.

Keywords: *Escherichia coli,* Colibacillosis, Antimicrobial Resistance, *Psidium guajava, Tithonia diversifolia,* Rabbits

Introduction

In the face of escalating global climate change and the urgent need for sustainable food systems, rabbit farming (*Oryctolagus cuniculus*) has emerged as an environmentally superior alternative to traditional livestock [1,2]. Characterized by a low carbon footprint, minimal water requirements, and the ability to thrive on non-competitive forage, the domestic rabbit industry is increasingly recognized as a cornerstone of eco-friendly agriculture and a vital contributor to rural economic development and food security worldwide [2]. Global rabbit meat production reached approximately 860,000 tons in 2021, with China leading the sector (53.1%), followed by a growing importance in developing nations [3]. This prominence of farming is driven by the rabbit's favourable biological characteristics, notably their high fecundity and efficient feed conversion [4]. Furthermore, rabbit meat is a highly nutritious, lean protein source, rich in essential micronutrients such as iron, zinc, and vitamin B12 [5].

Despite this potential, the rabbit farming industry faces substantial health challenges, with enteric diseases representing the most economically devastating hurdle [6]. Infectious

diarrhoea remains a primary cause of mortality and reduced growth performance, leading to high veterinary costs [7]. Colibacillosis, caused by enteropathogenic *Escherichia coli* (EPEC), is the most prevalent bacterial threat to young rabbits [6]. The pathogenesis of EPEC is defined by a sophisticated cytokine cascade: bacterial attachment (BFP) and effector protein injection (T3SS) trigger an immediate innate response (interleukin-1β and interleukin-6), which subsequently activates a Th1-mediated adaptive immune response [8]. The resulting attaching and effacing (A/E) lesions destroy the intestinal brush border, compromising the epithelial barrier and allowing systemic cytokines to exacerbate fluid loss and diarrhoea [8]. Furthermore, this bacterial colonization triggers an intense oxidative stress response. The host's ability to survive this oxidative storm depends heavily on the mobilization of its antioxidant defences system, particularly superoxide dismutase (SOD) and glutathione peroxidase (GPX) [9] .

The conventional management of colibacillosis has historically relied on a narrow spectrum of antimicrobials, predominantly trimethoprim-sulfamethoxazole and fluoroquinolones [10]. However, the livestock sector is now at a critical juncture due to the global escalation of antimicrobial resistance (AMR). Recent surveillance indicates a dramatic rise in multidrug-resistant (MDR) *E. coli* strains in rabbitries, with some isolates exhibiting total resistance to watch-list antibiotics like tetracyclines and aminoglycosides [11,12]. This phenomenon is not merely a veterinary concern, but a significant public health threat, as the transmission of resistant genes from livestock to the human food chain complicates the treatment of enteric infections in people [13,14]. Furthermore, the increasing regulatory restrictions on antibiotic growth promoters (AGPs) and the growing consumer demand for antibiotic-free animal products have rendered the status quo untenable [15,16]. Consequently, there is an urgent imperative to transition toward sustainable, bio-based therapeutic alternatives that can achieve clinical resolution without contributing to the environmental resistome.

Ethnoveterinary medicine offers a robust reservoir for therapeutic discovery, particularly through species such as *P. guajava*. (Myrtaceae) and *T diversifolia* (Asteraceae). While *P. guajava* is widely used for its tannin-rich antidiarrheal properties [17] and *T. diversifolia* for its anti-inflammatory sesquiterpene lactones [18], their selection for this study was based on the results from our previous investigation. In our preceding ethnopharmacological survey of rabbitries in Cameroon, these two species emerged as the most prominent remedies used by local farmers to manage bacterial enteritis [19]. Subsequent *in vitro* screening of forty-five different preparations revealed that only six extracts, exhibited potent antibacterial activity

against pathogenic *E. coli*. Notably, the combination of *T. diversifolia* and *P. guajava* yielded a synergistic interaction, achieving a Fractional Inhibitory Concentration Index (FICI) as low as 0.265 and reducing individual minimum inhibitory concentration (MIC) values by up to 64-fold [19]. This specific formulation demonstrated a high biological potency index, consistently maintaining activity levels that justify further investigation. Consequently, the present study was designed to validate these synergistic findings *in vivo*, utilizing an integrated multivariate approach to determine if the combination of *P. guajava* and *T. diversifolia* extracts can resolve systemic colibacillosis and restore host homeostasis as effectively as conventional antibiotherapy.

Materials and Methods

Ethical Approval, Ethical Statement, and Animal Welfare

All experimental protocols were reviewed and approved by the Cameroon National Ethical Committee (Ref No. FWIRB00001954). The study was conducted in strict accordance with the ARRIVE guidelines (Animal Research: Reporting of In Vivo Experiments).

Ethnobotanical Selection and Extract Preparation

The selection of plant materials was based on our previous ethnopharmacological survey, identifying active remedies against rabbit bacterial enteritis [19].

Fresh leaves of *P. guajava* (Myrtaceae) and *T. diversifolia* (Hemsl.) A. Gray (Asteraceae) were collected in April 2024 (corresponding to the onset of the rainy season) at approximately 07:00 h (local time) in Yaoundé, Cameroon. The botanical samples were authenticated at the National Herbarium of Cameroon (Voucher Nos. 2884 SRFK and 57410 HNC, respectively). The fresh leaves were air-dried at ambient temperature (25°C) for 14 days until a constant weight was achieved, followed by mechanical pulverization into a fine powder. For *P. guajava*, 2204 g of fresh leaves yielded 500 g of dry powder (fresh-to-dry ratio of approximately 4.4:1). For *T. diversifolia*, 3774 g of fresh leaves yielded 500 g of dry powder (fresh-to-dry ratio of approximately 7.5:1).

The extracts were prepared using cold extraction with distilled water at room temperature to mimic the traditional preparation, ensuring high extraction yield. For each species, 500 g of powder was macerated in 2 L of distilled water for 72 h under continuous agitation. The mixtures were filtered through hydrophilic cotton, and the filtrates were dehydrated under

forced ventilation at 25°C to remove the residual solvent. The resulting crude extracts were stored at -20°C.

Preparation of the combined extract solutions

Combined extract formulations of *P. guajava* and *T. diversifolia* were freshly prepared in distilled water daily. The ratio of the two extracts within the combination (96.975% *P. guajava* and 3.025% *T. diversifolia*) was determined based on the synergistic Fractional Inhibitory Concentration Index (FICI = 0.265) identified in our prior *in vitro* assays [19]. To administer the target doses of 400mg/kg and 800 mg/kg body weight (bw) at a standardized oral gavage volume of 1 mL per 100 g bw (10 mL/kg bw), two distinct stock solutions were prepared. The stock solution of 40 mg/mL (for the 400 mg/kg group) was prepared by dissolving 310.32 mg of *P. guajava* extract and 9.68 mg of *T. diversifolia* extract in 8 mL of sterile distilled water. This delivered 387.9 mg/kg of *P. guajava* and 12.1 mg/kg of *T. diversifolia in vivo*. The second stock solution of 80mg/mL (for the 800mg/kg group) was Prepared by dissolving 620.64 mg of *P. guajava* extract and 19.36 mg of *T. diversifolia* extract in 8mL of sterile distilled water. This delivered 775.8mg/kg of *P. guajava* and 24.2 mg/kg of *T. diversifolia in vivo*.

Characterization and Virulence of the Clinical Isolate

A clinical isolate of *E. coli* was recovered from a rabbit presenting acute colibacillosis symptoms. Identification was performed using the API 20E analytical profile index (bioMérieux, France). Virulence potential was assessed via the Congo red binding assay [20] on tryptic soy agar supplemented with 0.003 % Congo red and 1.5 % bile salts. Red-pigmented colonies, indicative of virulent plasmid expression, were selected and cultured in nutrient broth supplemented with bile salts (0.4%). Inoculum turbidity from a 24-hour culture was adjusted to 4 McFarland, corresponding to a concentration of approximately 10^9 CFU/mL in sterile 0.9 % saline [21].

Experimental Animals

Thirty hybrid rabbits (mixed sex: *three females and two males*, 6–7 weeks old, 800–900 g) were acclimatized for 7 days at the Laboratory of Phytobiochemistry and Medicinal Plant Studies, University of Yaoundé I. Animals were housed in pairs in galvanized cages (35 cm × 40 cm × 60 cm) under a 12-hour light/dark cycle. They were provided ad libitum access to water and a standard pelleted diet (composition: 21 kg corn flour, 4 kg cassava flour, 20 kg

Pennisetum sp., 31 kg wheat bran, 8 kg palm kernel cake, 6 kg soybean cake, 1 kg bone meal, 4 kg palm oil, and 5 kg dried fish). Following acclimatization, rabbits were weighed to establish a baseline, ensuring comparable mean body weights across all experimental groups.

Experimental Design and Induction of Colibacillosis

To ensure unbiased group allocation, animals were randomly assigned to one of six experimental cohorts (n = 5 per group) using a computer-generated randomization sequence (www.random.org). Throughout the study, the investigators adhered to a strict blinding protocol; all samples and histological slides were labelled with anonymous codes, which were unblinded only after the completion of the final statistical analysis. The experimental groups were designated as follows: T0 (normal control, non-infected, receiving sterile water), T1 (negative control, infected, untreated), T2 (positive control, infected, treated with Neomycin 10 mg/kg p.o. for 5 days), T3 (low dose, infected, 400 mg/kg combined extract p.o.), T4 (high dose, infected, 800 mg/kg combined extract p.o.), and T5 (immunosuppression control, non-infected, receiving 30 mg/kg cyclophosphamide i.p.).

To establish a model of acute colibacillosis, rabbits in groups T1 through T4 were pretreated with a combination of cyclophosphamide (30 mg/kg bw, intraperitoneally) and streptomycin (150 mg/kg bw, orally) 48 hours prior to *E. coli* inoculation. This dual-pretreatment protocol is essential in adult lagomorphs, as streptomycin disrupts resident microflora to overcome colonization resistance, while cyclophosphamide induces a transient state of immunosuppression, effectively mimicking the susceptibility of weanling or immunologically stressed rabbits to enteropathogenic *E. coli* colonization. Following these physiological disruptions, colibacillosis was induced in groups T1 through T4 via oral gavage of 1 mL of an *E. coli* suspension (10^9 CFU/mL in 0.9% saline). Therapeutic interventions were administered daily (10 mL/kg bw) for 7 consecutive days, commencing 24 hours post-infection. To ensure uniform experimental conditions and control for handling stress, animals in the T5 group, pretreated with cyclophosphamide (30 mg/kg bw, intraperitoneally) received a daily oral gavage of the vehicle (10 mL/kg bw of sterile distilled water) for the same 7-day duration.

Clinical and Microbiological Monitoring

Daily faecal samples were collected to quantify bacterial shedding. Stool samples (10 mg) were serially diluted (10^{-5}) in sterile saline and plated on MacConkey agar. After 24 h incubation at 37°C, typical *E. coli* colonies were counted and expressed as Log_{10} CFU/g of stool. Growth performance was monitored by weighing rabbits every two days.

Ethical Considerations and Humane Endpoints

A strict clinical monitoring protocol was implemented due to the risk of severe morbidity associated with inducing acute colibacillosis. Rabbits were monitored three times daily (every 8 hours) by for clinical signs of distress. Humane endpoints were strictly defined as: (i) loss of >20% of initial body weight; (ii) severe lethargy or inability to access food/water; (iii) persistent recumbency or lack of righting reflex; (iv) laboured or agonal breathing; and (v) hypothermia or severe dehydration (skin tent >2 seconds).

Any animal meeting these criteria was designated for immediate rescue euthanasia to minimize pain and distress. In this study, only one animal in the T1 (Negative Control) group reached humane endpoints on Day 3 post-infection due to severe dehydration and was euthanized immediately. No animals died spontaneously during the experimental period.

Anaesthesia and Methods of Sacrifice

On Day 7 post-infection (PI), a total of 24 surviving rabbits (n = 4 per group) were humanely sacrificed for sample collection. The remaining five healthy animals from the treatment groups were excluded from terminal sampling and were maintained under standard husbandry conditions for continued post-experimental monitoring. To ensure animal welfare and data integrity, rabbits were moved to a separate, quiet procedure room. Animals were fasted for 12 hours before euthanasia to reduce anaesthesia-related complications. Deep surgical anaesthesia was induced via intramuscular injection of ketamine (30 mg/kg) combined with diazepam (10 mg/kg). The depth of anaesthesia was strictly verified by the complete abolition of the pedal withdrawal and corneal reflexes before any procedures commenced. Euthanasia was completed under deep anaesthesia via cervical dislocation (for rabbits under 1 kg) or exsanguination via cardiac puncture, ensuring a rapid and painless death in accordance with AVMA Guidelines for the Euthanasia of Animals.

Sample Collection, Systemic Antioxidant and Histopathological Analysis

Blood was collected for haematology (EDTA tubes) and serum biochemistry (dry tubes). Haematological parameters included WBC (white blood cells), RBC (red blood cells), HGB (Haemoglobin), HCT (Haematocrit), and leukocyte differentials, analysed using a Mindray BC-5300 automated analyser. Serum biochemistry included ALT (Alanine aminotransferase), AST (aspartate aminotransferase), GGT (Gamma-Glutamyl Transferase), Creatinine, Uric Acid, Total Protein, and lipid profile, including TC (Total Cholesterol), TG (Triglycerides),

and HDL (High-Density Lipoprotein cholesterol), analysed using commercial kits (LABKIT, France). Cytokine levels (TNF-α, IL-1β, IL-6, IL-10, and CRP) were quantified via sandwich ELISA (Quantikine® Rabbit ELISA kits, Sigma-Aldrich).

Tissue samples from the ileum and caecum, liver, and kidney were collected post-mortem for antioxidant assays and histopathological analysis. Ileum and caecum segments were homogenized in 50 mM Tris-HCl buffer (pH 7.4) and centrifuged at 1500 g for 25 min. Supernatants were analysed for MDA (Malondialdehyde) [22], GSH (reduced glutathione) [23], SOD (Superoxide dismutase) [24] and Catalase [25].

For histopathological evaluation, liver, kidney, and caecum sections were fixed in 10% buffered formalin, embedded in paraffin wax. Sections (5 µm thick) were cut using a rotary microtome and stained with haematoxylin and eosin (H&E). Photomicrographs were captured using a digital camera attached to a light microscope. Tissue sections were subjected to a rigorous, independent qualitative descriptive analysis focusing on the presence, distribution, and resolution of classical pathological signs associated with enteric *E. coli* infection, including epithelial desquamation, villous blunting, inflammatory cell infiltration, and vascular congestion. After all sample collection, rabbit carcasses were autoclaved before disposal in compliance with biosafety regulations.

Advanced Statistical and Multivariate Analysis

The data were analysed using OriginLab Pro 2025 and GraphPad Prism version 8.0.1 (GraphPad Software, San Diego, CA, USA). All quantitative data are expressed as mean ±standard deviation (SD). Before hypothesis testing, the normality of data distribution was verified using the Shapiro–Wilk test, and homogeneity of variances was confirmed via Levene's test. Univariate comparisons across the six experimental groups were performed using one-way Analysis of Variance (ANOVA), followed by Tukey's Honest Significant Difference (HSD) post-hoc test to control for multiple comparison inflation and Type I error accumulation. Statistical significance was defined at $P < 0.05$.

To evaluate the holistic therapeutic trajectory, an integrated multivariate approach was performed in the RStudio environment (v4.4.3). Principal Component Analysis (PCA) was utilized to reduce dataset dimensionality, with variables auto-scaled (mean-centred and standardized) to account for differing units. We further generated a global biological fingerprint using hierarchical clustering, where heatmaps were constructed from Z-score-normalized data using Euclidean distance and Ward's linkage method to visualize metabolic

and inflammatory shifts across cohorts. A direct comparison between the 800 mg/kg dose and Neomycin (10 mg/kg) was conducted to assess clinical non-inferiority, defined by the absence of significant divergence ($P > 0.05$) in recovery endpoints.

The study sample size ($n = 4$ per group) was justified by an *a priori* power analysis. Based on historical indices of acute enteric inflammation (anticipating a Cohen's $f \geq 0.70$), this sample size was sufficient to achieve a statistical power ($1 - \beta$) of 0.80 at $\alpha = 0.05$, consistent with the 3Rs (Reduction) ethical framework. All multivariate analyses and visualizations were performed in the R statistical environment (v4.4.3) using the tidyverse for data manipulation, FactoMineR and factoextra for multivariate modelling, and ggplot2 (ggrepel) for high-resolution, colour-blind friendly graphical representation.

Results

Biochemical identification of *E. coli* isolates and virulence testing

The clinical isolate recovered from symptomatic rabbits was subjected to standardized biochemical characterization using the API 20E system (bioMérieux, Marcy l'Etoile, France). The isolate exhibited characteristic carbohydrate fermentation patterns, including positive reactions for glucose, sorbitol, and arabinose, while remaining negative for urease and H_2S production. The resulting biochemical profile confirmed the identity of the pathogen as *E. coli* with an identification probability of 100% (API 20E profile 5044572; identification quality: very good; probability: 100% for *E. coli* 1), validating its use for the subsequent experimental infection model (Figure 1, Suppl Table 1). The virulence potential of the *E. coli* isolate was phenotypically confirmed via the Congo red binding assay. The isolate exhibited strong dye uptake, resulting in distinct, red-pigmented colonies (CR+) after 24 hours of incubation (Suppl Figure 1). This positive phenotype indicates the presence of virulence-associated surface proteins and curli fimbriae, validating the isolate's capacity for host colonization and its suitability for inducing experimental colibacillosis.

Effects of the combination of *Psidium guava* and *Tithonia diversifolia* extracts on the body weight of experimentally infected rabbits

The effect of *E. coli* infection and subsequent treatment with the combination of *T. diversifolia* and *P. guajava* extracts on the growth trajectory of rabbits is presented in Figure 2 (Suppl Table 2). Following the experimental challenge, a significant divergence in body weight was observed between the study cohorts. The infected, untreated group (T1) exhibited a sharp

and persistent decline in body weight throughout the 7-day post-infection (PI) period. This pathological weight loss is associated with severe malabsorption of nutrient and dehydration from infectious diarrhoea.

In contrast, rabbits treated with the combined *P. guajava* and *T. diversifolia* extracts exhibited a reversal of the infection-induced weight loss. From Day 3 post-infection (PI) onward, the 800 mg/kg dose (T4) promoted body weight gain following a trajectory like that of the healthy control (T0) and Neomycin-treated (T2) groups ($P < 0.05$). However, the 400 mg/kg dose (T3) resulted in moderate weight recovery, indicating a clear dose-dependent therapeutic response. Notably, the 800 mg/kg dose demonstrated efficacy in restoring rabbits to their pre-infection growth curves. These results suggest that the combined extract could be facilitating nutrient assimilation and helping restore osmotic and fluid-solute equilibrium.

Effect of the combination of *Psidium guava* and *Tithonia diversifolia* extracts on the relative weight of some vital organs

The macroscopic effect of *E. coli* infection and the safety profile of the combined *P. guajava* and *T. diversifolia* extracts treatment were evaluated through relative organ weight analysis and visual inspection (Figure 3). Upon necropsy, all treatment groups exhibited normal organ coloration and morphology, with no visible lesions or congestion in the liver, kidneys, or heart. Statistical analysis revealed that the relative weights of the kidneys and liver in both the 400 and 800 mg/kg extract groups showed no significant variation ($P > 0.05$) when compared to the healthy control (T0) and the Neomycin-treated (T2) groups.

Notably, the 800 mg/kg cohort (T4) maintained liver and spleen weights comparable to healthy controls, contrasting with the inflammatory oedema and reactive splenomegaly typically observed in untreated colibacillosis. In the infected-untreated group (T1), increased relative spleen weight suggested a heightened systemic immune response to the bacterial load. Conversely, treatment with the *P. guajava* and *T. diversifolia* combination effectively preserved organ homeostasis. These results suggest that the extract could be providing systemic protection against *E. coli*-induced pathology while maintaining the structural integrity of vital metabolic organs, without inducing adverse toxic effects.

Therapeutic Efficacy and *in vivo* bacterial clearance of the combination of *P. guajava* and *T diversifolia*

The *in vivo* antibacterial potency of the *P. guajava* and *T. diversifolia* combination was assessed by monitoring the temporal kinetic of *E. coli* shedding in the faeces (Figure 4, Suppl Table 3). Bacterial load is a critical indicator of the extract's ability to disrupt intestinal colonization and prevent further mucosal invasion. Following the experimental challenge, all infected groups exhibited peak bacterial shedding (10^9 CFU/10 mg stool) before the initiation of therapy.

Treatment led to a progressive, time-dependent reduction in bacterial shedding across all cohorts. While the reference antibiotic (Neomycin, T2) achieved complete bacterial clearance within 72 hours (Day 3), the combined *P. guajava* and *T. diversifolia* extracts also demonstrated potent curative efficacy. Specifically, both the 400 mg/kg (T3) and 800 mg/kg (T4) doses resulted in no quantifiable bacterial load in faeces by Day 5 of treatment. This 5-day clearance window is clinically significant, as it correlates with the rapid resolution of diarrheic symptoms and the reversal of infection-induced weight loss observed (Figure 3). Collectively, these results indicate that the combined extract exhibits effective, dose-dependent curative activity against *E. coli*, achieving clinical recovery comparable to the reference antibiotic. These findings demonstrate that the *P. guajava* and *T. diversifolia* formulation particularly the 800 mg/kg dose could be a promising biological alternative for the management of clinical colibacillosis.

The effect of combined aqueous extract treatment on the Antioxidant Defense

The impact of *E. coli* infection and subsequent treatment with combined *P. guajava* and *T. diversifolia* extracts on the intestinal redox landscape was evaluated (Figure 5; Suppl Table 4). In the infected-untreated cohort (T1), experimental colibacillosis induced a state of biochemical collapse within both the caecum and ileum. This pathological state was characterized by a significant increase ($P < 0.05$) in malondialdehyde (MDA) levels linked to extensive lipid peroxidation, suggesting the depletion of the primary antioxidant defences, including Superoxide Dismutase (SOD), Catalase (CAT), and reduced Glutathione (GSH). These findings demonstrated that *E. coli* colonization rapidly exhausts the host's local antioxidant protection, leading to oxidative tissue damage.

Therapeutic administration of the *P. guajava* and *T. diversifolia* extracts effectively reversed this oxidative trend in a dose-dependent manner. While the 400 mg/kg dose (T3) provided moderate protection, the 800 mg/kg dose (T4) achieved a superior restorative profile,

returning MDA, CAT, and SOD levels to values statistically similar from those of the healthy control (T0).

Interestingly, in specific parameters such as caecal GSH and ileal SOD, the 800 mg/kg dose of combined *P. guajava* and *T. diversifolia* extracts treatment maintained the antioxidant levels that were numerically superior to the Neomycin standard (T2). This could indicate that while Neomycin serves as a potent bactericidal agent, the combined *P. guajava* and *T.diversifolia* extracts could be providing a dual-action benefit by simultaneously preventing the *E. coli* colonization but also restoring the intestinal antioxidant framework.

The effect of combined aqueous extract treatment on haematological stabilization and resolution of systemic inflammation

The systemic impact of the *E. coli* challenge and the subsequent restorative effects of the *P. guajava* and *T. diversifolia* combination are summarized in the haematological profiles (Figure 6; Suppl Table 5). The infected-untreated group (T1) exhibited a classic septic hemogram, characterized by profound leucocytosis and thrombocytosis. Specifically, White Blood Cell (WBC) counts nearly doubled and Platelet (PLT) counts nearly tripled compared to the healthy control (T0), signalling a severe systemic inflammatory response and the activation of the host's emergency myeloid defences against *E. coli* septicaemia.

Simultaneously, the T1 cohort suffered from anaemia of infection, evidenced by significant declines ($P < 0.05$) in Red Blood Cell (RBC) counts, Haemoglobin (HGB) levels, and Haematocrit (HCT) compared to all other groups. This erythrocytic collapse likely reflects the combined effects of bacterial hemolysins, iron sequestration, and the haemorrhagic nature of the colibacillosis-induced intestinal lesions.

Treatment with the combined *P. guajava* and *T. diversifolia* extracts achieved near-complete normalization of the hemogram in a dose-dependent manner. The 800 mg/kg dose (T4) proved to be particularly effective, successfully restoring the systemic inflammatory response by returning WBC and PLT counts to baseline levels. Furthermore, the bioactive extract reversed the erythrocytic decline, restoring haemoglobin concentrations from a pathological low of approximately 11 g/dL to a healthy 15 g/dL. Notably, the leukocyte differential (GRA%, LYM%, and MID %) was rebalanced in the T4 group, indicating a restored and functional immune landscape comparable to the neomycin-treated (T2) and healthy (T0) cohorts. These findings underscore the extract's potent haematoprotective efficacy and its ability to halt the progression of the infection from a local gut disturbance to a systemic crisis.

Immunomodulatory effects of combined aqueous extract treatment on *E. coli*-infected rabbits

The immunological response to *E. coli* infection and the restorative capacity of the combined *P. guajava* and *T. diversifolia* extracts intervention were evaluated through key pro-inflammatory and anti-inflammatory biomarkers (Figure 7; Suppl Table 6). In the infected-untreated cohort (T1), experimental colibacillosis triggered a massive cytokine production, evidenced by significant elevations ($P < 0.05$) in C-reactive protein (CRP), TNF-α, IL-1β, and IL-6. This excessive release of innate first-responder cytokines drives the systemic inflammatory response, leading to tissue damage and metabolic exhaustion observed in the T1 group.

Oral administration of the *P. guajava* and *T. diversifolia* combination at 400 and 800 mg/kg induced a significant reduction in these pro-inflammatory markers. Notably, for critical indicators like TNF-α and IL-6, the 800 mg/kg dose (T4) was more potent, suppressing cytokine levels as effectively as the Neomycin standard (T2). This suggests that the bioactive metabolites in the extract, such as flavonoids and sesquiterpene lactones, likely modulate the underlying signalling pathways (such as NF-kB) responsible for acute inflammatory cascades.

Conversely, the anti-inflammatory cytokine IL-10 showed a distinct and clinically relevant pattern. While IL-10 levels were significantly lower in the untreated group (T1), all treated cohorts (T2, T3, T4) maintained significantly higher concentrations of this cytokine ($P < 0.05$). This could be an indication that the combined *P. guajava* and *T. diversifolia* extract does not merely suppress the immune system; rather, it facilitates immunological rebalancing by promoting an anti-inflammatory environment that accelerates tissue healing and the resolution of diarrhoea. The normalization of CRP, a standard marker of acute systemic inflammation in rabbits, further validates that the T4 dose achieved complete systemic resolution of the infection**.**

Effects of combined aqueous extract treatment on Hepato-Renal Protection and Metabolic Homeostasis in *E. coli*-infected rabbits

The systemic metabolic profile and organ-functional integrity of the experimental rabbits are summarized in Figure 8 (Suppl Table 7). Induction of colibacillosis in the untreated cohort (T1) resulted in significant biochemical derangement ($P < 0.05$), characterized by a dyslipidaemia of infection and acute hepato-renal distress. Specifically, T1 rabbits exhibited an increase in

serum transaminases (ASAT, ALAT) and GGT, signalling acute hepatocellular injury and membrane leakage induced by bacterial endotoxins.

Renal function was similarly compromised in the untreated group, with Creatinine and Urate levels nearly doubling. This elevation may indicate a reduced Glomerular Filtration Rate (GFR), likely secondary to infection-induced dehydration and septic renal injury. Furthermore, a state of hypoproteinaemia and hypoalbuminemia was observed in T1, reflecting the systemic inflammatory response and potential protein-losing enteropathy associated with severe diarrhoea.

Therapeutic intervention with the *P. guajava* and *T. diversifolia* extracts effectively shielded the primary metabolic organs from this endotoxicity. The 800 mg/kg dose (T4) achieved a metabolic reset, returning ASAT, GGT, Creatinine, and Urate to baseline levels that were statistically indistinguishable from those of the healthy controls (T0). Notably, the extract restored the lipid profile by lowering triglycerides and LDL-cholesterol while significantly increasing HDL-cholesterol ($P < 0.05$) compared to the untreated group. By stabilizing hepatocellular membranes and maintaining renal blood flow, the 800 mg/kg dose showed organ-protective effects. This helped restore systemic balance comparable to the neomycin-treated group (T2).

Integrated Hierarchical Clustering: The Global Biological Fingerprint

Hierarchical clustering analysis was performed on the integrated biological dataset to visualizes the holistic therapeutic trajectory of the rabbits (Figure 9). The resulting heatmap successfully discriminated between the various experimental cohorts, establishing a clear molecular signature for both the pathological state and the subsequent recovery. The infected-untreated cohort (T1) formed a distinct cluster characterized by a massive upregulation of pro-inflammatory and damage-associated markers, specifically manifesting as elevated ASAT, creatinine, MDA, and pro-inflammatory cytokines (TNF-α, IL-6).

In stark contrast, the cohort treated with 800 mg/kg of combined *P.guajava* and *T. diversifolia* extracts (T4) exhibited a reversal of this fingerprint. The T4 group clustered closely with the healthy control (T0) and the neomycin-treated (T2) groups, displaying a synchronized restoration of antioxidant enzymes (SOD, CAT, GSH) and a normalization of systemic biochemical indices. This global transition from the oxidative-inflammatory cluster to the homeostatic cluster underscores the multifaceted curative potential of the *P. guajava* and *T. diversifolia* combination. These results could be suggesting that the bioactive extract does not

merely target isolated symptoms but induces a systemic homeostatic restoration, restoring the animal's physiological state to its pre-infection baseline.

Multivariate Trajectory and Key Biological Drivers

Principal Component Analysis (PCA) was applied to the combined dataset as an exploratory tool to visualize the overall multidimensional relationships and clustering trends across the experimental cohorts (Figure 10A). The unsupervised PCA model revealed a visible separation among groups, with the first two dimensions capturing a cumulative total of 57.7% of the total variance. Principal Component 1 (Dim1), which accounted for 48.0% of the variance, served as the primary axis of separation, segregating the coordinates of the infected-untreated cohort (T1) from the clustering space of the healthy controls (T0). Notably, the 800 mg/kg cohort (T4) exhibited a spatial shift along Dim1, positioning itself in closer proximity to the multivariate space occupied by the T0 and Neomycin (T2) groups. This spatial alignment suggests an exploratory trend wherein the global physiological profile of the high-dose extract cohort reflects an orientation toward the healthy and reference treatment spaces. However, given the exploratory nature of this analysis and the lack of an independent validation dataset, these spatial configurations represent descriptive clustering behaviours rather than definitive proof of systemic homeostatic equivalence.

The Variable Contribution analysis (Figure 10B) further highlighted the primary variables associated with the spatial distribution across the dimensions. The clustering trends observed in the PCA map were heavily influenced by coordinates related to bacterial clearance kinetics (load at Days 10, 12, and 13) and systemic inflammatory markers (WBC and CRP). While these variables exhibited the highest contribution scores along Dim1, these mathematical relationships remain exploratory in nature. In small-cohort designs, individual biological variation can heavily influence variable contribution weights. Thus, these parameters should be viewed as hypothesis-generating indicators, pointing to pathogen eradication and the subsequent quenching of the systemic leukocyte response as key therapeutic pathways of the extract rather than validated predictive biomarkers.

Comparative Functional Efficacy and Therapeutic Parity

A comparative functional analysis was conducted between the high-dose extract (800 mg/kg) and the Neomycin standard to evaluate the clinical viability of the combination of the *T. diversifolia and P. guajava* intervention as a sustainable alternative to conventional therapy (Figure 11).

The restorative capacity of the *P. guajava* and *T. diversifolia* combination was found to be functionally equivalent to Neomycin across all oxidative stress indices. In both the caecum and ileum, the 800 mg/kg cohort exhibited a robust recovery of Superoxide Dismutase (SOD) and Catalase (CAT) activities, with mean values statistically indistinguishable from the antibiotic-treated group ($P > 0.05$).

In terms of immunomodulation, the extract achieved complete systemic stabilization. The suppression of C-reactive protein (CRP), a sensitive marker of acute-phase inflammation, in rabbits, and the normalization of White Blood Cell (WBC) counts showed no significant divergence between the combined extract of *P. guajava and T. diversifolia* and Neomycin cohorts. This suggests that the extract possesses a systemic anti-inflammatory mechanism comparable to the resolution achieved through bacterial clearance by synthetic antimicrobials.

By resolving the *E. coli* challenge through both direct antimicrobial action and active tissue repair, the 800 mg/kg dose demonstrated therapeutic efficacy comparable to that of Neomycin. These findings suggest that this combined *P. guajava* and *T. diversifolia* extracts could be a potent bioactive agent, an alternative to conventional antibiotic that can maintain rabbit health and productivity.

Histopathology results

Effect of combined extract on the renal architecture

The protective effect of the *P. guajava* and *T. diversifolia* combination on the renal microarchitecture of *E. coli*-infected rabbits is presented in Figure 12. Microscopic examination of the kidney sections from the healthy control group (T0) revealed a well-preserved renal parenchyma. This was characterized by a distinct glomerular architecture (G) with a clear Bowman's space (US) and clearly differentiated proximal (PT) and distal (DT) convoluted tubules, indicating normal physiological filtration and reabsorption functions.

In contrast, the infected-untreated cohort (T1) exhibited significant histopathological derangement. The infection induced a localized inflammatory response marked by conspicuous leukocyte infiltration (IL) and mesangial expansion within the glomerulus. These alterations are indicative of acute renal distress and the systemic inflammatory burden caused by the pathogenic *E. coli* strain. However, therapeutic intervention with the combined *P. guajava* and *T. diversifolia* extracts effectively mitigated these structural damages. The renal

sections from the T3 (400 mg/kg) and T4 (800 mg/kg) groups, alongside the Neomycin-treated cohort (T2), demonstrated a restoration of the renal tissue architecture. While the 800 mg/kg dose (T4) showed minor residual leukocyte infiltrates, the overall prevention of mesangial expansion and tubular disruption confirms the extract's nephroprotective potential. This structural preservation is consistent with the biochemical results, where the extract successfully normalized serum creatinine and uric acid levels, confirming that the formulation prevents the acute kidney injury (AKI) typically associated with severe colibacillosis.

Protective Effect of Extracts on Hepatic Microarchitecture

The histological evaluation of the liver (Figure 13) confirms the organoprotective properties of the combination of *P. guajava* and *T. diversifolia* during acute *E. coli* infection. Microscopic analysis of the liver from the healthy control group (T0) showed a classical hexagonal lobular arrangement with a well-preserved hepatic parenchyma. The sections exhibited a distinct portal triad comprising the hepatic portal vein (PV), hepatic artery (HA), and bile canaliculus (BC) surrounded by radiating cords of well-defined, polyhedral hepatocytes (He) with prominent nuclei.

In the infected-untreated cohort (T1), the hepatic architecture was compromised by a localized inflammatory response, characterized by significant leukocyte infiltration (IL). This infiltration suggests that the systemic *E. coli* infection induced a degree of hepatitis or endotoxin-mediated tissue stress. However, administration of the combined *P. guajava* and *T. diversifolia* extracts formulation at both 400 mg/kg (T3) and 800 mg/kg (T4) successfully mitigated these inflammatory changes. The hepatic sections of the treated groups showed a restoration of the hepatocellular cords and a marked absence of inflammatory clusters, appearing histologically comparable to the Neomycin-treated (T2) and healthy control (T0) groups.

This structural preservation directly correlates with the enzymatic profiles discussed earlier; by preventing the infiltration of inflammatory cells and maintaining the integrity of the hepatocellular membranes, the extract effectively prevented the leakage of transaminases (ASAT and ALAT) into the systemic circulation. These findings reinforce the dual role of the combination of *P. guajava* and *T. diversifolia* as both an antimicrobial and a potent hepatoprotective agent.

Restorative Effects of extracts on Intestinal (Caecal) Microarchitecture

The impact of the *E. coli* challenge and the subsequent therapeutic intervention on the caecal microarchitecture is presented in Figure 14. Histological analysis of the caecal sections from healthy control rabbits (T0) revealed a robust and well-organized intestinal wall. The architecture followed a classic structural hierarchy, consisting of the serosa (Se), muscular layer (Mus), submucosa (Smu), and a well-developed mucosa (Mu) lining the intestinal lumen (Lu).

In the infected-untreated cohort (T1), the *E. coli* challenge induced severe mucosal degeneration. The sections exhibited a distinct narrowing of the intestinal mucosa and marked villous atrophy. This loss of structural height and complexity in the mucosal layer indicates a depletion of goblet cell function (leading to decreased mucus secretion) and a reduction in the total absorptive surface area. Such morphological damage provides a structural explanation for the persistent diarrhoea and negative growth performance observed in structurally comparable to the healthy controls and the Neomycin-treated cohort (T2). By maintaining the mucosal barrier and preventing villous blunting, the high-dose extract (800 mg/kg) preserved the intestinal function, facilitating nutrient absorption and preventing the translocation of enteric pathogens. This structural recovery serves as the biological foundation for the observed resolution of clinical diarrhoea and the restoration of the animals' growth trajectory.

Discussion

Even though rabbit meat continues to be considered a niche product, demand for it is increasing steadily in various parts of the world, particularly in Asia [26]. Beyond this expanding market, rabbit farming also offers advantages in social equity [27] and environmental sustainability [2]. Despite this potential, the industry, especially in developing nations, is severely constrained by enteric pathologies, most notably colibacillosis caused by pathogenic strains of *E. coli*. This disease compromises animal health and productivity through high morbidity and mortality, and poses a critical One Health risk because the transmission of multidrug-resistant (MDR) strains through the food chain represents a growing public health threat [28,29]. As the efficacy of conventional antimicrobials wanes with the global rise of antimicrobial resistance (AMR), the exploration of plant-based therapeutic alternatives has become a pharmacological necessity. Our previous ethnopharmacological screening of Cameroonian flora identified *P. guajava* and *T. diversifolia* as potent inhibitors of pathogenic *E. coli*, with their combination exhibiting a synergistic reduction in Minimum Inhibitory Concentration of up to 64-fold [19]. Building on that in vitro evidence, the present study evaluated the *in vivo* curative potential and systemic

restorative effects of the combined *P. guajava* and *T. diversifolia* extract in *E. coli*-infected rabbits.

The therapeutic efficacy of the combined extract was assessed in colibacillosis-infected rabbits, following the induction protocol of Pouofo et al.[30]. The treatment was conducted for 7 days, during which the bacterial shedding was used to monitor the treatment. By the end of the treatment period, the various parameters evaluated to determine the extract's therapeutic efficacy, including body weight, relative organ weight, bacterial shedding, haematological, biochemical, inflammatory, and oxidative markers, as well as histopathological findings, showed comparable values between T5 (immunosuppression group) and the healthy control group T0 (uninfected, untreated, and free of cyclophosphamide/streptomycin). This finding indicates that the immunosuppressive effect of cyclophosphamide was reversible and had no measurable lasting impact under our experimental conditions. Therefore, the variation observed in the infected groups could be attributed to the infection and/or extract treatment rather than to pretreatment toxicity.

The experimental induction of colibacillosis produced a profound clinical decline in the untreated cohort, characterized by acute weight loss, dehydration, and high-titer fecal shedding of Congo red–positive *E. coli*. This pathological state is consistent with the capacity of pathogenic *E. coli* to disrupt intestinal water transport and induce catabolic stress [31]. The reversal of these symptoms following administration of the combined extract, particularly at 800 mg/kg demonstrates clear therapeutic efficacy. Bacterial clearance by Day 5 of treatment by combined extract is notable and comparable to the fast-killing activity observed with Neomycin. This bacterial clearance is consistent with previously observed antimicrobial activity of these extracts and their combination [19,32].

Our LC-MS/MS profiling of the two extracts identified pedunculagin, an ellagitannin, in *P. guajava*, together with the flavonoid’s quercetin, quercetin-4′-O-glucoside, myricetin-3-O-pentoside, and 2′-hydroxy-α-naphthoflavone in *T. diversifolia* (Suppl. Table 8). In fact, tannins and flavonoids of *P. guajava* have been reported to interact with bacterial cell-wall proteins and inhibit *E. coli* growth. Dhiman et al. [33], showed that the methanolic extract *P. guajava* leaf exhibited antibacterial activity against *E. coli* with a minimum inhibitory concentration of 0.78 μg/ml and minimum bactericidal concentration of 50 μg/ml and the phytochemical screening revealed flavonoids, steroids, and tannins as the likely antimicrobial contributors. However, Pereira et al. [34] also found that an *aqueous P. guajava* leaf extract, contain flavonoid and tannin classes (rutin, hesperidin, quercetin). Recently, El-Deeb et al.

[32] conducted a comprehensive assessment of *P. cattleianum* aerial-part polyphenols and found that, beyond producing significant antidiarrheal effects in a castor oil-induced diarrhoea model, the extract also showed direct activity against several diarrhea-associated pathogens, with *in silico* docking implicating the bacterial fatty-acid synthesis enzyme FabH and the kappa opioid receptor as plausible molecular targets. For *T. diversifolia*, an independent chemical characterization of an *aqueous* leaf extract by Dongmo et al. [35] corroborates the presence of tannins and flavonoids alongside phenols, alkaloids, terpenoids, and saponins, consistent with our own LC-MS/MS annotation.

The septic hemogram observed in the untreated group, marked by leucocytosis, thrombocytosis, and secondary anaemia, reflects the systemic inflammatory burden of colibacillosis, consistent with findings reported by Petrov et al. [36]in both spontaneous and experimental colibacillosis in weaned rabbits. The normalization of these parameters in all treated groups (T2–T4) indicates resolution of systemic stress. The extract lowered circulating TNF-α, IL-6, and IL-1β and returned CRP to near-normal levels, consistent with an immunomodulatory rather than a purely immunosuppressive action. Phytochemicals from both plant sources may plausibly contribute to this effect. For *P. guajava*, the strongest supporting evidence comes from a recent study in carrageenan-induced inflammation in Wistar rats, which found that ethanolic *P. guajava* bark extract significantly reduced TNF-α, IL-6, and CRP relative to untreated controls, with molecular docking indicating binding of extract constituents to inflammatory target proteins [37]. For *T. diversifolia*, Broering et al. [38] reported that a *T. diversifolia* extract reduced secretion of TNF, IL-1β, IL-6, and nitrite, and inhibited neutrophil chemotaxis and CD18 expression in carrageenan-induced inflammation models. However, specific mechanisms such as NF-κB inhibition, direct cytokine receptor modulation, ROS scavenging, membrane stabilization, or inhibition of bacterial adhesion were not directly evaluated in the present study, therefore remain as plausible literature-supported hypotheses rather than demonstrated mechanisms. Unlike antibiotics, which primarily target the pathogen, this combined extract of *P. guajava* and *T. diversifolia* actively manages the host's immune response, preventing the tissue-damaging effects of chronic inflammation.

The hypoproteinaemia and hypoalbuminemia observed in Group T1 are consistent with a negative acute-phase response, in which the liver shifts protein synthesis toward inflammatory mediators such as CRP at the expense of albumin [39], a pattern previously reported in *E. coli* infection [40,41]. The concurrent rise in ASAT and GGT points to hepatocellular leakage,

while elevated uric acid suggests impaired renal clearance or accelerated purine catabolism secondary to tissue damage. The extract's ability to restore these metabolic markers, corroborated by our histological findings of preserved portal triad architecture and tubular integrity, is consistent with a hepatoprotective and nephroprotective effect [42].

At the primary site of infection, colibacillosis-induced oxidative stress (elevated MDA, depleted SOD/CAT) plausibly drove the mucosal damage observed histologically, with villous atrophy and mucosal narrowing providing a physical basis for the malabsorption seen in untreated animals. The tannins and flavonoids identified in both extracts are consistent with a protective, astringent mechanism in which these compounds could coat and stabilize the mucosal surface, limiting further epithelial damage. That could plausibly contribute to the restored villous height and mucosal thickness observed in treated groups [43]This mechanism was not directly tested in the present study and remains a hypothesis consistent with the compositional and histological findings.

The multivariate analyses (PCA and hierarchical clustering) show that, within this exploratory dataset, the 800 mg/kg dose cohort clusters closer to the healthy and Neomycin-treated groups than to the untreated group. Given the small per-group sample size ($n = 4$) relative to the number of variables analysed, and the absence of an independent validation cohort, these multivariate results should be interpreted as purely descriptive. Overall, the combined extract shows antimicrobial, immunomodulatory, antioxidant, and organ-protective effects comparable to Neomycin at 800 mg/kg, supporting further mechanistic and pharmacokinetic investigation as a candidate alternative for colibacillosis management.

Limitations of the Study

The chemically induced infection model (cyclophosphamide + streptomycin) is a manipulated, opportunistic model rather than a natural infection; the T5 control indicates pretreatment effects had resolved by the study endpoint, but early interactions between immunosuppression and infection remain a confounding variable that non-chemical, age-susceptible models could better address. Second, the small sample size ($n = 4$–5/group), while adequately powered for the large primary effects observed (a priori Cohen's $f \geq 0.70$, power = 0.80), limits detection of subtler effects and may not capture the full range of biological variability in outbred rabbits. Third, histopathological evaluation was qualitative, without semi-quantitative lesion scoring or morphometric measurement (villus height, crypt depth), due to equipment constraints; the polarized group differences were visually clear but not

numerically graded. Fourth, PCA/heatmap analyses are exploratory given the sample-to-variable ratio and lack an independent validation cohort. Finally, mechanistic claims regarding NF-κB inhibition, receptor binding, membrane disruption, or adhesion inhibition are hypotheses drawn from phenotypic and phytochemical data, not directly tested pathways; future work (bioactivity-guided fractionation, adhesion assays, pharmacokinetics, dedicated toxicology, and non-chemical infection models) is needed to confirm them.

Conclusion

The present study demonstrates the *in vivo* efficacy of the combined *P. guajava* and *T. diversifolia* extracts in mitigating *E. coli* infection in a rabbit model. In infected, immunocompromised rabbits, the combined extract at 800 mg/kg was associated with resolution of the clinical signs of colibacillosis, including restored body-weight gain and a reduction in faecal bacterial shedding to near-undetectable levels, alongside repair of infection-induced tissue damage and no overt signs of toxicity within the parameters assessed in this study. Treated animals showed biochemical, haematological, and antioxidant profiles approaching those of healthy controls, though consistent with the exploratory nature of our multivariate analysis this should be read as a marked improvement toward baseline rather than confirmed physiological equivalence. These findings indicate that the combined *P. guajava*–*T. diversifolia* extract is a promising candidate for managing colibacillosis in rabbits, warranting further development. By addressing both the pathogen and the host's systemic response, this combination represents a potentially sustainable approach to colibacillosis control amid rising antimicrobial resistance, consistent with One Health-aligned veterinary practice.

Before translational application, several steps remain. Phytochemical fingerprinting and standardization of the formulation are needed to support pharmacological interpretation and reproducibility. Evaluation against a broader panel of multidrug-resistant strains would clarify the extract's relevance to other enteric infections, and 16S rRNA metagenomic profiling would clarify its impact on commensal intestinal microbiota relative to conventional aminoglycosides. Critically, dedicated pharmacokinetic, dose-optimization, and long-term toxicology studies, together with larger confirmatory trials, are required before the clinical or field-level viability of this formulation can be established.

Supporting information

Data is provided within the supplementary information files.

Author contributions

Data curation, Formal analysis, Investigation, Methodology, Validation, Writing – original draft: Mireille Tuedom Chouegouong
Conceptualization, Data curation, Software, Formal analysis, Project administration, Resources, Supervision, Validation, Visualization, Writing – review & editing: Rufin Marie Kouipou Toghueo
Investigation, Methodology and Data analysis: Yanick Kevin Melogmo Dongmo[1],
Investigation, Methodology and Data analysis: Jaurès Marius Tsakem Nangap,
Investigation, Methodology and Data analysis: Michele Stella Majoumouo
Investigation, Methodology and Data analysis: Brice Pinlap
Conceptualization, Resources, Supervision: Fabrice Fekam Boyom and Kingsley Agbor Etchu
All the authors have read and approved the final manuscript.

Competing interests

The authors declare no competing interests.

References

1. Bordignon F, Xiccato G, Trocino A, Zuffellato A, Castellini C, Mattioli S, et al. Environmental impact of rabbit production systems: a farm-based cradle-to-gate analysis. animal. 2025;19: 101488. doi:10.1016/j.animal.2025.101488

2. Cesari V, Zucali M, Bava L, Gislon G, Tamburini A, Toschi I. Environmental impact of rabbit meat: The effect of production efficiency. Meat Science. 2018;145: 447–454. doi:10.1016/j.meatsci.2018.07.011

3. European Commission. Directorate General for Health and Food Safety. Commercial rabbit farming in the European Union: overview report. LU: Publications Office; 2017. doi:10.2772/62174

4. Mancini S, Paci G. Probiotics in Rabbit Farming: Growth Performance, Health Status, and Meat Quality. Animals. 2021;11: 3388. doi:10.3390/ani11123388

5. Siddiqui SA, Gerini F, Ikram A, Saeed F, Feng X, Chen Y. Rabbit Meat—Production, Consumption and Consumers' Attitudes and Behavior. Sustainability. 2023;15: 2008. doi:10.3390/su15032008

6. Solans L, Arnal JL, Sanz C, Benito A, Chacón G, Alzuguren O, et al. Rabbit Enteropathies on Commercial Farms in the Iberian Peninsula: Etiological Agents Identified in 2018–2019. Animals. 2019;9: 1142. doi:10.3390/ani9121142

7. Martignon M, Burel C, Licois D, Reperant E, Postollec G, Valat C, et al. Challenging the growing rabbit with a moderately pathogenic E. coli under ad libitum or limited feed intake conditions: impact on digestive physiology, bacterial communities, and on post-weaning growth. World Rabbit Sci. 2021;29: 1. doi:10.4995/wrs.2021.14089

8. Long TM, Nisa S, Donnenberg MS, Hassel BA. Enteropathogenic Escherichia coli Inhibits Type I Interferon- and RNase L-Mediated Host Defense To Disrupt Intestinal Epithelial Cell Barrier Function. Bäumler AJ, editor. Infect Immun. 2014;82: 2802–2814. doi:10.1128/IAI.00105-14

9. Farooq MZ, Wang X, Yan X. Effects of Aeriscardovia aeriphila on growth performance, antioxidant functions, immune responses, and gut microbiota in broiler chickens. J Zhejiang Univ Sci B. 2023;24: 1014–1026. doi:10.1631/jzus.B2200621

10. Kerek Á, Szabó Á, Barnácz F, Csirmaz B, Kovács L, Jerzsele Á. Antimicrobial Resistance in Commensal Bacteria from Large-Scale Chicken Flocks in the Dél-Alföld Region of Hungary. Veterinary Sciences. 2025;12: 691. doi:10.3390/vetsci12080691

11. Silva A, Silva V, Tavares T, López M, Rojo-Bezares B, Pereira JE, et al. Rabbits as a Reservoir of Multidrug-Resistant Escherichia coli: Clonal Lineages and Public Health Impact. Antibiotics. 2024;13: 376. doi:10.3390/antibiotics13040376

12. Sun C, Wang Z, Li Y, Huang J. Antibiotic resistance spectrums of Escherichia coli and Enterococcus spp. strains against commonly used antimicrobials from commercial meat-rabbit farms in Chengdu City, Southwest China. Front Vet Sci. 2024;11: 1369655. doi:10.3389/fvets.2024.1369655

13. Elbehiry A, Marzouk E. From Farm to Fork: Antimicrobial-Resistant Bacterial Pathogens in Livestock Production and the Food Chain. Veterinary Sciences. 2025;12: 862. doi:10.3390/vetsci12090862

14. Pandey S, Doo H, Keum GB, Kim ES, Kwak J, Ryu S, et al. Antibiotic resistance in livestock, environment and humans: One Health perspective. J Anim Sci Technol. 2024;66: 266–278. doi:10.5187/jast.2023.e129

15. Wickramasuriya SS, Ault J, Ritchie S, Gay CG, Lillehoj HS. Alternatives to antibiotic growth promoters for poultry: a bibliometric analysis of the research journals. Poultry Science. 2024;103: 103987. doi:10.1016/j.psj.2024.103987

16. Zheng S, Li Y, Chen C, Wang N, Yang F. Solutions to the Dilemma of Antibiotics Use in Livestock and Poultry Farming: Regulation Policy and Alternatives. Toxics. 2025;13: 348. doi:10.3390/toxics13050348

17. Liu J, Han X, Zhang T, Tian K, Li Z, Luo F. Reactive oxygen species (ROS) scavenging biomaterials for anti-inflammatory diseases: from mechanism to therapy. J Hematol Oncol. 2023;16: 116. doi:10.1186/s13045-023-01512-7

18. Abe AE, De Oliveira CE, Dalboni TM, Chagas-Paula DA, Rocha BA, De Oliveira RB, et al. Anti-inflammatory sesquiterpene lactones from Tithonia diversifolia trigger different effects on human neutrophils. Revista Brasileira de Farmacognosia. 2015;25: 111–116. doi:10.1016/j.bjp.2015.01.005

19. Chouegouong MT, Majoumouo MS, Menkem EZ, Yimgang LV, Toghueo RMK, Etchu KA, et al. Ethnopharmacological survey and antibacterial activity of medicinal plant extracts used against bacterial enteritis in rabbits. ADV TRADIT MED (ADTM). 2023;23: 213–223. doi:10.1007/s13596-021-00615-1

20. Yadav V. Congo red binding and Plasmid Profile of E. coli Isolates of Poultry Origin. J Anim Health Prod. 2014;2: 31–32. doi:10.14737/journal.jahp/2014/2.3.31.32

21. Yousif AA. Effects of E.coli O157:H7 Experimental Infections on Rabbits: Donia Hatem Fadhel al-Taii and Afaf Abdulrahman Yousif. Iraqi J Vet Med. 2019;43: 34–42. doi:10.30539/iraqijvm.v43i1.468

22. Wilbur KM, Bernheim F, Shapiro OW. Determination of lipid peroxidation. Archives of Biochemistry and Biophysics. 1949;24: 305–310.

23. Ellman GL. Tissue sulfhydryl groups. Archives of biochemistry and biophysics. 1959;82: 70–77.

24. Misra HP, Fridovich I. The role of superoxide anion in the autoxidation of epinephrine and a simple assay for superoxide dismutase. Journal of Biological chemistry. 1972;247: 3170–3175.

25. Sinha AK. Colorimetric assay of catalase. Analytical biochemistry. 1972;47: 389–394.

26. European Commission. Directorate General for Health and Food Safety. Commercial rabbit farming in the European Union: overview report. LU: Publications Office; 2017. Available: https://data.europa.eu/doi/10.2772/62174

27. Leroy F, Petracci M. Rabbit meat: valuable nutrition or too-cute-to-eat? World Rabbit Sci. 2021;29: 239–246. doi:10.4995/wrs.2021.12663

28. Mahmoudi P, Rashidi A, Idrees Hama Faraj S. Network meta-analysis of the prevalence and antimicrobial resistance of Escherichia coli isolated from bovine milk and dairy products: A global perspective. Journal of Dairy Science. 2025;108: 9330–9344. doi:10.3168/jds.2024-26197

29. Risalvato J, Sewid AH, Eda S, Gerhold RW, Wu JJ. Strategic Detection of Escherichia coli in the Poultry Industry: Food Safety Challenges, One Health Approaches, and Advances in Biosensor Technologies. Biosensors. 2025;15: 419. doi:10.3390/bios15070419

30. Pouofo Nguiam M, Njonte Wouamba SC, Longo F, Gounoue Kamkumo R, Donji Kenne Foweda L, Dzeufiet Djomeni PD, et al. Antibacterial and antishigellosis activity of Xylopia staudtii (engl. & diels), Annonaceae. Journal of Ethnopharmacology. 2021;280: 114406. doi:10.1016/j.jep.2021.114406

31. Swennes AG, Buckley EM, Madden CM, Byrd CP, Donocoff RS, Rodriguez L, et al. Enteropathogenic Escherichia coli prevalence in laboratory rabbits. Vet Microbiol. 2013;163. doi:10.1016/j.vetmic.2013.01.006

32. El-Deeb EM, Elsayed HE, Khazaal MT, El-Sayed EK, Ahmed AA, Mansour YE, et al. Comprehensive assessment of the antidiarrheal properties of polyphenols from Psidium cattleianum Sabine's aerial parts. Journal of Ethnopharmacology. 2025;346: 119670.

33. Dhiman A, Nanda A, Ahmad S, Narasimhan B. In vitro antimicrobial activity of methanolic leaf extract of Psidium guajava L. Journal of Pharmacy and Bioallied Sciences. 2011;3: 226–229. doi:10.4103/0975-7406.80776

34. Pereira GA, Chaves DSDA, Silva TME, Motta REDA, Silva ABRD, Patricio TCDC, et al. Antimicrobial Activity of Psidium guajava Aqueous Extract against Sensitive and Resistant Bacterial Strains. Microorganisms. 2023;11: 1784. doi:10.3390/microorganisms11071784

35. Dongmo AN, Nguefack J, Dongmo JBL, Fouelefack FR, Azah RU, Nkengfack EA, et al. Chemical characterization of an aqueous extract and the essential oil of Tithonia diversifolia and their

biocontrol activity against seed-borne pathogens of rice. J Plant Dis Prot. 2021;128: 703–713. doi:10.1007/s41348-021-00439-w

36. Petrov V, Lyutskanov M, Kanakov D. Effects of spontaneous and experimental colibacteriosis on some haematological and blood biochemical parameters in weaned rabbits. Bulgarian Journal of Veterinary Medicine. 2011;14.

37. Anorue EC, Ogara AL, Joshua PE, Ibekwe DG, Igbokwe AI. Impact of Psidium guajava bark extract on inflammatory biomarkers in Wistar rat models of inflammation. Inflammopharmacology. 2025; 1–27.

38. Broering MF, Nunes R, De Faveri R, De Faveri A, Melato J, Correa TP, et al. Effects of Tithonia diversifolia (Asteraceae) extract on innate inflammatory responses. Journal of ethnopharmacology. 2019;242: 112041.

39. Zhou H-H, Tang Y-L, Xu T-H, Cheng B. C-reactive protein: structure, function, regulation, and role in clinical diseases. Front Immunol. 2024;15: 1425168. doi:10.3389/fimmu.2024.1425168

40. Georgieva TM, Georgiev IP, Iliev Y, Petrov VS, Vachkov A, Kanelov I, et al. Blood serum concentrations of total proteins and main protein fractions in weaning rabbits experimentally infected with E. coli. Rev Med Vet. 2008;159: 431–436.

41. ALi G IE, A.Y. T, Naena N AA, Abeer E-KMS, Al-Sokary ET, Elbaz S, et al. CINNAMON NANO PARTICLES (CNPs) AS FEED ADDITIVES ON RABBIT GROWTH PERFORMANCE, IMMUNITY, ANTIOXIDANT CAPACITY, AND RESISTANCE TO ESCHERICHIA COLI. Assiut Veterinary Medical Journal. 2025;71: 608–634. doi:10.21608/avmj.2025.320513.1399

42. Yabo SA, Manga SB, Baki AS, Atata RF, Gambo S, Tahir H. Antibacterial activity and toxicological evaluation of Anogeissusleiocarpus and Psidium guajava on Escherichia coli and Staphylococcus aureus. Bayero Journal of Pure and Applied Sciences. 2021;14: 214–220.

43. Sonawane R. A review on the medicinal plant-psidium Guajava Linn. Int J Pharm Res Appl. 2021;6: 311–329.

Figure Click here to access/download;Figure;Figures_200726.doc

Figures

Fig 1. Phenotypic characterization of the pathogenic E. coli isolate using the API 20E analytical profile index system. Photograph of an API 20E strip (bioMérieux, Marcy-l'Étoile, France) showing the colorimetric reaction pattern generated by the clinical isolate across the 20 miniaturised biochemical test compartments after 24 h of incubation at 37 °C. The resulting 7-digit numerical profile (5044572) identified the isolate as *Escherichia coli* with an excellent identification percentage (>99.9%), determined using the apiweb identification software (bioMérieux). The isolate was subsequently confirmed as pathogenic using the Congo red binding assay (see Methods) prior to use in the experimental challenge described in Figs 2–14.

Fig 2. Effect of the combined aqueous extracts of *Psidium guajava* and *Tithonia diversifolia* on the body weight variation in colibacillosis-infected rabbits. Longitudinal relative body weight variation (%, expressed relative to the Day 0 pre-infection baseline) monitored over 8 days following *Escherichia coli* challenge in six experimental groups: Normal (uninfected, untreated control); Immunosuppressed (uninfected, cyclophosphamide-only control); Untreated (infected, untreated); Neomycin (infected, treated with neomycin, 10 mg/kg body weight (bw), reference antibiotic); 400 mg/kg bw (infected, treated with the combined extract at the low dose); and 800 mg/kg bw (infected, treated with the combined extract at the high dose). Data are mean ± standard deviation (SD), n = 4 animals per group. Group differences at each time point were assessed by two-way repeated-measures ANOVA followed by Tukey's honestly significant difference (HSD) post-hoc test (GraphPad Prism version 8.0.1; GraphPad Software, San Diego, CA, USA); $P < 0.05$ was considered significant.

Fig 3. Relative organ weights at sacrifice (Day 7 post-infection) in rabbits challenged with *Escherichia coli* and treated with the combined *Psidium guajava* and *Tithonia diversifolia* extracts. Boxplots of relative organ weights (g organ weight per 100 g body weight) for the heart, kidney, liver, lung and spleen, recorded at necropsy on Day 7 post-infection. Boxes show the median and interquartile range (IQR); whiskers extend to the most extreme values within 1.5 × IQR; individual points represent single animals (n = 4 per group). Experimental groups (Normal, Immunosuppressed, Untreated, Neomycin, 400 mg/kg, 800 mg/kg) are as defined in Fig 2. Between-group differences were assessed by one-way ANOVA followed by Tukey's HSD post-hoc test (GraphPad Prism v8.0.1); $P < 0.05$ was considered significant.

Fig 4. Fecal *Escherichia coli* shedding kinetics in rabbits treated with the combined *Psidium guajava* and *Tithonia diversifolia* extracts. Longitudinal fecal bacterial load, expressed as colony-forming units (CFU) × 10^7 per 10 mg of stool, monitored across the study timeline in five experimental groups: Normal, Untreated, Neomycin (10 mg/kg bw), 400 mg/kg bw and 800 mg/kg bw (group definitions as in Fig 2). Timeline phases are indicated above the plot: D, dietary deprivation/acclimatisation period; ID, immunosuppression (cyclophosphamide administration, Day 3); IF, bacterial

infection/challenge (Day 5); T, treatment period; S, sacrifice (Day 13). Data are mean ± SD, n = 4 animals per group.

Fig 5. Effect of the combined *Psidium guajava* and *Tithonia diversifolia* extracts on intestinal oxidative stress markers on Day 7 post-infection. Boxplots comparing oxidative stress and antioxidant biomarkers in the caecum (left column) and ileum (right column): catalase (CAT) activity, expressed in millimoles of hydrogen peroxide (H_2O_2) consumed per minute per gram of tissue; reduced glutathione (GSH) concentration, expressed as $\times 10^{-6}$ µmol per gram of tissue; malondialdehyde (MDA) concentration, an indicator of lipid peroxidation, expressed as $\times 10^{-6}$ µmol per gram of tissue; and superoxide dismutase (SOD) activity, expressed in units per gram of tissue. Experimental groups (Normal, Untreated, Neomycin, 400 mg/kg, 800 mg/kg, Immunosuppressed) are as defined in Fig 2; n = 4 animals per group. Boxes show median and IQR; whiskers extend to 1.5 × IQR. Between-group comparisons: one-way ANOVA with Tukey's HSD post-hoc test (GraphPad Prism v8.0.1); $P < 0.05$ was considered significant.

Fig 6. Hematological profile of rabbits on Day 7 post-infection following treatment with the combined *Psidium guajava* and *Tithonia diversifolia* extracts. Boxplots of hematological parameters: granulocyte percentage (GRA %); hematocrit (HCT, %); hemoglobin concentration (HGB, g/dL); lymphocyte percentage (LYM %); percentage of mid-sized cells (MID %; combined monocyte, eosinophil and basophil fraction); platelet count (PLT, $\times 10^3$/µL); red blood cell count (RBC, $\times 10^6$/µL); and white blood cell count (WBC, $\times 10^3$/µL). Six experimental groups as defined in Fig 2; n = 4 animals per group. Boxes show median and IQR; whiskers extend to 1.5 × IQR; individual points represent single animals. Between-group comparisons: one-way ANOVA with Tukey's HSD post-hoc test (GraphPad Prism v8.0.1); $P < 0.05$ was considered significant.

Fig 7. Systemic cytokine and acute-phase protein modulation by the combined *Psidium guajava* and *Tithonia diversifolia* extracts on Day 7 post-infection. Boxplots of serum concentrations of C-reactive protein (CRP, ng/mL), interferon-gamma (IFN-γ, ng/mL), interleukin-6 (IL-6, pg/mL), interleukin-10 (IL-10, pg/mL), interleukin-1β (IL-1β, pg/mL), interleukin-3 (IL-3, pg/mL) and tumor necrosis factor-alpha (TNF-α, pg/mL). Six experimental groups as defined in Fig 2; n = 4 animals per group, except where individual values fell below the analytical lower limit of detection, in which case the corresponding data point is omitted from the panel. Between-group comparisons: one-way ANOVA with Tukey's HSD post-hoc test (GraphPad Prism v8.0.1); $P < 0.05$ was considered significant.

Fig 8. Hepatic and renal function markers in rabbits treated with the combined *Psidium guajava* and *Tithonia diversifolia* extracts on Day 7 post-infection. Boxplots of serum alanine aminotransferase (ALAT, IU/L), aspartate aminotransferase (ASAT, IU/L), creatinine (mg/dL), gamma-glutamyl transferase (GGT, IU/L), total protein (mg/dL) and uric acid (mg/dL) concentrations. Six experimental groups as defined in Fig 2; n = 4 animals per group. Between-group comparisons: one-way ANOVA with Tukey's HSD post-hoc test (GraphPad Prism v8.0.1); $P < 0.05$ was considered significant.

Fig 9. Two-way hierarchical clustering heatmap of the integrated physiological profile of rabbits at the study endpoint. Row-standardised (Z-score) heatmap of measured parameters

(rows) grouped into five physiological categories: relative organ weights, hematological markers, serum biochemical markers, intestinal antioxidant markers, and longitudinal rabbit body weight (Days 7, 9, 11 and 13 post-infection). Columns represent the six experimental groups (800 mg/kg, Neomycin, 400 mg/kg, Normal, Immunosuppressed, Untreated), each summarising n = 4 animals. Red indicates values above the overall parameter mean (Z-score up to +2), blue indicates values below the mean (Z-score down to −2), and white/pale pink indicates values near the mean. Row and column dendrograms represent unsupervised hierarchical clustering based on Euclidean distance with complete linkage. Parameter abbreviations: GRA %, granulocyte percentage; LYM %, lymphocyte percentage; MID %, mid-sized cell percentage; WBC, white blood cells; PLT, platelets; HGB, hemoglobin; RBC, red blood cells; HCT, hematocrit; CHO, total cholesterol; ASAT, aspartate aminotransferase; LDL, low-density lipoprotein; GGT, gamma-glutamyl transferase; ALAT, alanine aminotransferase; TG, triglycerides; HDL, high-density lipoprotein; SOD, superoxide dismutase; GSH, reduced glutathione; MDA, malondialdehyde.

Fig 10. Multivariate trajectory and key biological drivers of the therapeutic response. (A) Unsupervised principal component analysis (PCA) score plot of individual rabbit multi-parametric profiles. Dimension 1 (Dim1) accounts for 48% and Dimension 2 (Dim2) for 9.7% of total variance. Small circles represent individual animals; large filled circles represent group centroids, each surrounded by its 95% confidence ellipse (Extract 400, grey; Extract 800, yellow; Neomycin, blue; Normal, green; Untreated, orange). **(B)** Bar plot of the top 10 parameters contributing to Dim1, expressed as percentage contribution; the dashed red line indicates the theoretical uniform contribution threshold (≈ 2.17%).

Fig 11. Comparative efficacy of the 800 mg/kg combined extract dose versus the reference antibiotic neomycin on Day 7 post-infection. Bar charts comparing mean ± standard error of the mean (SEM) values for the 800 mg/kg combined-extract group (yellow, n = 4) and the neomycin, 10 mg/kg group (grey, n = 4) across three physiological domains: antioxidant markers (caecal and ileal catalase, malondialdehyde (MDA) and superoxide dismutase (SOD)); the acute-phase marker C-reactive protein (CRP, ng/mL); and the hematological marker white blood cell count (WBC, $\times 10^3/\mu L$). Groups were compared for each parameter using an unpaired two-tailed t-test (or the non-parametric equivalent where normality assumptions were not met; GraphPad Prism v8.0.1). *Note: No statistically significant differences ($P > 0.05$) were observed between the two groups for any parameter shown, indicating comparable recovery at these specific endpoints. This finding is limited to the parameters tested and should not be interpreted as evidence of overall clinical, pharmacokinetic, or safety equivalence (see Discussion, Limitations of the Study).*

Fig 12. Photomicrographs of rabbit kidney sections following *Escherichia coli* challenge and treatment with the combined *Psidium guajava* and *Tithonia diversifolia* extracts (hematoxylin and eosin (H&E) stain; original magnification ×100; scale bar = 10µm). T0, uninfected control receiving distilled water only (normal control); T1, infected, untreated (vehicle control); T2, infected, treated with neomycin (10 mg/kg bw); T3 and T4, infected, treated with the combined extract at 400 and 800 mg/kg bw, respectively; T5, uninfected, cyclophosphamide-only (immunosuppressed) control. n = 4 kidneys examined per group; one representative field is shown per group. Abbreviations: Cd, cell degeneration; US, urinary space; G, glomerulus; IL, leukocyte infiltration; DT, distal convoluted tubule; PT, proximal

convoluted tubule. *Note: Histopathological evaluation was descriptive/qualitative, performed by a blinded pathologist; semi-quantitative lesion scoring was not performed in the present study (see Discussion, Limitations of the Study).*

Fig 13. Photomicrographs of rabbit liver sections following *Escherichia coli* challenge and treatment with the combined *Psidium guajava* and *Tithonia diversifolia* extracts (hematoxylin and eosin (H&E) stain; original magnification ×100; scale bar = 10 µm). Group designations (T0–T5) are as defined in the Fig 12 legend; n = 4 livers examined per group; one representative field is shown per group. Abbreviations: Cd, cell degeneration; He, hepatocytes; HA, hepatic artery; PV, portal vein; BC, bile canaliculus; IL, leukocyte infiltration. *Note: Histopathological evaluation was descriptive/qualitative (see Fig 12 legend and Discussion, Limitations of the Study).*

Fig 14. Photomicrographs of rabbit small intestine sections following *Escherichia coli* challenge and treatment with the combined *Psidium guajava* and *Tithonia diversifolia* extracts (hematoxylin and eosin (H&E) stain; original magnification ×100; scale bar = 10 µm).Group designations (T0–T5) are as defined in the Fig 12 legend; n = 4 intestinal segments examined per group; one representative field is shown per group. Abbreviations: Cd, cell degeneration; Lu, intestinal lumen; Mu, mucosa; Smu, submucosa; Mus, muscularis; Se, serosa. *Note: Villus height and crypt depth were not measured quantitatively; evaluation was descriptive/qualitative (see Fig 12 legend and Discussion, Limitations of the Study).*

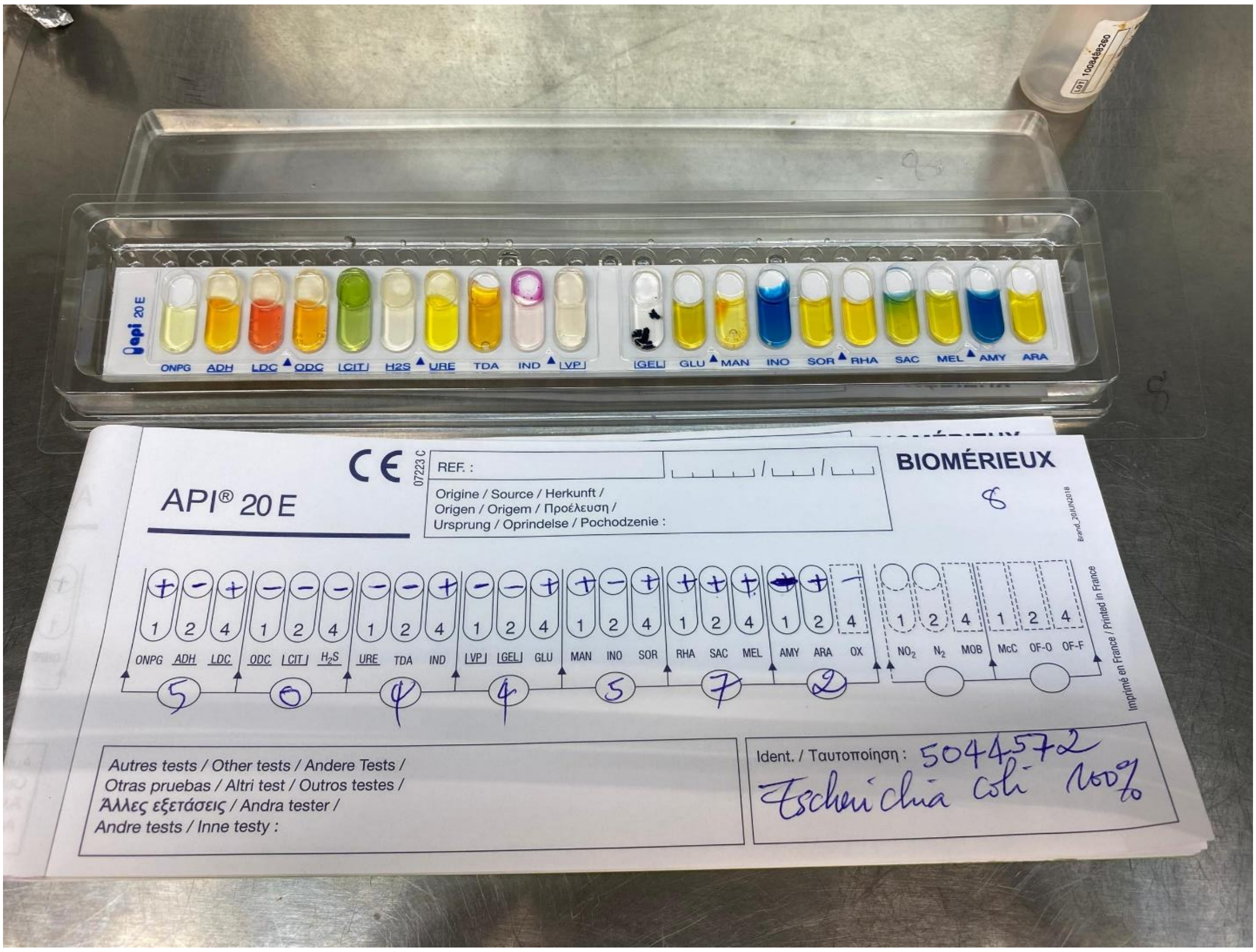
ONPG
ADH
LDC
ODC
CIT
H2S
URE
TDA
IND
VP
GEL
GLU
MAN
INO
SOR
RHA
SAC
MEL
AMY
ARA
API® 20 E
BIOMÉRIEUX
REF. :
Origine / Source / Herkunft /
Origen / Origem / Προέλευση /
Ursprung / Oprindelse / Pochodzenie :
Autres tests / Other tests / Andere Tests /
Otras pruebas / Altri test / Outros testes /
Άλλες εξετάσεις / Andra tester /
Andre tests / Inne testy :
Ident. / Ταυτοποίηση : 5044572
Escherichia coli 100%

Fig. 1

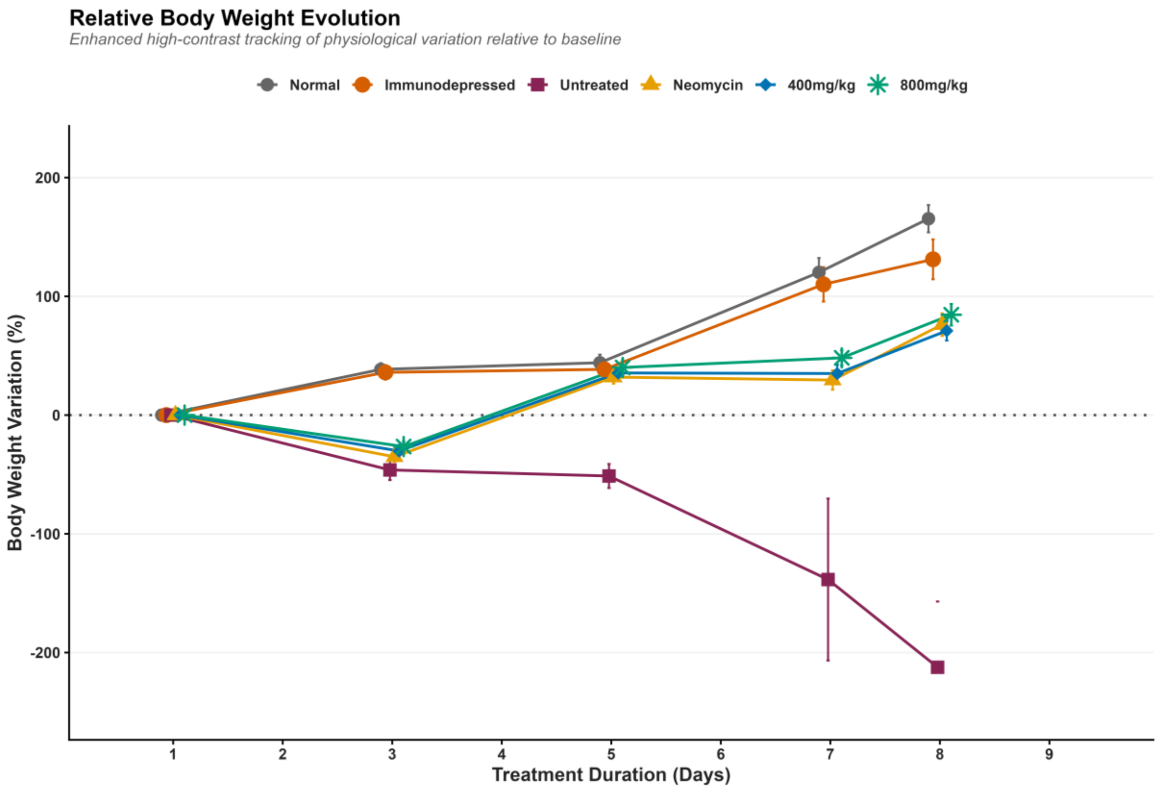

Relative Body Weight Evolution
Enhanced high-contrast tracking of physiological variation relative to baseline
Normal
Immunodepressed
Untreated
Neomycin
400mg/kg
800mg/kg
Body Weight Variation (%)
200
100
0
-100
-200
1
2
3
4
5
6
7
8
9
Treatment Duration (Days)

Fig. 2

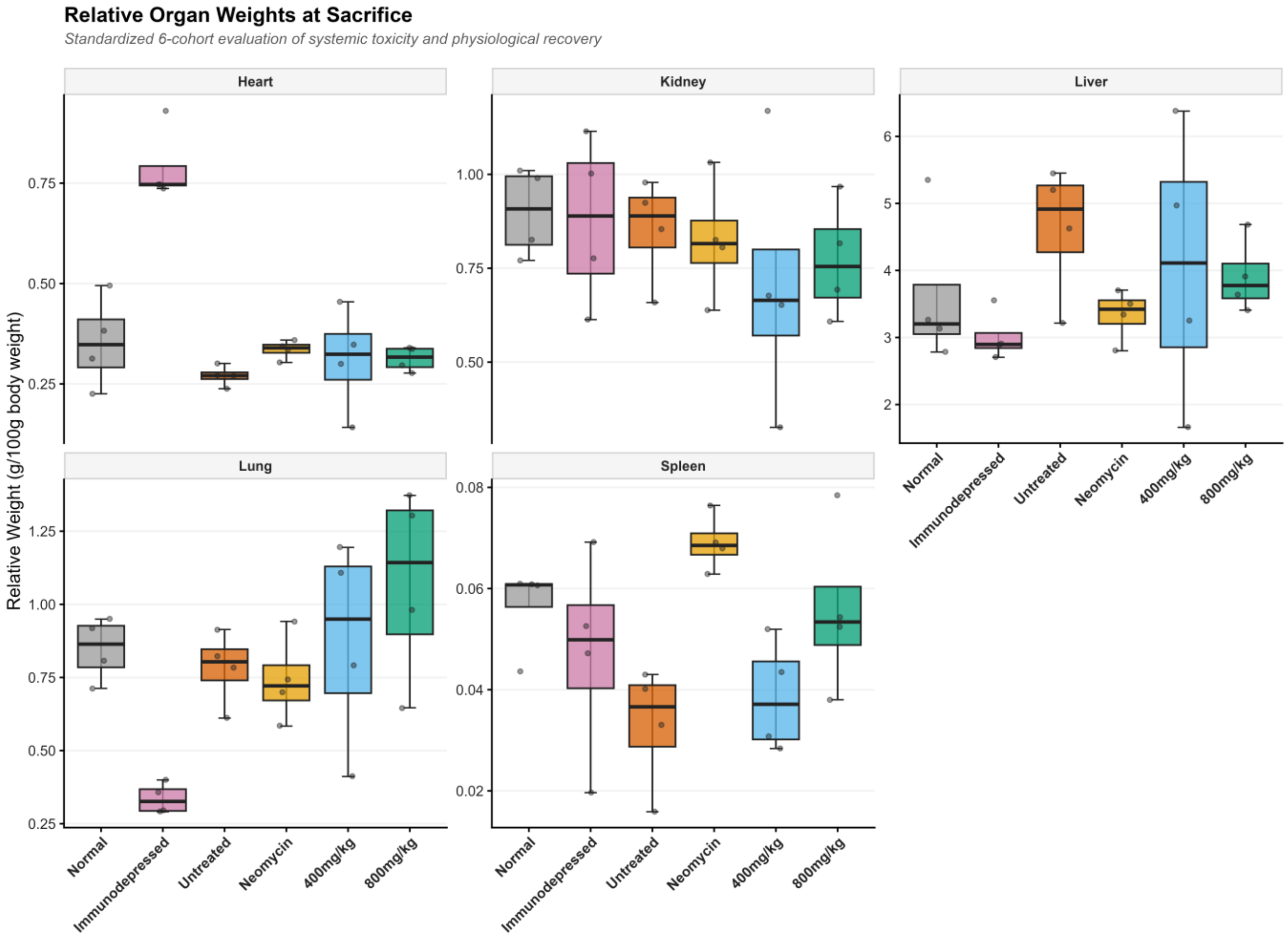
Relative Organ Weights at Sacrifice
Standardized 6-cohort evaluation of systemic toxicity and physiological recovery
Heart
Kidney
Liver
Lung
Spleen
Relative Weight (g/100g body weight)
Normal
Immunodepressed
Untreated
Neomycin
400mg/kg
800mg/kg

Fig. 3

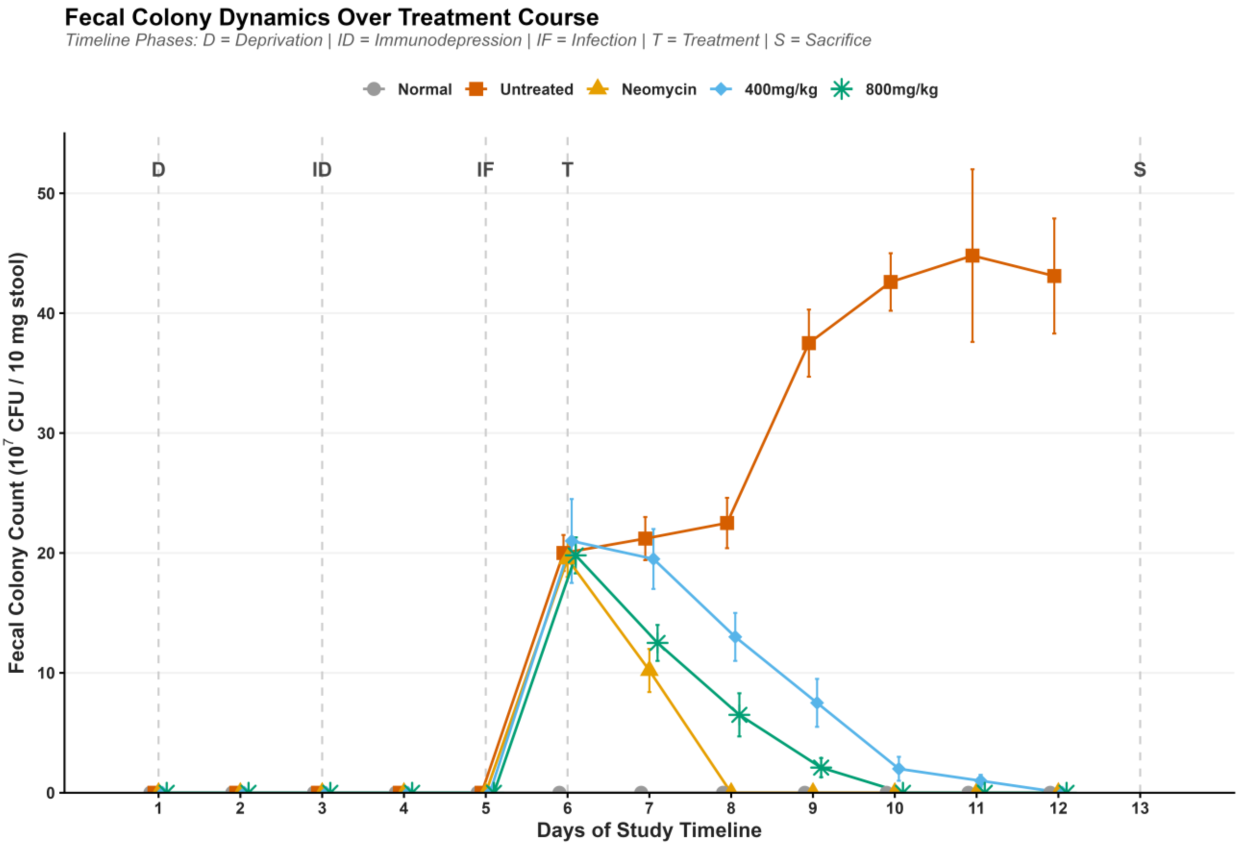

Fecal Colony Dynamics Over Treatment Course
Timeline Phases: D = Deprivation | ID = Immunodepression | IF = Infection | T = Treatment | S = Sacrifice
Normal
Untreated
Neomycin
400mg/kg
800mg/kg
D
ID
IF
T
S
Fecal Colony Count (10^7 CFU / 10 mg stool)
Days of Study Timeline
0
10
20
30
40
50
1
2
3
4
5
6
7
8
9
10
11
12
13

Fig. 4

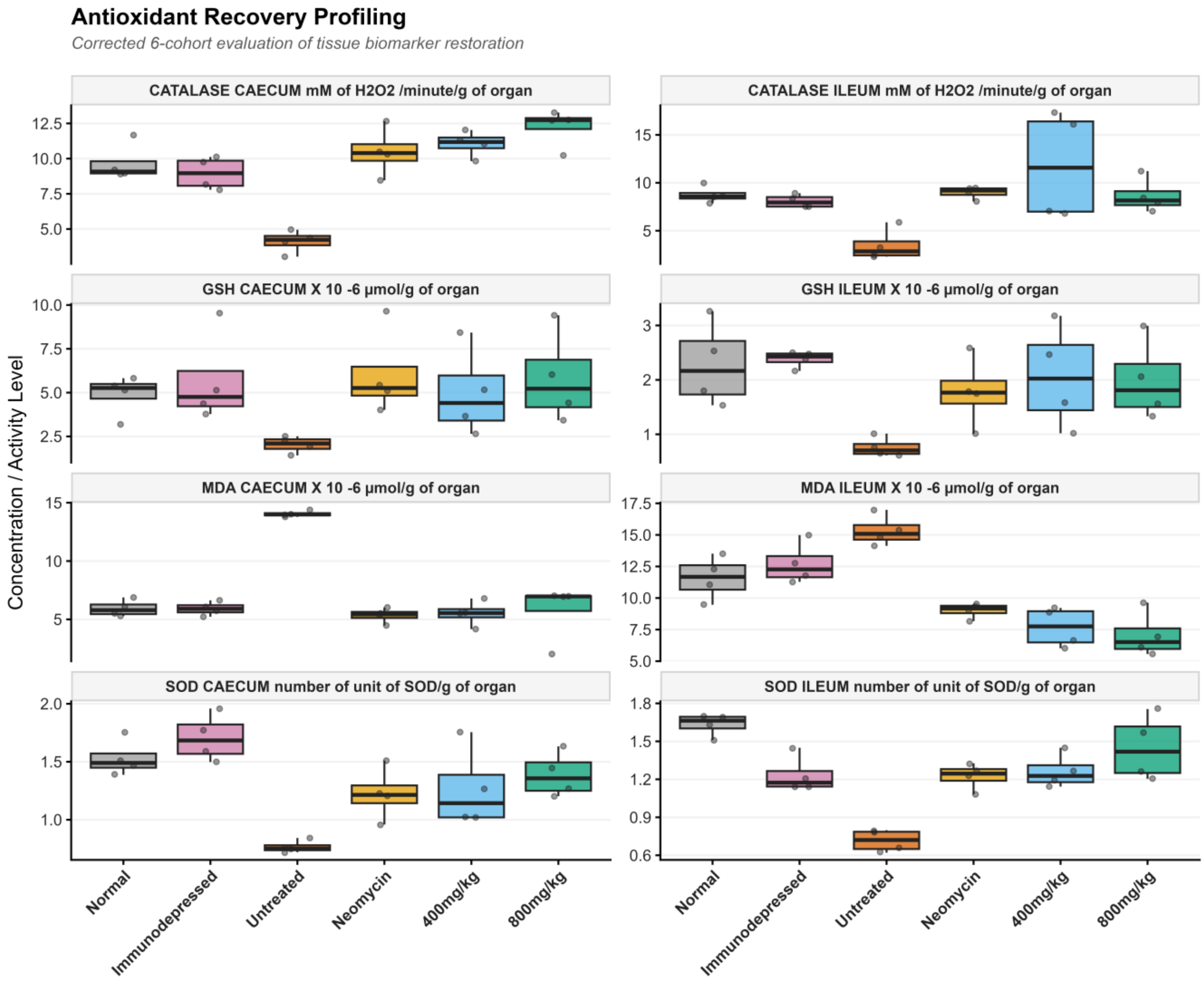
Antioxidant Recovery Profiling
Corrected 6-cohort evaluation of tissue biomarker restoration
Concentration / Activity Level
CATALASE CAECUM mM of H2O2 /minute/g of organ
CATALASE ILEUM mM of H2O2 /minute/g of organ
GSH CAECUM X 10 -6 µmol/g of organ
GSH ILEUM X 10 -6 µmol/g of organ
MDA CAECUM X 10 -6 µmol/g of organ
MDA ILEUM X 10 -6 µmol/g of organ
SOD CAECUM number of unit of SOD/g of organ
SOD ILEUM number of unit of SOD/g of organ
Normal
Immunodepressed
Untreated
Neomycin
400mg/kg
800mg/kg

Fig. 5

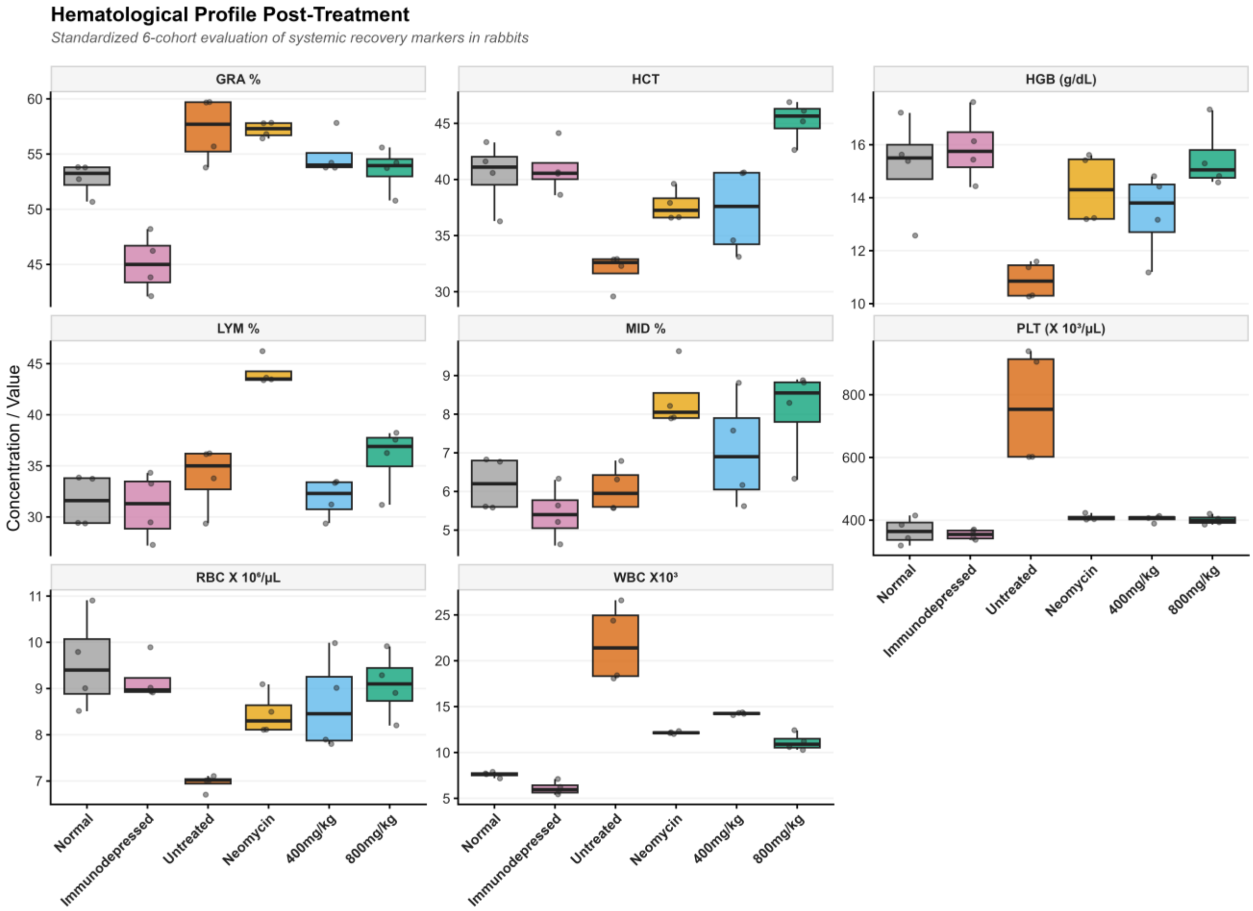
Hematological Profile Post-Treatment
Standardized 6-cohort evaluation of systemic recovery markers in rabbits
GRA %
HCT
HGB (g/dL)
LYM %
MID %
PLT (X 10³/μL)
RBC X 10⁶/μL
WBC X10³
Concentration / Value
Normal
Immunodepressed
Untreated
Neomycin
400mg/kg
800mg/kg

Fig.6

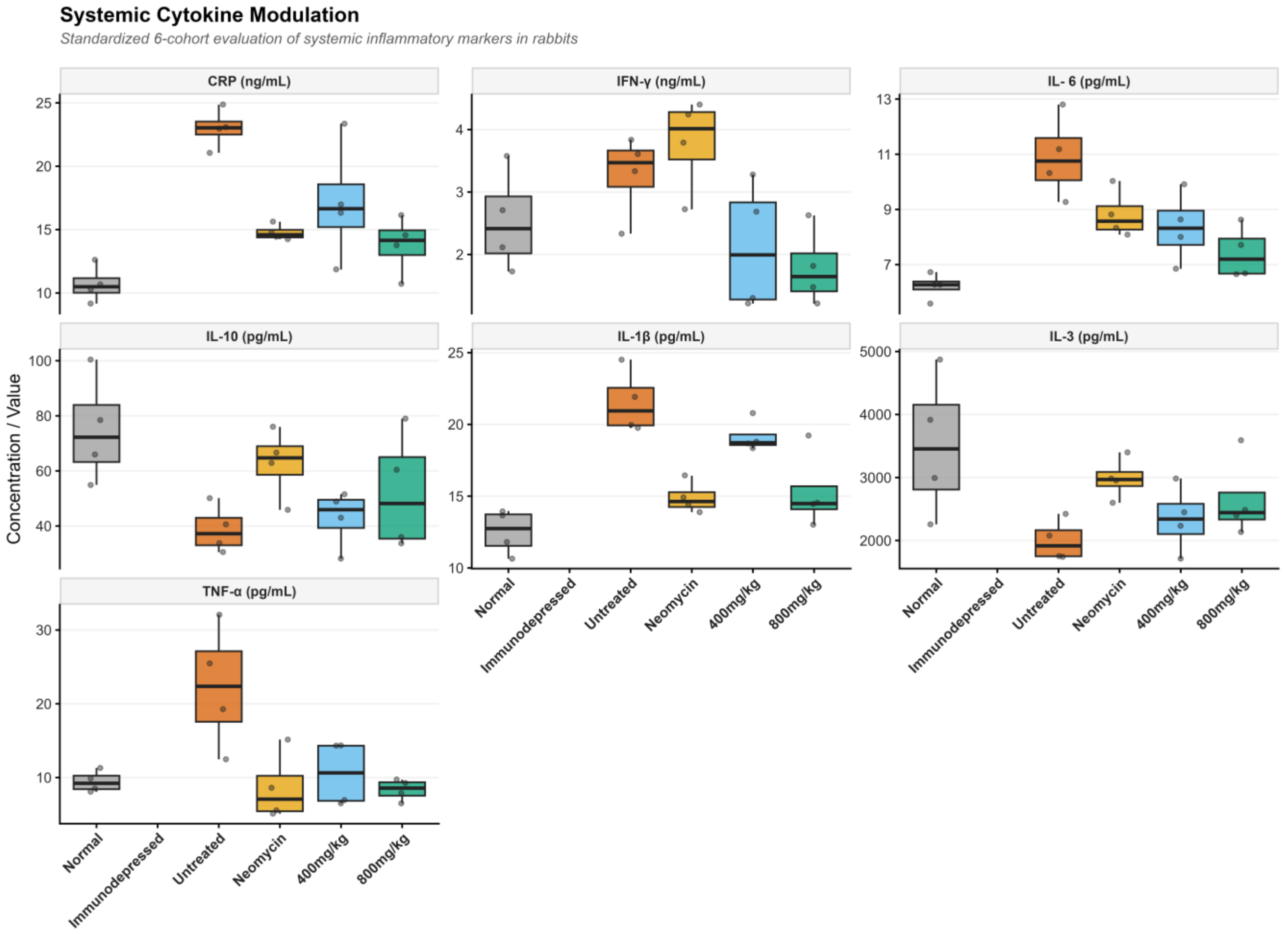

Systemic Cytokine Modulation
Standardized 6-cohort evaluation of systemic inflammatory markers in rabbits
CRP (ng/mL)
IFN-γ (ng/mL)
IL- 6 (pg/mL)
IL-10 (pg/mL)
IL-1β (pg/mL)
IL-3 (pg/mL)
TNF-α (pg/mL)
Concentration / Value
Normal
Immunodepressed
Untreated
Neomycin
400mg/kg
800mg/kg

Fig.7

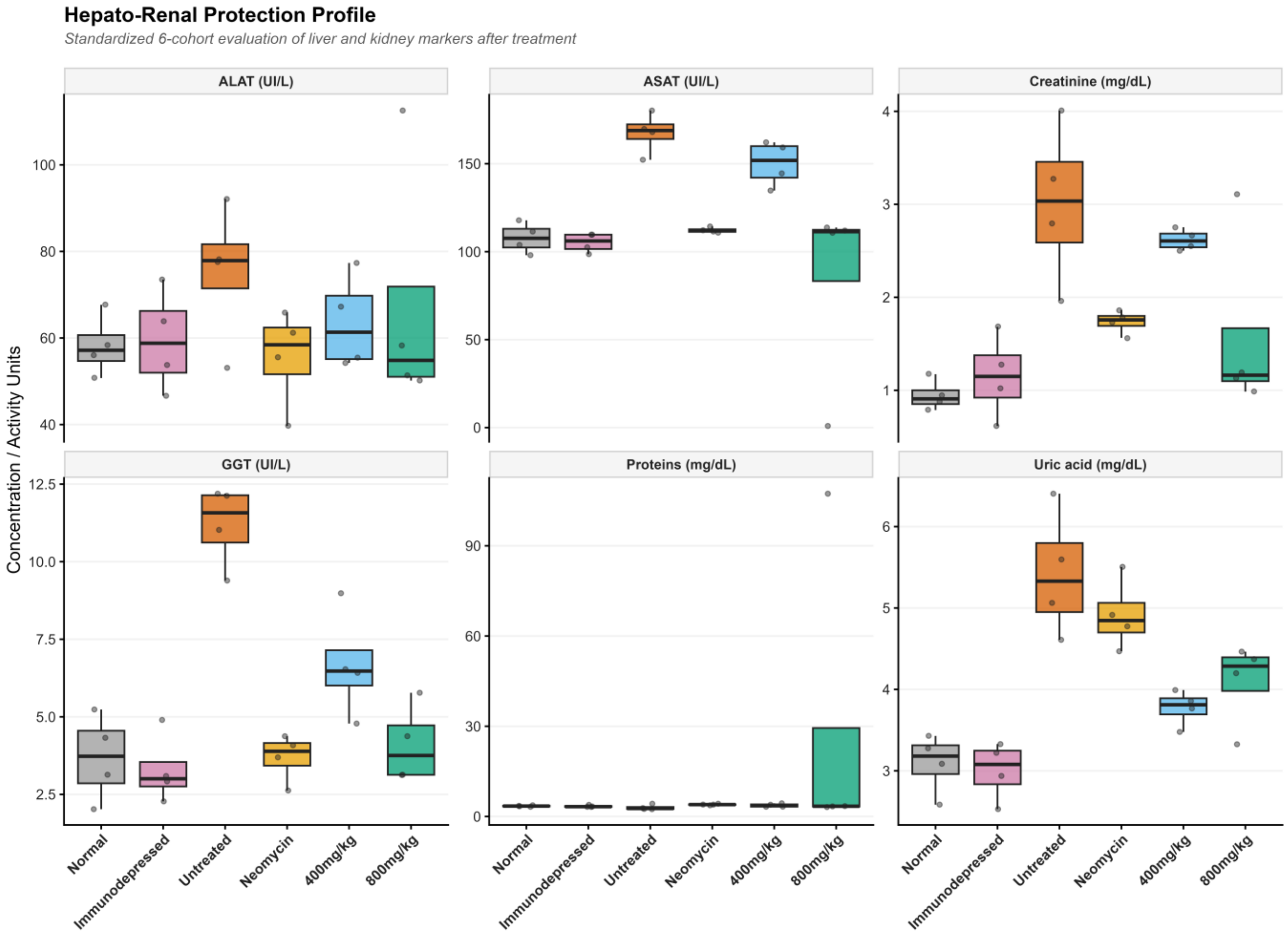
Hepato-Renal Protection Profile
Standardized 6-cohort evaluation of liver and kidney markers after treatment
ALAT (UI/L)
ASAT (UI/L)
Creatinine (mg/dL)
GGT (UI/L)
Proteins (mg/dL)
Uric acid (mg/dL)
Concentration / Activity Units
Normal
Immunodepressed
Untreated
Neomycin
400mg/kg
800mg/kg

Fig. 8

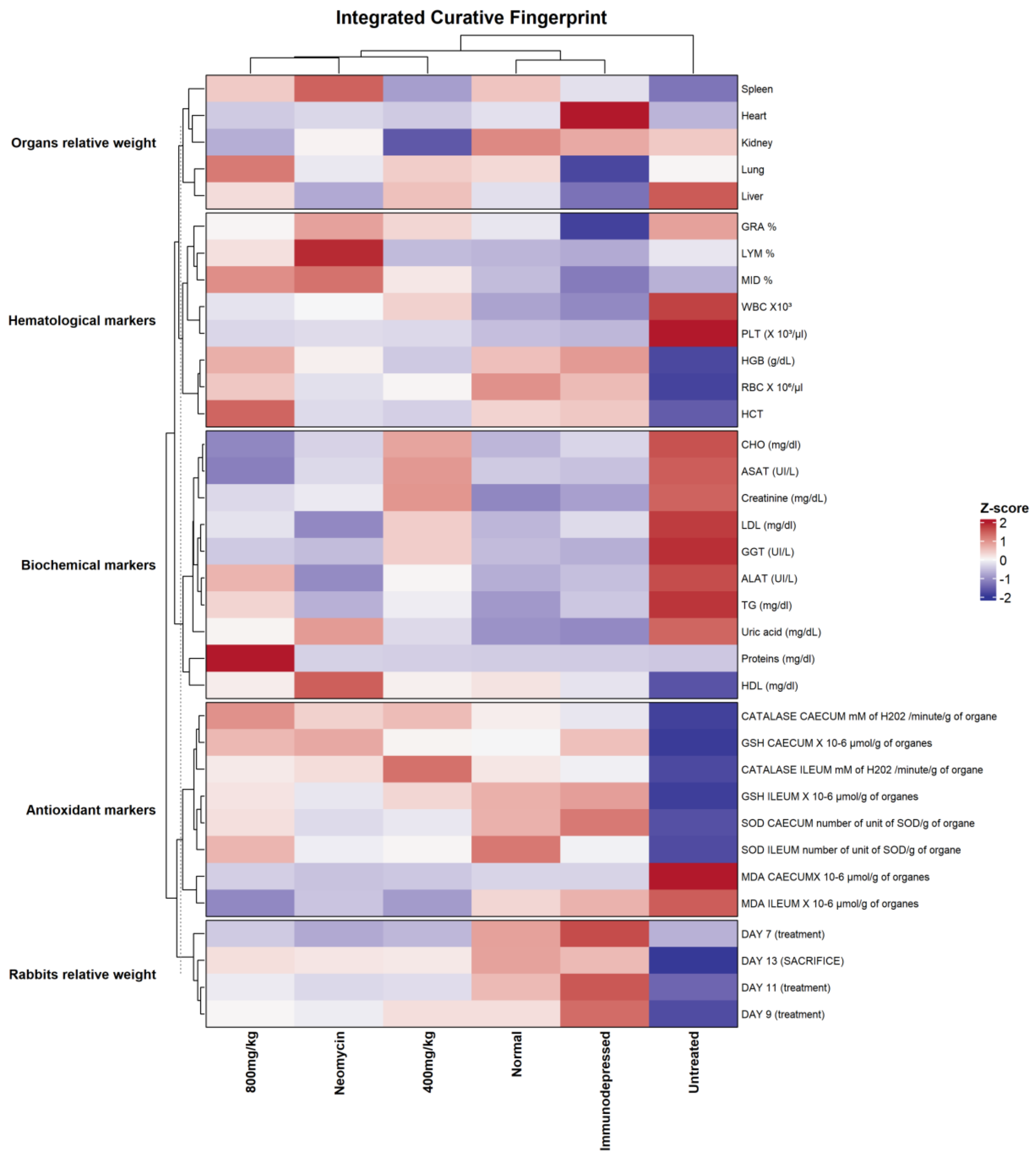
Integrated Curative Fingerprint
Organs relative weight
Hematological markers
Biochemical markers
Antioxidant markers
Rabbits relative weight
Spleen
Heart
Kidney
Lung
Liver
GRA %
LYM %
MID %
WBC X10³
PLT (X 10³/µl)
HGB (g/dL)
RBC X 10⁶/µl
HCT
CHO (mg/dl)
ASAT (UI/L)
Creatinine (mg/dL)
LDL (mg/dl)
GGT (UI/L)
ALAT (UI/L)
TG (mg/dl)
Uric acid (mg/dL)
Proteins (mg/dl)
HDL (mg/dl)
CATALASE CAECUM mM of H202 /minute/g of organe
GSH CAECUM X 10-6 µmol/g of organes
CATALASE ILEUM mM of H202 /minute/g of organe
GSH ILEUM X 10-6 µmol/g of organes
SOD CAECUM number of unit of SOD/g of organe
SOD ILEUM number of unit of SOD/g of organe
MDA CAECUMX 10-6 µmol/g of organes
MDA ILEUM X 10-6 µmol/g of organes
DAY 7 (treatment)
DAY 13 (SACRIFICE)
DAY 11 (treatment)
DAY 9 (treatment)
800mg/kg
Neomycin
400mg/kg
Normal
Immunodepressed
Untreated
Z-score
2
1
0
-1
-2

Fig. 9

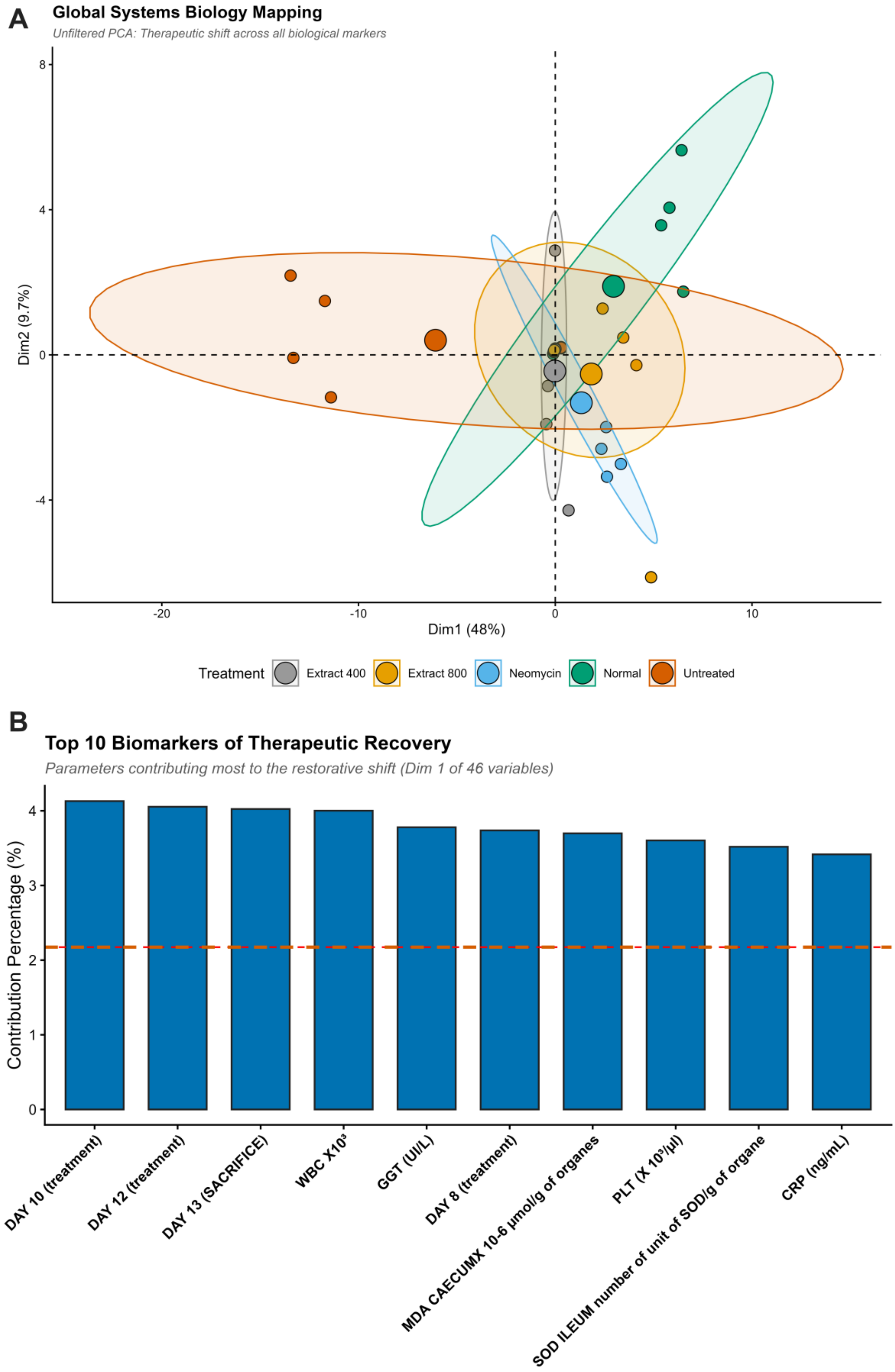
A
Global Systems Biology Mapping
Unfiltered PCA: Therapeutic shift across all biological markers
Dim2 (9.7%)
Dim1 (48%)
8
4
0
-4
-20
-10
0
10
Treatment
Extract 400
Extract 800
Neomycin
Normal
Untreated
B
Top 10 Biomarkers of Therapeutic Recovery
Parameters contributing most to the restorative shift (Dim 1 of 46 variables)
Contribution Percentage (%)
4
3
2
1
0
DAY 10 (treatment)
DAY 12 (treatment)
DAY 13 (SACRIFICE)
WBC X10³
GGT (UI/L)
DAY 8 (treatment)
MDA CAECUMX 10-6 µmol/g of organes
PLT (X 10³/µl)
SOD ILEUM number of unit of SOD/g of organe
CRP (ng/mL)

Fig. 10

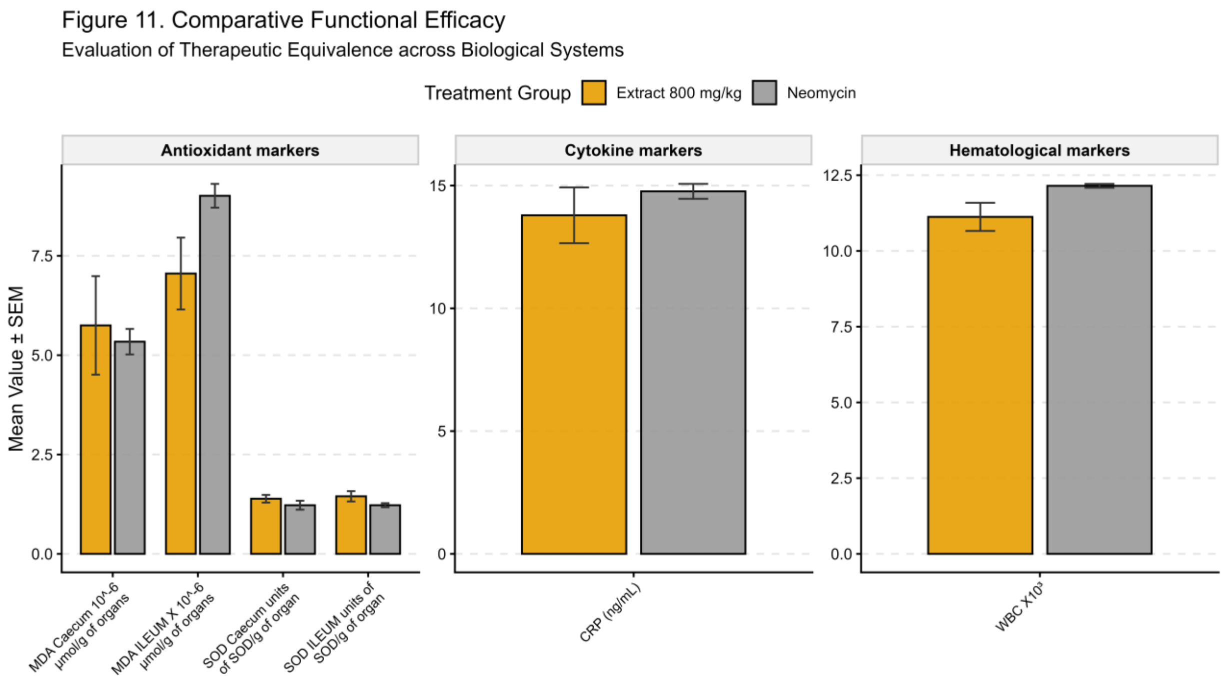
Figure 11. Comparative Functional Efficacy
Evaluation of Therapeutic Equivalence across Biological Systems
Treatment Group
Extract 800 mg/kg
Neomycin
Antioxidant markers
Cytokine markers
Hematological markers
Mean Value ± SEM
MDA Caecum 10^-6 µmol/g of organs
MDA ILEUM X 10^-6 µmol/g of organs
SOD Caecum units of SOD/g of organ
SOD ILEUM units of SOD/g of organ
CRP (ng/mL)
WBC X10³

Fig. 11

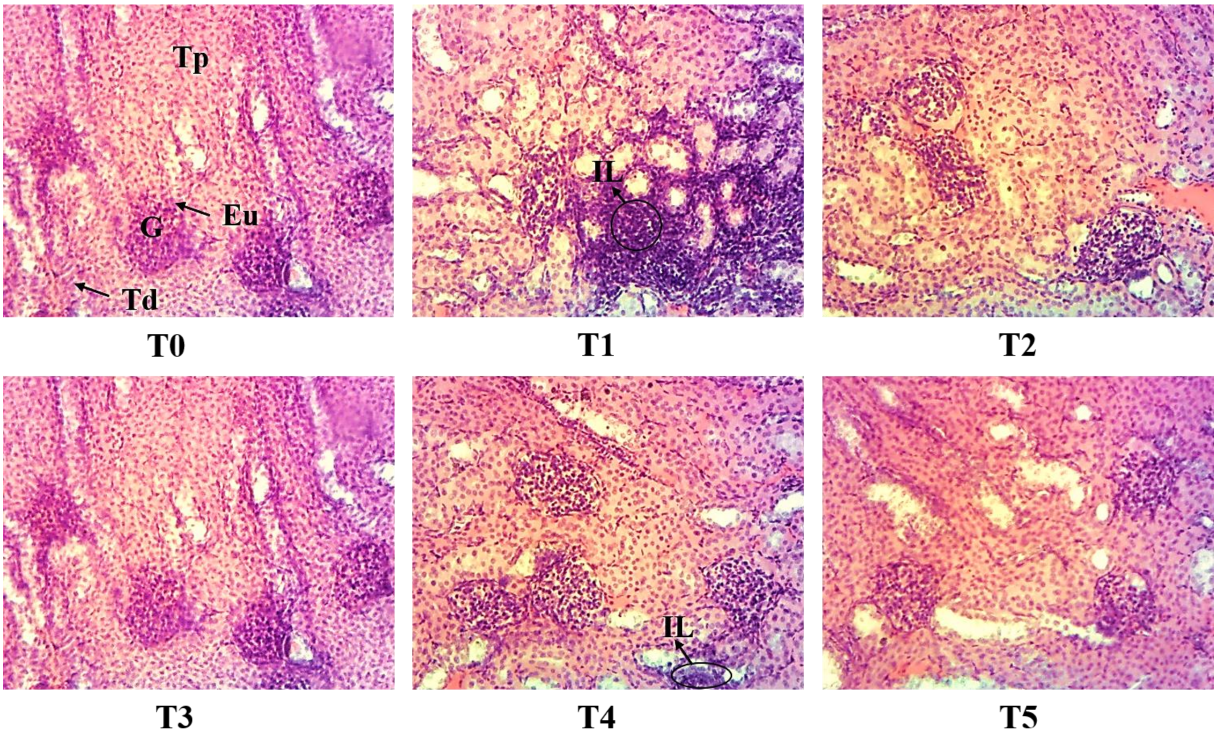

Fig. 12

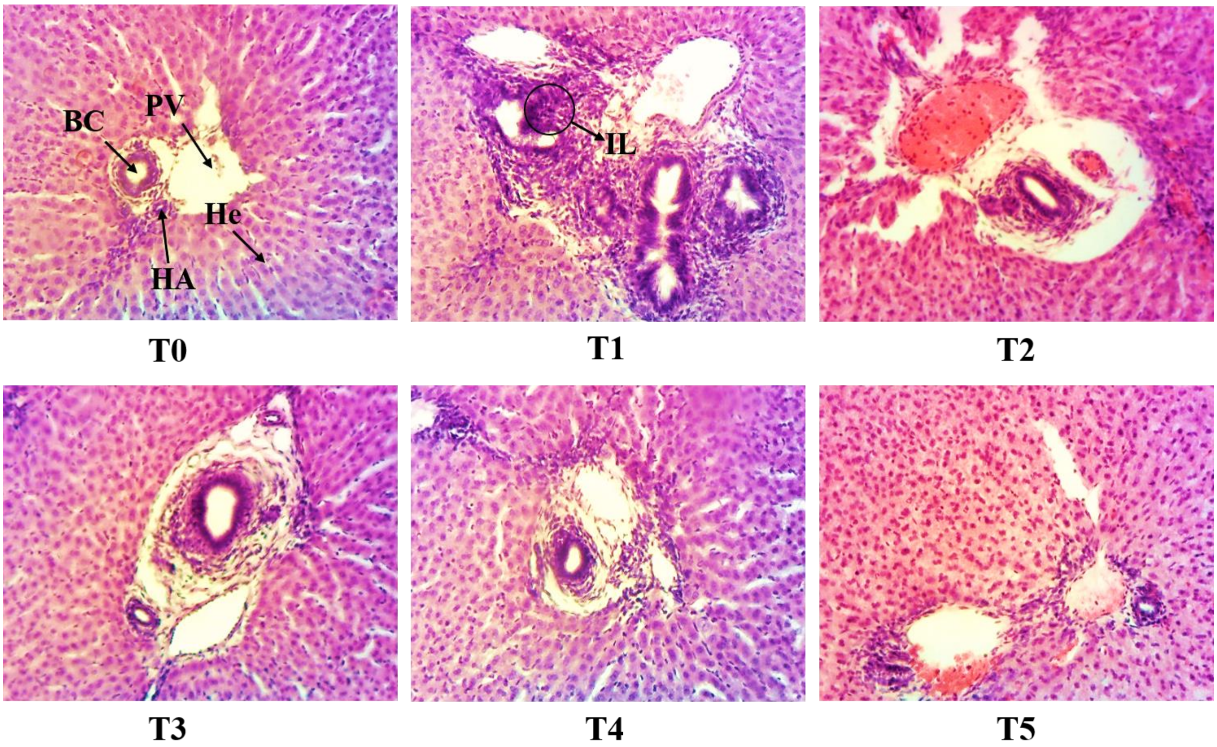

Fig. 13

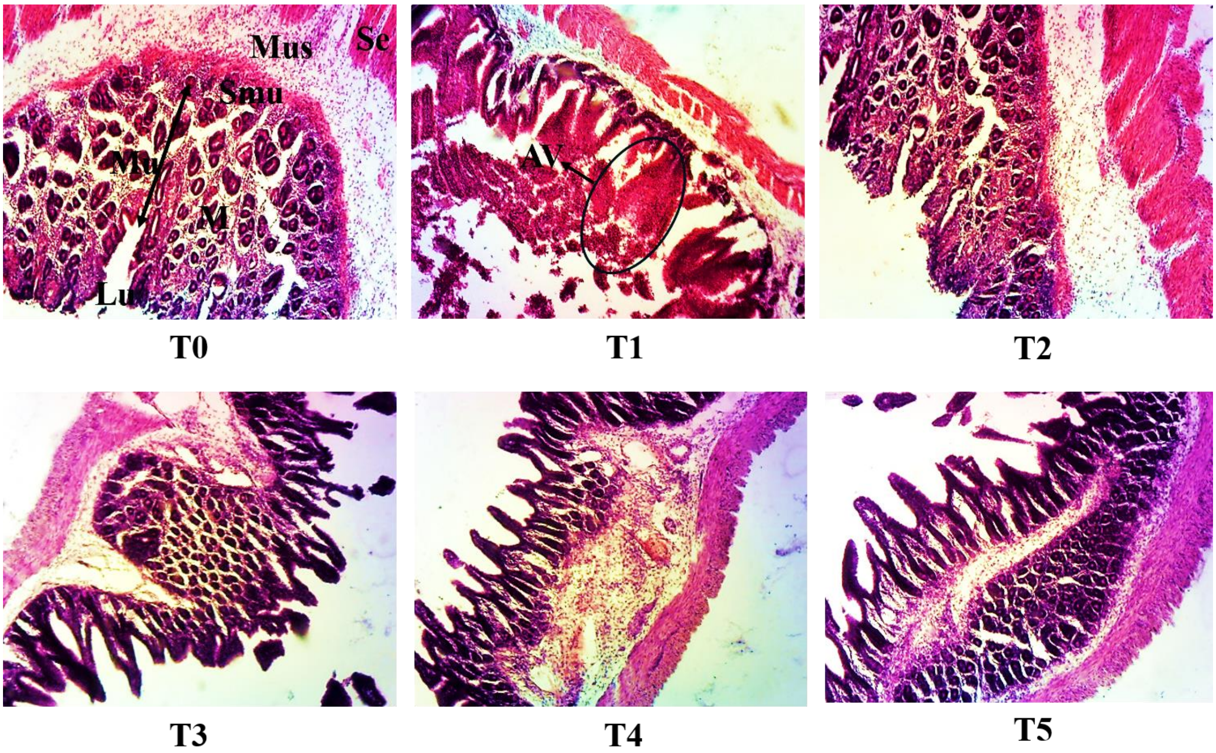

Fig.14

Click here to access/download
Supporting Information
Supplementary materials.docx

A Combined Aqueous Extract of *Psidium guajava* L. and *Tithonia diversifolia* (Hemsl.) A.Gray Improves Metabolic Homeostasis and Preserves Intestinal Integrity in Experimental Rabbit Colibacillosis

Mireille Tuedom Chouegouong[1,2], Rufin Marie Kouipou Toghueo[1,3*], Yanick Kevin Melogmo Dongmo[1], Jaurès Marius Tsakem Nangap[4], Michele Stella Majoumouo[1,5], Brice Pinlap[1] Kingsley Agbor Etchu[2], Fabrice Fekam Boyom[1*]

[1]Antimicrobial and Biocontrol Agents Unit (AmBcAU), Laboratory for Phytobiochemistry and Medicinal Plants Studies, Department of Biochemistry, Faculty of Science, University of Yaoundé I, P.O. Box 812, Messa-Yaoundé, Cameroon
[2]Institute of Agricultural Research and Development (IRAD), P.O. Box 2067, Yaoundé, Cameroon
[3]Laboratory for Antibiotic Resistance and Microbial Metabolomics, Institute of Microbiology, Czech Academy of Sciences, Videnska 1083, 142 20 Prague 4, Czech Republic
[4]Laboratory of Animal Physiology, Department of Animal Biology and Physiology, Faculty of Science, University of Yaoundé 1, PO Box 812, Yaoundé, Cameroon
[5]Division of Pulmonary, Critical Care & Sleep Medicine, Department of Internal Medicine, University of Cincinnati College of Medicine

Corresponding: toghueo.rufin@yahoo.fr (RMKT); fabrice.boyom@fulbrightmail.org (FFB)

Abstract

Background

Colibacillosis, caused by pathogenic *Escherichia coli*, remains a significant threat to rabbit production, particularly during the critical weaning period. With the emergence of multidrug-resistant strains limiting conventional antibiotic efficacy, there is an urgent need for sustainable phytotherapeutic alternatives. This study evaluated the therapeutic efficacy of a combined aqueous leaf extract of *Psidium guajava* L. and *Tithonia diversifolia* (Hemsl.) A. Gray on systemic biological markers and organ integrity in a rabbit model of colibacillosis.

Methods

Thirty healthy rabbits were randomly grouped into six cohorts: negative control (infected-untreated), healthy control (uninfected-untreated), positive control (infected-treated with Neomycin 10 mg/kg), immunodepressed (uninfected-cyclophosphamide), and two treatment groups (infected; receiving 400 or 800 mg/kg of the combined *P. guajava* and *T. diversifolia* extract). Colibacillosis was induced via oral administration of a pathogenic *E. coli* isolate (1 mL/100 g body weight; 10^9 CFU/mL). Systemic recovery was assessed through hematological, biochemical, and antioxidant profiling, alongside histological examination and multivariate Principal Component Analysis (PCA).

Formatted: Line spacing: 1.5 lines

Formatted: Line spacing: 1.5 lines

Results

E. coli infection triggered severe haematological, biochemical, and inflammatory dysregulation, characterized by significant increases in white blood cell counts, aspartate aminotransferase, creatinine, and triglycerides, concomitant with reduced red blood cell counts, haemoglobin, and albumin. The combined extract demonstrated dose-dependent efficacy. Notably, multivariate PCA mapping revealed that the 800 mg/kg dose achieved a superior therapeutic outcome, effectively resolving clinical diarrhoea and restoring biological profiles to states comparable to those of the healthy and Neomycin-treated cohorts. Furthermore, the extract significantly mitigated systemic oxidative stress and protected organ integrity.

Conclusion

These findings demonstrate the therapeutic efficacy and potential of the combined *P. guajava* and *T. diversifolia* extract in managing colibacillosis and associated systemic inflammation in rabbits. This study supports the traditional ethnomedicinal use of these plants and demonstrate that these combined extracts could be develop as natural alternatives to synthetic antibiotics in veterinary medicine for managing colibacillosis in rabbits.

Keywords: *Escherichia coli*, Colibacillosis, Antimicrobial Resistance, *Psidium guajava*, *Tithonia diversifolia*, Rabbits

Introduction

In the face of escalating global climate change and the urgent need for sustainable food systems, rabbit farming (*Oryctolagus cuniculus*) has emerged as an environmentally superior alternative to traditional livestock [1,2]. Characterized by a low carbon footprint, minimal water requirements, and the ability to thrive on non-competitive forage, the domestic rabbit industry is increasingly recognized as a cornerstone of eco-friendly agriculture and a vital contributor to rural economic development and food security worldwide [2]. Global rabbit meat production reached approximately 860,000 tons in 2021, with China leading the sector (53.1%), followed by a growing importance in developing nations [3]~~[3]~~. This prominence of farming is driven by the rabbit's favourable biological characteristics, notably their high fecundity and efficient feed conversion [4]. Furthermore, rabbit meat is a highly nutritious, lean protein source, rich in essential micronutrients such as iron, zinc, and vitamin B12 [5].

Despite this potential, the rabbit farming industry faces substantial health challenges, with enteric diseases representing the most economically devastating hurdle [6]. Infectious

Formatted: Justified, Line spacing: 1.5 lines

Formatted: Justified, Line spacing: 1.5 lines

Formatted: Justified

Formatted: Line spacing: 1.5 lines

Formatted: Justified, Line spacing: 1.5 lines

diarrhoea remains a primary cause of mortality and reduced growth performance, leading to high veterinary costs [7]. Colibacillosis, caused by enteropathogenic *Escherichia coli* (EPEC), is the most prevalent bacterial threat to young rabbits [6]. The pathogenesis of EPEC is defined by a sophisticated cytokine cascade: bacterial attachment (BFP) and effector protein injection (T3SS) trigger an immediate innate response (interleukin-1β and interleukin-6), which subsequently activates a Th1-mediated adaptive immune response [8]. The resulting attaching and effacing (A/E) lesions destroy the intestinal brush border, compromising the epithelial barrier and allowing systemic cytokines to exacerbate fluid loss and diarrhoea [8]. Furthermore, this bacterial colonization triggers an intense oxidative stress response. The host's ability to survive this oxidative storm depends heavily on the mobilization of its antioxidant defences system, particularly superoxide dismutase (SOD) and glutathione peroxidase (GPX) [9] .

The conventional management of colibacillosis has historically relied on a narrow spectrum of antimicrobials, predominantly trimethoprim-sulfamethoxazole and fluoroquinolones [10]. However, the livestock sector is now at a critical juncture due to the global escalation of antimicrobial resistance (AMR). Recent surveillance indicates a dramatic rise in multidrug-resistant (MDR) *E. coli* strains in rabbitries, with some isolates exhibiting total resistance to watch-list antibiotics like tetracyclines and aminoglycosides [11,12]. This phenomenon is not merely a veterinary concern, but a significant public health threat, as the transmission of resistant genes from livestock to the human food chain complicates the treatment of enteric infections in people [13,14]. Furthermore, the increasing regulatory restrictions on antibiotic growth promoters (AGPs) and the growing consumer demand for antibiotic-free animal products have rendered the status quo untenable [15,16]. Consequently, there is an urgent imperative to transition toward sustainable, bio-based therapeutic alternatives that can achieve clinical resolution without contributing to the environmental resistome.

Ethnoveterinary medicine offers a robust reservoir for therapeutic discovery, particularly through species such as *P. guajava*. (Myrtaceae) and *T diversifolia* (Asteraceae). While *P. guajava* is widely used for its tannin-rich antidiarrheal properties [17] and *T. diversifolia* for its anti-inflammatory sesquiterpene lactones [18], their selection for this study was based on the results from our previous investigation. In our preceding ethnopharmacological survey of rabbitries in Cameroon, these two species emerged as the most prominent remedies used by local farmers to manage bacterial enteritis [19]. Subsequent *in vitro* screening of forty-five different preparations revealed that only six extracts, exhibited potent antibacterial activity

against pathogenic *E. coli*. Notably, the combination of *T. diversifolia* and *P. guajava* yielded a synergistic interaction, achieving a Fractional Inhibitory Concentration Index (FICI) as low as 0.265 and reducing individual minimum inhibitory concentration (MIC) values by up to 64-fold [19]. This specific formulation demonstrated a high biological potency index, consistently maintaining activity levels that justify further investigation. Consequently, the present study was designed to validate these synergistic findings *in vivo*, utilizing an integrated multivariate approach to determine if the combination of *P. guajava* and *T. diversifolia* extracts can resolve systemic colibacillosis and restore host homeostasis as effectively as conventional antibiotherapy.

Materials and Methods

Ethical Approval, Ethical Statement, and Animal Welfare

All experimental protocols were reviewed and approved by the Cameroon National Ethical Committee (Ref No. FWIRB00001954). The study was conducted in strict accordance with the ARRIVE guidelines (Animal Research: Reporting of In Vivo Experiments).

Ethnobotanical Selection and Extract Preparation

The selection of plant materials was based on our previous ethnopharmacological survey, identifying active remedies against rabbit bacterial enteritis [19].

Fresh leaves of *P. guajava* (Myrtaceae) and *T. diversifolia* (Hemsl.) A. Gray (Asteraceae) were collected in April 2024 (corresponding to the onset of the rainy season) at approximately 07:00 h (local time) in Yaoundé, Cameroon. The botanical samples were authenticated at the National Herbarium of Cameroon (Voucher Nos. 2884 SRFK and 57410 HNC, respectively). The fresh leaves were air-dried at ambient temperature (25°C) for 14 days until a constant weight was achieved, followed by mechanical pulverization into a fine powder. For *P. guajava*, 2204 g of fresh leaves yielded 500 g of dry powder (fresh-to-dry ratio of approximately 4.4:1). For *T. diversifolia*, 3774 g of fresh leaves yielded 500 g of dry powder (fresh-to-dry ratio of approximately 7.5:1).

The extracts were prepared using cold extraction with distilled water at room temperature to mimic the traditional preparation, ensuring high extraction yield. For each species, 500 g of powder was macerated in 2 L of distilled water for 72 h under continuous agitation. The mixtures were filtered through hydrophilic cotton, and the filtrates were dehydrated under

Formatted: Line spacing: 1.5 lines

Formatted: Justified, Line spacing: 1.5 lines

Formatted: Line spacing: 1.5 lines

Formatted: Justified, Line spacing: 1.5 lines

forced ventilation at 25°C to remove the residual solvent. The resulting crude extracts were stored at -20°C.

Preparation of the combined extract solutions

Combined extract formulations of *P. guajava* and *T. diversifolia* were freshly prepared in distilled water daily. The ratio of the two extracts within the combination (96.975% *P. guajava* and 3.025% *T. diversifolia*) was determined based on the synergistic Fractional Inhibitory Concentration Index (FICI = 0.265) identified in our prior *in vitro* assays [19]. To administer the target doses of 400mg/kg and 800 mg/kg body weight (bw) at a standardized oral gavage volume of 1 mL per 100 g bw (10 mL/kg bw), two distinct stock solutions were prepared. The stock solution of 40 mg/mL (for the 400 mg/kg group) was prepared by dissolving 310.32 mg of *P. guajava* extract and 9.68 mg of *T. diversifolia* extract in 8 mL of sterile distilled water. This delivered 387.9 mg/kg of *P. guajava* and 12.1 mg/kg of *T. diversifolia in vivo*. The second stock solution of 80mg/mL (for the 800mg/kg group) was Prepared by dissolving 620.64 mg of *P. guajava* extract and 19.36 mg of *T. diversifolia* extract in 8mL of sterile distilled water. This delivered 775.8mg/kg of *P. guajava* and 24.2 mg/kg of *T. diversifolia in vivo*.

Characterization and Virulence of the Clinical Isolate

A clinical isolate of *E. coli* was recovered from a rabbit presenting acute colibacillosis symptoms. Identification was performed using the API 20E analytical profile index (bioMérieux, France). Virulence potential was assessed via the Congo red binding assay [20] on tryptic soy agar supplemented with 0.003 % Congo red and 1.5 % bile salts. Red-pigmented colonies, indicative of virulent plasmid expression, were selected and cultured in nutrient broth supplemented with bile salts (0.4%). Inoculum turbidity from a 24-hour culture was adjusted to 4 McFarland, corresponding to a concentration of approximately 10^9 CFU/mL in sterile 0.9 % saline [21].

Experimental Animals

Thirty hybrid rabbits (mixed sex: *three females and two males*, 6–7 weeks old, 800–900 g) were acclimatized for 7 days at the Laboratory of Phytobiochemistry and Medicinal Plant Studies, University of Yaoundé I. Animals were housed in pairs in galvanized cages (35 cm × 40 cm × 60 cm) under a 12-hour light/dark cycle. They were provided ad libitum access to water and a standard pelleted diet (composition: 21 kg corn flour, 4 kg cassava flour, 20 kg

Pennisetum sp., 31 kg wheat bran, 8 kg palm kernel cake, 6 kg soybean cake, 1 kg bone meal, 4 kg palm oil, and 5 kg dried fish). Following acclimatization, rabbits were weighed to establish a baseline, ensuring comparable mean body weights across all experimental groups.

Experimental Design and Induction of Colibacillosis

Formatted: Line spacing: 1.5 lines

To ensure unbiased group allocation, animals were randomly assigned to one of six experimental cohorts (n = 5 per group) using a computer-generated randomization sequence (www.random.org). Throughout the study, the investigators adhered to a strict blinding protocol; all samples and histological slides were labelled with anonymous codes, which were unblinded only after the completion of the final statistical analysis. The experimental groups were designated as follows: T0 (normal control, non-infected, receiving sterile water), T1 (negative control, infected, untreated), T2 (positive control, infected, treated with Neomycin 10 mg/kg p.o. for 5 days), T3 (low dose, infected, 400 mg/kg combined extract p.o.), T4 (high dose, infected, 800 mg/kg combined extract p.o.), and T5 (immunosuppression control, non-infected, receiving 30 mg/kg cyclophosphamide i.p.).

Formatted: Justified, Line spacing: 1.5 lines

To establish a model of acute colibacillosis, rabbits in groups T1 through T4 were pretreated with a combination of cyclophosphamide (30 mg/kg bw, intraperitoneally) and streptomycin (150 mg/kg bw, orally) 48 hours prior to *E. coli* inoculation. This dual-pretreatment protocol is essential in adult lagomorphs, as streptomycin disrupts resident microflora to overcome colonization resistance, while cyclophosphamide induces a transient state of immunosuppression, effectively mimicking the susceptibility of weanling or immunologically stressed rabbits to enteropathogenic *E. coli* colonization. Following these physiological disruptions, colibacillosis was induced in groups T1 through T4 via oral gavage of 1 mL of an *E. coli* suspension (10^9 CFU/mL in 0.9% saline). Therapeutic interventions were administered daily (10 mL/kg bw) for 7 consecutive days, commencing 24 hours post-infection. To ensure uniform experimental conditions and control for handling stress, animals in the T5 group, pretreated with cyclophosphamide (30 mg/kg bw, intraperitoneally) received a daily oral gavage of the vehicle (10 mL/kg bw of sterile distilled water) for the same 7-day duration.

Clinical and Microbiological Monitoring

Formatted: Line spacing: 1.5 lines

Daily faecal samples were collected to quantify bacterial shedding. Stool samples (10 mg) were serially diluted (10^{-5}) in sterile saline and plated on MacConkey agar. After 24 h incubation at 37°C, typical *E. coli* colonies were counted and expressed as Log_{10} CFU/g of stool. Growth performance was monitored by weighing rabbits every two days.

Formatted: Justified, Line spacing: 1.5 lines

Ethical Considerations and Humane Endpoints

A strict clinical monitoring protocol was implemented due to the risk of severe morbidity associated with inducing acute colibacillosis. Rabbits were monitored three times daily (every 8 hours) by for clinical signs of distress. Humane endpoints were strictly defined as: (i) loss of >20% of initial body weight; (ii) severe lethargy or inability to access food/water; (iii) persistent recumbency or lack of righting reflex; (iv) laboured or agonal breathing; and (v) hypothermia or severe dehydration (skin tent >2 seconds).

Any animal meeting these criteria was designated for immediate rescue euthanasia to minimize pain and distress. In this study, only one animal in the T1 (Negative Control) group reached humane endpoints on Day 3 post-infection due to severe dehydration and was euthanized immediately. No animals died spontaneously during the experimental period.

Anaesthesia and Methods of Sacrifice

On Day 7 post-infection (PI), a total of 24 surviving rabbits (n = 4 per group) were humanely sacrificed for sample collection. The remaining five healthy animals from the treatment groups were excluded from terminal sampling and were maintained under standard husbandry conditions for continued post-experimental monitoring. To ensure animal welfare and data integrity, rabbits were moved to a separate, quiet procedure room. Animals were fasted for 12 hours before euthanasia to reduce anaesthesia-related complications. Deep surgical anaesthesia was induced via intramuscular injection of ketamine (30 mg/kg) combined with diazepam (10 mg/kg). The depth of anaesthesia was strictly verified by the complete abolition of the pedal withdrawal and corneal reflexes before any procedures commenced. Euthanasia was completed under deep anaesthesia via cervical dislocation (for rabbits under 1 kg) or exsanguination via cardiac puncture, ensuring a rapid and painless death in accordance with AVMA Guidelines for the Euthanasia of Animals.

Sample Collection, Systemic Antioxidant and Histopathological Analysis

Blood was collected for haematology (EDTA tubes) and serum biochemistry (dry tubes). Haematological parameters included WBC (white blood cells), RBC (red blood cells), HGB (Haemoglobin), HCT (Haematocrit), and leukocyte differentials, analysed using a Mindray BC-5300 automated analyser. Serum biochemistry included ALT (Alanine aminotransferase), AST (aspartate aminotransferase), GGT (Gamma-Glutamyl Transferase), Creatinine, Uric Acid, Total Protein, and lipid profile, including TC (Total Cholesterol), TG (Triglycerides),

and HDL (High-Density Lipoprotein cholesterol), analysed using commercial kits (LABKIT, France). Cytokine levels (TNF-α, IL-1β, IL-6, IL-10, and CRP) were quantified via sandwich ELISA (Quantikine® Rabbit ELISA kits, Sigma-Aldrich).

Tissue samples from the ileum and caecum, liver, and kidney were collected post-mortem for antioxidant assays and histopathological analysis. Ileum and caecum segments were homogenized in 50 mM Tris-HCl buffer (pH 7.4) and centrifuged at 1500 g for 25 min. Supernatants were analysed for MDA (Malondialdehyde) [22], GSH (reduced glutathione) [23], SOD (Superoxide dismutase) [24] and Catalase [25].

For histopathological evaluation, liver, kidney, and caecum sections were fixed in 10% buffered formalin, embedded in paraffin wax. Sections (5 µm thick) were cut using a rotary microtome and stained with haematoxylin and eosin (H&E). Photomicrographs were captured using a digital camera attached to a light microscope. Tissue sections were subjected to a rigorous, independent qualitative descriptive analysis focusing on the presence, distribution, and resolution of classical pathological signs associated with enteric *E. coli* infection, including epithelial desquamation, villous blunting, inflammatory cell infiltration, and vascular congestion. After all sample collection, rabbit carcasses were autoclaved before disposal in compliance with biosafety regulations.

Advanced Statistical and Multivariate Analysis

The data were analysed using OriginLab Pro 2025 and GraphPad Prism version 8.0.1 (GraphPad Software, San Diego, CA, USA). All quantitative data are expressed as mean ±standard deviation (SD). Before hypothesis testing, the normality of data distribution was verified using the Shapiro–Wilk test, and homogeneity of variances was confirmed via Levene's test. Univariate comparisons across the six experimental groups were performed using one-way Analysis of Variance (ANOVA), followed by Tukey's Honest Significant Difference (HSD) post-hoc test to control for multiple comparison inflation and Type I error accumulation. Statistical significance was defined at $P < 0.05$.

Formatted: Justified, Line spacing: 1.5 lines

To evaluate the holistic therapeutic trajectory, an integrated multivariate approach was performed in the RStudio environment (v4.4.3). Principal Component Analysis (PCA) was utilized to reduce dataset dimensionality, with variables auto-scaled (mean-centred and standardized) to account for differing units. We further generated a global biological fingerprint using hierarchical clustering, where heatmaps were constructed from Z-score-normalized data using Euclidean distance and Ward's linkage method to visualize metabolic

and inflammatory shifts across cohorts. A direct comparison between the 800 mg/kg dose and Neomycin (10 mg/kg) was conducted to assess clinical non-inferiority, defined by the absence of significant divergence ($P > 0.05$) in recovery endpoints.

The study sample size (n = 4 per group) was justified by an *a priori* power analysis. Based on historical indices of acute enteric inflammation (anticipating a Cohen's $f \geq 0.70$), this sample size was sufficient to achieve a statistical power $(1 - \beta)$ of 0.80 at $\alpha = 0.05$, consistent with the 3Rs (Reduction) ethical framework. All multivariate analyses and visualizations were performed in the R statistical environment (v4.4.3) using the tidyverse for data manipulation, FactoMineR and factoextra for multivariate modelling, and ggplot2 (ggrepel) for high-resolution, colour-blind friendly graphical representation.

Results

Biochemical identification of *E. coli* isolates and virulence testing

The clinical isolate recovered from symptomatic rabbits was subjected to standardized biochemical characterization using the API 20E system (bioMérieux, Marcy l'Etoile, France). The isolate exhibited characteristic carbohydrate fermentation patterns, including positive reactions for glucose, sorbitol, and arabinose, while remaining negative for urease and H_2S production. The resulting biochemical profile confirmed the identity of the pathogen as *E. coli* with an identification probability of 100% (API 20E profile 5044572; identification quality: very good; probability: 100% for *E. coli* 1), validating its use for the subsequent experimental infection model (Figure 1, Suppl Table 1). The virulence potential of the *E. coli* isolate was phenotypically confirmed via the Congo red binding assay. The isolate exhibited strong dye uptake, resulting in distinct, red-pigmented colonies (CR+) after 24 hours of incubation (Suppl Figure 1). This positive phenotype indicates the presence of virulence-associated surface proteins and curli fimbriae, validating the isolate's capacity for host colonization and its suitability for inducing experimental colibacillosis.

Effects of the combination of *Psidium guava* and *Tithonia diversifolia* extracts on the body weight of experimentally infected rabbits

The effect of *E. coli* infection and subsequent treatment with the combination of *T. diversifolia* and *P. guajava* extracts on the growth trajectory of rabbits is presented in Figure 2 (Suppl Table 2). Following the experimental challenge, a significant divergence in body weight was observed between the study cohorts. The infected, untreated group (T1) exhibited a sharp

Formatted: Line spacing: 1.5 lines

Formatted: Justified, Line spacing: 1.5 lines

Formatted: Line spacing: 1.5 lines

Formatted: Justified, Line spacing: 1.5 lines

and persistent decline in body weight throughout the 7-day post-infection (PI) period. This pathological weight loss is associated with severe malabsorption of nutrient and dehydration from infectious diarrhoea.

In contrast, rabbits treated with the combined *P. guajava* and *T. diversifolia* extracts exhibited a reversal of the infection-induced weight loss. From Day 3 post-infection (PI) onward, the 800 mg/kg dose (T4) promoted body weight gain following a trajectory like that of the healthy control (T0) and Neomycin-treated (T2) groups ($P < 0.05$). However, the 400 mg/kg dose (T3) resulted in moderate weight recovery, indicating a clear dose-dependent therapeutic response. Notably, the 800 mg/kg dose demonstrated efficacy in restoring rabbits to their pre-infection growth curves. These results suggest that the combined extract could be facilitating nutrient assimilation and helping restore osmotic and fluid-solute equilibrium.

Effect of the combination of *Psidium guava* and *Tithonia diversifolia* extracts on the relative weight of some vital organs

Formatted: Line spacing: 1.5 lines

The macroscopic effect of *E. coli* infection and the safety profile of the combined *P. guajava* and *T. diversifolia* extracts treatment were evaluated through relative organ weight analysis and visual inspection (Figure 3). Upon necropsy, all treatment groups exhibited normal organ coloration and morphology, with no visible lesions or congestion in the liver, kidneys, or heart. Statistical analysis revealed that the relative weights of the kidneys and liver in both the 400 and 800 mg/kg extract groups showed no significant variation ($P > 0.05$) when compared to the healthy control (T0) and the Neomycin-treated (T2) groups.

Formatted: Justified, Line spacing: 1.5 lines

Notably, the 800 mg/kg cohort (T4) maintained liver and spleen weights comparable to healthy controls, contrasting with the inflammatory oedema and reactive splenomegaly typically observed in untreated colibacillosis. In the infected-untreated group (T1), increased relative spleen weight suggested a heightened systemic immune response to the bacterial load. Conversely, treatment with the *P. guajava* and *T. diversifolia* combination effectively preserved organ homeostasis. These results suggest that the extract could be providing systemic protection against *E. coli*-induced pathology while maintaining the structural integrity of vital metabolic organs, without inducing adverse toxic effects.

Therapeutic Efficacy and *in vivo* bacterial clearance of the combination of *P. guajava* and *T diversifolia*

Formatted: Line spacing: 1.5 lines

Formatted: Justified, Line spacing: 1.5 lines

The *in vivo* antibacterial potency of the *P. guajava* and *T. diversifolia* combination was assessed by monitoring the temporal kinetic of *E. coli* shedding in the faeces (Figure 4, Suppl Table 3). Bacterial load is a critical indicator of the extract's ability to disrupt intestinal colonization and prevent further mucosal invasion. Following the experimental challenge, all infected groups exhibited peak bacterial shedding (10^9 CFU/10 mg stool) before the initiation of therapy.

Treatment led to a progressive, time-dependent reduction in bacterial shedding across all cohorts. While the reference antibiotic (Neomycin, T2) achieved complete bacterial clearance within 72 hours (Day 3), the combined *P. guajava* and *T. diversifolia* extracts also demonstrated potent curative efficacy. Specifically, both the 400 mg/kg (T3) and 800 mg/kg (T4) doses resulted in no quantifiable bacterial load in faeces by Day 5 of treatment. This 5-day clearance window is clinically significant, as it correlates with the rapid resolution of diarrheic symptoms and the reversal of infection-induced weight loss observed (Figure 3). Collectively, these results indicate that the combined extract exhibits effective, dose-dependent curative activity against *E. coli*, achieving clinical recovery comparable to the reference antibiotic. These findings demonstrate that the *P. guajava* and *T. diversifolia* formulation particularly the 800 mg/kg dose could be a promising biological alternative for the management of clinical colibacillosis.

Formatted: Line spacing: 1.5 lines

The effect of combined aqueous extract treatment on the Antioxidant Defense

Formatted: Justified, Line spacing: 1.5 lines

The impact of *E. coli* infection and subsequent treatment with combined *P. guajava* and *T. diversifolia* extracts on the intestinal redox landscape was evaluated (Figure 5; Suppl Table 4). In the infected-untreated cohort (T1), experimental colibacillosis induced a state of biochemical collapse within both the caecum and ileum. This pathological state was characterized by a significant increase ($P < 0.05$) in malondialdehyde (MDA) levels linked to extensive lipid peroxidation, suggesting the depletion of the primary antioxidant defences, including Superoxide Dismutase (SOD), Catalase (CAT), and reduced Glutathione (GSH). These findings demonstrated that *E. coli* colonization rapidly exhausts the host's local antioxidant protection, leading to oxidative tissue damage.

Therapeutic administration of the *P. guajava* and *T. diversifolia* extracts effectively reversed this oxidative trend in a dose-dependent manner. While the 400 mg/kg dose (T3) provided moderate protection, the 800 mg/kg dose (T4) achieved a superior restorative profile,

returning MDA, CAT, and SOD levels to values statistically similar from those of the healthy control (T0).

Interestingly, in specific parameters such as caecal GSH and ileal SOD, the 800 mg/kg dose of combined *P. guajava* and *T. diversifolia* extracts treatment maintained the antioxidant levels that were numerically superior to the Neomycin standard (T2). This could indicate that while Neomycin serves as a potent bactericidal agent, the combined *P. guajava* and *T.diversifolia* extracts could be providing a dual-action benefit by simultaneously preventing the *E. coli* colonization but also restoring the intestinal antioxidant framework.

The effect of combined aqueous extract treatment on haematological stabilization and resolution of systemic inflammation

Formatted: Line spacing: 1.5 lines

The systemic impact of the *E. coli* challenge and the subsequent restorative effects of the *P. guajava* and *T. diversifolia* combination are summarized in the haematological profiles (Figure 6; Suppl Table 5). The infected-untreated group (T1) exhibited a classic septic hemogram, characterized by profound leucocytosis and thrombocytosis. Specifically, White Blood Cell (WBC) counts nearly doubled and Platelet (PLT) counts nearly tripled compared to the healthy control (T0), signalling a severe systemic inflammatory response and the activation of the host's emergency myeloid defences against *E. coli* septicaemia.

Formatted: Justified, Line spacing: 1.5 lines

Simultaneously, the T1 cohort suffered from anaemia of infection, evidenced by significant declines ($P < 0.05$) in Red Blood Cell (RBC) counts, Haemoglobin (HGB) levels, and Haematocrit (HCT) compared to all other groups. This erythrocytic collapse likely reflects the combined effects of bacterial hemolysins, iron sequestration, and the haemorrhagic nature of the colibacillosis-induced intestinal lesions.

Treatment with the combined *P. guajava* and *T. diversifolia* extracts achieved near-complete normalization of the hemogram in a dose-dependent manner. The 800 mg/kg dose (T4) proved to be particularly effective, successfully restoring the systemic inflammatory response by returning WBC and PLT counts to baseline levels. Furthermore, the bioactive extract reversed the erythrocytic decline, restoring haemoglobin concentrations from a pathological low of approximately 11 g/dL to a healthy 15 g/dL. Notably, the leukocyte differential (GRA%, LYM%, and MID %) was rebalanced in the T4 group, indicating a restored and functional immune landscape comparable to the neomycin-treated (T2) and healthy (T0) cohorts. These findings underscore the extract's potent haematoprotective efficacy and its ability to halt the progression of the infection from a local gut disturbance to a systemic crisis.

Immunomodulatory effects of combined aqueous extract treatment on *E. coli*-infected rabbits

The immunological response to *E. coli* infection and the restorative capacity of the combined *P. guajava* and *T. diversifolia* extracts intervention were evaluated through key pro-inflammatory and anti-inflammatory biomarkers (Figure 7; Suppl Table 6). In the infected-untreated cohort (T1), experimental colibacillosis triggered a massive cytokine production, evidenced by significant elevations ($P < 0.05$) in C-reactive protein (CRP), TNF-α, IL-1β, and IL-6. This excessive release of innate first-responder cytokines drives the systemic inflammatory response, leading to tissue damage and metabolic exhaustion observed in the T1 group.

Oral administration of the *P. guajava* and *T. diversifolia* combination at 400 and 800 mg/kg induced a significant reduction in these pro-inflammatory markers. Notably, for critical indicators like TNF-α and IL-6, the 800 mg/kg dose (T4) was more potent, suppressing cytokine levels as effectively as the Neomycin standard (T2). This suggests that the bioactive metabolites in the extract, such as flavonoids and sesquiterpene lactones, likely modulate the underlying signalling pathways (such as NF-kB) responsible for acute inflammatory cascades.

Conversely, the anti-inflammatory cytokine IL-10 showed a distinct and clinically relevant pattern. While IL-10 levels were significantly lower in the untreated group (T1), all treated cohorts (T2, T3, T4) maintained significantly higher concentrations of this cytokine ($P < 0.05$). This could be an indication that the combined *P. guajava* and *T. diversifolia* extract does not merely suppress the immune system; rather, it facilitates immunological rebalancing by promoting an anti-inflammatory environment that accelerates tissue healing and the resolution of diarrhoea. The normalization of CRP, a standard marker of acute systemic inflammation in rabbits, further validates that the T4 dose achieved complete systemic resolution of the infection**.**

Effects of combined aqueous extract treatment on Hepato-Renal Protection and Metabolic Homeostasis in *E. coli*-infected rabbits

The systemic metabolic profile and organ-functional integrity of the experimental rabbits are summarized in Figure 8 (Suppl Table 7). Induction of colibacillosis in the untreated cohort (T1) resulted in significant biochemical derangement ($P < 0.05$), characterized by a dyslipidaemia of infection and acute hepato-renal distress. Specifically, T1 rabbits exhibited an increase in

Formatted: Line spacing: 1.5 lines

Formatted: Justified, Line spacing: 1.5 lines

Formatted: Line spacing: 1.5 lines

Formatted: Justified, Line spacing: 1.5 lines

serum transaminases (ASAT, ALAT) and GGT, signalling acute hepatocellular injury and membrane leakage induced by bacterial endotoxins.

Renal function was similarly compromised in the untreated group, with Creatinine and Urate levels nearly doubling. This elevation may indicate a reduced Glomerular Filtration Rate (GFR), likely secondary to infection-induced dehydration and septic renal injury. Furthermore, a state of hypoproteinaemia and hypoalbuminemia was observed in T1, reflecting the systemic inflammatory response and potential protein-losing enteropathy associated with severe diarrhoea.

Therapeutic intervention with the *P. guajava* and *T. diversifolia* extracts effectively shielded the primary metabolic organs from this endotoxicity. The 800 mg/kg dose (T4) achieved a metabolic reset, returning ASAT, GGT, Creatinine, and Urate to baseline levels that were statistically indistinguishable from those of the healthy controls (T0). Notably, the extract restored the lipid profile by lowering triglycerides and LDL-cholesterol while significantly increasing HDL-cholesterol ($P < 0.05$) compared to the untreated group. By stabilizing hepatocellular membranes and maintaining renal blood flow, the 800 mg/kg dose showed organ-protective effects. This helped restore systemic balance comparable to the neomycin-treated group (T2).

Integrated Hierarchical Clustering: The Global Biological Fingerprint

Hierarchical clustering analysis was performed on the integrated biological dataset to visualizes the holistic therapeutic trajectory of the rabbits (Figure 9). The resulting heatmap successfully discriminated between the various experimental cohorts, establishing a clear molecular signature for both the pathological state and the subsequent recovery. The infected-untreated cohort (T1) formed a distinct cluster characterized by a massive upregulation of pro-inflammatory and damage-associated markers, specifically manifesting as elevated ASAT, creatinine, MDA, and pro-inflammatory cytokines (TNF-α, IL-6).

In stark contrast, the cohort treated with 800 mg/kg of combined *P.guajava* and *T. diversifolia* extracts (T4) exhibited a reversal of this fingerprint. The T4 group clustered closely with the healthy control (T0) and the neomycin-treated (T2) groups, displaying a synchronized restoration of antioxidant enzymes (SOD, CAT, GSH) and a normalization of systemic biochemical indices. This global transition from the oxidative-inflammatory cluster to the homeostatic cluster underscores the multifaceted curative potential of the *P. guajava* and *T. diversifolia* combination. These results could be suggesting that the bioactive extract does not

Formatted: Line spacing: 1.5 lines

Formatted: Justified, Line spacing: 1.5 lines

merely target isolated symptoms but induces a systemic homeostatic restoration, restoring the animal's physiological state to its pre-infection baseline.

Multivariate Trajectory and Key Biological Drivers

Formatted: Line spacing: 1.5 lines

Formatted: Justified, Line spacing: 1.5 lines

Principal Component Analysis (PCA) was applied to the combined dataset as an exploratory tool to visualize the overall multidimensional relationships and clustering trends across the experimental cohorts (Figure 10A). The unsupervised PCA model revealed a visible separation among groups, with the first two dimensions capturing a cumulative total of 57.7% of the total variance. Principal Component 1 (Dim1), which accounted for 48.0% of the variance, served as the primary axis of separation, segregating the coordinates of the infected-untreated cohort (T1) from the clustering space of the healthy controls (T0). Notably, the 800 mg/kg cohort (T4) exhibited a spatial shift along Dim1, positioning itself in closer proximity to the multivariate space occupied by the T0 and Neomycin (T2) groups. This spatial alignment suggests an exploratory trend wherein the global physiological profile of the high-dose extract cohort reflects an orientation toward the healthy and reference treatment spaces. However, given the exploratory nature of this analysis and the lack of an independent validation dataset, these spatial configurations represent descriptive clustering behaviours rather than definitive proof of systemic homeostatic equivalence.

The Variable Contribution analysis (Figure 10B) further highlighted the primary variables associated with the spatial distribution across the dimensions. The clustering trends observed in the PCA map were heavily influenced by coordinates related to bacterial clearance kinetics (load at Days 10, 12, and 13) and systemic inflammatory markers (WBC and CRP). While these variables exhibited the highest contribution scores along Dim1, these mathematical relationships remain exploratory in nature. In small-cohort designs, individual biological variation can heavily influence variable contribution weights. Thus, these parameters should be viewed as hypothesis-generating indicators, pointing to pathogen eradication and the subsequent quenching of the systemic leukocyte response as key therapeutic pathways of the extract rather than validated predictive biomarkers.

Comparative Functional Efficacy and Therapeutic Parity

Formatted: Line spacing: 1.5 lines

Formatted: Justified, Line spacing: 1.5 lines

A comparative functional analysis was conducted between the high-dose extract (800 mg/kg) and the Neomycin standard to evaluate the clinical viability of the combination of the *T. diversifolia and P. guajava* intervention as a sustainable alternative to conventional therapy (Figure 11).

The restorative capacity of the *P. guajava* and *T. diversifolia* combination was found to be functionally equivalent to Neomycin across all oxidative stress indices. In both the caecum and ileum, the 800 mg/kg cohort exhibited a robust recovery of Superoxide Dismutase (SOD) and Catalase (CAT) activities, with mean values statistically indistinguishable from the antibiotic-treated group ($P > 0.05$).

In terms of immunomodulation, the extract achieved complete systemic stabilization. The suppression of C-reactive protein (CRP), a sensitive marker of acute-phase inflammation, in rabbits, and the normalization of White Blood Cell (WBC) counts showed no significant divergence between the combined extract of *P. guajava and T. diversifolia* and Neomycin cohorts. This suggests that the extract possesses a systemic anti-inflammatory mechanism comparable to the resolution achieved through bacterial clearance by synthetic antimicrobials.

By resolving the *E. coli* challenge through both direct antimicrobial action and active tissue repair, the 800 mg/kg dose demonstrated therapeutic efficacy comparable to that of Neomycin. These findings suggest that this combined *P. guajava* and *T. diversifolia* extracts could be a potent bioactive agent, an alternative to conventional antibiotic that can maintain rabbit health and productivity.

Histopathology results

Formatted: Line spacing: 1.5 lines

Effect of combined extract on the renal architecture

The protective effect of the *P. guajava* and *T. diversifolia* combination on the renal microarchitecture of *E. coli*-infected rabbits is presented in Figure 12. Microscopic examination of the kidney sections from the healthy control group (T0) revealed a well-preserved renal parenchyma. This was characterized by a distinct glomerular architecture (G) with a clear Bowman's space (US) and clearly differentiated proximal (PT) and distal (DT) convoluted tubules, indicating normal physiological filtration and reabsorption functions.

Formatted: Justified, Line spacing: 1.5 lines

In contrast, the infected-untreated cohort (T1) exhibited significant histopathological derangement. The infection induced a localized inflammatory response marked by conspicuous leukocyte infiltration (IL) and mesangial expansion within the glomerulus. These alterations are indicative of acute renal distress and the systemic inflammatory burden caused by the pathogenic *E. coli* strain. However, therapeutic intervention with the combined *P. guajava* and *T. diversifolia* extracts effectively mitigated these structural damages. The renal

sections from the T3 (400 mg/kg) and T4 (800 mg/kg) groups, alongside the Neomycin-treated cohort (T2), demonstrated a restoration of the renal tissue architecture. While the 800 mg/kg dose (T4) showed minor residual leukocyte infiltrates, the overall prevention of mesangial expansion and tubular disruption confirms the extract's nephroprotective potential. This structural preservation is consistent with the biochemical results, where the extract successfully normalized serum creatinine and uric acid levels, confirming that the formulation prevents the acute kidney injury (AKI) typically associated with severe colibacillosis.

Protective Effect of Extracts on Hepatic Microarchitecture

Formatted: Line spacing: 1.5 lines

Formatted: Justified, Line spacing: 1.5 lines

The histological evaluation of the liver (Figure 13) confirms the organoprotective properties of the combination of *P. guajava* and *T. diversifolia* during acute *E. coli* infection. Microscopic analysis of the liver from the healthy control group (T0) showed a classical hexagonal lobular arrangement with a well-preserved hepatic parenchyma. The sections exhibited a distinct portal triad comprising the hepatic portal vein (PV), hepatic artery (HA), and bile canaliculus (BC) surrounded by radiating cords of well-defined, polyhedral hepatocytes (He) with prominent nuclei.

In the infected-untreated cohort (T1), the hepatic architecture was compromised by a localized inflammatory response, characterized by significant leukocyte infiltration (IL). This infiltration suggests that the systemic *E. coli* infection induced a degree of hepatitis or endotoxin-mediated tissue stress. However, administration of the combined *P. guajava* and *T. diversifolia* extracts formulation at both 400 mg/kg (T3) and 800 mg/kg (T4) successfully mitigated these inflammatory changes. The hepatic sections of the treated groups showed a restoration of the hepatocellular cords and a marked absence of inflammatory clusters, appearing histologically comparable to the Neomycin-treated (T2) and healthy control (T0) groups.

This structural preservation directly correlates with the enzymatic profiles discussed earlier; by preventing the infiltration of inflammatory cells and maintaining the integrity of the hepatocellular membranes, the extract effectively prevented the leakage of transaminases (ASAT and ALAT) into the systemic circulation. These findings reinforce the dual role of the combination of *P. guajava* and *T. diversifolia* as both an antimicrobial and a potent hepatoprotective agent.

Restorative Effects of extracts on Intestinal (Caecal) Microarchitecture

Formatted: Line spacing: 1.5 lines

The impact of the *E. coli* challenge and the subsequent therapeutic intervention on the caecal microarchitecture is presented in Figure 14. Histological analysis of the caecal sections from healthy control rabbits (T0) revealed a robust and well-organized intestinal wall. The architecture followed a classic structural hierarchy, consisting of the serosa (Se), muscular layer (Mus), submucosa (Smu), and a well-developed mucosa (Mu) lining the intestinal lumen (Lu).

Formatted: Justified, Line spacing: 1.5 lines

In the infected-untreated cohort (T1), the *E. coli* challenge induced severe mucosal degeneration. The sections exhibited a distinct narrowing of the intestinal mucosa and marked villous atrophy. This loss of structural height and complexity in the mucosal layer indicates a depletion of goblet cell function (leading to decreased mucus secretion) and a reduction in the total absorptive surface area. Such morphological damage provides a structural explanation for the persistent diarrhoea and negative growth performance observed in structurally comparable to the healthy controls and the Neomycin-treated cohort (T2). By maintaining the mucosal barrier and preventing villous blunting, the high-dose extract (800 mg/kg) preserved the intestinal function, facilitating nutrient absorption and preventing the translocation of enteric pathogens. This structural recovery serves as the biological foundation for the observed resolution of clinical diarrhoea and the restoration of the animals' growth trajectory.

Discussion

Formatted: Line spacing: 1.5 lines

Even though rabbit meat continues to be considered a niche product, demand for it is increasing steadily in various parts of the world, particularly in Asia [26]. Beyond this expanding market, rabbit farming also offers advantages in social equity [27] and environmental sustainability [2]. Despite this potential, the industry, especially in developing nations, is severely constrained by enteric pathologies, most notably colibacillosis caused by pathogenic strains of *E. coli*. This disease compromises animal health and productivity through high morbidity and mortality, and poses a critical One Health risk because the transmission of multidrug-resistant (MDR) strains through the food chain represents a growing public health threat [28,29]. As the efficacy of conventional antimicrobials wanes with the global rise of antimicrobial resistance (AMR), the exploration of plant-based therapeutic alternatives has become a pharmacological necessity. Our previous ethnopharmacological screening of Cameroonian flora identified *P. guajava* and *T. diversifolia* as potent inhibitors of pathogenic *E. coli*, with their combination exhibiting a synergistic reduction in Minimum Inhibitory Concentration of up to 64-fold [19]. Building on that in vitro evidence, the present study evaluated the *in vivo* curative potential and systemic

Formatted: Justified, Line spacing: 1.5 lines

restorative effects of the combined *P. guajava* and *T. diversifolia* extract in *E. coli*-infected rabbits.

The therapeutic efficacy of the combined extract was assessed in colibacillosis-infected rabbits, following the induction protocol of Pouofo et al.[30]~~Nguim et al. (2021)~~. The treatment was conducted for 7 days, during which the bacterial shedding was used to monitor the treatment. By the end of the treatment period, the various parameters evaluated to determine the extract's therapeutic efficacy, including body weight, relative organ weight, bacterial shedding, haematological, biochemical, inflammatory, and oxidative markers, as well as histopathological findings, showed comparable values between T5 (immunosuppression group) and the healthy control group T0 (uninfected, untreated, and free of cyclophosphamide/streptomycin). This finding indicates that the immunosuppressive effect of cyclophosphamide was reversible and had no measurable lasting impact under our experimental conditions. Therefore, the variation observed in the infected groups could be attributed to the infection and/or extract treatment rather than to pretreatment toxicity.

The experimental induction of colibacillosis produced a profound clinical decline in the untreated cohort, characterized by acute weight loss, dehydration, and high-titer fecal shedding of Congo red–positive *E. coli*. This pathological state is consistent with the capacity of pathogenic *E. coli* to disrupt intestinal water transport and induce catabolic stress [31]. The reversal of these symptoms following administration of the combined extract, particularly at 800 mg/kg demonstrates clear therapeutic efficacy. Bacterial clearance by Day 5 of treatment by combined extract is notable and comparable to the fast-killing activity observed with Neomycin. This bacterial clearance is consistent with previously observed antimicrobial activity of these extracts and their combination- [19,32].

Our LC-MS/MS profiling of the two extracts identified pedunculagin, an ellagitannin, in *P. guajava*, together with the flavonoid's quercetin, quercetin-4′-O-glucoside, myricetin-3-O-pentoside, and 2′-hydroxy-α-naphthoflavone in *T. diversifolia* (Suppl. Table 8). In fact, tannins and flavonoids of *P. guajava* have been reported to interact with bacterial cell-wall proteins and inhibit *E. coli* growth. Dhiman et al. [33], showed that the methanolic extract *P. guajava* leaf exhibited antibacterial activity against *E. coli* with a minimum inhibitory concentration of 0.78 µg/ml and minimum bactericidal concentration of 50 µg/ml and the phytochemical screening revealed flavonoids, steroids, and tannins as the likely antimicrobial contributors. However, Pereira et al. [34] also found that an *aqueous P. guajava* leaf extract, contain flavonoid and tannin classes (rutin, hesperidin, quercetin). Recently, El-Deeb et al.

[30][32] conducted a comprehensive assessment of *P. cattleianum* aerial-part polyphenols and found that, beyond producing significant antidiarrheal effects in a castor oil-induced diarrhoea model, the extract also showed direct activity against several diarrhea-associated pathogens, with *in silico* docking implicating the bacterial fatty-acid synthesis enzyme FabH and the kappa opioid receptor as plausible molecular targets.– For *T. diversifolia*, an independent chemical characterization of an *aqueous* leaf extract by Dongmo et al. [35] corroborates the presence of tannins and flavonoids alongside phenols, alkaloids, terpenoids, and saponins, consistent with our own LC-MS/MS annotation.

The septic hemogram observed in the untreated group, marked by leucocytosis, thrombocytosis, and secondary anaemia, reflects the systemic inflammatory burden of colibacillosis, consistent with findings reported by Petrovou et al. [36][35] in both spontaneous and experimental colibacillosis in weaned rabbits. The normalization of these parameters in all treated groups (T2–T4) indicates resolution of systemic stress. The extract lowered circulating TNF-α, IL-6, and IL-1β and returned CRP to near-normal levels, consistent with an immunomodulatory rather than a purely immunosuppressive action. Phytochemicals from both plant sources may plausibly contribute to this effect. For *P. guajava*, the strongest supporting evidence comes from a recent study in carrageenan-induced inflammation in Wistar rats, which found that ethanolic *P. guajava* bark extract significantly reduced TNF-α, IL-6, and CRP relative to untreated controls, with molecular docking indicating binding of extract constituents to inflammatory target proteins [37]. For *T. diversifolia*, Broering et al. [38] [38] reported that a *T. diversifolia* extract reduced secretion of TNF, IL-1β, IL-6, and nitrite, and inhibited neutrophil chemotaxis and CD18 expression in carrageenan-induced inflammation models. However, specific mechanisms such as NF-κB inhibition, direct cytokine receptor modulation, ROS scavenging, membrane stabilization, or inhibition of bacterial adhesion were not directly evaluated in the present study, therefore remain as plausible literature-supported hypotheses rather than demonstrated mechanisms. Unlike antibiotics, which primarily target the pathogen, this combined extract of *P. guajava* and *T. diversifolia* actively manages the host's immune response, preventing the tissue-damaging effects of chronic inflammation.

The hypoproteinaemia and hypoalbuminemia observed in Group T1 are consistent with a negative acute-phase response, in which the liver shifts protein synthesis toward inflammatory mediators such as CRP at the expense of albumin [39], a pattern previously reported in *E. coli* infection [40,41]. The concurrent rise in ASAT and GGT points to hepatocellular leakage,

Formatted: Justified, Line spacing: 1.5 lines

while elevated uric acid suggests impaired renal clearance or accelerated purine catabolism secondary to tissue damage. The extract's ability to restore these metabolic markers, corroborated by our histological findings of preserved portal triad architecture and tubular integrity, is consistent with a hepatoprotective and nephroprotective effect [42].

At the primary site of infection, colibacillosis-induced oxidative stress (elevated MDA, depleted SOD/CAT) plausibly drove the mucosal damage observed histologically, with villous atrophy and mucosal narrowing providing a physical basis for the malabsorption seen in untreated animals. The tannins and flavonoids identified in both extracts are consistent with a protective, astringent mechanism in which these compounds could coat and stabilize the mucosal surface, limiting further epithelial damage. That could plausibly contribute to the restored villous height and mucosal thickness observed in treated groups [43]This mechanism was not directly tested in the present study and remains a hypothesis consistent with the compositional and histological findings.

The multivariate analyses (PCA and hierarchical clustering) show that, within this exploratory dataset, the 800 mg/kg dose cohort clusters closer to the healthy and Neomycin-treated groups than to the untreated group. Given the small per-group sample size ($n = 4$) relative to the number of variables analysed, and the absence of an independent validation cohort, these multivariate results should be interpreted as purely descriptive. Overall, the combined extract shows antimicrobial, immunomodulatory, antioxidant, and organ-protective effects comparable to Neomycin at 800 mg/kg, supporting further mechanistic and pharmacokinetic investigation as a candidate alternative for colibacillosis management.

Limitations of the Study

Formatted: Line spacing: 1.5 lines

Formatted: Justified, Line spacing: 1.5 lines

The chemically induced infection model (cyclophosphamide + streptomycin) is a manipulated, opportunistic model rather than a natural infection; the T5 control indicates pretreatment effects had resolved by the study endpoint, but early interactions between immunosuppression and infection remain a confounding variable that non-chemical, age-susceptible models could better address. Second, the small sample size ($n = 4$–5/group), while adequately powered for the large primary effects observed (a priori Cohen's $f \geq 0.70$, power = 0.80), limits detection of subtler effects and may not capture the full range of biological variability in outbred rabbits. Third, histopathological evaluation was qualitative, without semi-quantitative lesion scoring or morphometric measurement (villus height, crypt depth), due to equipment constraints; the polarized group differences were visually clear but not

numerically graded. Fourth, PCA/heatmap analyses are exploratory given the sample-to-variable ratio and lack an independent validation cohort. Finally, mechanistic claims regarding NF-κB inhibition, receptor binding, membrane disruption, or adhesion inhibition are hypotheses drawn from phenotypic and phytochemical data, not directly tested pathways; future work (bioactivity-guided fractionation, adhesion assays, pharmacokinetics, dedicated toxicology, and non-chemical infection models) is needed to confirm them.

Conclusion

Formatted: Line spacing: 1.5 lines

Formatted: Justified, Line spacing: 1.5 lines

The present study demonstrates the *in vivo* efficacy of the combined *P. guajava* and *T. diversifolia* extracts in mitigating *E. coli* infection in a rabbit model. In infected, immunocompromised rabbits, the combined extract at 800 mg/kg was associated with resolution of the clinical signs of colibacillosis, including restored body-weight gain and a reduction in faecal bacterial shedding to near-undetectable levels, alongside repair of infection-induced tissue damage and no overt signs of toxicity within the parameters assessed in this study. Treated animals showed biochemical, haematological, and antioxidant profiles approaching those of healthy controls, though consistent with the exploratory nature of our multivariate analysis this should be read as a marked improvement toward baseline rather than confirmed physiological equivalence. These findings indicate that the combined *P. guajava*–*T. diversifolia* extract is a promising candidate for managing colibacillosis in rabbits, warranting further development. By addressing both the pathogen and the host's systemic response, this combination represents a potentially sustainable approach to colibacillosis control amid rising antimicrobial resistance, consistent with One Health-aligned veterinary practice.

Before translational application, several steps remain. Phytochemical fingerprinting and standardization of the formulation are needed to support pharmacological interpretation and reproducibility. Evaluation against a broader panel of multidrug-resistant strains would clarify the extract's relevance to other enteric infections, and 16S rRNA metagenomic profiling would clarify its impact on commensal intestinal microbiota relative to conventional aminoglycosides. Critically, dedicated pharmacokinetic, dose-optimization, and long-term toxicology studies, together with larger confirmatory trials, are required before the clinical or field-level viability of this formulation can be established.

Supporting information

Formatted: Line spacing: 1.5 lines

Data is provided within the supplementary information files.

Author contributions

Data curation, Formal analysis, Investigation, Methodology, Validation, Writing – original draft: Mireille Tuedom Chouegouong

Conceptualization, Data curation, Software, Formal analysis, Project administration, Resources, Supervision, Validation, Visualization, Writing – review & editing: Rufin Marie Kouipou Toghueo

Investigation, Methodology and Data analysis: Yanick Kevin Melogmo Dongmo[1],

Investigation, Methodology and Data analysis: Jaurès Marius Tsakem Nangap,

Investigation, Methodology and Data analysis: Michele Stella Majoumouo

Investigation, Methodology and Data analysis: Brice Pinlap

Conceptualization, Resources, Supervision: Fabrice Fekam Boyom and Kingsley Agbor Etchu

All the authors have read and approved the final manuscript.

Formatted: Justified

Competing interests

The authors declare no competing interests.

References

1. Bordignon F, Xiccato G, Trocino A, Zuffellato A, Castellini C, Mattioli S, et al. Environmental impact of rabbit production systems: a farm-based cradle-to-gate analysis. animal. 2025;19: 101488. doi:10.1016/j.animal.2025.101488

2. Cesari V, Zucali M, Bava L, Gislon G, Tamburini A, Toschi I. Environmental impact of rabbit meat: The effect of production efficiency. Meat Science. 2018;145: 447–454. doi:10.1016/j.meatsci.2018.07.011

3. European Commission. Directorate General for Health and Food Safety. Commercial rabbit farming in the European Union: overview report. LU: Publications Office; 2017. doi:10.2772/62174

4. Mancini S, Paci G. Probiotics in Rabbit Farming: Growth Performance, Health Status, and Meat Quality. Animals. 2021;11: 3388. doi:10.3390/ani11123388

5. Siddiqui SA, Gerini F, Ikram A, Saeed F, Feng X, Chen Y. Rabbit Meat—Production, Consumption and Consumers' Attitudes and Behavior. Sustainability. 2023;15: 2008. doi:10.3390/su15032008

6. Solans L, Arnal JL, Sanz C, Benito A, Chacón G, Alzuguren O, et al. Rabbit Enteropathies on Commercial Farms in the Iberian Peninsula: Etiological Agents Identified in 2018–2019. Animals. 2019;9: 1142. doi:10.3390/ani9121142

7. Martignon M, Burel C, Licois D, Reperant E, Postollec G, Valat C, et al. Challenging the growing rabbit with a moderately pathogenic E. coli under ad libitum or limited feed intake conditions: impact on digestive physiology, bacterial communities, and on post-weaning growth. World Rabbit Sci. 2021;29: 1. doi:10.4995/wrs.2021.14089

Formatted: French (France)

Formatted: French (France)

8. Long TM, Nisa S, Donnenberg MS, Hassel BA. Enteropathogenic Escherichia coli Inhibits Type I Interferon- and RNase L-Mediated Host Defense To Disrupt Intestinal Epithelial Cell Barrier Function. Bäumler AJ, editor. Infect Immun. 2014;82: 2802–2814. doi:10.1128/IAI.00105-14

9. Farooq MZ, Wang X, Yan X. Effects of Aeriscardovia aeriphila on growth performance, antioxidant functions, immune responses, and gut microbiota in broiler chickens. J Zhejiang Univ Sci B. 2023;24: 1014–1026. doi:10.1631/jzus.B2200621

10. Kerek Á, Szabó Á, Barnácz F, Csirmaz B, Kovács L, Jerzsele Á. Antimicrobial Resistance in Commensal Bacteria from Large-Scale Chicken Flocks in the Dél-Alföld Region of Hungary. Veterinary Sciences. 2025;12: 691. doi:10.3390/vetsci12080691

11. Silva A, Silva V, Tavares T, López M, Rojo-Bezares B, Pereira JE, et al. Rabbits as a Reservoir of Multidrug-Resistant Escherichia coli: Clonal Lineages and Public Health Impact. Antibiotics. 2024;13: 376. doi:10.3390/antibiotics13040376

12. Sun C, Wang Z, Li Y, Huang J. Antibiotic resistance spectrums of Escherichia coli and Enterococcus spp. strains against commonly used antimicrobials from commercial meat-rabbit farms in Chengdu City, Southwest China. Front Vet Sci. 2024;11: 1369655. doi:10.3389/fvets.2024.1369655

13. Elbehiry A, Marzouk E. From Farm to Fork: Antimicrobial-Resistant Bacterial Pathogens in Livestock Production and the Food Chain. Veterinary Sciences. 2025;12: 862. doi:10.3390/vetsci12090862

14. Pandey S, Doo H, Keum GB, Kim ES, Kwak J, Ryu S, et al. Antibiotic resistance in livestock, environment and humans: One Health perspective. J Anim Sci Technol. 2024;66: 266–278. doi:10.5187/jast.2023.e129

15. Wickramasuriya SS, Ault J, Ritchie S, Gay CG, Lillehoj HS. Alternatives to antibiotic growth promoters for poultry: a bibliometric analysis of the research journals. Poultry Science. 2024;103: 103987. doi:10.1016/j.psj.2024.103987

16. Zheng S, Li Y, Chen C, Wang N, Yang F. Solutions to the Dilemma of Antibiotics Use in Livestock and Poultry Farming: Regulation Policy and Alternatives. Toxics. 2025;13: 348. doi:10.3390/toxics13050348

17. Liu J, Han X, Zhang T, Tian K, Li Z, Luo F. Reactive oxygen species (ROS) scavenging biomaterials for anti-inflammatory diseases: from mechanism to therapy. J Hematol Oncol. 2023;16: 116. doi:10.1186/s13045-023-01512-7

18. Abe AE, De Oliveira CE, Dalboni TM, Chagas-Paula DA, Rocha BA, De Oliveira RB, et al. Anti-inflammatory sesquiterpene lactones from Tithonia diversifolia trigger different effects on human neutrophils. Revista Brasileira de Farmacognosia. 2015;25: 111–116. doi:10.1016/j.bjp.2015.01.005

19. Chouegouong MT, Majoumouo MS, Menkem EZ, Yimgang LV, Toghueo RMK, Etchu KA, et al. Ethnopharmacological survey and antibacterial activity of medicinal plant extracts used against bacterial enteritis in rabbits. ADV TRADIT MED (ADTM). 2023;23: 213–223. doi:10.1007/s13596-021-00615-1

20. Yadav V. Congo red binding and Plasmid Profile of E. coli Isolates of Poultry Origin. J Anim Health Prod. 2014;2: 31–32. doi:10.14737/journal.jahp/2014/2.3.31.32

Formatted: French (France)

Formatted: French (France)

21. Yousif AA. Effects of E.coli O157:H7 Experimental Infections on Rabbits: Donia Hatem Fadhel al-Taii and Afaf Abdulrahman Yousif. Iraqi J Vet Med. 2019;43: 34–42. doi:10.30539/iraqijvm.v43i1.468

22. Wilbur KM, Bernheim F, Shapiro OW. Determination of lipid peroxidation. Archives of Biochemistry and Biophysics. 1949;24: 305–310.

23. Ellman GL. Tissue sulfhydryl groups. Archives of biochemistry and biophysics. 1959;82: 70–77.

24. Misra HP, Fridovich I. The role of superoxide anion in the autoxidation of epinephrine and a simple assay for superoxide dismutase. Journal of Biological chemistry. 1972;247: 3170–3175.

25. Sinha AK. Colorimetric assay of catalase. Analytical biochemistry. 1972;47: 389–394.

26. European Commission. Directorate General for Health and Food Safety. Commercial rabbit farming in the European Union: overview report. LU: Publications Office; 2017. Available: https://data.europa.eu/doi/10.2772/62174

27. Leroy F, Petracci M. Rabbit meat: valuable nutrition or too-cute-to-eat? World Rabbit Sci. 2021;29: 239–246. doi:10.4995/wrs.2021.12663

28. Mahmoudi P, Rashidi A, Idrees Hama Faraj S. Network meta-analysis of the prevalence and antimicrobial resistance of Escherichia coli isolated from bovine milk and dairy products: A global perspective. Journal of Dairy Science. 2025;108: 9330–9344. doi:10.3168/jds.2024-26197

29. Risalvato J, Sewid AH, Eda S, Gerhold RW, Wu JJ. Strategic Detection of Escherichia coli in the Poultry Industry: Food Safety Challenges, One Health Approaches, and Advances in Biosensor Technologies. Biosensors. 2025;15: 419. doi:10.3390/bios15070419

30. Pouofo Nguiam M, Njonte Wouamba SC, Longo F, Gounoue Kamkumo R, Donji Kenne Foweda L, Dzeufiet Djomeni PD, et al. Antibacterial and antishigellosis activity of Xylopia staudtii (engl. & diels), Annonaceae. Journal of Ethnopharmacology. 2021;280: 114406. doi:10.1016/j.jep.2021.114406

31. Swennes AG, Buckley EM, Madden CM, Byrd CP, Donocoff RS, Rodriguez L, et al. Enteropathogenic Escherichia coli prevalence in laboratory rabbits. Vet Microbiol. 2013;163. doi:10.1016/j.vetmic.2013.01.006

32. El-Deeb EM, Elsayed HE, Khazaal MT, El-Sayed EK, Ahmed AA, Mansour YE, et al. Comprehensive assessment of the antidiarrheal properties of polyphenols from Psidium cattleianum Sabine’s aerial parts. Journal of Ethnopharmacology. 2025;346: 119670.

33. Dhiman A, Nanda A, Ahmad S, Narasimhan B. In vitro antimicrobial activity of methanolic leaf extract of Psidium guajava L. Journal of Pharmacy and Bioallied Sciences. 2011;3: 226–229. doi:10.4103/0975-7406.80776

34. Pereira GA, Chaves DSDA, Silva TME, Motta REDA, Silva ABRD, Patricio TCDC, et al. Antimicrobial Activity of Psidium guajava Aqueous Extract against Sensitive and Resistant Bacterial Strains. Microorganisms. 2023;11: 1784. doi:10.3390/microorganisms11071784

35. Dongmo AN, Nguefack J, Dongmo JBL, Fouelefack FR, Azah RU, Nkengfack EA, et al. Chemical characterization of an aqueous extract and the essential oil of Tithonia diversifolia and their

Formatted: French (France)

biocontrol activity against seed-borne pathogens of rice. J Plant Dis Prot. 2021;128: 703–713. doi:10.1007/s41348-021-00439-w

36. Petrov V, Lyutskanov M, Kanakov D. Effects of spontaneous and experimental colibacteriosis on some haematological and blood biochemical parameters in weaned rabbits. Bulgarian Journal of Veterinary Medicine. 2011;14.

37. Anorue EC, Ogara AL, Joshua PE, Ibekwe DG, Igbokwe AI. Impact of Psidium guajava bark extract on inflammatory biomarkers in Wistar rat models of inflammation. Inflammopharmacology. 2025; 1–27.

38. Broering MF, Nunes R, De Faveri R, De Faveri A, Melato J, Correa TP, et al. Effects of Tithonia diversifolia (Asteraceae) extract on innate inflammatory responses. Journal of ethnopharmacology. 2019;242: 112041.

39. Zhou H-H, Tang Y-L, Xu T-H, Cheng B. C-reactive protein: structure, function, regulation, and role in clinical diseases. Front Immunol. 2024;15: 1425168. doi:10.3389/fimmu.2024.1425168

40. Georgieva TM, Georgiev IP, Iliev Y, Petrov VS, Vachkov A, Kanelov I, et al. Blood serum concentrations of total proteins and main protein fractions in weaning rabbits experimentally infected with E. coli. Rev Med Vet. 2008;159: 431–436.

41. ALi G IE, A.Y. T, Naena N AA, Abeer E-KMS, Al-Sokary ET, Elbaz S, et al. CINNAMON NANO PARTICLES (CNPs) AS FEED ADDITIVES ON RABBIT GROWTH PERFORMANCE, IMMUNITY, ANTIOXIDANT CAPACITY, AND RESISTANCE TO ESCHERICHIA COLI. Assiut Veterinary Medical Journal. 2025;71: 608–634. doi:10.21608/avmj.2025.320513.1399

42. Yabo SA, Manga SB, Baki AS, Atata RF, Gambo S, Tahir H. Antibacterial activity and toxicological evaluation of Anogeissusleiocarpus and Psidium guajava on Escherichia coli and Staphylococcus aureus. Bayero Journal of Pure and Applied Sciences. 2021;14: 214–220.

43. Sonawane R. A review on the medicinal plant-psidium Guajava Linn. Int J Pharm Res Appl. 2021;6: 311–329.

Formatted: Justified

Response to Reviewers' Comments

Comments reviewers and editor: We thank the Editor and the three Reviewers for their constructive evaluation of our manuscript. Based on they inputs and recommendations, we have revised the entire manuscript, remade all figures and figure captions. Below, we tried to the best of our ability to respond point-by-point to every comment. Reviewer comments are reproduced in bold italics; our responses follow in red.

Slight change in the title: The original title was revised to align with the revised manuscript. The new title is: A Combined Aqueous Extract of *Psidium guajava* L. and *Tithonia diversifolia* (Hemsl.) A. Gray Improves Metabolic Homeostasis and Preserves Intestinal Integrity in Experimental Rabbit Colibacillosis

Manuscript ID: PONE-D-26-07694

Reviewer #1

The manuscript is interesting, but some corrections need to be addressed.

Comment 1: *The volume of the bacterial suspension used for induction should be included.*

Response 1: The disease was induced by administering 1 mL /100 g of body weight of bacterial suspension, adjusted to a 10^9 CFU/mL in 0.9% saline. This information has been added to the Materials and Methods section.

Comment 2: *The software used for data analysis should also be included.*

Response 2**:** Data were analysed using GraphPad Prism and OriginPro, and multivariate analysis was performed in the R environment. This information has been added to the corresponding Methods section (see also Reviewer #2, Comment 10, for the full software citation).

Comment 3: *The term "polyherbal extract" should be replaced with "the combined extract of …".*

Response 3: The term "polyherbal extract" has been replaced throughout the manuscript with "the combined *Psidium guajava* and *Tithonia diversifolia* extracts," as suggested.

Comment 4: *The preparation of the combined extract should be described, and the rationale for the doses used in the experiment should be explained (the doses appear high).*

Response 4: The preparation of the combined extract has been fully described in the Methods section. Briefly, the two extracts were combined in a 96.975% *P. guajava* : 3.025% *T. diversifolia* ratio (w/w), determined from the synergistic Fractional Inhibitory Concentration Index (FICI = 0.265) established in our prior in vitro work (Chouegouong et al., 2023;

doi:10.1007/s13596-021-00615-1). Fresh stock solutions were prepared daily in sterile distilled water at 40 mg/mL and 80 mg/mL to deliver 400 and 800 mg/kg bw, respectively. The administration was done by oral gavage at a fixed volume of 10 mL/kg bw (i.e., 387.9 mg/kg *P. guajava* + 12.1 mg/kg *T. diversifolia* for the low dose, and 775.8 mg/kg *P. guajava* + 24.2 mg/kg *T. diversifolia* for the high dose). The selected doses (400 and 800 mg/kg bw) are consistent with standard pharmacological evaluations of crude phytotherapeutic agents in lagomorph models, as previously reported by Hirudkar et al. (2020); this rationale, together with the full preparation protocol, has been added to the Methods section.

Comment 5: *Most sentences should be structured appropriately; sentences should not begin with "to" or "by".*

Response 5: Constructions beginning with "To" or "By" have been revised throughout the manuscript to improve phrasing and fluency.

Comment 6: *The explanations of the results obtained do not accurately reflect the results or experiments performed; there is considerable overstatement.*

Response 6: The results section has been rephrased to remove overstatement and to ensure that the interpretations presented are directly supported by the data obtained.

Comment 7: *The conclusion should be rewritten to accurately reflect the work done, both in the abstract and throughout the manuscript.*

Response 7: The conclusion has been rewritten in both the abstract and the main text to align precisely with the results obtained (see also Reviewer #2, Comment 12, and Reviewer #3, Minor Comment 6).

Comment 8: *The manuscript should be rewritten to better reflect the results obtained; the figures are confusing for the reader.*

Response 8: The manuscript has been revised to better reflect the data generated, all figures are now explicitly cited and described within the text, and every figure legend has been rewritten to be self-contained and self-interpretable (see the accompanying Revised Figure Legends document, and Reviewer #2, Minor Comment 4).

Comment 9: *The manuscript contains some inconsistencies, which have been highlighted.*

Response 9: Thank you to all reviewers for flagging these inconsistencies. We have revised the entire manuscript and checked all results individually against the raw data and corrected; the corresponding changes are marked in the revised manuscript.

Reviewer #2

Comment 1: *Some grammatical, alignment, and typographical errors are noted in the manuscript; it should be thoroughly checked and corrected throughout.*

<u>Response 1:</u> We thank the Reviewer for this observation. We have carefully re-read the entire manuscript and performed a thorough revision to correct all grammatical, alignment, and typographical errors identified.

Comment 2: *The use of abbreviations in the abstract may distract readers who wish to quickly skim several publications before deciding to read one in full. It may help to write terms out in full in the abstract (for example, AST).*

<u>Response 2:</u> We have revised the abstract to write out all abbreviated terms in full.

Comment 3: *Abbreviations should be checked throughout the manuscript: the full term should be introduced at first mention in the abstract and again at first mention in the main text, after which only the abbreviation should be used (for example, IL-1β, IL-6, superoxide dismutase (SOD), etc.). Only terms repeated at least three times in the text should be abbreviated.*

<u>Response 3:</u> We have thoroughly reviewed all abbreviations throughout the manuscript, introducing each full term at first mention before subsequent use of the abbreviation, and removing abbreviations for terms that did not meet the repetition threshold. The corresponding revisions have been incorporated into the updated manuscript and into the revised figure legends (see accompanying document).

Comment 4: *The full species name should be given at first mention in both the abstract and the main text, followed thereafter by the abbreviated genus initial (for example, Psidium guajava / Tithonia diversifolia at first mention, followed by P. guajava / T. diversifolia); this should be checked for all species used in the manuscript.*

<u>Response 4:</u> We have revised the entire manuscript and edited the full species of the two plants.

Comment 5: *The time (season) of collection of the plant species should be added, since the collection period is known to affect phytochemical constituents; this should be given top priority.*

<u>Response 5:</u> We completely agree with the reviewer that transparency in botanical processing is fundamental for reproducibility. We have now updated the "Ethnobotanical Selection and Extract Preparation" section to include the exact exact collection dates and season for both plant species, the starting fresh weights, resulting dry powder weights, and calculated fresh-to-dry ratios for both species.

Comment 6: *The quantity of fresh plant material used and the quantity of powder obtained (i.e., the fresh-to-powder ratio) should be included under Materials and Methods.*

<u>Response 6:</u> We have added the quantities of fresh plant material and the corresponding dry powder yield to the Materials and Methods section.

Comment 7: *The standard diet used in the study should be described, including its composition and source.*

Response 7: Information regarding the composition of the standard diet has been incorporated into the revised manuscript.

Comment 8: *In the Materials and Methods, references should be cited for any standard protocols or manufacturer instructions used, and the corresponding citations should be added to the reference list.*

Response 8: The Methods section has been updated to include references for the standard protocols used, and the corresponding citations have been added to the reference list.

Comment 9: *The dose selected for the present study should be justified in the Materials and Methods, either based on the available literature or a previous study; if derived from another study, it should be supported with the appropriate reference.*

Response 9: The dose justification, supported by the relevant reference (Chouegouong et al., 2023; Hirudkar et al., 2020), has been incorporated into the revised Materials and Methods section (see also Reviewer #1, Comment 4).

Comment 10: *The authors should cite the software fully when referring to it. GraphPad Prism version 8.0.1 (GraphPad Software, San Diego, CA, USA).*

Response 10: We have updated the Methods section to include this full software citation.

Comment 11: *Necessary expansions should be given in the figure legends, for example for MDA, SOD, CAT, and GSH.*

Response 11: We have rewritten every figure legend so that all abbreviations (including MDA, SOD, CAT and GSH) are expanded at first mention within each individual legend, in line with journal style for self-contained legends. Please see the accompanying Revised Figure Legends document.

Comment 12: *The conclusion provides a comprehensive overview of the work but could be more realistic in relation to the evidence presented, to better convey the outcome of the investigation. The authors may also wish to include the limitations of the present findings.*

Response 12: We have revised the conclusion to be more precise and directly grounded in the evidence generated by the present study, and we have added a dedicated statement on the limitations of the study and on future investigations, as recommended (see also Reviewer #3, Comments A–D, H, and Minor Comment 6).

Reviewer #3

Major Comments

Comment A — Rationale for the infection model and experimental design: *The authors induced infection after administering both cyclophosphamide and streptomycin prior to E. coli*

inoculation. This creates a highly artificial disease model. Are the observed pathological changes truly due to E. coli infection, or are they partly due to cyclophosphamide-induced immunotoxicity and streptomycin-induced dysbiosis? The rationale for combining cyclophosphamide and streptomycin should be clearly explained.

Response A1: We thank the Reviewer for raising this critical point. We agree that pretreatment with cyclophosphamide and streptomycin adds complexity to the physiological baseline; however, this combination follows a well-established, highly reproducible methodological precedent for inducing an acute, standardised intestinal *E. coli* infection in adult rabbits (Nguiam et al., 2021), for two complementary reasons: (1) streptomycin transiently clears the facultative anaerobic Gram-negative gut microflora that would otherwise confer strong colonisation resistance, opening an ecological niche that allows the challenge strain to colonise and proliferate; and (2) cyclophosphamide induces transient immunosuppression that mimics the opportunistic susceptibility seen in young, weanling, or stressed animals in the field, ensuring a reliable, synchronised onset of acute clinical colibacillosis across cohorts. This rationale has been added to the revised Methods section.

Addressing Potential Confounding Pathological Changes:

To determine whether the pathological changes observed were attributable to the infection itself rather than to the pretreatment, we included a dedicated immunosuppression-only control group (Group T5), which received the same cyclophosphamide pretreatment but was not inoculated with *E. coli* (see Response A2 below for the exact T5 regimen). By Day 7 post-infection (9 days post-pretreatment), Group T5 showed no significant differences ($P > 0.05$) from the healthy, untreated control group (T0) in clinical scores, body weight gain, hematological profile, or tissue histopathology, whereas the infected, untreated group (T1) exhibited severe diarrhoea, weight loss, systemic inflammation, and marked histopathological damage. This parity between T5 and T0 indicates that the physiological and immunological perturbations caused by the pretreatment were transient and largely resolved by the study endpoint, supporting the interpretation that the pathology documented in the infected groups reflects the *E. coli* challenge.

We nonetheless agree that the reliance on chemically induced susceptibility is an inherent limitation of this model, and we have added this point explicitly to the new "Limitations of the Study" subsection of the Discussion.

Comment - *The manuscript includes an "immunosuppression control" group (T5), without a sufficiently detailed description of the treatment regimen. The authors should clarify whether T5 received cyclophosphamide alone or both cyclophosphamide and streptomycin.*

Response A2: We confirm that Group T5 (Immunosuppression Control) received cyclophosphamide alone and did not receive streptomycin, so as to isolate the biological effects of chemical immunosuppression on hematological profile, oxidative stress, and tissue architecture, independent of antibiotic-induced gut dysbiosis and of the *E. coli* challenge. We have expanded the Materials and Methods to state explicitly that animals in Group T5 received a single intraperitoneal injection of

cyclophosphamide (30 mg/kg bw) 48 hours before the designated infection timeline, did not undergo streptomycin clearance or bacterial inoculation, and received daily oral gavage of vehicle (10 mL/kg bw sterile distilled water) for 7 consecutive days starting 24 hours post-induction, matching the handling stress and administration volume of the treated cohorts.

Comment- *The authors need to discuss the limitations of this highly manipulated infection model, as cyclophosphamide itself can induce oxidative stress, haematological changes, and organ toxicity. Accordingly, several reported outcomes may not solely reflect colibacillosis.*

Response A3: We agree that cyclophosphamide is a potent alkylating agent capable of inducing transient myelosuppression, systemic oxidative stress, and organ toxicity, and that this is an important limitation of chemically facilitated infection models. To isolate the contribution of cyclophosphamide from that of the infection, we compared Group T5 (cyclophosphamide alone, uninfected) with Group T0 (healthy control) and Group T1 (infected, untreated). By Day 7 post-infection, the hematological, biochemical, and tissue lipid-peroxidation parameters of Group T5 were statistically indistinguishable from those of Group T0 ($P > 0.05$), in clear contrast to the severe, persistent leukopenia, systemic inflammation (elevated TNF-α, IL-6, CRP), oxidative stress (elevated MDA; depleted SOD and catalase), and tissue damage observed in Group T1. This indicates that the acute toxicological effects of cyclophosphamide had largely resolved by the time of sample collection, although we agree that early-stage cyclophosphamide toxicity remains a confounding variable and a limitation of the model. A dedicated "Limitations of the Study" subsection has been added to the Discussion, and the T5-versus-T0/T1 comparison is now presented there explicitly to help the reader distinguish the relative contribution of cyclophosphamide from that of the infection.

Comment B — Lack of phytochemical characterisation and extract standardisation: *The major weakness of the study is the absence of phytochemical profiling and standardisation of the two extracts used. The authors repeatedly attribute the biological effects to the presence of flavonoids, tannins, and sesquiterpene lactones, and to NF-κB modulation, without quantification or characterisation of these compounds (e.g., by HPLC, LC-MS/MS, total phenolic/flavonoid quantification, or chromatographic fingerprinting). Otherwise, the mechanistic claims should be substantially toned down.*

Response B: Our previous study (Chouegouong et al., 2023) reported the chemical fingerprint of these same two extracts, confirming the presence of several chemical classes, including terpenes, flavonoids, and tannins; this reference has been cited in the revised manuscript. Moreover, we also performer an LC-MS analysis of crude extracts from *Psidium guajava* L. and *Tithonia diversifolia* that allow us to annotate **Pedunculagin (**from *P. guajava***),** and **Quercetin, Quercetin 4′-O-glucoside, Myricetin-3-O-pentoside,** and **2′-Hydroxy-alpha-naphthoflavone** (from *T. diversifolia*). This new information has been added to the discussion and the supplementary materials.

Moreover, all mechanistic statements attributing the observed effects to specific phytochemical classes or to NF-κB modulation have been moderated and reframed as plausible hypotheses consistent with the prior phytochemical characterisation, rather than as demonstrated mechanisms (see also Comment E).

C) Small sample size and statistical power concerns

The study used only n = 5 rabbits/group, with one mortality in the untreated infected group and only four animals/group ultimately analyzed histologically and biochemically. The endpoints measured are quite large in comparison with the number of animals per group. Accordingly, The authors should**:**

• provide statistical power justification,

• clarify whether normality testing was performed,

• explain how multiple testing inflation was controlled,

• and discuss the limitations imposed by the small sample size.

Response C: We completely acknowledge the reviewer's valuable methodological concerns regarding statistical power, sample sizes, and the risk of Type I and Type II errors when evaluating multiple physiological endpoints. We have addressed each point as follows and updated the Statistical Analysis and Limitations subsections accordingly.

1. Statistical power: The initial (n = 5/group) and final analytical (n = 4/group, following early humane euthanasia in Group T1 and the planned reduction of one animal per surviving cohort under the 3Rs framework) sample sizes were set based on the large, polarised effect sizes typically observed between healthy and untreated-infected phenotypes in acute lagomorph enteric infection models. An a priori power analysis indicated that for a large anticipated effect size (Cohen's $f \geq 0.70$), $n = 4$ animals per group is sufficient to achieve power ($1 - \beta$) of 0.80 at $\alpha = 0.05$, balancing statistical reliability against the 3Rs (Reduction) principle.

2. Normality testing: Data normality was assessed using the Shapiro-Wilk test, and homogeneity of variance using Levene's test; all datasets satisfied these assumptions ($P > 0.05$), supporting the use of parametric statistical models.

3. Multiple-testing control: To avoid Type I error inflation from repeated pairwise comparisons across six groups, all multi-group variables were analysed by one-way ANOVA followed by Tukey's HSD post-hoc test, which controls the family-wise error rate at $\alpha = 0.05$.

4. Limitations: We acknowledge that, while adequately powered for the large-magnitude primary therapeutic effects observed, the small sample size restricts our ability to detect subtler or secondary effects and may not fully capture natural physiological variability in outbred rabbit populations; this is now explicitly stated in the Limitations of the Study.

D) Absence of quantitative histopathology evaluation

The histopathological analysis is entirely descriptive mentioning "leukocyte infiltration, villous atrophy, restoration," etc. However, no histological scoring system was used, and no semi-quantitative analysis was provided. The authors should: introduce validated histopathological

scoring systems, quantify inflammatory infiltration and villus height/crypt depth, and provide lesion severity scores.

Response D: We completely agree with the reviewer that semi-quantitative scoring and morphometric measurement (e.g., villus height, crypt depth) would add valuable numerical depth. At the time of this study, our facility did not have access to digital histomorphometric software or automated image-analysis systems required for these sub-micron measurements in lagomorph intestinal tissue; the histopathological evaluation was therefore designed as a rigorous, descriptive, qualitative assessment. To limit subjectivity, sections were evaluated by a blinded observer focused on identifying unambiguous, pathognomonic signs of acute colibacillosis (e.g., mucosal desquamation, denuded villi, extensive inflammatory infiltrates). As shown in the revised figures, the structural differences between groups are marked: the infected-untreated group shows extensive destruction of intestinal architecture, while the high-dose extract group shows visibly preserved mucosal integrity comparable to the healthy control. We have added the absence of semi-quantitative scoring as an explicit limitation in the Discussion and clarified the qualitative nature of the histological methodology in the Materials and Methods.

E) Cytokine and mechanistic claims are overstated

The authors repeatedly conclude that the extracts used caused NF-κB inhibition, immunomodulation, ROS scavenging mechanisms, membrane stabilization, and bacterial adhesion inhibition, without experimental evaluation of these pathways. Only general screening parameters were measured. Mechanistic statements should be rewritten more cautiously and presented as hypotheses rather than demonstrated mechanisms.

For example: “may involve modulation of inflammatory pathways”

instead of: “likely mediated through NF-κB inhibition.”

Response E**:** We have rewritten the Results and Discussion so that mechanistic statements are consistently framed as hypotheses supported by our observed endpoints (e.g., “may involve modulation of inflammatory pathways”) rather than as demonstrated mechanisms (e.g., “likely mediated through NF-κB inhibition”), throughout the manuscript.

Comment F — Important missing methodological details

Several experimental details are insufficiently described, including the following:

Randomisation: the manuscript states that rabbits were “randomly assigned,” but the randomisation method is not described.

Response F1 — Randomisation**:** We thank the reviewer for pointing out this missing methodological detail. We agree that specifying the exact method of randomization is essential for transparency and reproducibility. In the revised manuscript, we have updated the Materials and Methods section to explicitly describe the randomization protocol. Specifically, we have clarified that a simple randomization procedure was performed using a computer-generated random number sequence

(https://www.random.org/lists/), ensuring that each animal had an equal and independent probability of being assigned to any of the six experimental cohorts.

Response F2 — Blinding**:** To eliminate observer and detection bias, a strict blinding protocol was implemented across multiple stages of the study. The investigators conducting daily clinical scoring were blinded to the treatment groups by having an independent colleague manage the preparation and coding of the treatment solutions. Furthermore, all downstream laboratory analyses, including hematological processing, biochemical biomarker assays, and histopathological tissue evaluations were performed by investigators who were completely blinded to the experimental groups. All samples and tissue slides were labeled with anonymous, randomized tracking codes, which were only unblinded after the final statistical analysis was completed.

Sex distribution: the rabbits were of "mixed sex," but the distribution per group was not provided. Sex-dependent differences may influence the immune response.

Response F3 — Sex distribution**:** Statistical analysis revealed no significant difference between males and females in infection or treatment response under the present experimental conditions. The sex distribution per group (2 males / 3 females per group) has been added to the revised manuscript.

Extract formulation: the exact ratio between the two combined extracts is not clearly stated.

Response F4 — Extract formulation: Combined extract formulations of *P. guajava* and *T. diversifolia* were freshly prepared in distilled water daily. The ratio of the two extracts within the combination (96.975% *P. guajava* and 3.025% *T. diversifolia*) was determined based on the synergistic Fractional Inhibitory Concentration Index (FICI = 0.265) identified in our prior *in vitro* assays (Chouegouong et al., 2023). To administer the target doses of 400mg/kg and 800 mg/kg body weight (bw) at a standardized oral gavage volume of 1 mL per 100 g bw (10 mL/kg bw), two distinct stock solutions were prepared. The stock solution of 40 mg/mL (for the 400 mg/kg group) was prepared by dissolving 310.32 mg of *P. guajava* extract and 9.68 mg of *T. diversifolia* extract in 8 mL of sterile distilled water. This delivered 387.9 mg/kg of *P. guajava* and 12.1 mg/kg of *T. diversifolia in vivo*. The second stock solution of 80mg/mL (for the 800mg/kg group) was Prepared by dissolving 620.64 mg of *P. guajava* extract and 19.36 mg of *T. diversifolia* extract in 8mL of sterile distilled water. This delivered 775.8mg/kg of *P. guajava* and 24.2 mg/kg of *T. diversifolia in vivo*.

Comment G: *Bacterial characterisation is insufficient. The isolate was identified only by API 20E and the Congo red binding assay. However, the study repeatedly refers to EPEC, virulence, pathogenicity, and attaching/effacing lesions. No molecular confirmation of virulence genes was performed. The authors should avoid definitive classification as EPEC unless molecular virulence markers are confirmed, or should include PCR confirmation for the relevant virulence genes.*

Response G: We agree that definitive classification as Enteropathogenic *E. coli* (EPEC) requires molecular confirmation of diagnostic virulence markers (e.g., eaeA, bfpA) by PCR, which was not performed here; our isolate was characterised only phenotypically, using the API 20E system for species-level identification (Fig 1) and the Congo red binding assay to confirm pathogenic phenotype. We have therefore removed the term "EPEC" throughout the manuscript (Abstract, Introduction, Methods, Results, Discussion) and replaced it with "pathogenic *E. coli*", and we have toned down or rephrased speculative references to specific microscopic attaching/effacing lesions to describe generalised enteropathogenic tissue alterations consistent with our phenotypic and histological data.

Comment H: *PCA and multivariate analysis appear overinterpreted. The PCA and heatmap analyses are visually attractive, but the sample size is small, the number of variables is large, and no validation strategy is described. Statements such as "biological reset" and "systemic recovery indistinguishable from healthy baseline" are exaggerated given the exploratory nature of PCA. The authors should moderate their interpretation, clarify the variance explained, and discuss the limitations of multivariate modelling with small datasets.*

Response H: We agree that applying PCA to a high-dimensional dataset (46 variables) with a small sample size (n = 4/group) without an independent validation cohort carries a substantial risk of overfitting, and that these results must not be overinterpreted. We have removed exaggerated or definitive claims (e.g., "mathematically demonstrates", references to an absolute "biological reset"), clarified the exact variance explained by each dimension (Dim1 = 48%, Dim2 = 9.7%; Fig 10), and explicitly reframed the PCA and heatmap as descriptive, exploratory visualisations rather than predictive or confirmatory models. A statement on the limitations of multivariate analysis with small sample sizes has been added to the Discussion (see also the revised legends for Figs 9 and 10).

Comment I: *The discussion following several results sections lacks sufficient referencing and critical comparison with the literature. The authors should discuss their findings in relation to previous studies, emphasising agreements, discrepancies, possible explanations, and remaining research gaps. For example, reports on Psidium species indicate antidiarrheal activity through antimicrobial effects, not solely through tannins (DOI: https://doi.org/10.1016/j.jep.2025.119670).*

Response I: We have substantially revised the Discussion to integrate and critically compare our findings with the relevant literature, including the reference suggested by the Reviewer (DOI: 10.1016/j.jep.2025.119670) on antimicrobial, rather than purely tannin-mediated, antidiarrheal mechanisms of Psidium species. All new text is original and properly cited, and the corresponding changes are indicated in the revised manuscript (see also Minor Comment 2).

Minor Comments

Minor Comment 1: *Several sections contain overly promotional language inappropriate for scientific writing, including:*

- "biological reset"
- "high-activity extract"

- “profound therapeutic trajectory”
- “gold-standard treatment”
- *“extinguishing inflammatory cascades.”*

The manuscript should adopt a more objective scientific tone.

Response 1: All overstated expressions, including those listed, have been replaced with objective, scientifically appropriate language throughout the manuscript.

Minor Comment 2: *The discussion is excessively long and repetitive. Several statements restate the results instead of critically interpreting them (see also Comment I above).*

Response 2**:** To eliminate redundancy, we condensed and refocused the Discussion. The revised text (1) compares our findings with previous studies, (2) proposes plausible mechanistic pathways while explicitly noting their hypothetical status, (3) discusses the constraints of the present study, and (4) outlines specific follow-up experiments (fractionation, adhesion assays, pharmacokinetics, safety/toxicology). References have been reorganised to support this structure.

Minor Comment 3: *The authors should clarify whether the units used for ALT and AST, cytokine concentrations, and oxidative stress markers were standardised and validated for rabbit samples (i.e., that the kits used are validated for use with rabbit samples).*

Response 3: We have revised the Materials and Methods section to clearly specify the assay validation, species applicability, and units of measurement for all biochemical and immunological parameters.

Minor Comment 4: *Figure legends appear insufficiently detailed. They should be revised to be self-interpretable in some sections, including magnification, stain details, scale bars, and statistical annotations.*

Response 4: We agree that legends must be fully self-interpretable. All 14 figure legends have been rewritten (see the accompanying Revised Figure Legends document): each now states the sample size (n = 4 animals/group analysed), expands all technical abbreviations at first mention, and specifies the statistical test and software used. For the histopathological figures (Figs 12–14), the legends now specify hematoxylin and eosin staining, original magnification (×100), and a scale bar; because scale-bar values were not recorded in the original image files available to us, these are flagged with a placeholder for the corresponding author to insert before resubmission.

Minor Comment 5: *Adherence to the ARRIVE guidelines is mentioned, but several ARRIVE-required elements remain incomplete, namely sample size justification, exclusion criteria, and blinding details.*

Response 5: The Methods section now includes an explanation of the sample size (see Response C), the predetermined inclusion/exclusion criteria, and a full description of the blinding procedure at the clinical-scoring, laboratory-analysis, and data-interpretation stages (see

Response F2). The updated ARRIVE 2.0 checklist (https://arriveguidelines.org/arrive-guidelines) is submitted as a supplementary submission

Minor Comment 6: *The conclusion somewhat overstates translational applicability. The statement that the extract is "clinically equivalent to conventional aminoglycosides" is not fully supported without pharmacokinetic data, toxicity studies, dose optimisation, long-term safety data, and field validation. The conclusion must be revised accordingly.*

Response 6: The conclusion has been rewritten to remove the claim of clinical equivalence to conventional aminoglycosides and to present the translational potential of the extract in a manner consistent with the evidence available (comparable performance on the specific biomarkers measured at Day 7, without pharmacokinetic, long-term safety, or field validation data), in line with the newly added Limitations of the Study.
