# Supplementary material for: A combined aqueous extract of Psidium guajava L. and Tithonia diversifolia (Hemsl.) A. gray improves metabolic homeostasis and preserves intestinal integrity in experimental rabbit colibacillosis
Source: PLoS One. 2026 Sep 15;21(9):e0357951. doi: 10.1371/journal.pone.0357951 (PMC13577389; doi:10.1371/journal.pone.0357951)
Supplement: Supplementary file 3 [file pone.0357951.s001.docx]

**S1 Figure:** Red-pigmented colonies on the Congo Red binding assay (CR+)

**S1 Table:** Identification (ID) Code and Responses of Isolate Identified as *E. coli* based on the API 20E.

|  | **SAMPLES** | **LK10** |
| --- | --- | --- |
|  | ID Code | 5044572 |
|  | **ONPG** | + |
|  | **ADH** | **-** |
| **Test result** | **LDC** | **+** |
|  | **ODC** | **-** |
|  | **CIT** | **-** |
|  | **H_2_S** | **-** |
|  | **URE** | **-** |
|  | **TDA** | **-** |
|  | **IND** | **+** |
|  | **VP** | **-** |
|  | **GEL** | **-** |
|  | **GLU** | **+** |
|  | **MAN** | **+** |
|  | **INO** | **-** |
|  | **SOR** | **+** |
|  | **RHA** | **+** |
|  | **SAC** | **+** |
|  | **MEL** | **+** |
|  | **AMY** | **-** |
|  | **ARA** | **+** |
|  | **OX** | **-** |

Biochemical tests: ONPG, 2-nitrophenyl-β-D-galactopyranoside to detect β-galactosidase; ADH, arginine dihydrolase; LDC, lysine decarboxylase; ODC, ornithine decarboxylase; CIT, citrate utilization; H_2_S, H_2_S production; URE, urease; TDA, tryptophan deaminase; IND, indole production; VP, Voges-Proskauer test for acetoin; GEL, gelatinase; GLU, glucose fermentation; MAN, mannitol fermentation; INO, inositol fermentation; SOR, sorbitol fermentation; RHA, rhamnose fermentation; SAC, saccharose (sucrose) fermentation; MEL, melibiose fermentation; AMY, amygdalin fermentation; ARA, arabinose fermentation; OX, oxidase.

**S2 Table:** Effect of the combined aqueous extracts of Psidium guajava and Tithonia diversifolia on the body weight variation in colibacillosis-infected rabbits.

|  | DAY 5: (infection) | DAY 7 (treatment) | DAY 9 (treatment) | DAY 11 (treatment) | DAY 13 (Sacrifice) |
| --- | --- | --- | --- | --- | --- |
| T0 | 0±0 | 40,25±1,60 | 45±9,66 | 124,5±,32 | 164±12,06 |
| T1 | 0±0 | -46±10,92 | -51,25±11,84 | -139,25±67,09 | -212,25±53,50 |
| T2 | 0±0 | -38,33±0,23 | 34,83±0,44 | 29,5±,19 | 75,25±8,02 |
| T3 | 0±0 | -41±3,34 | 38±0,91 | 33,5±3,47 | 71,25±4,26 |
| T4 | 0±0 | -26,75±6,34 | 36,25±1,31 | 48,25±7,70 | 84±8,02 |
| T5 | 0±0 | 37,5±0,95 | 39±1,77 | 112,25±2,75 | 132±17,31 |

**S3 Table:** Dynamics of fecal *E. coli* shedding in rabbits following treatment with combined *P. guajava* and *T. diversifolia* extracts.

|  | DAY 3 (Immunodepression: 48hours) X 10^7^ | DAY 5: (infection)  X 10^7^ | DAY 6 (treatment) | DAY 7 (treatment)  X 10^7^ | DAY 8 (treatment) X 10^7^ | DAY 9 (treatment) X 10^7^ | DAY 10 (treatment) X 10^7^ | DAY 11 (treatment) X 10^7^ | DAY 12 (treatment) X 10^7^ |
| --- | --- | --- | --- | --- | --- | --- | --- | --- | --- |
| T0 | 0±0 | 0±0 | 0±0 | 0±0 | 0±0 | 0±0 | 0±0 | 0±0 | 0±0 |
| T1 | 0±0 | 0±0 | 20±0,81 | 21,25±1,03 | 22,5±1,04 | 37,25±1,43 | 42,5±1,04 | 44,5±3,32 | 43,25±2,05 |
| T2 | 0±0 | 0±0 | 20±0,81 | 10,25±0,85 | 0,75±0,25 | 0±0 | 0±0 | 0±0 | 0±0 |
| T3 | 0±0 | 0±0 | 21,5±1,55 | 19±1,47 | 12,5±1,04 | 7±1,22 | 1,5±0,86 | 0,75±0,47 | 0±0 |
| T4 | 0±0 | 0±0 | 20,5±0,64 | 12,5±1,04 | 6,75±0,85 | 2,25±0,94 | 0,25±0,25 | 0±0 | 0±0 |

**S4 Table:** Effect of combined extracts of *Psidium guava* and *Tithonia diversifolia* on Oxydative stress parameters in experimentally infected rabbits**.**

|  | **T0** | **T1** | **T2** | **T3** | **T4** | **T5** |
| --- | --- | --- | --- | --- | --- | --- |
| **CATALASE ILEON mM de H_2_0_2_ /minute/g d’organe** | 8.73±0.88* | 3.46±1.65 | 8.971±0.64* | 11.82±5.66** | 8.63±1.81* | 8.08±0.68 |
| **CATALASE CAECUM mM de H_2_0_2_ /minute/g d’organe** | 9.68±1.33**** | 4.11±0.79 | 10.48±1.71**** | 11.05±0.919**** | 12.24±1.36**** | 8.95±1.15*** |
| **GSH ILEO x 10^-6^µmol/g d’organes** | 2.28±0.77* | 0.75±0.18 | 1.783±0.64 | 2.06±0.95** | 1.98±0.73 | 2.38±0.15** |
| **GSH CAECUM x 10^-6^µmol/g d’organes** | 4.88±1.16 | 2.02±0.47 | 6.047±2.47 | 4.97±2.52 | 5.82±2.62 | 5.70±2.61 |
| **SOD ILEON nombre d’unité de SOD/g d’organe** | 1.63± 0.08**** | 0.714±0.08 | 1.22±0.10*** | 1.26±0.13*** | 1.44±0.25**** | 1.23±0.14**** |
| **SOD CAECUM nombre d’unité de SOD/g d’organe** | 1.53±0.15* | 0.76±0.05 | 1.22±0.22* | 1.26±0.34* | 1.38±0.19** | 1.70±0.20**** |
| **MDA ILEON x 10^-6^ µmol/g d’organes** | 11.58±1.72** | 15.32±1.21 | 9.01±0.59**** | 7.68±1.59**** | 7.05±1.80**** | 12.70±1.64 |
| **MDA CAECUM X 10^-6^ µmol/g d’organes** | 5.93±0.70**** | 14.04±0.25 | 5.34±0.64**** | 5.51±1.07**** | 5.74±2.47**** | 5.91±0.59**** |

Values are expressed as mean ± SEM.Significance are determined with p-values: *p < 0.05, **p < 0.01, ***p <0.001, ****p < 0.0001.

**S5 Table:** Impact of treatments using a combined aqueous extract of *Tithonia diversifolia* and *Psidium guajava* on the haematological parameters of infected animals

|  | **T0** | **T1** | **T2** | **T3** | **T4** | **T5** |
| --- | --- | --- | --- | --- | --- | --- |
| **WBC** | 7.60±0.29**** | 21.88±4.28 | 12.15±0.12**** | 14.25±0.12**** | 11.13±0.92**** | 6.10±0.74**** |
| **LYM%** | 31.60±2.54 | 33.90±3.20 | 44.15±1.36*** | 31.85±1.93 | 35.80±3.17 | 31.03±3.30 |
| **MID%** | 6.20±0.69 | 6.07±0.58 | 8.400±0.81* | 7.050±1.43 | 8.075±1.21* | 5.425±0.71 |
| **GRA%** | 52.75±1.461* | 57.23±2.96 | 57.20±0.71 | 54.90±1.94 | 53.58±2.01 | 45.08±2.67**** |
| **RBC (X 10^6^ µL)** | 9.55±1.04*** | 6.96±0.17 | 8.450±0.46 | 8.675±1.03* | 9.07±0.71** | 9.18±0.47** |
| **HGB** | 15.20±1.91** | 10.90±0.69 | 14.35±1.33* | 13.40±1.61 | 15.50±1.23* | 15.88±1.34*** |
| **HCT** | 40.45±2.98*** | 31.93±1.57 | 37.68±1.42* | 37.28±3.94* | 45.20±2.28* | 40.95±2.29*** |
| **PLT** | 365.5±42.81**** | 762±185.27 | 408.80 ±9.74**** | 404.50±10.63**** | 401.00±14.89**** | 354.00±16.43**** |

**S6 Table:** Effect of treatments with extract plants on cytokines values in experimentally infected rabbits.

|  | **T0** | **T1** | **T2** | **T3** | **T4** | **T5** |
| --- | --- | --- | --- | --- | --- | --- |
| **TNF alpha (pg/mL)** | 9.45±1.45* | 22.32±8.40 | 8.59±4.63* | 9.25±4.38* | 9.49±3.42* | 8.75±2.31* |
| **INF gama (ng/mL)** | 2.28±0.45 | 3.52±0.24 | 2.78±0.69 | 2.12±1.02* | 1.78±0.61* | 1.67±0.60* |
| **IL 10 (pg/mL)** | 67.48±9.80* | 38.77±8.66 | 60.34±12.58* | 47.89±10.44* | 67.26±21.51* | 67.37±15.34 |
| **IL 6 (pg/mL)** | 6.21±0.47* | 10.89±1.49 | 8.81±0.86* | 8.35±1.27* | 7.41±0.94* | 6.67±0.14* |
| **IL 1B (pg/mL)** | 12.53±1.56* | 21.14±2.20 | 14.89±1.09* | 19.14±1.11 | 15.30±2.69* | 13.35±1. 62* |
| **CRP (ng/mL)** | 10.69±1.44* | 23.00±1.55 | 14.76±0.60* | 17.13±4.73* | 13.79±2.27* | 14.23±2.97* |

TNF alpha: Tumor Necrosis Factor alpha, INF gama: Interferon gamma (IFN-γ), , IL 10: Interleukin-10, IL 6: Interleukin-6, IL 1B: Interleukin-1 beta, CRP:C-reactive protein. Values are expressed as mean ± SEM.Significance are determined with p-values: *p < 0.05.

**S7 Table:** Effect of combined extracts of *Psidium guava* and *Tithonia diversifolia* on the biochemical parameters in experimentally infected rabbits

|  | **T0** | **T1** | **T2** | **T3** | **T4** | **T5** |
| --- | --- | --- | --- | --- | --- | --- |
| **ALAT (UI/L)** | 58.19±7.07 | 75.25±16.24 | 55.61±11.43 | 63.56±10.89 | 54.58±4.45 | 59.44±11.72 |
| **ASAT (UI/L)** | 107.8±8.67**** | 167.6±11.55 | 112.20±1.53**** | 150.20±12.86* | 112.30±1.24**** | 105.10±5.50**** |
| **Creatinin (mg/dL)** | 0.94±0.16**** | 3.011±0.85 | 1.73±0.12** | 2.61±0.11 | 1.05±0.13**** | 1.14±0.44**** |
| **Uric acid (mg/dL)** | 3.09±0.36**** | 5.418±0.77 | 4.91±0.43**** | 3.77±0.21*** | 4.03±0.62** | 3.00±0.35**** |
| **GGT (UI/L)** | 3.67±1.39**** | 11.18±1.30 | 3.69±0.76* | 6.67±1.73*** | 4.15±1.21**** | 3.29±1.12**** |
| **proteins (mg/dL)** | 3.46±0.20** | 2.447±0.31 | 3.96±0.21*** | 3.70±0.54*** | 3.26±0.16** | 3.39±0.31** |
| **Albumine (g/L)** | 27.07±2.31** | 18.54±1.91 | 24.41±1.21* | 23.59±3.74 | 26.29±3.63** | 24.02±1.81 |
| **HDL (mg/dl)** | 41.90±8.44** | 27.45±2.18 | 51.80±2.33**** | 40.95±4.72** | 41.16±3.68** | 38.93±1.99* |
| **CHO (mg/dl)** | 102.70±4.49**** | 128.5±6.67 | 105.90±2.58*** | 119.40±9.03 | 110.1±3.994** | 106.2±6.45*** |
| **LDL (mg/dl)** | 56.24±4.48**** | 87.77±1.76 | 44.24±1.01**** | 64.45±3.74** | 57.31±1.33**** | 56.77±7.09**** |
| **TG (mg/dl)** | 47.27±3.80**** | 66.26±3.81 | 49.23±3.71**** | 53.18±5.85** | 55.93±3.88* | 50.73±4.14*** |

ALAT: Alanine transaminases; ASAT: Aspatate transaminases; GGT: Gamma glutamyl transferase; TG: Triglyseride; CHO: Cholesterol; HDL: high-density lipoprotein; LDL: Low-density lipoprotein.Values are expressed as mean ± SEM. Significance are determined with p values: *p < 0.05, **p < 0.01, ***p < 0.001, ****p < 0.0001.

**S8 Table:** *LC-MS/MS profiling of annotated compounds from Psidium guajava and Tithonia diversifolia extracts.*

| **No.** | **Compound Name** | **Retention Time (min)** | **Molecular Formula** | **Molecular Weight (g/mol)** | **Present In** | **Chemical Group** |
| --- | --- | --- | --- | --- | --- | --- |
| **1** | **Pedunculagin** | **6.958** | **C34H24O22** | **784.5** | ***P.guajava*** | **Tannins (ellagitannin)** |
| **2** | **Quercetin 4′-O-glucoside** | **14.439** | **C21H20O12** | **464.4** | ***T.diversifolia*** | **Flavonoids** |
| **3** | **Myricetin-3-O-pentoside** | **11.703** | **C20H18O12** | **450.3** | ***T.diversifolia*** | **Flavonoids** |
| **4** | **Quercetin** | **12.899** | **C15H10O7** | **302.2** | ***T.diversifolia*** | **Flavonoids** |
| **5** | **2′-Hydroxy-alpha-naphthoflavone** | **11.703** | **C19H12O3** | **288.3** | ***T.diversifolia*** | **Flavonoids** |

Compounds were putatively annotated by LC-MS/MS analysis of the aqueous extracts of Psidium guajava (bark) and Tithonia diversifolia (leaves) used in the present study. Retention times, molecular formulae, and molecular weights were derived from the acquired mass spectra. Compound classes (Group) are indicated for each annotated feature. Abbreviations: RT, retention time; MW, molecular weight.
