# Supplementary material for: A combined aqueous extract of Psidium guajava L. and Tithonia diversifolia (Hemsl.) A. gray improves metabolic homeostasis and preserves intestinal integrity in experimental rabbit colibacillosis
Source: PLoS One. 2026 Sep 15;21(9):e0357951. doi: 10.1371/journal.pone.0357951 (PMC13577389; doi:10.1371/journal.pone.0357951)
Supplement: Supplementary file 4 [file pone.0357951.s003.docx]

**Evaluation of** the manuscript entitled *“A Combined Aqueous Extract of Psidium guajava and Tithonia diversifolia Restores Metabolic Homeostasis and Intestinal Integrity in Experimental Rabbit Colibacillosis”*

**ID: PONE-D-26-07694**

The manuscript investigates the therapeutic efficacy of a combined herbal extract in an experimentally induced rabbit colibacillosis model. The study addresses an important topic regarding increased incidence of antimicrobial resistance and the search for alternative veterinary therapeutics.

The manuscript includes microbiological, biochemical, hematological, immunological, antioxidant, and histopathological evaluations. However, there are several major methodological and scientific concerns, particularly regarding the in vivo study design, characterization of the infection model, extract standardization, statistical analysis, and interpretation of the results.

**Major comments:**

A) **The rationale for choosing the infection model and experimental design**
1. The authors induced infection after administering both cyclophosphamide and streptomycin prior to E. coli inoculation. This creates a highly artificial model of disease but:
   **Are the observed pathological changes truly due to E. coli infection, or partially due to cyclophosphamide-induced immunotoxicity and streptomycin-induced dysbiosis?** Clearly explain the rationale for combining cyclophosphamide and streptomycin.
2. The manuscript includes an “immunosuppression control” group (T5), without mentioning a sufficient description of the treatment regimen: The authors should:

- Clarify whether T5 received cyclophosphamide alone or both cyclophosphamide and streptomycin,
- Provide complete data and discussion for T5.

3. The authors need to discuss the limitations of this highly manipulated infection model as cyclophosphamide itself can induce several effects like oxidative stress, hematological changes, and organ toxicity. Accordingly, several reported outcomes may not solely reflect colibacillosis.
B) **Lack of phytochemical characterization and extract standardization**

The major weakness of the study is the absence of phytochemical profiling and standardization of the two used extracts. The authors repeatedly referred the biological effects to the presence of flavonoids, tannins, and sesquiterpene lactones, with NF-κB modulation, without quantification and characterization of the present compounds using, for example, HPLC, LC-MS/MS, total phenolic/flavonoid quantification, or identification of the major compounds, including chromatographic fingerprinting. Otherwise, mechanistic claims should be toned down substantially.

C) **Small sample size and statistical power concerns**

The study used only **n = 5 rabbits/group**, with one mortality in the untreated infected group and only four animals/group ultimately analyzed histologically and biochemically. The endpoints measured are quite large in comparison with the number of animals per group. Accordingly, The authors should:

- provide statistical power justification,
- clarify whether normality testing was performed,
- explain how multiple testing inflation was controlled,
- and discuss the limitations imposed by the small sample size.

D) **Absence of quantitative histopathology evaluation**

The histopathological analysis is entirely descriptive mentioning “leukocyte infiltration, villous atrophy, restoration,” etc. However, no histological scoring system was used, and no semi-quantitative analysis was provided. The authors should: introduce validated histopathological scoring systems, quantify inflammatory infiltration and villus height/crypt depth, and provide lesion severity scores.

E) **Cytokine and mechanistic claims are overstated**

The authors repeatedly conclude that the extracts used caused NF-κB inhibition, immunomodulation, ROS scavenging mechanisms, membrane stabilization, and bacterial adhesion inhibition, without experimental evaluation of these pathways. Only general screening parameters were measured. Mechanistic statements should be rewritten more cautiously and presented as hypotheses rather than demonstrated mechanisms.

For example: “may involve modulation of inflammatory pathways”

instead of: “likely mediated through NF-κB inhibition.”

F) **Important missing methodological details**

Several experimental details are insufficiently described, including:

1. **Randomization:** The manuscript states rabbits were “randomly assigned,” but the randomization method is not described.
2. **Blinding:** No mention of blinding during treatment administration, histological evaluation, or data analysis.
3. **Sex distribution:** The rabbits were “mixed sex,” but the distribution per group was not provided. Sex-dependent differences may influence immune response.
4. **Extract formulation:** The exact ratio between the two combined extracts is not clearly stated.
5. **Dose justification:** The rationale for the selected doses is not mentioned
G) **Bacterial characterization is insufficient**

The isolate was identified only by API 20E and Congo red binding assay. However, the study repeatedly refers to EPEC, virulence, pathogenicity, attaching/effacing lesions.

No molecular confirmation of virulence genes was performed. The authors should avoid definitive classification as EPEC unless molecular virulence markers are confirmed,
or include PCR confirmation for relevant virulence genes.

H) **PCA and multivariate analysis appear overinterpreted**

The PCA and heatmap analyses are visually attractive, but the sample size is extremely small, the number of variables is very large, and no validation strategy is described. Statements such as: “biological reset” and “systemic recovery indistinguishable from healthy baseline” are exaggerated considering the exploratory nature of PCA.

The authors should moderate interpretation, clarify variance explained, and discuss limitations of multivariate modeling with small datasets.

I) **Discussion parts after each results section need references and referral to the literature**

The discussion following several results sections lacks sufficient referencing and critical comparison with the literature. The authors should discuss their findings in relation to previous studies, emphasizing agreements, discrepancies, possible explanations, and remaining research gaps. For example, *Psidium* species records indicate antidiarrheal activity through antimicrobial effects, not only for tannins (DOI: https://doi.org/10.1016/j.jep.2025.119670)

**Minor Comments**

1. **Several sections contain overly promotional language inappropriate for scientific writing, including:**

- “biological reset,”
- “high-activity extract,”
- “profound therapeutic trajectory,”
- “gold-standard treatment,”
- “extinguishing inflammatory cascades.”

The manuscript should include a more objective scientific tone.

2. **The discussion is excessively long and repetitive. Several statements restate results instead of critically interpreting them** (back to **comment no. I** in the major comments).
3. **The authors should clarify whether** ALT and AST units, cytokine units, and oxidative stress marker units, were standardized and validated for rabbit samples (ensure the kits are valid for rabbit samples).
4. **Figure legends appear insufficiently detailed.** They should be revised to be self-interpreted in some sections through including:

- magnification,
- stain details,
- scale bars,
- and statistical annotations.

5. **The ARRIVE guideline adherence is mentioned, but several ARRIVE-required elements remain incomplete:**

- sample size justification,
- exclusion criteria,
- blinding details.

6. **The conclusion somewhat overstates translational applicability.** The statement that the extract is “clinically equivalent to conventional aminoglycosides” is not fully supported without pharmacokinetics, toxicity studies, dose optimization, long-term safety, and field validation. Accordingly, the conclusion must be revised.

The work should not be accepted in its current form, but it may become suitable for publication after major methodological clarification, further specific analysis, and thorough revision of the interpretation and presentation of the data.
