# Supplementary material for: A combined aqueous extract of Psidium guajava L. and Tithonia diversifolia (Hemsl.) A. gray improves metabolic homeostasis and preserves intestinal integrity in experimental rabbit colibacillosis
Source: PLoS One. 2026 Sep 15;21(9):e0357951. doi: 10.1371/journal.pone.0357951 (PMC13577389; doi:10.1371/journal.pone.0357951)
Supplement: Supplementary file 10003 [file pone.0357951.s002.pdf]

PLOS One

A Combined Aqueous Extract of Psidium guajava and Tithonia diversifolia Restores Metabolic Homeostasis and Intestinal Integrity in Experimental Rabbit Colibacillosis

--Manuscript Draft--

Manuscript Number:	PONE-D-26-07694
Article Type:	Research Article
Full Title:	A Combined Aqueous Extract of Psidium guajava and Tithonia diversifolia Restores Metabolic Homeostasis and Intestinal Integrity in Experimental Rabbit Colibacillosis
Short Title:	Natural Products Restore Metabolic Homeostasis and Intestinal Integrity in Experimental Rabbit Colibacillosis
Corresponding Author:	Fabrice Fekam Boyom, Ph.D.<br>University of Yaounde I Faculty of Sciences: Universite de Yaounde I Faculte des Sciences<br>Yaounde, CAMEROON
Keywords:	Escherichia coli, Colibacillosis, Antimicrobial Resistance, Psidium guajava, Tithonia diversifolia, Rabbits
Abstract:	Background<br>Colibacillosis, caused by pathogenic Escherichia coli, remains a significant threat to rabbit production, particularly during the critical weaning period. The superbugs' resistivity against conventional drugs severely limits conventional treatment options. Therefore, there is an urgent need for high-activity biological alternatives. This study evaluated the therapeutic efficacy of a combined aqueous leaf extract of Psidium guajava and Tithonia diversifolia on systemic biological markers and organ integrity in an experimental rabbit model of colibacillosis.<br>Methods<br>Thirty healthy rabbits were assigned to six cohorts, including a negative control (infected-untreated), a healthy control (Uninfected-Untreated), a standard drug control (infected and treated [Neomycin 10 mg/kg]), immunodepressed, and two treatment groups receiving the polyherbal extract (400 and 800 mg/kg). Colibacillosis was induced via oral administration of a clinical isolate of E. coli (10^9 CFU/mL). Systemic recovery was assessed through haematological, biochemical, and antioxidant profiling, alongside histological examination and multivariate analysis (PCA and Hierarchical Clustering).<br>Results<br>E. coli infection induced a profound haematology, biochemical, and inflammatory state, characterized by significant increases in WBC counts, AST, creatinine, and triglycerides ($P < 0.05$), with a concomitant decline in RBCs, haemoglobin, and albumin. Treatment with the combined extract demonstrated dose-dependent efficacy. Notably, multivariate PCA mapping revealed that the 800 mg/kg dose achieved a superior therapeutic trajectory, successfully restoring the biological signature to a state comparable to the Neomycin and Normal groups. This extract dose effectively resolved diarrhoea, cleared bacterial load, and mitigated oxidative stress and organ damage.<br>Conclusion<br>The P. guajava and T. diversifolia combination possesses potent antimicrobial, anti-inflammatory, and antioxidant properties. These findings validate its traditional use and support its potential as a robust natural alternative to conventional antibiotics for managing colibacillosis in infected-rabbits.
Order of Authors:	Mireille Tuedom Chouegouong
	Rufin Marie Toghueo Kouipou, Ph.D.
	Yanick Kevin Melogmo Dongmo
	Jaurès Marius Tsakem Nangap
	Michele Stella Majoumouo, Ph.D.
	Brice Pinlap

Powered by Editorial Manager® and ProduXion Manager® from Aries Systems Corporation

	Kingsley Agbor Etchu
	Fabrice Fekam Boyom, Ph.D.
**Additional Information:**	
**Question**	**Response**
**Financial Disclosure**<br><br>Enter a financial disclosure statement that describes the sources of funding for the work included in this submission. Review the submission guidelines for detailed requirements. View published research articles from *PLOS ONE* for specific examples.<br><br>This statement is required for submission and **will appear in the published article** if the submission is accepted. Please make sure it is accurate.<br><br>**Funded studies**<br>Enter a statement with the following details:<br>• Initials of the authors who received each award<br>• Grant numbers awarded to each author<br>• The full name of each funder<br>• URL of each funder website<br>• Did the sponsors or funders play any role in the study design, data collection and analysis, decision to publish, or preparation of the manuscript?<br><br>Did you receive funding for this work?	The author(s) received no specific funding for this work.
**Competing Interests**<br><br>Use the instructions below to enter a competing interest statement for this submission. On behalf of all authors, disclose any competing interests that could be perceived to bias this work—acknowledging all financial support and any other relevant financial or non-financial competing interests.<br><br>This statement is required for submission and **will appear in the published article** if	The authors have declared that no competing interests exist.

the submission is accepted. Please make sure it is accurate and that any funding sources listed in your Funding Information later in the submission form are also declared in your Financial Disclosure statement.<br><br>View published research articles from *PLOS ONE* for specific examples.<br><br>**NO authors have competing interests**<br><br>Enter: *The authors have declared that no competing interests exist.*<br><br>**Authors with competing interests**<br><br>Enter competing interest details beginning with this statement:<br><br>*I have read the journal's policy and the authors of this manuscript have the following competing interests: [insert competing interests here]*<br><br>* typeset	
**Ethics Statement**<br><br>Enter an ethics statement for this submission. This statement is required if the study involved:<br><br>• Human participants<br>• Human specimens or tissue<br>• Vertebrate animals or cephalopods<br>• Vertebrate embryos or tissues<br>• Field research<br><br>Write "N/A" if the submission does not require an ethics statement.<br><br>General guidance is provided below. Consult the submission guidelines for detailed instructions. **Make sure that all information entered here is included in the Methods section of the manuscript.**	All procedures were approved by the Cameroon National Ethical Committee (Ref N. FWIRB00001954).

**Format for specific study types**<br><br>**Human Subject Research (involving human participants and/or tissue)**<br>• Give the name of the institutional review board or ethics committee that approved the study<br>• Include the approval number and/or a statement indicating approval of this research<br>• Indicate the form of consent obtained (written/oral) or the reason that consent was not obtained (e.g. the data were analyzed anonymously)<br><br>**Animal Research (involving vertebrate animals, embryos or tissues)**<br>• Provide the name of the Institutional Animal Care and Use Committee (IACUC) or other relevant ethics board that reviewed the study protocol, and indicate whether they approved this research or granted a formal waiver of ethical approval<br>• Include an approval number if one was obtained<br>• If the study involved *non-human primates*, add *additional details* about animal welfare and steps taken to ameliorate suffering<br>• If anesthesia, euthanasia, or any kind of animal sacrifice is part of the study, include briefly which substances and/or methods were applied<br><br>**Field Research**<br><br>Include the following details if this study involves the collection of plant, animal, or other materials from a natural setting:<br>• Field permit number<br>• Name of the institution or relevant body that granted permission	
**Data Availability**<br><br>Authors are required to make all data underlying the findings described fully available, without restriction, and from the time of publication. PLOS allows rare exceptions to address legal and ethical concerns. See the PLOS Data Policy and FAQ for detailed information.	Yes - all data are fully available without restriction

A Data Availability Statement describing where the data can be found is required at submission. Your answers to this question constitute the Data Availability Statement and **will be published in the article**, if accepted.<br><br>**Important:** Stating 'data available on request from the author' is not sufficient. If your data are only available upon request, select 'No' for the first question and explain your exceptional situation in the text box.<br><br>Do the authors confirm that all data underlying the findings described in their manuscript are fully available without restriction?	
**Describe where the data may be found in full sentences. If you are copying our sample text, replace any instances of XXX with the appropriate details.**<br><br>• If the data are **held or will be held in a public repository**, include URLs, accession numbers or DOIs. If this information will only be available after acceptance, indicate this by ticking the box below. For example: *All XXX files are available from the XXX database (accession number(s) XXX, XXX.*).<br>• If the data are all contained **within the manuscript and/or Supporting Information files**, enter the following: *All relevant data are within the manuscript and its Supporting Information files.*<br>• If neither of these applies but you are able to provide **details of access elsewhere**, with or without limitations, please do so. For example:<br><br>*Data cannot be shared publicly because of [XXX]. Data are available from the XXX Institutional Data Access / Ethics Committee (contact via XXX) for researchers who meet the criteria for access to confidential data.*<br><br>*The data underlying the results presented in the study are available from (include the name of the third party*	All relevant data are within the manuscript and its Supporting Information files.

and contact information or URL).<br>• This text is appropriate if the data are owned by a third party and authors do not have permission to share the data.<br><br>* typeset	
Additional data availability information:	

Manuscript

Click here to access/download;Manuscript;PONE-D-26-07694_Clean_100326.doc

A Combined Aqueous Extract of *Psidium guajava* and *Tithonia diversifolia* Restores Metabolic Homeostasis and Intestinal Integrity in Experimental Rabbit Colibacillosis

Mireille Tuedom Chouegouong[1,2], Rufin Marie Kouipou Toghueo[1,3], Yanick Kevin Melogmo Dongmo[1], Jaurès Marius Tsakem Nangap[4], Michele Stella Majoumouo[1,5], Brice Pinlap[1] Kingsley Agbor Etchu[2], Fabrice Fekam Boyom[1]

[1]Antimicrobial and Biocontrol Agents Unit (AmBcAU), Laboratory for Phytobiochemistry and Medicinal Plants Studies, Department of Biochemistry, Faculty of Science, University of Yaoundé I, P.O. Box 812, Messa-Yaoundé, Cameroon

[2]Institute of Agricultural Research and Development (IRAD), P.O. Box 2067, Yaoundé, Cameroon

[3]Laboratory for Antibiotic Resistance and Microbial Metabolomics, Institute of Microbiology, Czech Academy of Sciences, Videnska 1083, 142 20 Prague 4, Czech Republic

[4]Laboratory of Animal Physiology, Department of Animal Biology and Physiology, Faculty of Science, University of Yaoundé 1, PO Box 812, Yaoundé, Cameroon

[5]Division of Pulmonary, Critical Care & Sleep Medicine, Department of Internal Medicine, University of Cincinnati College of Medicine

Abstract

Background

Colibacillosis, caused by pathogenic *Escherichia coli*, remains a significant threat to rabbit production, particularly during the critical weaning period. The superbugs' resistivity against conventional drugs severely limits conventional treatment options. Therefore, there is an urgent need for high-activity biological alternatives. This study evaluated the therapeutic efficacy of a combined aqueous leaf extract of *Psidium guajava* and *Tithonia diversifolia* on systemic biological markers and organ integrity in an experimental rabbit model of colibacillosis.

Methods

Thirty healthy rabbits were assigned to six cohorts, including a negative control (infected-untreated), a healthy control (Uninfected-Untreated), a standard drug control (infected and treated [Neomycin 10 mg/kg]), immunodepressed, and two treatment groups receiving the polyherbal extract (400 and 800 mg/kg). Colibacillosis was induced via oral administration of a clinical isolate of *E. coli* (10^9 CFU/mL). Systemic recovery was assessed through haematological, biochemical, and antioxidant profiling, alo[illegible]de histological examination and multivariate analysis (PCA and Hierarchical Clustering).

Results

E. coli infection induced a profound haematology, biochemical, and inflammatory state, characterized by significant increases in WBC counts, AST, creatinine, and triglycerides (P <0.05), with a concomitant decline in RBCs, haemoglobin, and albumin. Treatment with the combined extract demonstrated dose-dependent efficacy. Notably, multivariate PCA mapping

revealed that the 800 mg/kg dose achieved a superior therapeutic trajectory, successfully restoring the biological signature to a state comparable to the Neomycin and Normal groups. This extract dose effectively resolved diarrhoea, cleared bacterial load, and mitigated oxidative stress and organ damage.

Conclusion

The *P. guajava* and *T. diversifolia* combination possesses potent antimicrobial, anti-inflammatory, and antioxidant properties. These findings validate its traditional use and support its potential as a robust natural alternative to conventional antibiotics for managing colibacillosis in infected-rabbits.

Keywords: *Escherichia coli*, Colibacillosis, Antimicrobial Resistance, *Psidium guajava*, *Tithonia diversifolia*, Rabbits

Introduction

In the face of escalating global climate change and the urgent need for sustainable food systems, rabbit farming (*Oryctolagus cuniculus*) has emerged as an environmentally superior alternative to traditional livestock [1, 2]. Characterized by a low carbon footprint, minimal water requirements, and the ability to thrive on non-competitive forage, the domestic rabbit industry is increasingly recognized as a cornerstone of eco-friendly agriculture and a vital contributor to rural economic development and food security worldwide [2]. Global rabbit meat production reached approximately 860,000 tons in 2021, with China leading the sector (53.1%), followed by a growing importance in developing nations [3].This prominence is driven by the rabbit's favorable biological profile, including high fecundity and efficient feed conversion [4], producing a highly nutritious, lean protein source rich in essential micronutrients like iron, zinc, and vitamin B12 [5].

Despite this potential, the rabbit farming industry faces substantial health challenges, with enteric diseases representing the most economically devastating hurdle [6]. Infectious diarrhoea remains a primary cause of mortality and reduced growth performance, leading to high veterinary costs [7]. Colibacillosis, caused by enteropathogenic *Escherichia coli* (EPEC), stands out as the most prevalent bacterial threat to young rabbits [6]. The pathogenesis of EPEC is defined by a sophisticated cytokine cascade: bacterial attachment (BFP) and effector protein injection (T3SS) trigger an immediate innate response (IL-1β, IL-6), which subsequently activates a Th1-mediated adaptive immune response [8]. The resulting attaching and effacing (A/E) lesions destroy the intestinal brush border, compromising the epithelial barrier and allowing systemic cytokines to exacerbate fluid loss and diarrhoea [8]. Furthermore, this bacterial colonization triggers an intense oxidative stress response. The host's ability to survive this oxidative storm depends heavily on the mobilization of its antioxidant defense system, particularly superoxide dismutase (SOD) and glutathione peroxidase (GPX) [9].

The conventional management of colibacillosis has historically relied on a narrow spectrum of antimicrobials, predominantly trimethoprim-sulfamethoxazole and fluoroquinolones [10].

However, the livestock sector is now at a critical juncture due to the global escalation of Antimicrobial Resistance (AMR). Recent surveillance indicates a dramatic rise in multi-drug resistant (MDR) *E. coli* strains in rabbitries, with some isolates exhibiting total resistance to 'watch-list' antibiotics like tetracyclines and aminoglycosides [11, 12]. This phenomenon is not merely a veterinary concern but a significant public health threat, as the transmission of resistant genes from livestock to the human food chain complicates the treatment of enteric infections in people [13, 14]. Furthermore, the increasing regulatory restrictions on antibiotic growth promoters (AGPs) and the growing consumer demand for 'antibiotic-free' animal products have rendered the status quo untenable [15, 16]. Consequently, there is an urgent imperative to transition toward sustainable, bio-based therapeutic alternatives that can achieve clinical resolution without contributing to the environmental resistome.

Ethnoveterinary medicine offers a robust reservoir for therapeutic discovery, particularly through species such as *Psidium guajava* L. (Myrtaceae) and *Tithonia diversifolia* (Hemsl.) A. Gray (Asteraceae). While *P. guajava* is globally utilized for its tannin-rich antidiarrheal properties [17] and *T. diversifolia* for its anti-inflammatory sesquiterpene lactones [18], their selection for this study was predicated on the results from our previous investigation. In our preceding ethnopharmacological survey of rabbitries in Cameroon, these two species emerged as the most prominent remedies used by local farmers to manage bacterial enteritis [19]. Subsequent *in vitro* screening of forty-five different preparations revealed that only six extracts, exhibited potent antibacterial activity against enteropathogenic *Escherichia coli* (EPEC). Crucially, the combination of *T. diversifolia* and *P. guajava* yielded a profound synergistic interaction, achieving a Fractional Inhibitory Concentration Index (FICI) as low as 0.265 and reducing individual MIC values by up to 64-fold [19]. This specific formulation demonstrated a high biological potency index, consistently maintaining activity levels that justify its further investigations. Consequently, the present study was designed to validate these synergistic findings *in vivo*, utilizing an integrated multivariate approach to determine if this polyherbal extract can resolve systemic colibacillosis and restore host homeostasis as effectively as conventional antibiotherapy.

Materials and Methods

Ethical approval, Ethical Statement and Animal Welfare

All experimental protocols were reviewed and approved by the Cameroon National Ethical Committee (Ref No. FWIRB00001954). The study was conducted in strict accordance with the ARRIVE guidelines (Animal Research: Reporting of In Vivo Experiments).

Ethnobotanical Selection and Extract Preparation

The selection of plant materials was based on our previous ethnopharmacological survey identifying highly active remedies against rabbit bacterial enteritis [19]. Fresh leaves of *Psidium guajava* L. (Myrtaceae) and *Tithonia diversifolia* (Hemsl.) A. Gray (Asteraceae) were collected in Yaoundé, Cameroon, and authenticated at the National Herbarium of Cameroon (Voucher Nos. 2884 SRFK and 57410 HNC, respectively).

Leaves were air-dried at room temperature (25 °C) for 14 days and pulverized. To maintain consistency with traditional preparation methods while ensuring high extraction yield, aqueous extracts were prepared using cold extraction. For each species, 500 g of powder was macerated in 2 L of distilled water for 72 h under continuous agitation. The mixtures were filtered through hydrophilic cotton, and the filtrates were dehydrated under forced ventilation at 25°C. The resulting crude extracts were stored at -20°C.

Characterization and Virulence of the Clinical Isolate

A clinical isolate of *Escherichia coli* was recovered from a rabbit presenting acute colibacillosis symptoms. Identification was confirmed using the API 20E analytical profile index (bioMérieux, France). Virulence potential was assessed via the Congo red binding assay [30] on tryptic soy agar supplemented with 0.003 % Congo red and 1.5 % bile salts. Red pigmented colonies, indicative of virulent plasmid expression, were selected and cultured in nutrient broth supplemented with bile salt (0.4%). Inoculum turbidity from a 24-hour culture was adjusted to 4 McFarland, corresponding to a concentration of approximately 10^9 CFU/mL in sterile 0.9 % saline [31].

Experimental Animals

Thirty hybrid rabbits (*mixed sex*, 6–7 weeks old, weighing 800 to 900 g) were acclimatized for 7 days at the Laboratory Phytobiochemistry and Medicinal Plant studies of the University of Yaoundé I. Animals were housed in pairs in galvanized cages (35 cm × 40 cm × 60 cm (width × height × length)) under a 12h light/dark cycle with *ad libitum* access to a standard pelleted diet and water.

Experimental Design and Induction of Colibacillosis

To facilitate enteric colonization, rabbits underwent a preliminary dysbiosis protocol: immunosuppression via cyclophosphamide (30 mg/kg of bw, i.p.) and intestinal microbiota clearing via streptomycin (200 mg/kg of bw, p.o.) 48 hours before infection. Following this, colibacillosis was induced by oral gavage of 1 mL of the 10^9 CFU *E. coli* suspension (in 0.9% saline).

Rabbits were randomly assigned to six groups (n=5): T0: Normal Control (non-infected, sterile water); T1: Negative Control (infected, untreated); T2: Positive Control (infected, Neomycin 10 mg/kg p.o. for 5 days); T3: Low Dose (infected, 400 mg/kg polyherbal extract p.o.); T4: High Dose (infected, 800 mg/kg polyherbal extract p.o.); T5: Immunosuppression control. Treatments were daily administered (10 mL/kg bwt/dose) for 7 days, starting 24 hours post-infection (PI), once the colibacillosis appeared.

Clinical and Microbiological Monitoring

Daily faecal samples were collected to quantify bacterial shedding. Stool samples (10 mg) were serially diluted (10^{-5}) in sterile saline and plated on MacConkey agar. After 24 h incubation at 37°C, typical *E. coli* colonies were counted and expressed as Log_{10} CFU/g of stool. Growth performance was monitored by weighing rabbits every two days.

Ethical Considerations and Humane Endpoints

Because the induction of acute colibacillosis carries a risk of severe morbidity, a rigorous clinical monitoring protocol was established. Rabbits were monitored three times daily (every 8 hours) by trained personnel for clinical signs of distress. Humane endpoints were strictly defined as: (i) loss of >20% of initial body weight; (ii) severe lethargy or inability to access food/water; (iii) persistent recumbency or lack of righting reflex; (iv) labored or agonal breathing; and (v) hypothermia or severe dehydration (skin tent >2 seconds).

Any animal meeting these criteria was designated for immediate rescue euthanasia to minimize pain and distress. In this study, only one animal in the T1 (Negative Control) group reached humane endpoints on Day 3 post-infection due to severe dehydration and was euthanized immediately. No animals died spontaneously during the experimental period.

Anesthesia and Methods of Sacrifice

On Day 7 post-infection (PI), a total of 24 surviving rabbits (n = 4 per group) were humanely sacrificed for sample collection. The remaining five healthy animals from the treatment groups were excluded from terminal sampling and were maintained under standard husbandry conditions for continued post-experimental monitoring. To ensure animal welfare and data integrity, rabbits were moved to a separate, quiet procedure room. Animals were fasted for 12 hours prior to euthanasia to reduce anesthesia-related complications. Deep surgical anesthesia was induced via intramuscular injection of ketamine (30 mg/kg) combined with diazepam (10 mg/kg). The depth of anesthesia was strictly verified by the complete abolition of the pedal withdrawal and corneal reflexes before any procedures commenced. Euthanasia was completed under deep anesthesia via cervical dislocation (for rabbits under 1 kg) or exsanguination via cardiac puncture, ensuring a rapid and painless death in accordance with AVMA Guidelines for the Euthanasia of Animals.

Sample Collection, Systemic Antioxidant and Histopathological Analysis

~~Immediately after euthanasia~~, whole blood was collected for hematology (EDTA tubes) and serum biochemistry (dry tubes). Hematological parameters included WBC, RBC, HGB, HCT, and leukocyte differentials, analyzed using a Mindray BC-5300 automated analyzer. Serum biochemistry included ALT, AST, GGT, Creatinine, Uric Acid, Total Protein, and lipid profile (TC, TG, HDL), analyzed using commercial kits (LABKIT, France). Cytokine levels (TNF-α, IL-1β, IL-6, IL-10, CRP) were quantified via sandwich ELISA (Quantikine® Rabbit ELISA kits, Sigma-Aldrich).

Tissue samples from the ileum and caecum, liver, and kidney were collected post-mortem for antioxidant assays and histopathological analysis. Ileum and caecum segments were homogenized in 50 mM Tris-HCl buffer (pH 7.4) and centrifuged at 1500 g for 25 min. Supernatants were analyzed for MDA, GSH, SOD, and Catalase. Liver, kidney, and caecum sections were fixed in 10% buffered formalin, embedded in paraffin, sectioned at 5 µm, and stained with hematoxylin and eosin (H&E) for microscopic examination. After all sample collection, rabbit carcasses were autoclaved prior to disposal in compliance with biosafety regulations.

Advanced Statistical and Multivariate Analysis

Data were expressed as Mean ± SEM. Statistical significance was determined by one-way ANOVA followed by Fisher's LSD post-hoc test ($P \leq 0.05$) using OriginLab Pro 2025 and GraphPad Prism 8.0.1. To evaluate the holistic therapeutic trajectory of the rabbits, an integrated multivariate approach was employed. A Principal Component Analysis (PCA) was performed to reduce the dimensionality of the biological dataset (comprising growth, microbiological, biochemical, and immunological parameters). Data were auto-scaled (mean-centered and divided by the standard deviation) to account for differences in units and magnitudes. This enabled the visualization of the spatial coordinates of each treatment cohort and the identification of the primary biological drivers of recovery. A global biological fingerprint was generated using hierarchical clustering. The heatmap was constructed based on Z-score-normalized data to visualize the intensity of the inflammatory, oxidative, and metabolic shifts across groups. Clustering was performed using the Euclidean distance and Ward's linkage method. To assess the clinical non-inferiority of the polyherbal extract compared to the reference antibiotic, a direct comparison between the 800 mg/kg dose and Neomycin (10 mg/kg) was conducted. Statistical parity was defined by the absence of significant divergence ($P > 0.05$) in recovery endpoints across faceted biological systems. All multivariate analyses and visualizations were performed in the R statistical environment (v4.4.3) using the tidyverse for data manipulation, FactoMineR and factoextra for multivariate modelling, and ggplot2 (ggrepel) for high-resolution, colour-blind friendly graphical representation.

Results

Biochemical identification of *E. coli* isolates and virulence testing

The clinical isolate recovered from symptomatic rabbits was subjected to standardized biochemical characterization using the API 20E system (bioMérieux, Marcy l'Etoile, France). The isolate exhibited characteristic carbohydrate fermentation patterns, including positive reactions for glucose, sorbitol, and arabinose, while remaining negative for urease and H_2S production. The resulting biochemical profile confirmed the identity of the pathogen as *Escherichia coli* with an identification probability of (API 20E profile 5044572; identification quality: very good; probability: 100% *E. coli* 1), validating its use for the subsequent experimental infection model (Fig. 1, Suppl Table 1). The virulence potential of the *E. coli* isolate was phenotypically confirmed via the Congo red binding assay. The isolate exhibited strong dye uptake, resulting in distinct, red-pigmented colonies (CR+) after 24 hours of incubation (Suppl Fig. 1). This positive phenotype indicates the presence of virulence-associated surface proteins and curli fimbriae, validating the isolate's capacity for host colonization and its suitability for inducing experimental colibacillosis.

Effects of the combination of *Psidium guava* and *Tithonia diversifolia* extracts on the body weight of experimentally infected rabbits

The ~~impact~~ of *E. coli* infection and subsequent polyherbal intervention on the growth trajectory of rabbits is presented in Fig. 2 (Suppl Table 2). Following the experimental challenge, a significant divergence in body weight was observed between the study cohorts.

The infected, untreated group (T1) exhibited a sharp and persistent decline in body weight throughout the 7-day post-infection (PI) period. This pathological weight loss is associated with severe malabsorption and dehydration from infectious diarrhoea.

In contrast, rabbits treated with the combined extracts of *P. guajava* and *T. diversifolia* showed a notable reversal of this trend. From Day 3 PI onward, the 800 mg/kg dose (T4) effectively halted weight loss and initiated a phase of compensatory growth, resulting in a weight gain profile that mirrored the Neomycin-treated (T2) and healthy control (T0) groups ($P < 0.05$). While the 400 mg/kg dose (T3) provided moderate protection against growth retardation, the 800 mg/kg dose demonstrated superior efficacy in restoring the rabbits to their pre-infection growth curve. These results suggest that the polyherbal extract not only resolves the core infection but also facilitates rapid physiological recovery and nutrient assimilation.

Effect of the combination of *Psidium guava* and *Tithonia diversifolia* extracts on the relative weight of some internal organs

The macroscopic impact of *E. coli* infection and the safety profile of the polyherbal intervention were evaluated through relative organ weight analysis and visual inspection (Fig. 3). Upon necropsy, all treatment groups exhibited normal organ coloration and morphology, with no visible lesions or congestion in the liver, kidneys, or heart. Statistical analysis revealed that the relative weights of the kidneys and liver in both the 400 and 800 mg/kg extract groups showed no significant variation ($P > 0.05$) when compared to the healthy control (T0) and the Neomycin-treated (T2) groups.

This lack of organ hypertrophy or atrophy is a critical indicator of the extract's safety, suggesting an absence of sub-acute toxicity at these therapeutic doses. Notably, the maintenance of normal liver and spleen weights in the 800 mg/kg cohort (T4) contrasts with the inflammatory oedema and reactive splenomegaly typically observed in untreated colibacillosis. While the infected-untreated group (T1) showed tendencies toward increased relative spleen weight consistent with the physiological demand of filtering bacterial debris, immune activation treatment with the high-activity extract effectively preserved organ homeostasis. These results, coupled with the biochemical data, confirm that the *P. guajava* and *T. diversifolia* combination provides systemic protection against associated infection effects without inducing adverse pathological changes in vital metabolic organs.

Therapeutic Efficacy and *in vivo* bacterial clearance of the combination of *Psidium guajava* and *Tithonia diversifilia*

The *in vivo* antibacterial potency of the *P. guajava* and *T. diversifolia* combination was quantified by monitoring the temporal dynamics of *E. coli* shedding in the faeces (Fig. 4, Suppl Table 3). Bacterial load is a critical indicator of the extract's ability to disrupt intestinal colonization and prevent further mucosal invasion. Following the experimental challenge, all infected groups exhibited peak bacterial shedding (10^9 CFU/10 mg stool) prior to the initiation of therapy.

Upon treatment, a progressive, time-dependent reduction in the bacterial burden was observed across all intervention groups. While the reference antibiotic (Neomycin, T2) achieved

sterilized stool samples within 72 hours (Day 3 of treatment), the polyherbal extracts demonstrated robust curative activity. Specifically, the 800 mg/kg dose (T4) and the 400 mg/kg dose (T3) facilitated complete pathogen eradication by the fifth day of administration. This 5-day clearance window is clinically significant, as it correlates with the rapid resolution of diarrheic symptoms and the reversal of weight loss observed in the growth performance data (Fig. 2).

These findings suggest that the combined extract acts as a potent luminal antimicrobial agent, effectively neutralizing enteropathogenic *E. coli* within the complex gastrointestinal environment. By significantly reducing the duration of bacterial shedding, the 800 mg/kg dose minimizes the risk of environmental re-contamination and horizontal transmission within rabbitries, positioning this polyherbal formulation as a viable biological alternative for the management of clinical colibacillosis.

The effect of combined aqueous extract treatment on the Restoration of Intestinal Redox Homeostasis and Antioxidant Defense

The impact of *E. coli* infection and subsequent polyherbal intervention on the intestinal redox landscape is illustrated in Fig. 5 (Suppl Table 4). In the infected-untreated cohort (T1), experimental colibacillosis induced a state of biochemical collapse within both the caecum and ileum. This pathological state was characterized by a significant spike ($P < 0.05$) in malondialdehyde (MDA) levels linked to extensive lipid peroxidation, coinciding with a dramatic depletion of the primary antioxidant defenses, including Superoxide Dismutase (SOD), Catalase (CAT), and reduced Glutathione (GSH). These findings confirm that *E. coli* colonization rapidly exhausts the host's local antioxidant protection, leading to oxidative tissue damage.

Therapeutic administration of the *P. guajava* and *T. diversifolia* extract effectively reversed this oxidative trend in a dose-dependent manner. While the 400 mg/kg dose (T3) provided moderate protection, the 800 mg/kg dose (T4) achieved a superior restorative profile, returning MDA, CAT, and SOD levels to values statistically indistinguishable from the healthy control (T0).

~~Crucially,~~ in specific parameters such as caecal GSH and ileal SOD, the 800 mg/kg polyherbal treatment maintained the antioxidant levels that were numerically superior to the Neomycin standard (T2). This highlights a key therapeutic advantage: while Neomycin serves as a potent bactericidal agent, the polyherbal extract provides a dual-action benefit by simultaneously eradicating the pathogen and actively repairing the intestinal antioxidant framework. This neutralization of Reactive Oxygen Species (ROS) likely underpins the structural recovery of the intestinal mucosa observed in our histological analysis.

The effect of combined aqueous extract treatment on haematological stabilization and resolution of systemic inflammation

The systemic impact of the *E. coli* challenge and the subsequent restorative effects of the *P. guajava* and *T. diversifolia* combination are summarized in the haematological profiles (Figure 6; Suppl Table 5). The infected-untreated group (T1) exhibited a classic septic hemogram,

characterized by profound leukocytosis and thrombocytosis. Specifically, White Blood Cell (WBC) counts nearly doubled and Platelet (PLT) counts nearly tripled compared to the healthy control (T0), signalling a severe systemic inflammatory response and the activation of the host's emergency myeloid defense against *E. coli* septicemia.

Simultaneously, the T1 cohort suffered from anaemia of infection, evidenced by significant declines ($P < 0.05$) in Red Blood Cell (RBC) counts, Haemoglobin (HGB) levels, and Haematocrit (HCT) compared to all other groups. This erythrocytic collapse likely reflects the combined effects of bacterial hemolysins, iron sequestration, and the haemorrhagic nature of the colibacillosis-induced intestinal lesions.

Treatment with the polyherbal extract achieved near-complete normalization of the hemogram in a dose-dependent manner. The 800 mg/kg dose (T4) proved to be particularly effective, successfully extinguishing the systemic inflammatory response by returning WBC and PLT counts to baseline levels. Furthermore, the high-activity extract completely reversed the erythrocytic decline, restoring haemoglobin concentrations from a pathological low of approximately 11 g/dL to a healthy 15 g/dL. Notably, the leukocyte differential (GRA%, LYM%, and MID%) was rebalanced in the T4 group, indicating a restored and functional immune landscape comparable to the neomycin-treated (T2) and healthy (T0) cohorts. These findings underscore the extract's potent hematoprotective efficacy and its ability to halt the progression of the infection from a local gut disturbance to a systemic crisis.

Immunomodulatory effects of combined aqueous extract treatment on *E. coli*-infected rabbits

The immunological response to *E. coli* infection and the restorative capacity of the polyherbal intervention were evaluated through key pro-inflammatory and anti-inflammatory biomarkers (Figure 7; Suppl Table 6). In the infected-untreated cohort (T1), experimental colibacillosis triggered a massive cytokine production, evidenced by significant elevations ($P < 0.05$) in C-reactive protein (CRP), TNF-α, IL-1β, and IL-6. This excessive release of innate first-responder cytokines drives the systemic inflammatory response, leading to tissue damage and metabolic exhaustion observed in the T1 group.

Oral administration of the *P. guajava* and *T. diversifolia* combination at 400 and 800 mg/kg induced a profound, significant reduction in these pro-inflammatory markers. Notably, for critical indicators like TNF-α and IL-6, the 800 mg/kg dose (T4) was more potent, suppressing cytokine levels as effectively as the Neomycin standard (T2). This suggests that the bioactive metabolites in the extract, such as flavonoids and sesquiterpene lactones, likely modulate the underlying signalling pathways (such as NF-kB) responsible for acute inflammatory cascades.

Conversely, the anti-inflammatory cytokine IL-10 showed a distinct and clinically relevant pattern. While IL-10 levels were significantly lower in the untreated group (T1), all treated cohorts (T2, T3, T4) maintained significantly higher concentrations of this cytokine ($P < 0.05$). This indicates that the polyherbal extract does not merely suppress the immune system; rather, it facilitates immunological rebalancing by promoting an anti-inflammatory environment that accelerates tissue healing and the resolution of diarrhoea. The normalization

of CRP, a gold-standard marker of acute systemic inflammation in rabbits, further validates that the T4 dose achieved complete systemic resolution of the infection**.**

Effects of combined aqueous extract treatment on Hepato-Renal Protection and Metabolic Homeostasis in *E. coli*-infected rabbits

The systemic metabolic profile and organ-functional integrity of the experimental rabbits are summarized in Figure 8 (Suppl Table 7). Induction of colibacillosis in the untreated cohort (T1) resulted in significant biochemical derangement ($P < 0.05$), characterized by a dyslipidemia of infection and acute hepato-renal distress. Specifically, T1 rabbits exhibited an increase in serum transaminases (ASAT, ALAT) and GGT, signalling acute hepatocellular injury and membrane leakage induced by bacterial endotoxins.

Renal function was similarly compromised in the untreated group, with Creatinine and Urate levels nearly doubling. This elevation indicates a reduced Glomerular Filtration Rate (GFR), likely secondary to infection-induced dehydration and septic renal injury. Furthermore, a state of hypoproteinemia and hypoalbuminemia was observed in T1, reflecting the systemic inflammatory response and potential protein-losing enteropathy associated with severe diarrhoea.

Therapeutic intervention with the *P. guajava* and *T. diversifolia* extracts effectively shielded the primary metabolic organs from this endotoxicity. The 800 mg/kg dose (T4) achieved a metabolic reset, returning ASAT, GGT, Creatinine, and Urate to baseline levels statistically indistinguishable from the healthy controls (T0). Notably, the extract restored the lipid profile by lowering triglycerides and LDL-cholesterol while significantly increasing HDL-cholesterol ($P < 0.05$) compared to the untreated group. By stabilizing hepatocellular membranes and maintaining renal perfusion, the 800 mg/kg dose proved to be a potent organoprotective agent, facilitating a return to systemic homeostasis comparable to the neomycin-treated cohort (T2).

Integrated Hierarchical Clustering: The Global Biological Fingerprint

To visualize the holistic therapeutic trajectory of the rabbits, hierarchical clustering analysis was performed on the integrated biological dataset (Figure 9). The resulting heatmap successfully discriminated between the various experimental cohorts, establishing a clear 'molecular signature' for both the pathological state and the subsequent recovery. The infected-untreated cohort (T1) formed a distinct cluster characterized by a massive upregulation of pro-inflammatory and damage-associated markers, specifically manifesting as elevated ASAT, creatinine, MDA, and pro-inflammatory cytokines (TNF-α, IL-6).

In stark contrast, the cohort treated with the 800 mg/kg polyherbal extract (T4) exhibited a dramatic reversal of this fingerprint. The T4 group clustered closely with the healthy control (T0) and the neomycin-treated (T2) groups, displaying a synchronized restoration of antioxidant enzymes (SOD, CAT, GSH) and a normalization of systemic biochemical indices. This global transition from the oxidative-inflammatory cluster to the homeostatic cluster underscores the multifaceted curative potential of the *P. guajava* and *T. diversifolia* combination. These results suggest that the high-activity extract does not merely target

isolated symptoms but induces a systemic biological reset, restoring the animal's physiological state to its pre-infection baseline.

Multivariate Trajectory and Key Biological Drivers

To synthesize the multidimensional recovery process, a Principal Component Analysis (PCA) was performed on the integrated dataset (Figure 10A). The PCA model revealed a distinct therapeutic trajectory, with the first two dimensions (Dim1 and Dim2) capturing over 50% of the total variance. Dim1, accounting for 48% of the variance, served as the primary axis of pathological discrimination, effectively segregating the 'diseased' coordinates of the untreated cohort (T1) from the 'homeostatic' cluster of the healthy controls (T0). Notably, the 800 mg/kg cohort (T4) exhibited a profound spatial shift, converging into the same multivariate space as the T0 and Neomycin (T2) groups. This spatial alignment mathematically demonstrates that the high activity polyherbal extract restores systemic homeostasis as effectively as conventional antibiotherapy.

The Variable Contribution analysis (Figure 10B) further elucidated the primary drivers of this systemic recovery. The restorative shift observed in the PCA was largely mediated by significant modulations in bacterial clearance kinetics (load at Day 10, 12, and 13) and systemic inflammatory markers (WBC and CRP). These variables exhibited the highest contribution scores, suggesting that the clinical success of the *P. guajava* and *T. diversifolia* combination is fundamentally rooted in its dual capacity for rapid intestinal pathogen eradication and the subsequent quenching of the systemic leukocyte response. By identifying these parameters as the dominant variance-drivers, the model confirms that bacterial clearance and inflammatory resolution are the definitive benchmarks of the extract's therapeutic profile.

Comparative Functional Efficacy and Therapeutic Parity

To evaluate the clinical viability of the polyherbal intervention as a sustainable alternative to conventional chemotherapy, a comparative functional analysis was conducted between the high-dose extract (800 mg/kg) and the Neomycin standard (Figure 11).

The restorative capacity of the *P. guajava* and *T. diversifolia* combination was found to be functionally equivalent to Neomycin across all oxidative stress indices. In both the caecum and ileum, the 800 mg/kg cohort exhibited a robust recovery of Superoxide Dismutase (SOD) and Catalase (CAT) activities, with mean values statistically indistinguishable from the antibiotic-treated group ($P > 0.05$). This indicates that the extract’s secondary metabolites effectively neutralize reactive oxygen species (ROS) and stabilize the intestinal redox landscape with a potency matching that of the gold-standard treatment.

In terms of immunomodulation, the extract achieved complete systemic stabilization. The suppression of C-reactive protein (CRP) a sensitive marker of acute-phase inflammation in rabbits, and the normalization of White Blood Cell (WBC) counts showed no significant divergence between the polyherbal and Neomycin cohorts. This demonstrates that the extract possesses a systemic anti-inflammatory mechanism comparable to the resolution achieved through bacterial clearance by synthetic antimicrobials.

The spatial proximity of the data points and the overlapping error bars across the faceted biological categories (Growth, Antioxidants, and Hematology) mathematically validate the high-activity profile of the formulation. By resolving the *E. coli* challenge through both direct antimicrobial action and active tissue repair, the 800 mg/kg dose demonstrated therapeutic parity with Neomycin. These findings support the use of this polyherbal combination as a potent biological alternative, capable of maintaining rabbit health and productivity without the necessity of conventional antibiotic administration.

Histopathology results

Effect of combined extract on the renal architecture

The protective effect of the *P. guajava* and *T. diversifolia* combination on the renal microarchitecture of *E. coli*-infected rabbits is presented in Figure 12. Microscopic examination of the kidney sections from the healthy control group (T0) revealed a well-preserved renal parenchyma. This was characterized by a distinct glomerular architecture (G) with a clear Bowman's space (US) and clearly differentiated proximal (PT) and distal (DT) convoluted tubules, indicating normal physiological filtration and reabsorption functions.

In contrast, the infected-untreated cohort (T1) exhibited significant histopathological derangement. The infection induced a localized inflammatory response marked by conspicuous leukocyte infiltration (IL) and mesangial expansion within the glomerulus. These alterations are indicative of acute renal distress and the systemic inflammatory burden caused by the pathogenic *E. coli* strain. However, therapeutic intervention with the polyherbal extract effectively mitigated these structural damages. The renal sections from the T3 (400 mg/kg) and T4 (800 mg/kg) groups, alongside the Neomycin-treated cohort (T2), demonstrated a restoration of the renal tissue architecture. While the 800 mg/kg dose (T4) showed minor residual leukocyte infiltrates, the overall prevention of mesangial expansion and tubular disruption confirms the extract's nephroprotective potential. This structural preservation is consistent with the biochemical results, where the extract successfully normalized serum creatinine and uric acid levels, confirming that the formulation prevents the acute kidney injury (AKI) typically associated with severe colibacillosis.

Protective Effect of Extracts on Hepatic Microarchitecture

The histological evaluation of the liver (Figure 13) confirms the organoprotective properties of the *P. guajava* and *T. diversifolia* combination during acute *E. coli* infection. Microscopic analysis of the liver from the healthy control group (T0) showed a classical hexagonal lobular arrangement with a well-preserved hepatic parenchyma. The sections exhibited a distinct portal triad comprising the hepatic portal vein (PV), hepatic artery (HA), and bile canaliculus (BC) surrounded by radiating cords of well-defined, polyhedral hepatocytes (He) with prominent nuclei.

In the infected-untreated cohort (T1), the hepatic architecture was compromised by a localized inflammatory response, characterized by significant leukocyte infiltration (IL). This

infiltration suggests that the systemic *E. coli* infection induced a degree of hepatitis or endotoxin-mediated tissue stress. However, administration of the polyherbal formulation at both 400 mg/kg (T3) and 800 mg/kg (T4) successfully mitigated these inflammatory changes. The hepatic sections of the treated groups showed a restoration of the hepatocellular cords and a marked absence of inflammatory clusters, appearing histologically comparable to the Neomycin-treated (T2) and healthy control (T0) groups.

This structural preservation directly correlates with the enzymatic profiles discussed earlier; by preventing the infiltration of inflammatory cells and maintaining the integrity of the hepatocellular membranes, the extract effectively prevented the leakage of transaminases (ASAT and ALAT) into the systemic circulation. These findings reinforce the dual role of the *P. guajava* and *T. diversifolia* combination as both an antimicrobial and a potent hepatoprotective agent.

Restorative Effects of extracts on Intestinal (Caecal) Microarchitecture

The impact of the *E. coli* challenge and the subsequent therapeutic intervention on the caecal microarchitecture is presented in Figure 14. Histological analysis of the caecal sections from healthy control rabbits (T0) revealed a robust and well-organized intestinal wall. The architecture followed a classic structural hierarchy, consisting of the serosa (Se), muscular layer (Mus), submucosa (Smu), and a well-developed mucosa (Mu) lining the intestinal lumen (Lu).

In the infected-untreated cohort (T1), the *E. coli* challenge induced severe mucosal degeneration. The sections exhibited a distinct narrowing of the intestinal mucosa and marked villous atrophy. This loss of structural height and complexity in the mucosal layer indicates a depletion of goblet cell function (leading to decreased mucus secretion) and a reduction in the total absorptive surface area. Such morphological damage provides a structural explanation for the persistent diarrheoa and negative growth performance observed in the T1 group.

Therapeutic intervention with the *P. guajava* and *T. diversifolia* combination (T3 and T4) effectively reversed these pathological shifts. The intestinal epithelium in the treated groups showed a restoration of mucosal thickness and villous integrity, appearing structurally comparable to the healthy controls and the Neomycin-treated cohort (T2). By maintaining the mucosal barrier and preventing villous blunting, the high-dose extract (800 mg/kg) preserved the intestinal 'gatekeeper' function, facilitating nutrient absorption and preventing the translocation of enteric pathogens. This structural recovery serves as the biological foundation for the observed resolution of clinical diarrhoea and the restoration of the animals' growth trajectory.

Discussion

While rabbit meat is often categorized as a niche product, its global demand is experiencing a significant upward trajectory, particularly within Asian markets [3]. Beyond its market growth, rabbit production offers a superior alternative for animal protein in terms of social equity [20] and environmental sustainability [2]. Despite this potential, the industry, especially

in developing nations, is severely constrained by enteric pathologies, most notably colibacillosis. Caused by pathogenic strains of *Escherichia coli*, this disease not only compromises animal health and productivity through high morbidity and mortality but also poses a critical One Health risk. The potential transmission of multidrug-resistant (MDR) strains through the food chain represents a burgeoning public health threat [21, 22]. As the efficacy of conventional antimicrobials wanes due to the global rise of antimicrobial resistance (AMR), the exploration of plant-based therapeutic alternatives has become a pharmacological necessity. Our previous ethnopharmacological screening of the Cameroonian flora identified *Psidium guajava* and *Tithonia diversifolia* as potent inhibitors of enteropathogenic *E. coli* (EPEC), with their combination exhibiting a remarkable synergistic reduction in Minimum Inhibitory Concentrations (MIC) by up to 64-fold [19]. Building upon that *in vitro* evidence, the present study was designed to evaluate the *in vivo* curative potential and systemic restorative effects of the *P. guajava* and *T. diversifolia* combination in a *E. coli*-infected rabbit.

The experimental induction of colibacillosis led to a profound clinical decline in the untreated cohort, characterized by acute weight loss, dehydration, and high-titer fecal shedding of CR+ *E. coli*. This pathological state is characteristic of enteropathogenic *E. coli* (EPEC) or enterohemorrhagic *E. coli* (EHEC) strains, which disrupt intestinal water transport and induce catabolic stress [23]. The rapid reversal of these symptoms following administration of the combined *P. guajava* and *T. diversifolia* extract specifically at 800 mg/kg demonstrates potent therapeutic efficacy. The achievement of bacterial clearance by Day 10 (Day 5 of treatment) is particularly significant. While Neomycin provides rapid onset killing, the combined extract matched its terminal efficacy. This clinical parity suggests that the polyherbal synergy documented in our previous *in vitro* studies [19] translates effectively to the complex *in vivo* environment. The dual-action mechanism likely involves the tannins and flavonoids of *P. guajava*, which cross-link bacterial cell wall proteins to inhibit adhesion and subsequent invasion [24], and the sesquiterpene lactones of *T. diversifolia*, which disrupt bacterial membrane integrity and enzymatic metabolism [25].

The septic hemogram observed in the untreated group, marked by leukocytosis, thrombocytosis, and secondary anemia, reflects the systemic inflammatory burden of colibacillosis [26]. The subsequent normalization of these parameters in all treated groups (T2–T4) indicates a total resolution of systemic stress. Crucially, the extract functioned as a robust immunomodulator rather than a simple immunosuppressant. By suppressing the cytokine production (TNF-α, IL-6, and IL-1β) and normalizing CRP levels, the formulation effectively extinguished the inflammatory cascade. This is likely mediated by the inhibition of the NF-kB signaling pathway, a known target of the polyphenolic compounds found in *T. diversifolia* [27]. Unlike antibiotics, which primarily target the pathogen, this polyherbal combination actively manages the host's immune response, preventing the tissue-damaging effects of chronic inflammation.

The observed hypoproteinemia and hypoalbuminemia in the T1 group highlight a state of negative acute phase response, where the liver shifts protein synthesis toward inflammatory mediators like CRP at the expense of albumin [28]. The simultaneous rise in ASAT and GGT

points to hepatocellular leakage, while elevated uric acid suggests impaired renal clearance or accelerated purine catabolism due to tissue necrosis. The ability of the polyherbal extract to restore these metabolic markers, as corroborated by our histological findings, underscores its hepatoprotective and nephroprotective properties. The preservation of the Portal Triad architecture in the liver and the tubular integrity in the kidney confirms that the formulation is non-toxic and actively shields vital organs from the endotoxicity associated with *E. coli* septicemia.

At the primary site of infection, colibacillosis-induced oxidative stress (elevated MDA and depleted SOD/CAT) serves as a driver for mucosal damage. The villous atrophy and mucosal narrowing observed in our histological sections provided a physical basis for the observed malabsorption. The combined extract functioned as a biological shield, restoring the mucosal thickness and villous height. This structural repair is inextricably linked to the restoration of the intestinal redox balance. By directly scavenging ROS and upregulating endogenous enzymes [29], the formulation preserved the vital function of the caecal epithelium. This suggests that the tannins in *P. guajava* provide a physical protective coating (astringent effect), while the antioxidants in *T. diversifolia* mitigate the underlying molecular damage, collectively promoting rapid mucosal healing.

The multivariate spatial convergence (PCA) and hierarchical clustering (Heatmap) demonstrate that the 800 mg/kg dose induces a systemic recovery indistinguishable from the healthy baseline and the antibiotic standard. By integrating antimicrobial, antioxidant, and immunomodulatory pathways, this *P. guajava* and *T. diversifolia* combination offers a pleiotropic therapeutic advantage over conventional monotherapies like Neomycin. This study provides a strong pharmacological foundation for the development of standardized botanical drugs to combat antibiotic-resistant colibacillosis in rabbit production.

Conclusion

The present study demonstrates that the combined aqueous extract of *Psidium guajava* and *Tithonia diversifolia* is a potent, high-activity therapeutic agent for the management of rabbit colibacillosis. The formulation achieved a biological reset, effectively transitioning the animals from a pathological state of sepsis and oxidative stress to a homeostatic state functionally equivalent to healthy controls. These findings demonstrate that this polyplant-based formulation provides a viable, non-toxic, and clinically equivalent alternative to conventional aminoglycoside antibiotics. By addressing both the pathogen and the host's systemic response, the *P. guajava* and *T. diversifolia* combination represents a sustainable solution against colibacillosis, particularly in the face of rising antimicrobial resistance, supporting the transition toward One Health-aligned veterinary practices.

While the present study establishes the curative efficacy of the *P. guajava* and *T. diversifolia* combination, further research is warranted to fully elucidate the molecular mechanisms involved. Specifically, phytochemical fingerprinting via LC-MS/MS is required for extract standardization, and 16S rRNA metagenomic profiling would provide deeper insights into the formulation's impact on the commensal intestinal microbiota compared to conventional aminoglycosides.

Supporting information

Data is provided within the supplementary information files.

Author contributions

Data curation, Formal analysis, Investigation, Methodology, Validation, Writing – original draft: Mireille Tuedom Chouegouong

Conceptualization, Data curation, Software, Formal analysis, Project administration, Resources, Supervision, Validation, Visualization, Writing – review & editing: Rufin Marie Kouipou Toghueo

Investigation, Methodology and Data analysis: Yanick Kevin Melogmo Dongmo[1],

Investigation, Methodology and Data analysis: Jaurès Marius Tsakem Nangap,

Investigation, Methodology and Data analysis: Michele Stella Majoumouo

Investigation, Methodology and Data analysis: Brice Pinlap

Conceptualization, Resources, Supervision: Fabrice Fekam Boyom and Kingsley Agbor Etchu

All the authors have read and approved the final manuscript.

Competing interests

The authors declare no competing interests.

Additional information

Supplementary information

References

1. Bordignon F, Xiccato G, Trocino A, Zuffellato A, Castellini C, Mattioli S, et al. Environmental impact of rabbit production systems : A farm-based cradle-to-gate analysis. Animal. 2025; 19(5): 101488. https://doi.org/10.1016/j.animal.2025.101488
2. Cesari V, Zucali M, Bava L, Gislon G, Tamburini A, Toschi I. Environmental impact of rabbit meat : The effect of production efficiency. Meat Science. 2018; 145: 447-454. https://doi.org/10.1016/j.meatsci.2018.07.011
3. European Commission. Directorate General for Health and Food Safety. Commercial rabbit farming in the European Union : Overview report. Publications Office. 2024. Available from: https://data.europa.eu/doi/10.2772/62174
4. Mancini S, Paci G. Probiotics in Rabbit Farming : Growth Performance, Health Status, and Meat Quality. Animals. 2021; 11(12): 3388. https://doi.org/10.3390/ani11123388
5. Siddiqui SA, Gerini F, Ikram A, Saeed F, Feng X, Chen Y. Rabbit Meat—Production, Consumption and Consumers' Attitudes and Behavior. Sustainability. 2023; 15(3): 2008. https://doi.org/10.3390/su15032008

6. Solans L, Arnal JL, Sanz C, Benito A., Chacón G, Alzuguren O.et al. Rabbit Enteropathies on Commercial Farms in the Iberian Peninsula : Etiological Agents Identified in 2018–2019. Animals. 2019; 9(12): 1142. https://doi.org/10.3390/ani9121142

7. Martignon M, Burel C, Licois D, Reperant E, Postollec G, Valat C, et al. Challenging the growing rabbit with a moderately pathogenic E. coli under ad libitum or limited feed intake conditions : Impact on digestive physiology, bacterial communities, and on post-weaning growth. World Rabbit Science. 2021; 29(1): 1. https://doi.org/10.4995/wrs.2021.14089

8. Long TM, Nisa S, Donnenberg MS, Hassel BA. Enteropathogenic Escherichia coli Inhibits Type I Interferon- and RNase L-Mediated Host Defense To Disrupt Intestinal Epithelial Cell Barrier Function. Infection and Immunity. 2014; 82(7): 2802-2814. https://doi.org/10.1128/IAI.00105-14

9. Farooq MZ, Wang X, Yan X. Effects of Aeriscardovia aeriphila on growth performance, antioxidant functions, immune responses, and gut microbiota in broiler chickens. Journal of Zhejiang University-SCIENCE B. 2023; 24(11): 1014-1026. https://doi.org/10.1631/jzus.B2200621

10. Kerek Á, Szabó Á, Barnácz F, Csirmaz B, Kovács L, Jerzsele Á. Antimicrobial Resistance in Commensal Bacteria from Large-Scale Chicken Flocks in the Dél-Alföld Region of Hungary. Veterinary Sciences. 2025; 12(8): 691. https://doi.org/10.3390/vetsci12080691

11. Silva A, Silva V, Tavares T, López M, Rojo-Bezares B, Pereira JE,et al. Rabbits as a Reservoir of Multidrug-Resistant Escherichia coli : Clonal Lineages and Public Health Impact. Antibiotics. 2024; 13(4): 376. https://doi.org/10.3390/antibiotics13040376

12. Sun C, Wang Z, Li Y, Huang J. Antibiotic resistance spectrums of Escherichia coli and Enterococcus spp. Strains against commonly used antimicrobials from commercial meat-rabbit farms in Chengdu City, Southwest China. Frontiers in Veterinary Science. 2024; 11: 1369655. https://doi.org/10.3389/fvets.2024.1369655

13. Elbehiry A, Marzouk E. From Farm to Fork : Antimicrobial-Resistant Bacterial Pathogens in Livestock Production and the Food Chain. Veterinary Sciences. 2025; 12(9): 862. https://doi.org/10.3390/vetsci12090862

14. Pandey S, Doo H, Keum GB, Kim ES, Kwak J, Ryu S, et al. Antibiotic resistance in livestock, environment and humans : One Health perspective. Journal of Animal Science and Technology. 2024; 66(2): 266-278. https://doi.org/10.5187/jast.2023.e129

15. Wickramasuriya SS, Ault J, Ritchie S, Gay CG, Lillehoj HS. Alternatives to antibiotic growth promoters for poultry : A bibliometric analysis of the research journals. Poultry Science. 2024; 103(9): 103987. https://doi.org/10.1016/j.psj.2024.103987

16. Zheng S, Li Y, Chen C, Wang N, Yang F. Solutions to the Dilemma of Antibiotics Use in Livestock and Poultry Farming : Regulation Policy and Alternatives. Toxics. 2025; 13(5): 348. https://doi.org/10.3390/toxics13050348

17. Liu C, Jullian V, Chassagne F. (2024). Ethnobotany, phytochemistry, and biological activities of Psidium guajava in the treatment of diarrhea : A review. Frontiers in Pharmacology, 15, 1459066. https://doi.org/10.3389/fphar.2024.1459066

18. Abe AE, De Oliveira CE, Dalboni TM, Chagas-Paula DA, Rocha BA, De Oliveira RB, et al. Anti-inflammatory sesquiterpene lactones from Tithonia diversifolia trigger different effects on human neutrophils. Revista Brasileira de Farmacognosia. 2015; 25(2): 111-116. https://doi.org/10.1016/j.bjp.2015.01.005

19. Chouegouong MT, Majoumouo MS, Menkem EZ, Yimgang LV, Toghueo RMK, Etchu KA, et al. Ethnopharmacological survey and antibacterial activity of medicinal plant extracts used against bacterial enteritis in rabbits. Advances in Traditional Medicine. 2023; 23(1): 213-223. https://doi.org/10.1007/s13596-021-00615-1

20. Leroy F, Petracci M. Rabbit meat : Valuable nutrition or too-cute-to-eat? World Rabbit Science. 2021; 29(4): 239-246. https://doi.org/10.4995/wrs.2021.12663

21. Mahmoudi P, Rashidi A, Idrees Hama Faraj S. Network meta-analysis of the prevalence and antimicrobial resistance of Escherichia coli isolated from bovine milk and dairy products : A global perspective. Journal of Dairy Science. 2025; 108(9): 9330-9344. https://doi.org/10.3168/jds.2024-26197

22. Risalvato J, Sewid AH, Eda S, Gerhold RW, Wu JJ. Strategic Detection of Escherichia coli in the Poultry Industry : Food Safety Challenges, One Health Approaches, and Advances in Biosensor Technologies. Biosensors. 2025; 15(7): 419. https://doi.org/10.3390/bios15070419

23. Swennes AG, Buckley EM, Parry NMA, Madden CM, García A, Morgan PB, et al. Enzootic enteropathogenic Escherichia coli infection in laboratory rabbits. J Clin Microbiol. 2012; 50. https://doi.org/10.1128/JCM.00832-12

24. Pereira GA, Chaves DSDA, Silva TME, Motta REDA, Silva ABRD, Patricio TCDC, et al. Antimicrobial Activity of Psidium guajava Aqueous Extract against Sensitive and Resistant Bacterial Strains. Microorganisms. 2023; 11(7): 1784. https://doi.org/10.3390/microorganisms11071784

25. Dongmo AN, Nguefack J, Dongmo JBL, Fouelefack FR, Azah, RU, Nkengfack EA, et al. Chemical characterization of an aqueous extract and the essential oil of Tithonia diversifolia and their biocontrol activity against seed-borne pathogens of rice. Journal of Plant Diseases and Protection. 2021; 128(3): 703-713. https://doi.org/10.1007/s41348-021-00439-w

26. Peek SF, Mcguirk SM, Sweeney RW, Cummings KJ. Infectious Diseases of the Gastrointestinal Tract. In Rebhun's Diseases of Dairy Cattle. Elsevier. 2018; 249-356. https://doi.org/10.1016/B978-0-323-39055-2.00006-1

27. Méndez-Martínez Y, Mendoza-Carranza PD, Ordóñez-Iglesia JP, Segovia-Montesdeoca VD. C, Moreno-Vera AN, Casanova-Erazo MA, et al. Dietary Tithonia diversifolia improves hematology, antioxidant capacity, liver health, and disease

resistance in hybrid of Oreochromis mossambicus × Oreochromis niloticus. Notulae Botanicae Horti Agrobotanici Cluj-Napoca. 2025; 53(2): 14399. https://doi.org/10.15835/nbha53214399

28. Zhou HH, Tang YL, Xu TH, Cheng B. C-reactive protein : Structure, function, regulation, and role in clinical diseases. Frontiers in Immunology. 2024; 15: 1425168. https://doi.org/10.3389/fimmu.2024.1425168

29. Liu J, Han X, Zhang T, Tian K, Li Z, Luo F. Reactive oxygen species (ROS) scavenging biomaterials for anti-inflammatory diseases : From mechanism to therapy. Journal of Hematology & Oncology. 2023; 16(1): 116. https://doi.org/10.1186/s13045-023-01512-7

30. Yadav V. Congo red binding and Plasmid Profile of E. coli Isolates of Poultry Origin. Journal of Animal Health and Production. 2014; 2(3): 31-32. https://doi.org/10.14737/journal.jahp/2014/2.3.31.32

31. Yousif AA. Effects of E.coli O157:H7 Experimental Infections on Rabbits : Donia Hatem Fadhel al-Taii and Afaf Abdulrahman Yousif. The Iraqi Journal of Veterinary Medicine. 2019; 43(1): 34-42. https://doi.org/10.30539/iraqijvm.v43i1.468

Figure Click here to access/download;Figure;Figures.doc

Figures

Fig.1: Phenotypic characterization of the enteropathogenic *E. coli* isolate using the API 20E analytical profile index system.

Fig. 2: Effect of the combined aqueous extracts of *Psidium guajava* and *Tithonia diversifolia* on the body weight variation in colibacillosis-infected rabbits.

Fig.3: Relative organ weights and macroscopic safety assessment of rabbits infected with *E. coli* following 7 days of treatment with *P. guajava* and *T. diversifolia* extracts.

Fig. 4: Dynamics of fecal *E. coli* shedding in rabbits following treatment with combined *P. guajava* and *T. diversifolia* extracts.

Fig. 5: Impact of combined *P. guajava* and *T. diversifolia* extracts on intestinal oxidative stress markers (MDA, SOD, CAT, and GSH) in *E. coli*-infected rabbits.

Fig.6: The effect of combined aqueous extract treatment on hematological stabilization and resolution of systemic inflammation.

Fig.7: Immunomodulatory Effects of combined *Psidium guajava* and *Tithonia diversifolia* extract in colibacillosis-infected rabbits.

Fig.8: Effects of combined aqueous extract treatment on Hepato-Renal Protection and Metabolic Homeostasis in *E. coli*-infected rabbits.

Fig. 9: intergrate curative fingerprint of combined *Psidium guajava* and *Tithonia diversifolia* extract.

Fig. 10: Multivariate Trajectory and Key Biological Drivers.

Fig.11: Comparative functional efficacy of 800 mg/kg dose vs Neomycin.

Fig.12: Micophotographs of the kidneys of rabbit challenged with *E. coli* infection and administratered with the combined of *Psidium guava* and *Tithonia diversifolia* extracts (Hematoxylin eosin X 100). T0: Uninfected rabbit that received only distilled water (normal control); T1: Rabbit challenged with *Escherichia coli* infection and left untreated (vehicle control); T2: Rabbit infected with *Escherichia coli* and treated with neomycin (10 mg/kg); T3 and T4 : Rabbit challenged with *Escherichia coli* infection and treated using 400 and 800 mg/kg of combined of *Psidium guava* and *Tithonia diversifolia* extracts; **Cd**: cell degeneration ;
US: urinary space; **G:** Glomerula; **IL**: Leukocyte infiltration; **DT:** differentiated distal bypass tubes; **PT:** differentiated proximal bypass tubes.

Fig. 13: Micophotographs of the liver of rabbit challenged with *E. coli* infection and administratered with the combined of *Psidium guava* and *Tithonia diversifolia* extracts (Hematoxylin eosin X 100). T0: Uninfected rabbit that received only distilled water (normal control); T1: Rabbit challenged with *Escherichia coli* infection and left untreated (vehicle control); T2: Rabbit infected with *Escherichia coli* and treated with neomycin (10 mg/kg); T3 and T4 : Rabbit challenged with *Escherichia coli* infection and treated using 400 and 800 mg/kg of combined of *Psidium guava* and *Tithonia diversifolia* extracts; **Cd**: cell degeneration

; **He** : Hepatocytes; **HA** : Hepatic artery; **PV** : Veine porte; **BC** : bile canaliculus; **IL** : Infiltrations of leucocytes

Fig. 14: Micophotographs of small of rabbit intestine challenged with *E. coli* infection and administratered with the combined of *Psidium guava* and *Tithonia diversifolia* extracts (Hematoxylin eosin X 100).
T0: Uninfected rabbit that received only distilled water (normal control); T1: Rabbit challenged with *Escherichia coli* infection and left untreated (vehicle control); T2: Rabbit infected with *Escherichia coli* and treated with neomycin (10 mg/kg); T3 and T4 : Rabbit challenged with *Escherichia coli* infection and treated using 400 and 800 mg/kg of combined of *Psidium guava* and *Tithonia diversifolia* extracts; **Cd**: cell degeneration ; **Lu** : intestinale lumen, **Mu** : mucous layer, **Smu** : the submucosa, **Mus** : muscular layer, **Se** : serosa

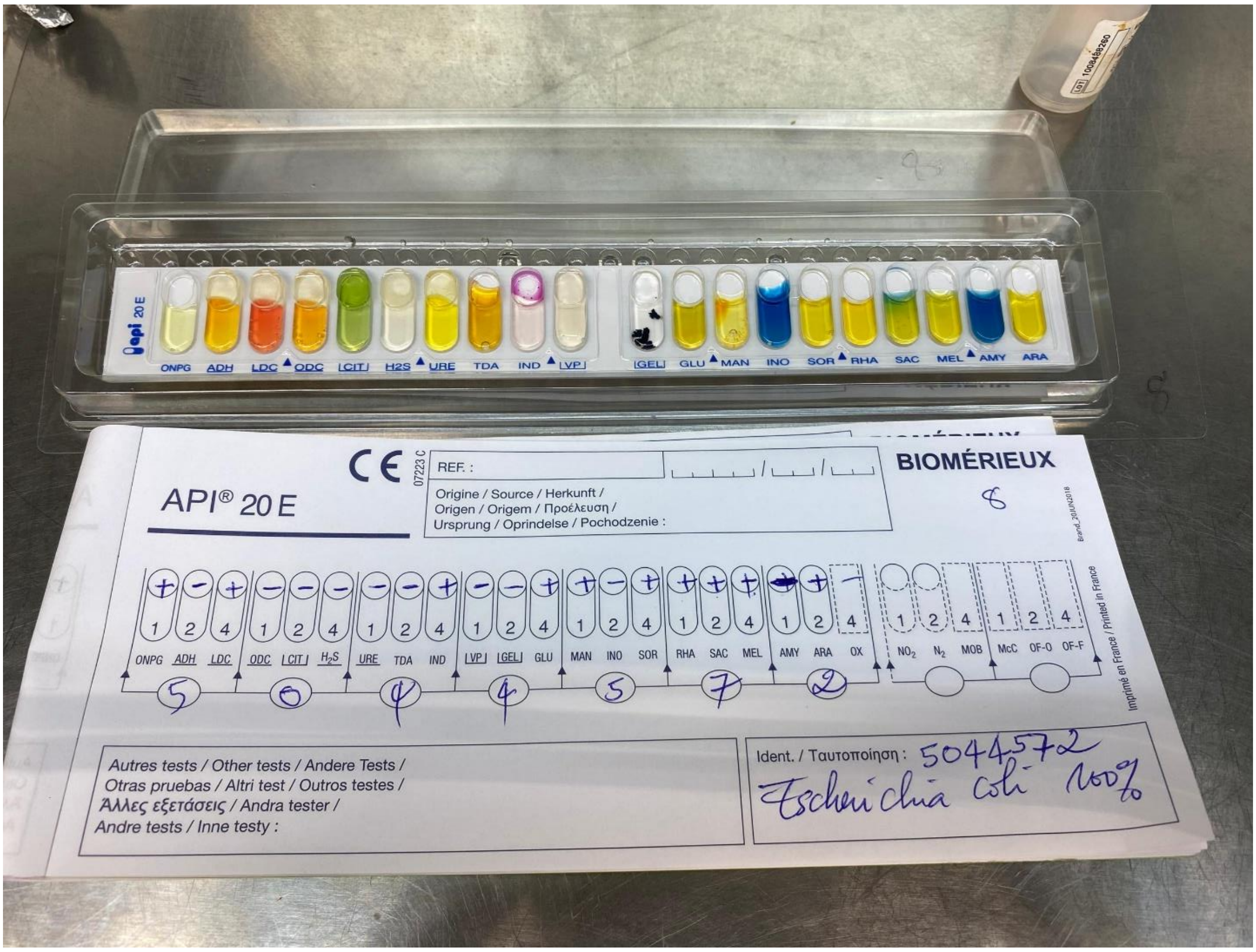
api 20 E
ONPG ADH LDC ODC CIT H2S URE TDA IND VP
GEL GLU MAN INO SOR RHA SAC MEL AMY ARA
API® 20 E
BIOMÉRIEUX
REF. :
Origine / Source / Herkunft /
Origen / Origem / Προέλευση /
Ursprung / Oprindelse / Pochodzenie :
ONPG ADH LDC ODC CIT H2S URE TDA IND VP GEL GLU MAN INO SOR RHA SAC MEL AMY ARA OX
NO2 N2 MOB McC OF-O OF-F
Autres tests / Other tests / Andere Tests /
Otras pruebas / Altri test / Outros testes /
Άλλες εξετάσεις / Andra tester /
Andre tests / Inne testy :
Ident. / Ταυτοποίηση : 5044572
Imprimé en France / Printed in France

Fig. 1

Fig. 2

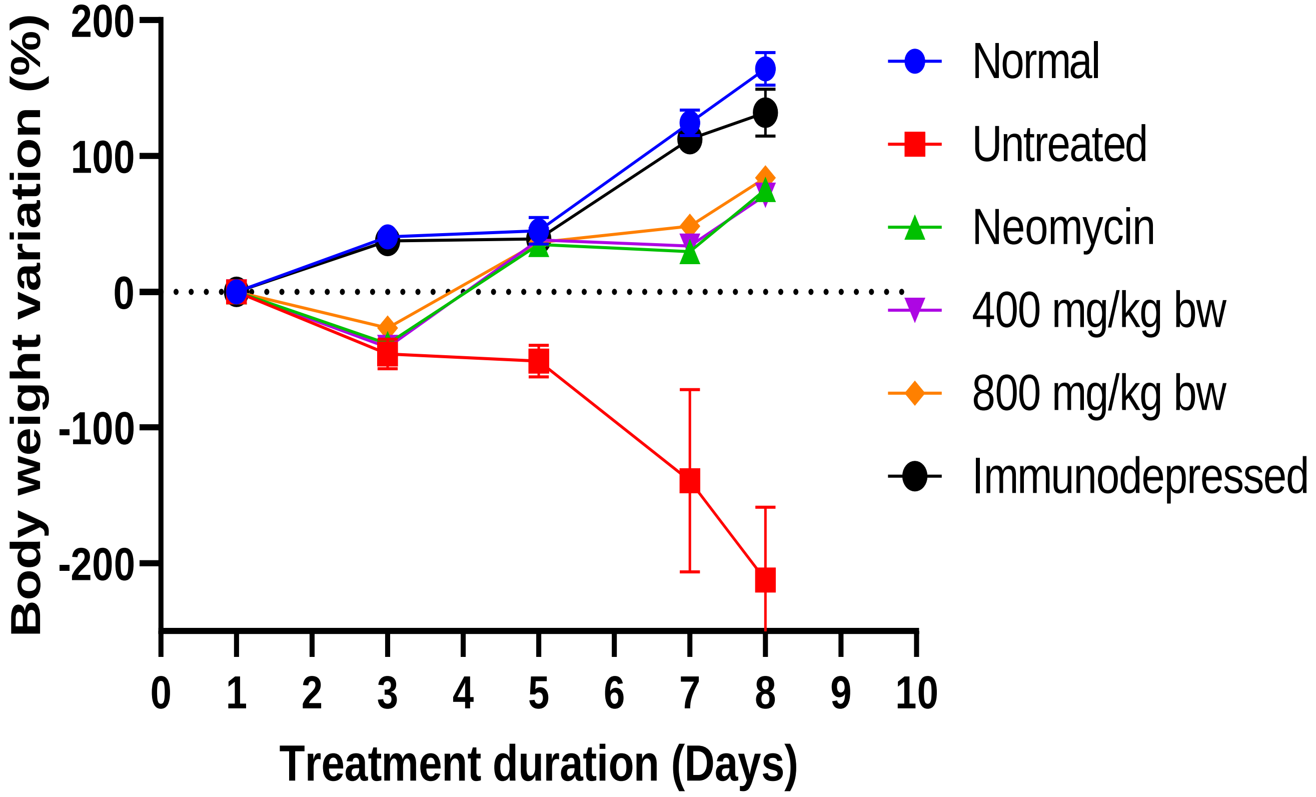
Body weight variation (%)
200
100
0
-100
-200
0
1
2
3
4
5
6
7
8
9
10
Treatment duration (Days)
Normal
Untreated
Neomycin
400 mg/kg bw
800 mg/kg bw
Immunodepressed

Fig. 3

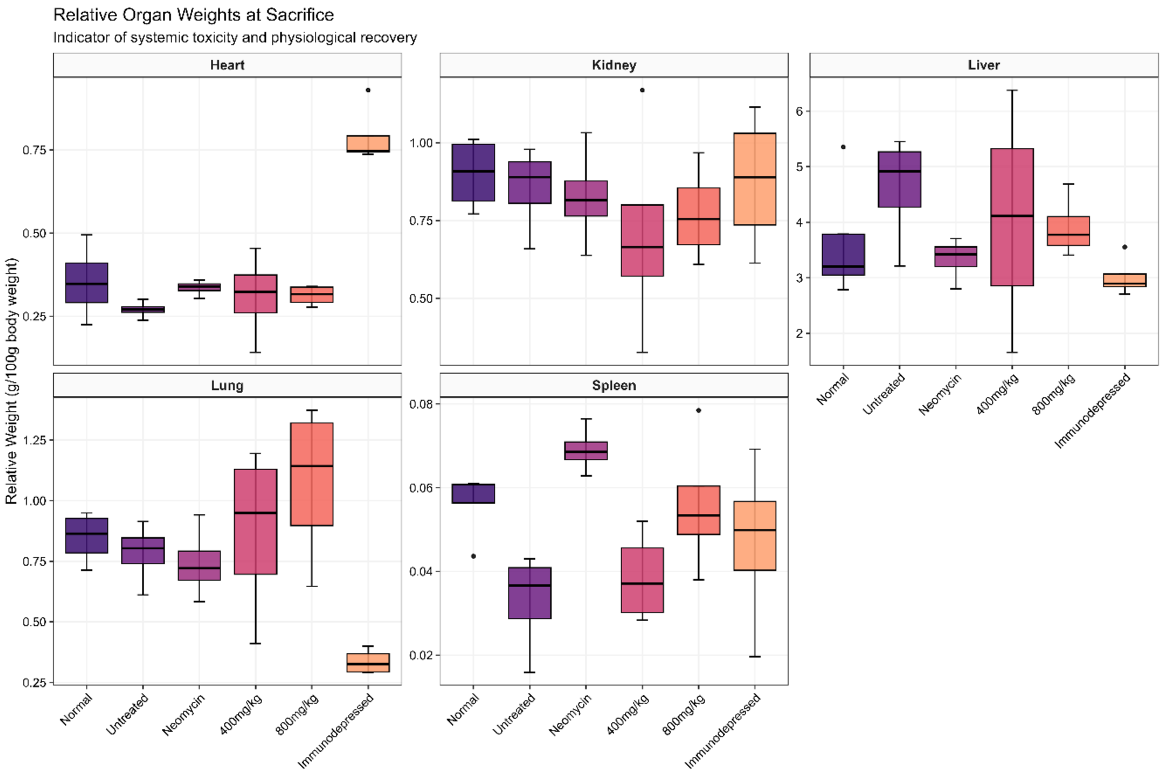
Relative Organ Weights at Sacrifice
Indicator of systemic toxicity and physiological recovery
Heart
Kidney
Liver
Lung
Spleen
Relative Weight (g/100g body weight)
Normal
Untreated
Neomycin
400mg/kg
800mg/kg
Immunodepressed

Fig. 4

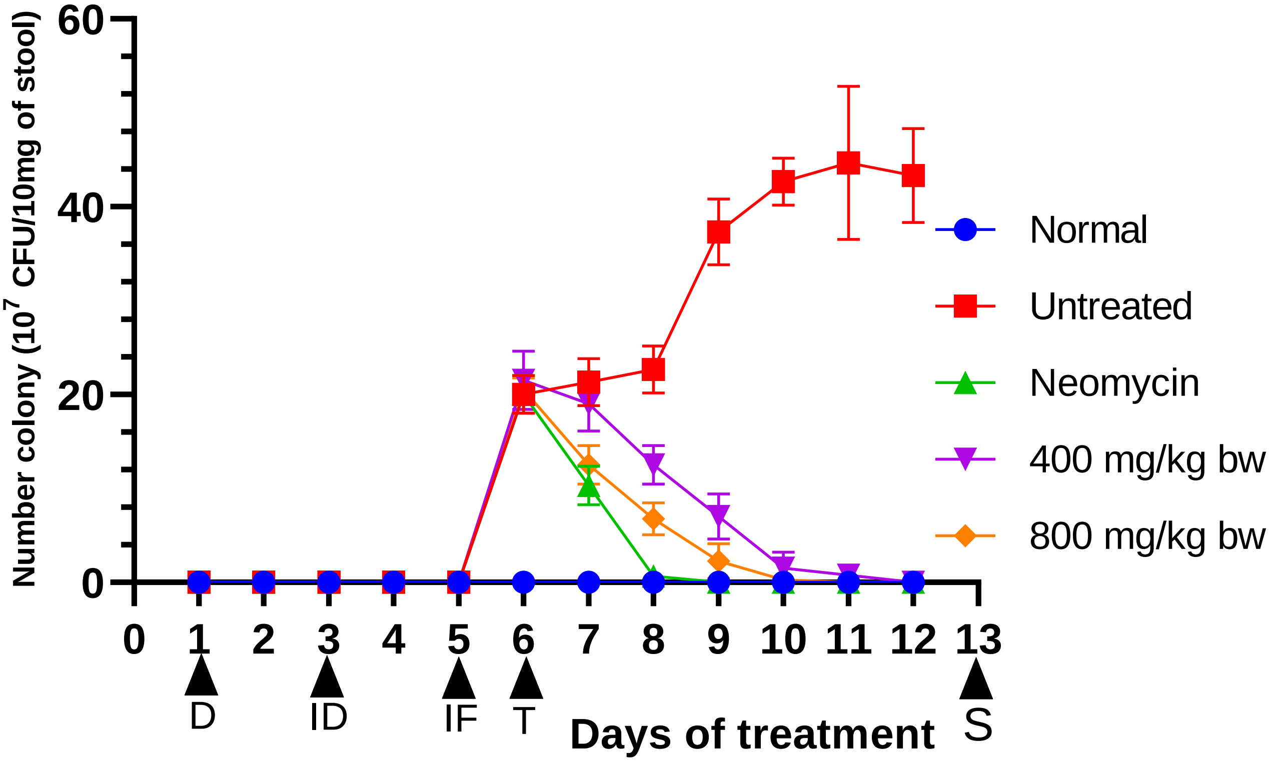
Number colony (10^7 CFU/10mg of stool)
60
40
20
0
0 1 2 3 4 5 6 7 8 9 10 11 12 13
D
ID
IF
T
S
Days of treatment
Normal
Untreated
Neomycin
400 mg/kg bw
800 mg/kg bw

Fig. 5

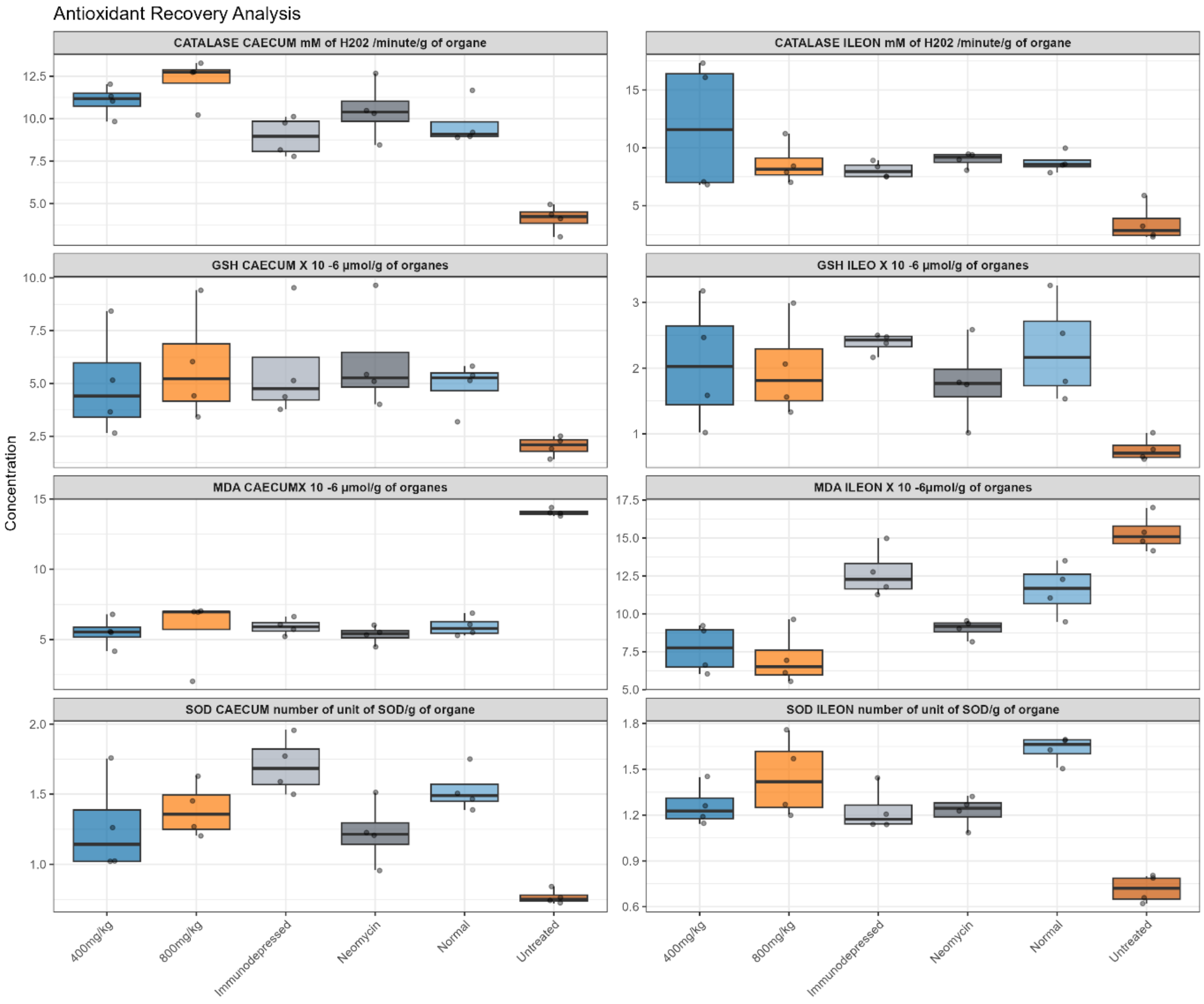
Antioxidant Recovery Analysis
CATALASE CAECUM mM of H202 /minute/g of organe
CATALASE ILEON mM of H202 /minute/g of organe
GSH CAECUM X 10 -6 µmol/g of organes
GSH ILEO X 10 -6 µmol/g of organes
MDA CAECUMX 10 -6 µmol/g of organes
MDA ILEON X 10 -6µmol/g of organes
SOD CAECUM number of unit of SOD/g of organe
SOD ILEON number of unit of SOD/g of organe
Concentration
400mg/kg
800mg/kg
Immunodepressed
Neomycin
Normal
Untreated

Fig.6

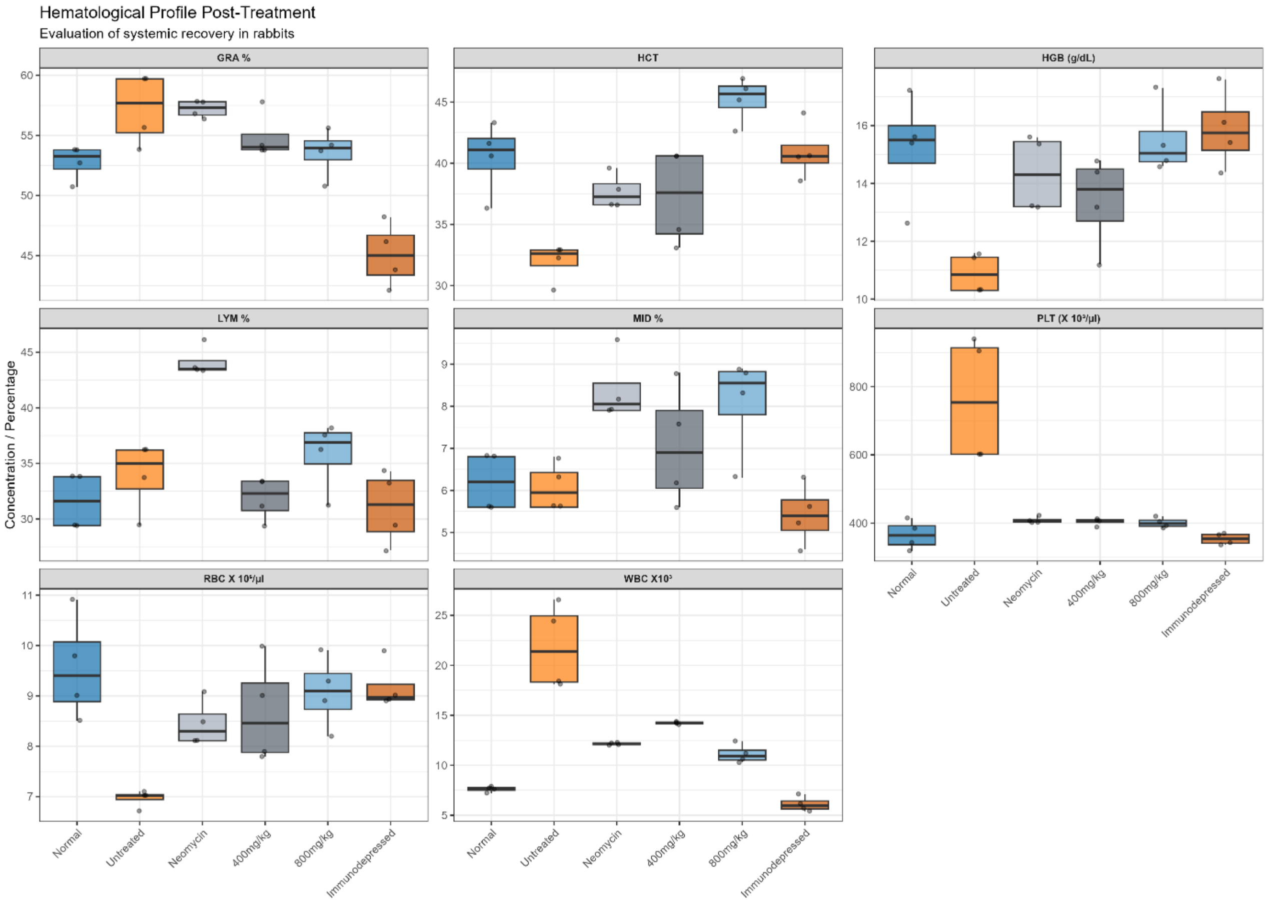
Hematological Profile Post-Treatment
Evaluation of systemic recovery in rabbits
GRA %
HCT
HGB (g/dL)
LYM %
MID %
PLT (X 10³/µl)
RBC X 10⁶/µl
WBC X10³
Concentration / Percentage
Normal
Untreated
Neomycin
400mg/kg
800mg/kg
Immunodepressed

Fig.7

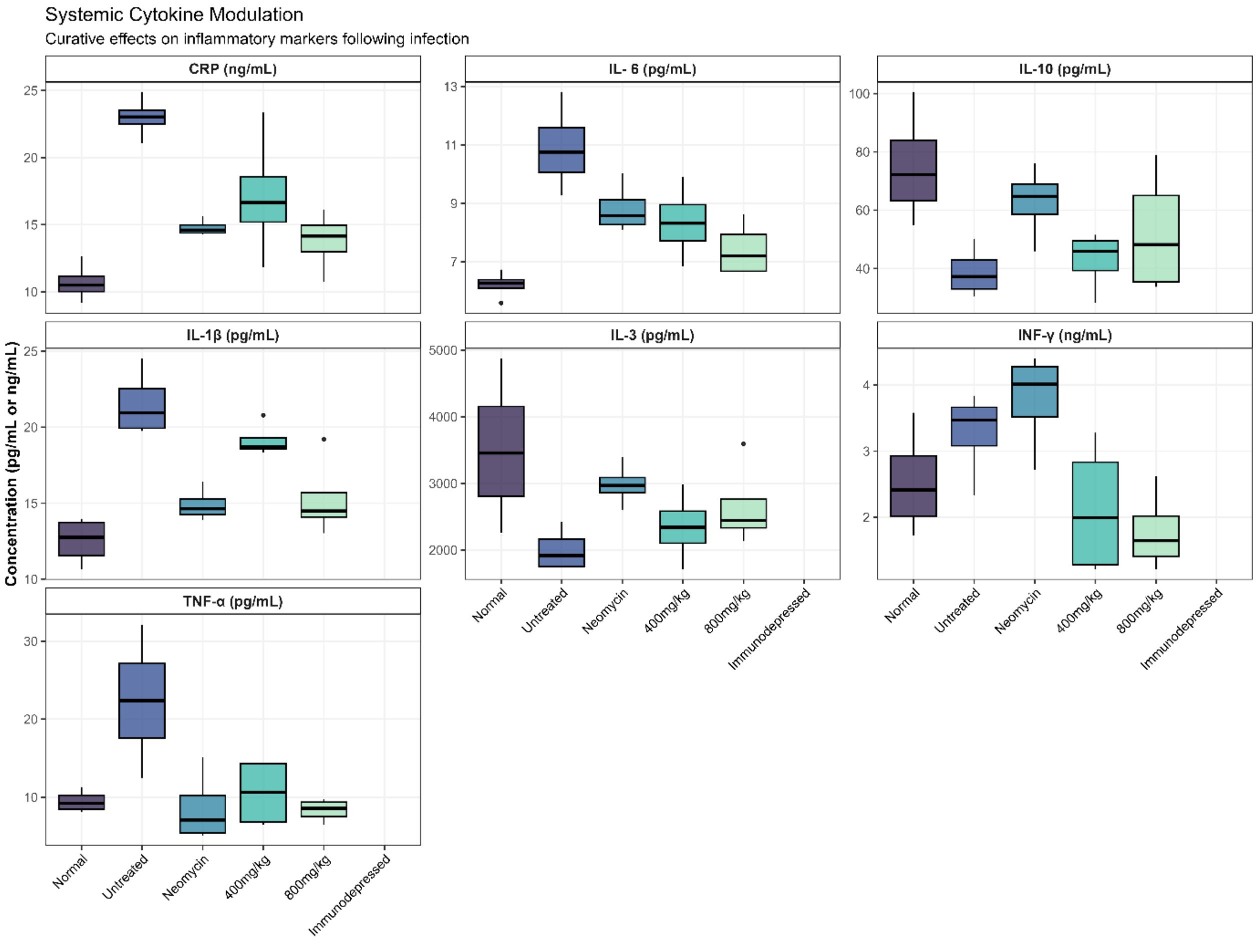
Systemic Cytokine Modulation
Curative effects on inflammatory markers following infection
CRP (ng/mL)
IL- 6 (pg/mL)
IL-10 (pg/mL)
IL-1β (pg/mL)
IL-3 (pg/mL)
INF-γ (ng/mL)
TNF-α (pg/mL)
Concentration (pg/mL or ng/mL)
Normal
Untreated
Neomycin
400mg/kg
800mg/kg
Immunodepressed

Fig. 8

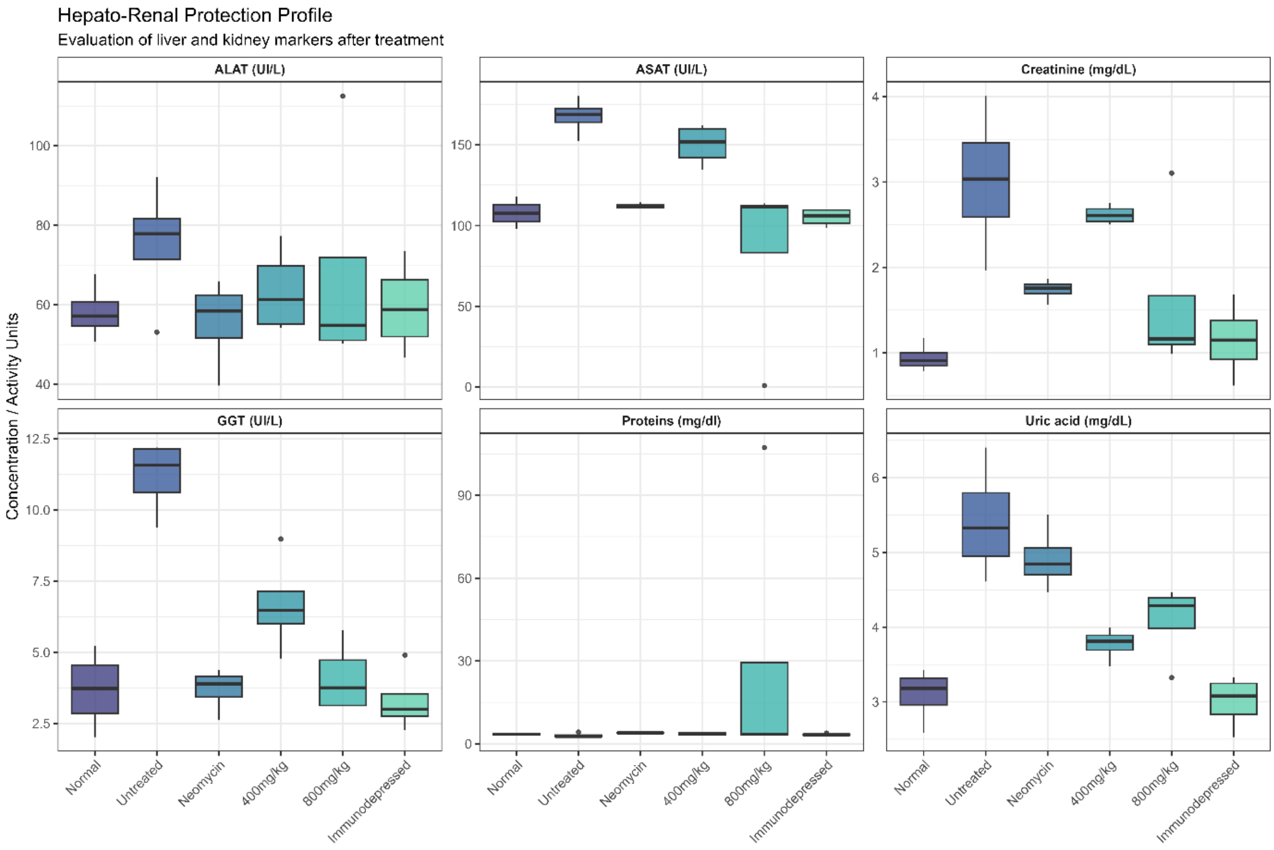
Hepato-Renal Protection Profile
Evaluation of liver and kidney markers after treatment
ALAT (UI/L)
ASAT (UI/L)
Creatinine (mg/dL)
GGT (UI/L)
Proteins (mg/dl)
Uric acid (mg/dL)
Concentration / Activity Units
Normal
Untreated
Neomycin
400mg/kg
800mg/kg
Immunodepressed

Fig. 9

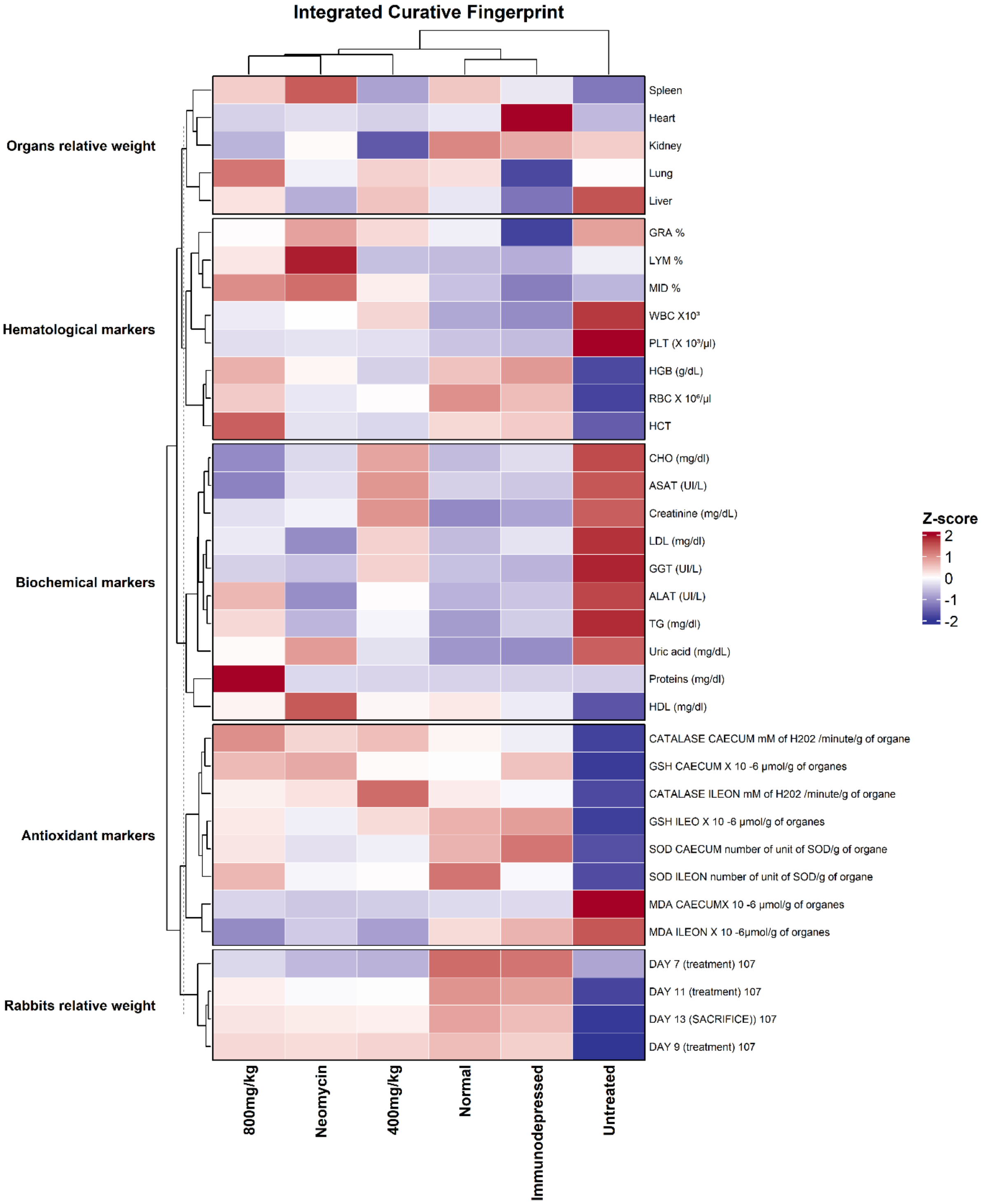
Integrated Curative Fingerprint
Organs relative weight
Hematological markers
Biochemical markers
Antioxidant markers
Rabbits relative weight
Spleen
Heart
Kidney
Lung
Liver
GRA %
LYM %
MID %
WBC X10³
PLT (X 10³/µl)
HGB (g/dL)
RBC X 10⁶/µl
HCT
CHO (mg/dl)
ASAT (UI/L)
Creatinine (mg/dL)
LDL (mg/dl)
GGT (UI/L)
ALAT (UI/L)
TG (mg/dl)
Uric acid (mg/dL)
Proteins (mg/dl)
HDL (mg/dl)
CATALASE CAECUM mM of H2O2 /minute/g of organe
GSH CAECUM X 10 -6 µmol/g of organes
CATALASE ILEON mM of H2O2 /minute/g of organe
GSH ILEO X 10 -6 µmol/g of organes
SOD CAECUM number of unit of SOD/g of organe
SOD ILEON number of unit of SOD/g of organe
MDA CAECUMX 10 -6 µmol/g of organes
MDA ILEON X 10 -6µmol/g of organes
DAY 7 (treatment) 107
DAY 11 (treatment) 107
DAY 13 (SACRIFICE)) 107
DAY 9 (treatment) 107
800mg/kg
Neomycin
400mg/kg
Normal
Immunodepressed
Untreated
Z-score
2
1
0
-1
-2

Fig. 10

10A

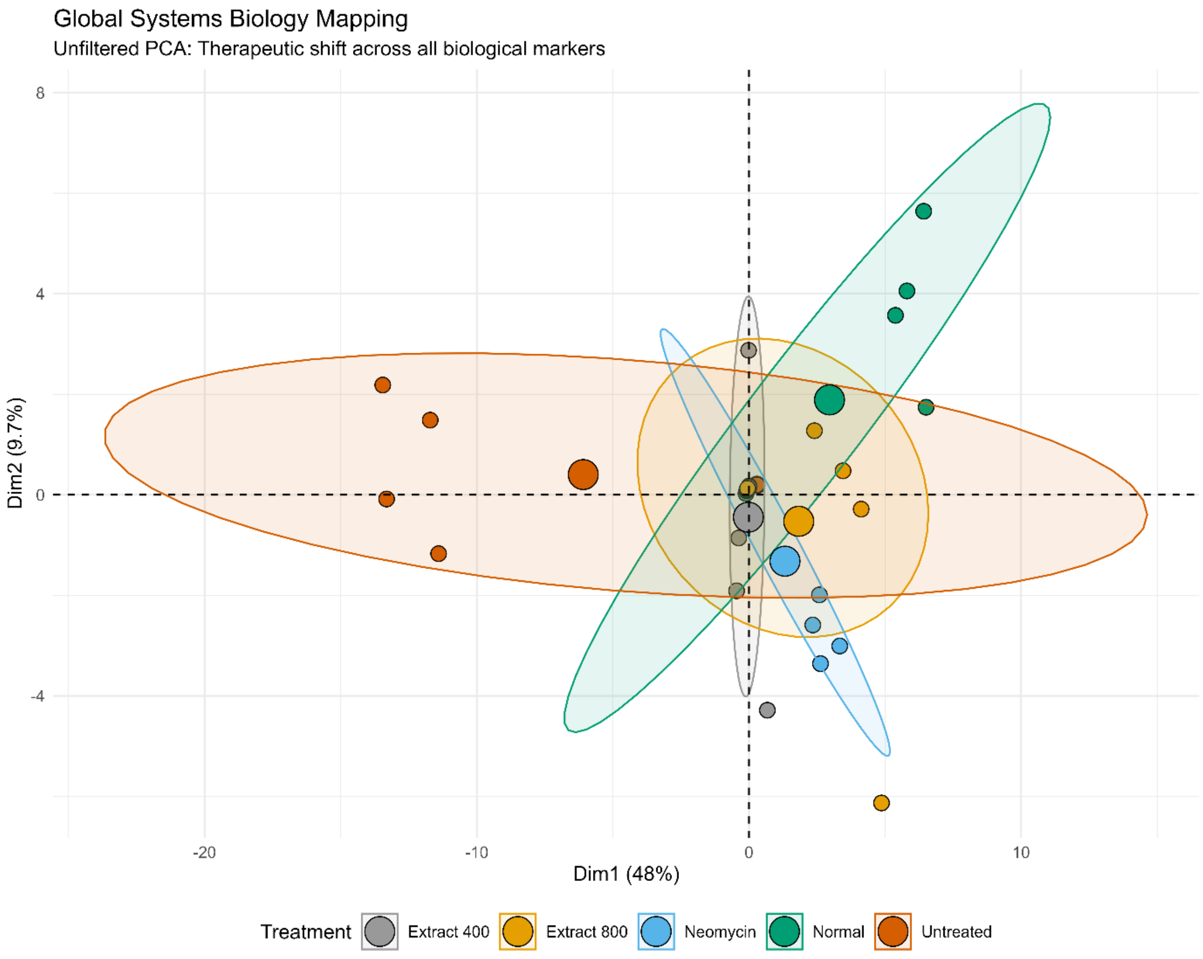
Global Systems Biology Mapping
Unfiltered PCA: Therapeutic shift across all biological markers
8
4
0
-4
Dim2 (9.7%)
-20
-10
0
10
Dim1 (48%)
Treatment
Extract 400
Extract 800
Neomycin
Normal
Untreated

10B

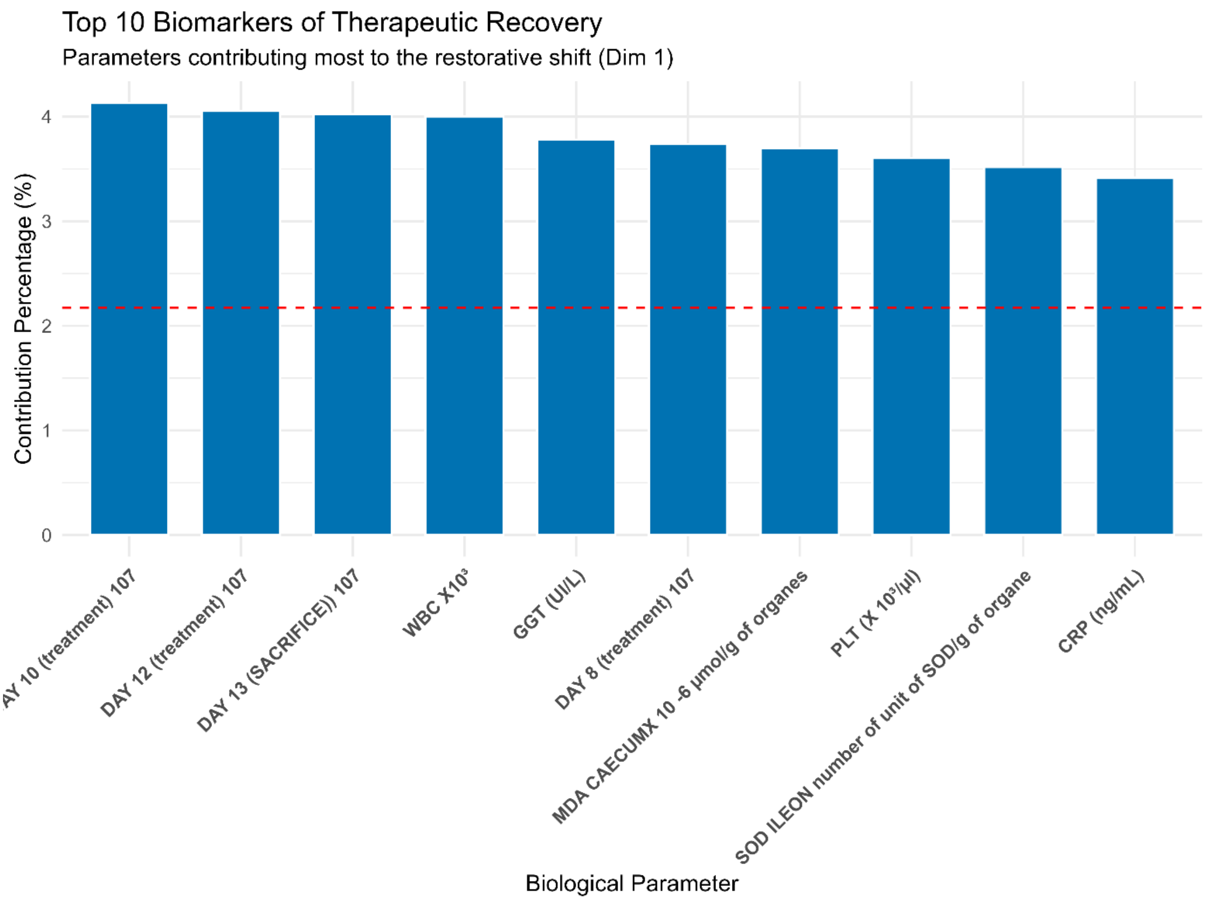

Top 10 Biomarkers of Therapeutic Recovery
Parameters contributing most to the restorative shift (Dim 1)
Contribution Percentage (%)
0
1
2
3
4
AY 10 (treatment) 107
DAY 12 (treatment) 107
DAY 13 (SACRIFICE)) 107
WBC X10³
GGT (UI/L)
DAY 8 (treatment) 107
MDA CAECUMX 10 -6 µmol/g of organes
PLT (X 10³/µl)
SOD ILEON number of unit of SOD/g of organe
CRP (ng/mL)
Biological Parameter

Fig. 11

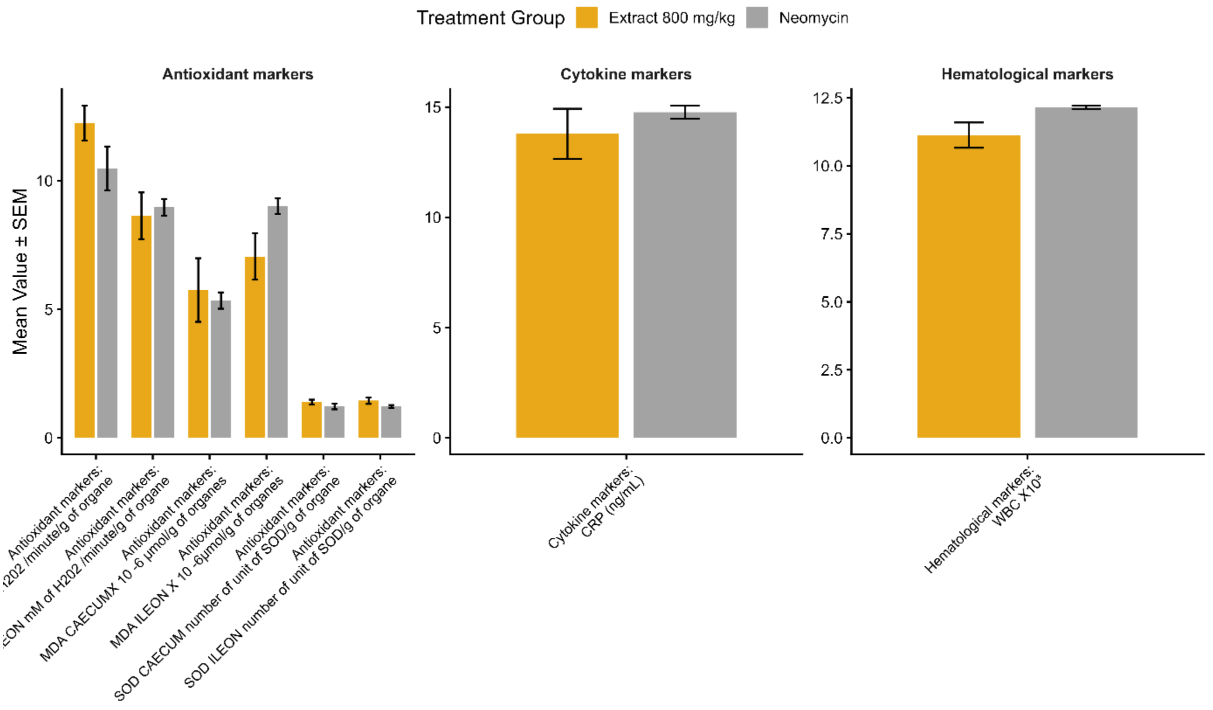
Treatment Group
Extract 800 mg/kg
Neomycin
Antioxidant markers
Cytokine markers
Hematological markers
Mean Value ± SEM
0
5
10
15
0.0
2.5
5.0
7.5
10.0
12.5
Antioxidant markers:
202 /minute/g of organe
Antioxidant markers:
ON mM of H202 /minute/g of organe
Antioxidant markers:
MDA CAECUMX 10 -6 µmol/g of organes
Antioxidant markers:
MDA ILEON X 10 -6µmol/g of organes
Antioxidant markers:
SOD CAECUM number of unit of SOD/g of organe
Antioxidant markers:
SOD ILEON number of unit of SOD/g of organe
Cytokine markers:
CRP (ng/mL)
Hematological markers:
WBC X10³

Fig. 12

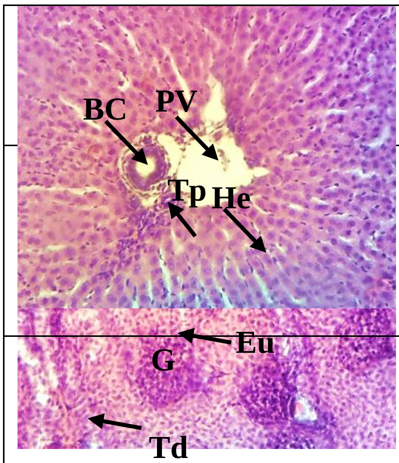	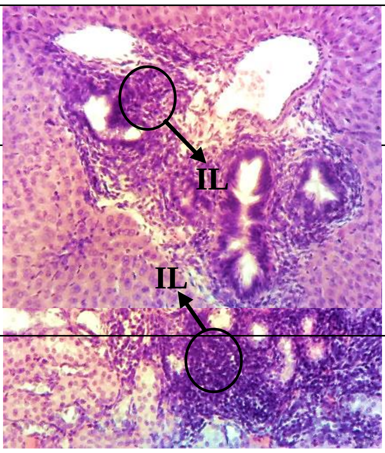	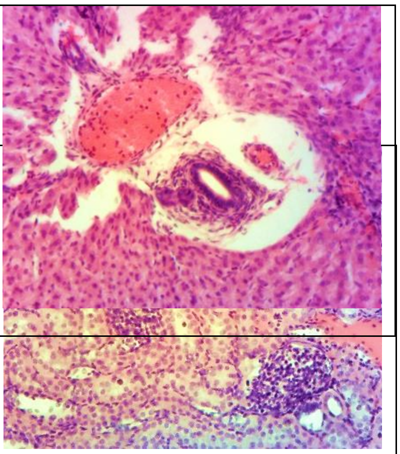
**T0**	**T1**	**T2**
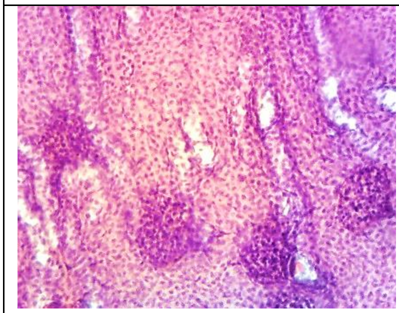	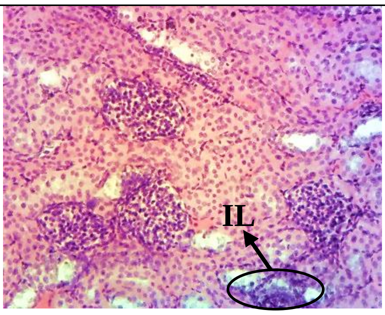	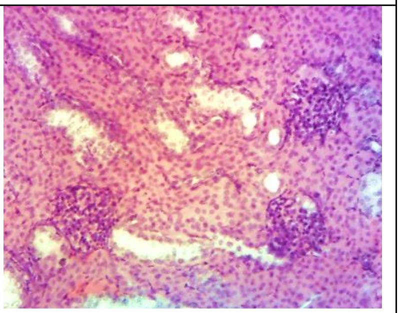
**T3**	**T4**	**T5**

HA

**T0**	**T1**	**T2**
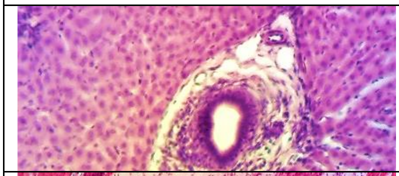<br>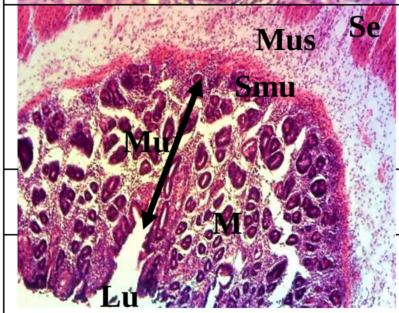 	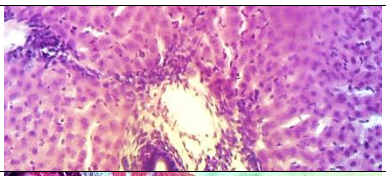<br>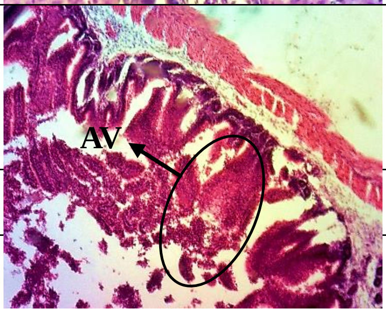 	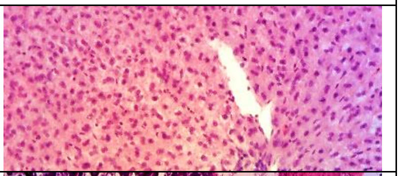<br>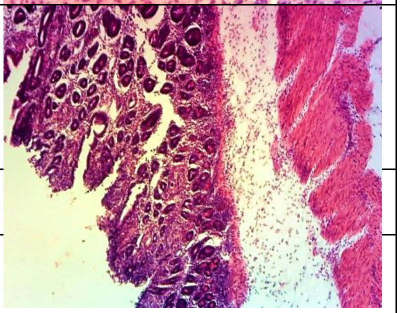

Fig. 13

Fig.14

**T0**	**T1**	**T2**
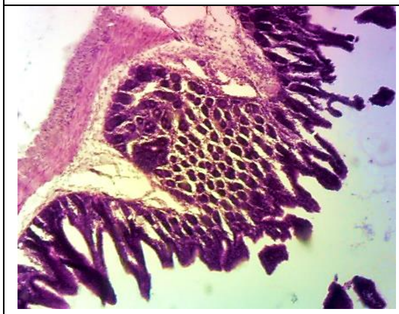	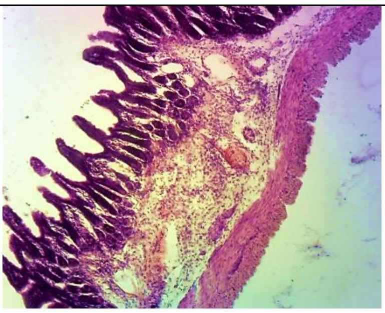	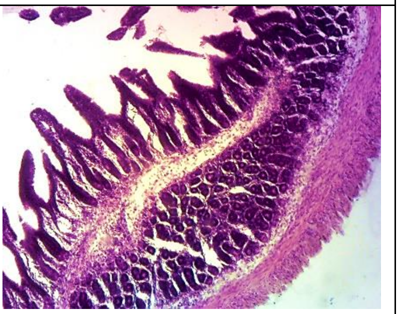
**T3**	**T4**	**T5**

Click here to access/download
Supporting Information
Supplementary materials.docx
