# Supplementary material for: A combined aqueous extract of Psidium guajava L. and Tithonia diversifolia (Hemsl.) A. gray improves metabolic homeostasis and preserves intestinal integrity in experimental rabbit colibacillosis
Source: PLoS One. 2026 Sep 15;21(9):e0357951. doi: 10.1371/journal.pone.0357951 (PMC13577389; doi:10.1371/journal.pone.0357951)
Supplement: Supplementary file 6 [file pone.0357951.s007.doc]

**Response to Reviewers' Comments**

**Manuscript ID: PONE-D-26-07694R1**

**Reviewer #1:**

**Comment1:** The authors should include the statistical software used in the methods section of the abstract.

**Response 1**: *The names of the softwares employed for the data analysis have now been mentioned in the Methods section of the abstract.*

**Comment2:** Also, the sentences starting with to, for, and any, should be constructed appropriately.

**Response 2**: *The sentences starting with " to, for, and any " have been revised in the entire manuscript.*

**Comment 3:** The graph axis should be checked and labelled. The limitations should be written in point form.

**Response 3:** *The figures axis was reviewed and revised, and the limitations section written in point form.*

**Comment 4:** The references should be checked and edited, font size checked too. The manuscript has been highlighted.

**Response 4:** *The references and manuscript formatting, including font size, were carefully checked and corrected according to the journal’s guidelines.*

**Reviewer #2:**

**Comment** 1. This suggestion is not carried out properly and it should be rectified (Check the abbreviations throughout the manuscript and introduce the abbreviation when the full word appears first time in the abstract and the remaining for the text and then use only the abbreviation). For example, IL-1β, IL-6, C-reactive protein (CRP), superoxide dismutase (SOD), and these types of corrections need to be checked all other abbreviations used in the manuscript.

**Response 1:** *All abbreviations have been carefully checked and standardized throughout the manuscript.*

**Comment 2**. The technical terms (Latin Phrase) "in vitro" should be italic and it should be checked all over the manuscript.

**Response 2:** *The expression in vitro, as well as other Latin expressions such as in vivo, has been italicized consistently throughout the manuscript*.
