# Supplementary material for: A combined aqueous extract of Psidium guajava L. and Tithonia diversifolia (Hemsl.) A. gray improves metabolic homeostasis and preserves intestinal integrity in experimental rabbit colibacillosis
Source: PLoS One. 2026 Sep 15;21(9):e0357951. doi: 10.1371/journal.pone.0357951 (PMC13577389; doi:10.1371/journal.pone.0357951)
Supplement: Supplementary file 5 [file pone.0357951.s006.docx]

**Response to Reviewers' Comments**

**Comments reviewers and editor**: We thank the Editor and the three Reviewers for their constructive evaluation of our manuscript. Based on they inputs and recommendations, we have revised the entire manuscript, remade all figures and figure captions. Below, we tried to the best of our ability to respond point-by-point to every comment. Reviewer comments are reproduced in bold italics; our responses follow in red.

**Slight change in the title**: The original title was revised to align with the revised manuscript. The new title is: A Combined Aqueous Extract of *Psidium guajava* L. and *Tithonia diversifolia* (Hemsl.) A. Gray Improves Metabolic Homeostasis and Preserves Intestinal Integrity in Experimental Rabbit Colibacillosis

**Manuscript ID: PONE-D-26-07694**

# Reviewer #1

*The manuscript is interesting, but some corrections need to be addressed.*

**Comment 1:** *The volume of the bacterial suspension used for induction should be included.*

Response 1: The disease was induced by administering 1 mL /100 g of body weight of bacterial suspension, adjusted to a 10^9^ CFU/mL in 0.9% saline. This information has been added to the Materials and Methods section.

**Comment 2:** *The software used for data analysis should also be included.*

Response 2**:** Data were analysed using GraphPad Prism and OriginPro, and multivariate analysis was performed in the R environment. This information has been added to the corresponding Methods section (see also Reviewer #2, Comment 10, for the full software citation).

**Comment 3:** *The term “polyherbal extract” should be replaced with “the combined extract of …”.*

Response 3: The term “polyherbal extract” has been replaced throughout the manuscript with “the combined *Psidium guajava* and *Tithonia diversifolia* extracts,” as suggested.

**Comment 4:** *The preparation of the combined extract should be described, and the rationale for the doses used in the experiment should be explained (the doses appear high).*

Response 4: The preparation of the combined extract has been fully described in the Methods section. Briefly, the two extracts were combined in a 96.975% *P. guajava* : 3.025% *T. diversifolia* ratio (w/w), determined from the synergistic Fractional Inhibitory Concentration Index (FICI = 0.265) established in our prior in vitro work (Chouegouong et al., 2023; doi:10.1007/s13596-021-00615-1). Fresh stock solutions were prepared daily in sterile distilled water at 40 mg/mL and 80 mg/mL to deliver 400 and 800 mg/kg bw, respectively. The administration was done by oral gavage at a fixed volume of 10 mL/kg bw (i.e., 387.9 mg/kg *P. guajava* + 12.1 mg/kg *T. diversifolia* for the low dose, and 775.8 mg/kg *P. guajava* + 24.2 mg/kg *T. diversifolia* for the high dose). The selected doses (400 and 800 mg/kg bw) are consistent with standard pharmacological evaluations of crude phytotherapeutic agents in lagomorph models, as previously reported by Hirudkar et al. (2020); this rationale, together with the full preparation protocol, has been added to the Methods section.

**Comment 5:** *Most sentences should be structured appropriately; sentences should not begin with “to” or “by”.*

Response 5: Constructions beginning with “To” or “By” have been revised throughout the manuscript to improve phrasing and fluency.

**Comment 6:** *The explanations of the results obtained do not accurately reflect the results or experiments performed; there is considerable overstatement.*

Response 6: The results section has been rephrased to remove overstatement and to ensure that the interpretations presented are directly supported by the data obtained.

**Comment 7:** *The conclusion should be rewritten to accurately reflect the work done, both in the abstract and throughout the manuscript.*

Response 7: The conclusion has been rewritten in both the abstract and the main text to align precisely with the results obtained (see also Reviewer #2, Comment 12, and Reviewer #3, Minor Comment 6).

**Comment 8:** *The manuscript should be rewritten to better reflect the results obtained; the figures are confusing for the reader.*

Response 8: The manuscript has been revised to better reflect the data generated, all figures are now explicitly cited and described within the text, and every figure legend has been rewritten to be self-contained and self-interpretable (see the accompanying Revised Figure Legends document, and Reviewer #2, Minor Comment 4).

**Comment 9:** *The manuscript contains some inconsistencies, which have been highlighted.*

Response 9: Thank you to all reviewers for flagging these inconsistencies. We have revised the entire manuscript and checked all results individually against the raw data and corrected; the corresponding changes are marked in the revised manuscript.

# Reviewer #2

**Comment 1:** *Some grammatical, alignment, and typographical errors are noted in the manuscript; it should be thoroughly checked and corrected throughout.*

Response 1: We thank the Reviewer for this observation. We have carefully re-read the entire manuscript and performed a thorough revision to correct all grammatical, alignment, and typographical errors identified.

**Comment 2:** *The use of abbreviations in the abstract may distract readers who wish to quickly skim several publications before deciding to read one in full. It may help to write terms out in full in the abstract (for example, AST).*

Response 2: We have revised the abstract to write out all abbreviated terms in full.

**Comment 3:** *Abbreviations should be checked throughout the manuscript: the full term should be introduced at first mention in the abstract and again at first mention in the main text, after which only the abbreviation should be used (for example, IL-1β, IL-6, superoxide dismutase (SOD), etc.). Only terms repeated at least three times in the text should be abbreviated.*

Response 3: We have thoroughly reviewed all abbreviations throughout the manuscript, introducing each full term at first mention before subsequent use of the abbreviation, and removing abbreviations for terms that did not meet the repetition threshold. The corresponding revisions have been incorporated into the updated manuscript and into the revised figure legends (see accompanying document).

**Comment 4:** *The full species name should be given at first mention in both the abstract and the main text, followed thereafter by the abbreviated genus initial (for example, Psidium guajava / Tithonia diversifolia at first mention, followed by P. guajava / T. diversifolia); this should be checked for all species used in the manuscript.*

Response 4: We have revised the entire manuscript and edited the full species of the two plants.

**Comment 5:** *The time (season) of collection of the plant species should be added, since the collection period is known to affect phytochemical constituents; this should be given top priority.*

Response 5: We completely agree with the reviewer that transparency in botanical processing is fundamental for reproducibility. We have now updated the "Ethnobotanical Selection and Extract Preparation" section to include the exact exact collection dates and season for both plant species, the starting fresh weights, resulting dry powder weights, and calculated fresh-to-dry ratios for both species.

**Comment 6:** *The quantity of fresh plant material used and the quantity of powder obtained (i.e., the fresh-to-powder ratio) should be included under Materials and Methods.*

Response 6: We have added the quantities of fresh plant material and the corresponding dry powder yield to the Materials and Methods section.

**Comment 7:** *The standard diet used in the study should be described, including its composition and source.*

**Response 7:** Information regarding the composition of the standard diet has been incorporated into the revised manuscript.

**Comment 8:** *In the Materials and Methods, references should be cited for any standard protocols or manufacturer instructions used, and the corresponding citations should be added to the reference list.*

Response 8: The Methods section has been updated to include references for the standard protocols used, and the corresponding citations have been added to the reference list.

**Comment 9:** *The dose selected for the present study should be justified in the Materials and Methods, either based on the available literature or a previous study; if derived from another study, it should be supported with the appropriate reference.*

Response 9: The dose justification, supported by the relevant reference (Chouegouong et al., 2023; Hirudkar et al., 2020), has been incorporated into the revised Materials and Methods section (see also Reviewer #1, Comment 4).

**Comment 10:** *The authors should cite the software fully when referring to it. GraphPad Prism version 8.0.1 (GraphPad Software, San Diego, CA, USA).*

Response 10: We have updated the Methods section to include this full software citation.

**Comment 11:** *Necessary expansions should be given in the figure legends, for example for MDA, SOD, CAT, and GSH.*

Response 11: We have rewritten every figure legend so that all abbreviations (including MDA, SOD, CAT and GSH) are expanded at first mention within each individual legend, in line with journal style for self-contained legends. Please see the accompanying Revised Figure Legends document.

**Comment 12:** *The conclusion provides a comprehensive overview of the work but could be more realistic in relation to the evidence presented, to better convey the outcome of the investigation. The authors may also wish to include the limitations of the present findings.*

Response 12: We have revised the conclusion to be more precise and directly grounded in the evidence generated by the present study, and we have added a dedicated statement on the limitations of the study and on future investigations, as recommended (see also Reviewer #3, Comments A–D, H, and Minor Comment 6).

# Reviewer #3

## Major Comments

**Comment A — Rationale for the infection model and experimental design:** *The authors induced infection after administering both cyclophosphamide and streptomycin prior to E. coli inoculation. This creates a highly artificial disease model. Are the observed pathological changes truly due to E. coli infection, or are they partly due to cyclophosphamide-induced immunotoxicity and streptomycin-induced dysbiosis? The rationale for combining cyclophosphamide and streptomycin should be clearly explained.*

Response A1: We thank the Reviewer for raising this critical point. We agree that pretreatment with cyclophosphamide and streptomycin adds complexity to the physiological baseline; however, this combination follows a well-established, highly reproducible methodological precedent for inducing an acute, standardised intestinal *E. coli* infection in adult rabbits (Nguiam et al., 2021), for two complementary reasons: (1) streptomycin transiently clears the facultative anaerobic Gram-negative gut microflora that would otherwise confer strong colonisation resistance, opening an ecological niche that allows the challenge strain to colonise and proliferate; and (2) cyclophosphamide induces transient immunosuppression that mimics the opportunistic susceptibility seen in young, weanling, or stressed animals in the field, ensuring a reliable, synchronised onset of acute clinical colibacillosis across cohorts. This rationale has been added to the revised Methods section.

Addressing Potential Confounding Pathological Changes:

To determine whether the pathological changes observed were attributable to the infection itself rather than to the pretreatment, we included a dedicated immunosuppression-only control group (Group T5), which received the same cyclophosphamide pretreatment but was not inoculated with *E. coli* (see Response A2 below for the exact T5 regimen). By Day 7 post-infection (9 days post-pretreatment), Group T5 showed no significant differences (P > 0.05) from the healthy, untreated control group (T0) in clinical scores, body weight gain, hematological profile, or tissue histopathology, whereas the infected, untreated group (T1) exhibited severe diarrhoea, weight loss, systemic inflammation, and marked histopathological damage. This parity between T5 and T0 indicates that the physiological and immunological perturbations caused by the pretreatment were transient and largely resolved by the study endpoint, supporting the interpretation that the pathology documented in the infected groups reflects the *E. coli* challenge.

We nonetheless agree that the reliance on chemically induced susceptibility is an inherent limitation of this model, and we have added this point explicitly to the new "Limitations of the Study" subsection of the Discussion.

**Comment -** *The manuscript includes an “immunosuppression control” group (T5), without a sufficiently detailed description of the treatment regimen. The authors should clarify whether T5 received cyclophosphamide alone or both cyclophosphamide and streptomycin.*

Response A2: We confirm that Group T5 (Immunosuppression Control) received cyclophosphamide alone and did not receive streptomycin, so as to isolate the biological effects of chemical immunosuppression on hematological profile, oxidative stress, and tissue architecture, independent of antibiotic-induced gut dysbiosis and of the *E. coli* challenge. We have expanded the Materials and Methods to state explicitly that animals in Group T5 received a single intraperitoneal injection of cyclophosphamide (30 mg/kg bw) 48 hours before the designated infection timeline, did not undergo streptomycin clearance or bacterial inoculation, and received daily oral gavage of vehicle (10 mL/kg bw sterile distilled water) for 7 consecutive days starting 24 hours post-induction, matching the handling stress and administration volume of the treated cohorts.

***Comment-*** *The authors need to discuss the limitations of this highly manipulated infection model, as cyclophosphamide itself can induce oxidative stress, haematological changes, and organ toxicity. Accordingly, several reported outcomes may not solely reflect colibacillosis.*

Response A3: We agree that cyclophosphamide is a potent alkylating agent capable of inducing transient myelosuppression, systemic oxidative stress, and organ toxicity, and that this is an important limitation of chemically facilitated infection models. To isolate the contribution of cyclophosphamide from that of the infection, we compared Group T5 (cyclophosphamide alone, uninfected) with Group T0 (healthy control) and Group T1 (infected, untreated). By Day 7 post-infection, the hematological, biochemical, and tissue lipid-peroxidation parameters of Group T5 were statistically indistinguishable from those of Group T0 (P > 0.05), in clear contrast to the severe, persistent leukopenia, systemic inflammation (elevated TNF-α, IL-6, CRP), oxidative stress (elevated MDA; depleted SOD and catalase), and tissue damage observed in Group T1. This indicates that the acute toxicological effects of cyclophosphamide had largely resolved by the time of sample collection, although we agree that early-stage cyclophosphamide toxicity remains a confounding variable and a limitation of the model. A dedicated “Limitations of the Study” subsection has been added to the Discussion, and the T5-versus-T0/T1 comparison is now presented there explicitly to help the reader distinguish the relative contribution of cyclophosphamide from that of the infection.

**Comment B — Lack of phytochemical characterisation and extract standardisation:** *The major weakness of the study is the absence of phytochemical profiling and standardisation of the two extracts used. The authors repeatedly attribute the biological effects to the presence of flavonoids, tannins, and sesquiterpene lactones, and to NF-κB modulation, without quantification or characterisation of these compounds (e.g., by HPLC, LC-MS/MS, total phenolic/flavonoid quantification, or chromatographic fingerprinting). Otherwise, the mechanistic claims should be substantially toned down.*

Response B: Our previous study (Chouegouong et al., 2023) reported the chemical fingerprint of these same two extracts, confirming the presence of several chemical classes, including terpenes, flavonoids, and tannins; this reference has been cited in the revised manuscript. Moreover, we also performer an LC-MS analysis of crude extracts from *Psidium guajava* L. and *Tithonia diversifolia* that allow us to annotate **Pedunculagin (**from *P. guajava***),** and **Quercetin, Quercetin 4′-O-glucoside, Myricetin-3-O-pentoside,** and **2′-Hydroxy-alpha-naphthoflavone** (from *T. diversifolia*). This new information has been added to the discussion and the supplementary materials.

Moreover, all mechanistic statements attributing the observed effects to specific phytochemical classes or to NF-κB modulation have been moderated and reframed as plausible hypotheses consistent with the prior phytochemical characterisation, rather than as demonstrated mechanisms (see also Comment E).

**C) Small sample size and statistical power concerns**

The study used only n = 5 rabbits/group, with one mortality in the untreated infected group and only four animals/group ultimately analyzed histologically and biochemically. The endpoints measured are quite large in comparison with the number of animals per group. Accordingly, The authors should**:**

• provide statistical power justification,

• clarify whether normality testing was performed,

• explain how multiple testing inflation was controlled,

• and discuss the limitations imposed by the small sample size.

Response C: We completely acknowledge the reviewer’s valuable methodological concerns regarding statistical power, sample sizes, and the risk of Type I and Type II errors when evaluating multiple physiological endpoints. We have addressed each point as follows and updated the Statistical Analysis and Limitations subsections accordingly.

1. Statistical power: The initial (n = 5/group) and final analytical (n = 4/group, following early humane euthanasia in Group T1 and the planned reduction of one animal per surviving cohort under the 3Rs framework) sample sizes were set based on the large, polarised effect sizes typically observed between healthy and untreated-infected phenotypes in acute lagomorph enteric infection models. An a priori power analysis indicated that for a large anticipated effect size (Cohen's f ≥ 0.70), n = 4 animals per group is sufficient to achieve power (1 − β) of 0.80 at α = 0.05, balancing statistical reliability against the 3Rs (Reduction) principle.

2. Normality testing: Data normality was assessed using the Shapiro-Wilk test, and homogeneity of variance using Levene's test; all datasets satisfied these assumptions (P > 0.05), supporting the use of parametric statistical models.

3. Multiple-testing control: To avoid Type I error inflation from repeated pairwise comparisons across six groups, all multi-group variables were analysed by one-way ANOVA followed by Tukey's HSD post-hoc test, which controls the family-wise error rate at α = 0.05.

4. Limitations: We acknowledge that, while adequately powered for the large-magnitude primary therapeutic effects observed, the small sample size restricts our ability to detect subtler or secondary effects and may not fully capture natural physiological variability in outbred rabbit populations; this is now explicitly stated in the Limitations of the Study.

**D) Absence of quantitative histopathology evaluation**

The histopathological analysis is entirely descriptive mentioning “leukocyte infiltration, villous atrophy, restoration,” etc. However, no histological scoring system was used, and no semi-quantitative analysis was provided. The authors should: introduce validated histopathological scoring systems, quantify inflammatory infiltration and villus height/crypt depth, and provide lesion severity scores.

Response D: We completely agree with the reviewer that semi-quantitative scoring and morphometric measurement (e.g., villus height, crypt depth) would add valuable numerical depth. At the time of this study, our facility did not have access to digital histomorphometric software or automated image-analysis systems required for these sub-micron measurements in lagomorph intestinal tissue; the histopathological evaluation was therefore designed as a rigorous, descriptive, qualitative assessment. To limit subjectivity, sections were evaluated by a blinded observer focused on identifying unambiguous, pathognomonic signs of acute colibacillosis (e.g., mucosal desquamation, denuded villi, extensive inflammatory infiltrates). As shown in the revised figures, the structural differences between groups are marked: the infected-untreated group shows extensive destruction of intestinal architecture, while the high-dose extract group shows visibly preserved mucosal integrity comparable to the healthy control. We have added the absence of semi-quantitative scoring as an explicit limitation in the Discussion and clarified the qualitative nature of the histological methodology in the Materials and Methods.

**E) Cytokine and mechanistic claims are overstated**

The authors repeatedly conclude that the extracts used caused NF-κB inhibition, immunomodulation, ROS scavenging mechanisms, membrane stabilization, and bacterial adhesion inhibition, without experimental evaluation of these pathways. Only general screening parameters were measured. Mechanistic statements should be rewritten more cautiously and presented as hypotheses rather than demonstrated mechanisms.

For example: “may involve modulation of inflammatory pathways”

instead of: “likely mediated through NF-κB inhibition.”

Response E**:** We have rewritten the Results and Discussion so that mechanistic statements are consistently framed as hypotheses supported by our observed endpoints (e.g., “may involve modulation of inflammatory pathways”) rather than as demonstrated mechanisms (e.g., “likely mediated through NF-κB inhibition”), throughout the manuscript.

## Comment F — Important missing methodological details

Several experimental details are insufficiently described, including the following:

*Randomisation: the manuscript states that rabbits were “randomly assigned,” but the randomisation method is not described.*

Response F1 — Randomisation**:** We thank the reviewer for pointing out this missing methodological detail. We agree that specifying the exact method of randomization is essential for transparency and reproducibility. In the revised manuscript, we have updated the Materials and Methods section to explicitly describe the randomization protocol. Specifically, we have clarified that a simple randomization procedure was performed using a computer-generated random number sequence (https://www.random.org/lists/), ensuring that each animal had an equal and independent probability of being assigned to any of the six experimental cohorts.

Response F2 — Blinding**:** To eliminate observer and detection bias, a strict blinding protocol was implemented across multiple stages of the study. The investigators conducting daily clinical scoring were blinded to the treatment groups by having an independent colleague manage the preparation and coding of the treatment solutions. Furthermore, all downstream laboratory analyses, including hematological processing, biochemical biomarker assays, and histopathological tissue evaluations were performed by investigators who were completely blinded to the experimental groups. All samples and tissue slides were labeled with anonymous, randomized tracking codes, which were only unblinded after the final statistical analysis was completed.

*Sex distribution: the rabbits were of “mixed sex,” but the distribution per group was not provided. Sex-dependent differences may influence the immune response.*

Response F3 — Sex distribution**:** Statistical analysis revealed no significant difference between males and females in infection or treatment response under the present experimental conditions. The sex distribution per group (2 males / 3 females per group) has been added to the revised manuscript.

*Extract formulation: the exact ratio between the two combined extracts is not clearly stated.*

Response F4 — Extract formulation: Combined extract formulations of *P. guajava* and *T. diversifolia* were freshly prepared in distilled water daily. The ratio of the two extracts within the combination (96.975% *P. guajava* and 3.025% *T. diversifolia*) was determined based on the synergistic Fractional Inhibitory Concentration Index (FICI = 0.265) identified in our prior *in vitro* assays (Chouegouong et al., 2023). To administer the target doses of 400mg/kg and 800 mg/kg body weight (bw) at a standardized oral gavage volume of 1 mL per 100 g bw (10 mL/kg bw), two distinct stock solutions were prepared. The stock solution of 40 mg/mL (for the 400 mg/kg group) was prepared by dissolving 310.32 mg of *P. guajava* extract and 9.68 mg of *T. diversifolia* extract in 8 mL of sterile distilled water. This delivered 387.9 mg/kg of *P. guajava* and 12.1 mg/kg of *T. diversifolia* *in vivo*. The second stock solution of 80mg/mL (for the 800mg/kg group) was Prepared by dissolving 620.64 mg of *P. guajava* extract and 19.36 mg of *T. diversifolia* extract in 8mL of sterile distilled water. This delivered 775.8mg/kg of *P. guajava* and 24.2 mg/kg of *T. diversifolia* *in vivo*.

**Comment G:** *Bacterial characterisation is insufficient. The isolate was identified only by API 20E and the Congo red binding assay. However, the study repeatedly refers to EPEC, virulence, pathogenicity, and attaching/effacing lesions. No molecular confirmation of virulence genes was performed. The authors should avoid definitive classification as EPEC unless molecular virulence markers are confirmed, or should include PCR confirmation for the relevant virulence genes.*

Response G: We agree that definitive classification as Enteropathogenic *E. coli* (EPEC) requires molecular confirmation of diagnostic virulence markers (e.g., eaeA, bfpA) by PCR, which was not performed here; our isolate was characterised only phenotypically, using the API 20E system for species-level identification (Fig 1) and the Congo red binding assay to confirm pathogenic phenotype. We have therefore removed the term “EPEC” throughout the manuscript (Abstract, Introduction, Methods, Results, Discussion) and replaced it with “pathogenic *E. coli*”, and we have toned down or rephrased speculative references to specific microscopic attaching/effacing lesions to describe generalised enteropathogenic tissue alterations consistent with our phenotypic and histological data.

**Comment H:** *PCA and multivariate analysis appear overinterpreted. The PCA and heatmap analyses are visually attractive, but the sample size is small, the number of variables is large, and no validation strategy is described. Statements such as “biological reset” and “systemic recovery indistinguishable from healthy baseline” are exaggerated given the exploratory nature of PCA. The authors should moderate their interpretation, clarify the variance explained, and discuss the limitations of multivariate modelling with small datasets.*

Response H: We agree that applying PCA to a high-dimensional dataset (46 variables) with a small sample size (n = 4/group) without an independent validation cohort carries a substantial risk of overfitting, and that these results must not be overinterpreted. We have removed exaggerated or definitive claims (e.g., “mathematically demonstrates”, references to an absolute “biological reset”), clarified the exact variance explained by each dimension (Dim1 = 48%, Dim2 = 9.7%; Fig 10), and explicitly reframed the PCA and heatmap as descriptive, exploratory visualisations rather than predictive or confirmatory models. A statement on the limitations of multivariate analysis with small sample sizes has been added to the Discussion (see also the revised legends for Figs 9 and 10).

**Comment I:** *The discussion following several results sections lacks sufficient referencing and critical comparison with the literature. The authors should discuss their findings in relation to previous studies, emphasising agreements, discrepancies, possible explanations, and remaining research gaps. For example, reports on Psidium species indicate antidiarrheal activity through antimicrobial effects, not solely through tannins (DOI: https://doi.org/10.1016/j.jep.2025.119670).*

Response I: We have substantially revised the Discussion to integrate and critically compare our findings with the relevant literature, including the reference suggested by the Reviewer (DOI: 10.1016/j.jep.2025.119670) on antimicrobial, rather than purely tannin-mediated, antidiarrheal mechanisms of Psidium species. All new text is original and properly cited, and the corresponding changes are indicated in the revised manuscript (see also Minor Comment 2).

## Minor Comments

**Minor Comment 1:** *Several sections contain overly promotional language inappropriate for scientific writing, including:*

• “biological reset”

• “high-activity extract”

• “profound therapeutic trajectory”

• “gold-standard treatment”

*• “extinguishing inflammatory cascades.”*

*The manuscript should adopt a more objective scientific tone.*

Response 1: All overstated expressions, including those listed, have been replaced with objective, scientifically appropriate language throughout the manuscript.

**Minor Comment 2:** *The discussion is excessively long and repetitive. Several statements restate the results instead of critically interpreting them (see also Comment I above).*

Response 2**:** To eliminate redundancy, we condensed and refocused the Discussion. The revised text (1) compares our findings with previous studies, (2) proposes plausible mechanistic pathways while explicitly noting their hypothetical status, (3) discusses the constraints of the present study, and (4) outlines specific follow-up experiments (fractionation, adhesion assays, pharmacokinetics, safety/toxicology). References have been reorganised to support this structure.

**Minor Comment 3:** *The authors should clarify whether the units used for ALT and AST, cytokine concentrations, and oxidative stress markers were standardised and validated for rabbit samples (i.e., that the kits used are validated for use with rabbit samples).*

Response 3: We have revised the Materials and Methods section to clearly specify the assay validation, species applicability, and units of measurement for all biochemical and immunological parameters.

**Minor Comment 4:** *Figure legends appear insufficiently detailed. They should be revised to be self-interpretable in some sections, including magnification, stain details, scale bars, and statistical annotations.*

Response 4: We agree that legends must be fully self-interpretable. All 14 figure legends have been rewritten (see the accompanying Revised Figure Legends document): each now states the sample size (n = 4 animals/group analysed), expands all technical abbreviations at first mention, and specifies the statistical test and software used. For the histopathological figures (Figs 12–14), the legends now specify hematoxylin and eosin staining, original magnification (×100), and a scale bar; because scale-bar values were not recorded in the original image files available to us, these are flagged with a placeholder for the corresponding author to insert before resubmission.

**Minor Comment 5:** *Adherence to the ARRIVE guidelines is mentioned, but several ARRIVE-required elements remain incomplete, namely sample size justification, exclusion criteria, and blinding details.*

Response 5: The Methods section now includes an explanation of the sample size (see Response C), the predetermined inclusion/exclusion criteria, and a full description of the blinding procedure at the clinical-scoring, laboratory-analysis, and data-interpretation stages (see Response F2). The updated ARRIVE 2.0 checklist (https://arriveguidelines.org/arrive-guidelines) is submitted as a supplementary submission

**Minor Comment 6:** *The conclusion somewhat overstates translational applicability. The statement that the extract is “clinically equivalent to conventional aminoglycosides” is not fully supported without pharmacokinetic data, toxicity studies, dose optimisation, long-term safety data, and field validation. The conclusion must be revised accordingly.*

Response 6: The conclusion has been rewritten to remove the claim of clinical equivalence to conventional aminoglycosides and to present the translational potential of the extract in a manner consistent with the evidence available (comparable performance on the specific biomarkers measured at Day 7, without pharmacokinetic, long-term safety, or field validation data), in line with the newly added Limitations of the Study.
