# Supplementary material for: A combined aqueous extract of Psidium guajava L. and Tithonia diversifolia (Hemsl.) A. gray improves metabolic homeostasis and preserves intestinal integrity in experimental rabbit colibacillosis
Source: PLoS One. 2026 Sep 15;21(9):e0357951. doi: 10.1371/journal.pone.0357951 (PMC13577389; doi:10.1371/journal.pone.0357951)
Supplement: Peer review history

# Peer review: Decision Letter 0

**DOI:** 10.1371/journal.pone.0357951.r001

**Authors:** Seth Domfeh

4 Jun 2026

Dear Dr. Boyom,

Thank you for submitting your manuscript to PLOS ONE. After careful consideration, we feel that it has merit but does not fully meet PLOS ONE’s publication criteria as it currently stands. Therefore, we invite you to submit a revised version of the manuscript that addresses the points raised during the review process.

Please submit your revised manuscript by Jul 19 2026 11:59PM. If you will need more time than this to complete your revisions, please reply to this message or contact the journal office at plosone@plos.org. When you're ready to submit your revision, log on to https://www.editorialmanager.com/pone/ and select the 'Submissions Needing Revision' folder to locate your manuscript file.

- A letter that responds to each point raised by the academic editor and reviewer(s). You should upload this letter as a separate file labeled 'Response to Reviewers'.
- A marked-up copy of your manuscript that highlights changes made to the original version. You should upload this as a separate file labeled 'Revised Manuscript with Track Changes'.
- An unmarked version of your revised paper without tracked changes. You should upload this as a separate file labeled 'Manuscript'.

If you would like to make changes to your financial disclosure, please include your updated statement in your cover letter. Guidelines for resubmitting your figure files are available below the reviewer comments at the end of this letter.

If applicable, we recommend that you deposit your laboratory protocols in protocols.io to enhance the reproducibility of your results. Protocols.io assigns your protocol its own identifier (DOI) so that it can be cited independently in the future. For instructions see: https://journals.plos.org/plosone/s/submission-guidelines#loc-laboratory-protocols. Additionally, PLOS ONE offers an option for publishing peer-reviewed Lab Protocol articles, which describe protocols hosted on protocols.io. Read more information on sharing protocols at https://plos.org/protocols?utm_medium=editorial-email&utm_source=authorletters&utm_campaign=protocols.

As the corresponding author, your ORCID iD is verified in the submission system and will appear in the published article. PLOS supports the use of ORCID, and we encourage all coauthors to register for an ORCID iD and use it as well. Please encourage your coauthors to verify their ORCID iD within the submission system before final acceptance, as unverified ORCID iDs will not appear in the published article. *Only*  the individual author can complete the verification step; PLOS staff *cannot*  verify ORCID iDs on behalf of authors.

We look forward to receiving your revised manuscript.

Kind regards,

Seth Agyei Domfeh, PhD

Academic Editor

PLOS One

Journal Requirements:

When submitting your revision, we need you to address these additional requirements.

1.Please ensure that your manuscript meets PLOS ONE’s style requirements, including those for file naming. The PLOS ONE style templates can be found at

https://journals.plos.org/plosone/s/file?id=wjVg/PLOSOne_formatting_sample_main_body.pdf and

https://journals.plos.org/plosone/s/file?id=ba62/PLOSOne_formatting_sample_title_authors_affiliations.pdf

2. As part of your revision, please complete and submit a copy of the Full ARRIVE 2.0 Guidelines checklist, a document that aims to improve experimental reporting and reproducibility of animal studies for purposes of post-publication data analysis and reproducibility: https://arriveguidelines.org/sites/arrive/files/documents/Author%20Checklist%20-%20Full.pdf Please include your completed checklist as a Supporting Information file. Note that if your paper is accepted for publication, this checklist will be published as part of your article.

3. Please include your full ethics statement in the ‘Methods’ section of your manuscript file. In your statement, please include the full name of the IRB or ethics committee who approved or waived your study, as well as whether or not you obtained informed written or verbal consent. If consent was waived for your study, please include this information in your statement as well.

4. If the reviewer comments include a recommendation to cite specific previously published works, please review and evaluate these publications to determine whether they are relevant and should be cited. There is no requirement to cite these works unless the editor has indicated otherwise.

[Note: HTML markup is below. Please do not edit.]

Reviewer’s Responses to Questions

**Comments to the Author**

1. Is the manuscript technically sound, and do the data support the conclusions?

Reviewer #1: Yes

Reviewer #2: Partly

Reviewer #3: Partly

**********

2. Has the statistical analysis been performed appropriately and rigorously?

Reviewer #1: Yes

Reviewer #2: Yes

Reviewer #3: No

**********

3. Have the authors made all data underlying the findings in their manuscript fully available?

The PLOS Data policy

Reviewer #1: Yes

Reviewer #2: Yes

Reviewer #3: Yes

**********

4. Is the manuscript presented in an intelligible fashion and written in standard English?

Reviewer #1: Yes

Reviewer #2: Yes

Reviewer #3: No

**********

Reviewer #1: The manuscript is interesting but some corrections need to be addressed. The volume of the bacterial suspension for induction should be included. Also, the software used for data analysis should also be included. The term polyherbal extract should be replaced with the combined extract of ....... Moreso, The preparation of the combined extract should be included, and how the doses for the experiment was determined (It seems high). Most sentences should start appropriately, avoid starting sentence with 'to,by' . The explanations of the results obtained do not reflect the results or experiments done. There is alot of overstatement. The conclusion should be written to reflect the work done, be it for the abstract and the entire manuscript. Authors should rewrite the manuscript reflecting the results obtained. So figures are confusing to the understanding of the audience. The manuscript has some indications and has been highlighted.

Reviewer #2: 1. There are some grammatical, alignments and typographical errors are noted in the manuscript and it should be thoroughly checked and corrected throughout the manuscript.

2. The use of abbreviations in the abstract may distract readers who wish to quickly skim through several publications before deciding to read one in full. It may therefore help to write out terms fully in abstract (For example, AST).

3. Check the abbreviations throughout the manuscript and introduce the abbreviation when the full word appears first time in the abstract and the remaining for the text and then use only the abbreviation (For example, IL-1β, IL-6, superoxide dismutase (SOD), etc.,). Make a word abbreviated in the article that is repeated at least three times in the text, not all words to be abbreviated.

4. The full form of the species should be given when the first time appears in both the abstract and in the remaining part of the manuscript and it should be followed by only the first letter of the genus (For example, Psidium guajava / Tithonia diversifolia when the first time appear and followed by P. guajava / T. diversifolia) and this should be checked all other species used in the manuscript.

5. The time (season) of collection of this species should be added. As it is well-known that one of the factors affecting the constituents, is the time of collection, so please give top priority to this issue.

6. The authors may include the details of the quantity of fresh sample used and the quantity of the powder obtained (the ratio between fresh plant and powder) under the heading materials and methods.

7. The standard diet used in the present investigation should also be included with its composition and source of the same.

8. In the materials and methods, the authors may cite references for standard protocol, manufacture instructions, if reference is given with it and the same should be added in the reference section.

9. The authors may justify how the dose optimized for the present investigation in the materials and methods (either based on available literature or previous study). If from the others study should be supported with reference.

10. The authors should mention the following when referring to GraphPad Prism version 8.0.1 (GraphPad Software, San Diego, CA, USA).

11. The auhors should be given necessary expansion in the figure legend, for example, MDA, SOD, CAT, and GSH.

12. The conclusion provides a comprehensive overview of the work, but it could be a bit more realistic based on the evidence of the results for better understanding of the outcome of the present investigation. Moreover, the authors may also include the limitation of the present findings for a better understanding of the manuscript.

Reviewer #3: Evaluation of the manuscript entitled “A Combined Aqueous Extract of Psidium guajava and Tithonia diversifolia Restores Metabolic Homeostasis and Intestinal Integrity in Experimental Rabbit Colibacillosis”

ID: PONE-D-26-07694

The manuscript investigates the therapeutic efficacy of a combined herbal extract in an experimentally induced rabbit colibacillosis model. The study addresses an important topic regarding increased incidence of antimicrobial resistance and the search for alternative veterinary therapeutics.

The manuscript includes microbiological, biochemical, hematological, immunological, antioxidant, and histopathological evaluations. However, there are several major methodological and scientific concerns, particularly regarding the in vivo study design, characterization of the infection model, extract standardization, statistical analysis, and interpretation of the results.

Major comments:

A) The rationale for choosing the infection model and experimental design

1. The authors induced infection after administering both cyclophosphamide and streptomycin prior to E. coli inoculation. This creates a highly artificial model of disease but:

Are the observed pathological changes truly due to E. coli infection, or partially due to cyclophosphamide-induced immunotoxicity and streptomycin-induced dysbiosis? Clearly explain the rationale for combining cyclophosphamide and streptomycin.

2. The manuscript includes an “immunosuppression control” group (T5), without mentioning a sufficient description of the treatment regimen: The authors should:

• Clarify whether T5 received cyclophosphamide alone or both cyclophosphamide and streptomycin,

• Provide complete data and discussion for T5.

3. The authors need to discuss the limitations of this highly manipulated infection model as cyclophosphamide itself can induce several effects like oxidative stress, hematological changes, and organ toxicity. Accordingly, several reported outcomes may not solely reflect colibacillosis.

B) Lack of phytochemical characterization and extract standardization

The major weakness of the study is the absence of phytochemical profiling and standardization of the two used extracts. The authors repeatedly referred the biological effects to the presence of flavonoids, tannins, and sesquiterpene lactones, with NF-κB modulation, without quantification and characterization of the present compounds using, for example, HPLC, LC-MS/MS, total phenolic/flavonoid quantification, or identification of the major compounds, including chromatographic fingerprinting. Otherwise, mechanistic claims should be toned down substantially.

C) Small sample size and statistical power concerns

The study used only n = 5 rabbits/group, with one mortality in the untreated infected group and only four animals/group ultimately analyzed histologically and biochemically. The endpoints measured are quite large in comparison with the number of animals per group. Accordingly, The authors should:

• provide statistical power justification,

• clarify whether normality testing was performed,

• explain how multiple testing inflation was controlled,

• and discuss the limitations imposed by the small sample size.

D) Absence of quantitative histopathology evaluation

The histopathological analysis is entirely descriptive mentioning “leukocyte infiltration, villous atrophy, restoration,” etc. However, no histological scoring system was used, and no semi-quantitative analysis was provided. The authors should: introduce validated histopathological scoring systems, quantify inflammatory infiltration and villus height/crypt depth, and provide lesion severity scores.

E) Cytokine and mechanistic claims are overstated

The authors repeatedly conclude that the extracts used caused NF-κB inhibition, immunomodulation, ROS scavenging mechanisms, membrane stabilization, and bacterial adhesion inhibition, without experimental evaluation of these pathways. Only general screening parameters were measured. Mechanistic statements should be rewritten more cautiously and presented as hypotheses rather than demonstrated mechanisms.

For example: “may involve modulation of inflammatory pathways”

instead of: “likely mediated through NF-κB inhibition.”

F) Important missing methodological details

Several experimental details are insufficiently described, including:

1. Randomization: The manuscript states rabbits were “randomly assigned,” but the randomization method is not described.

2. Blinding: No mention of blinding during treatment administration, histological evaluation, or data analysis.

3. Sex distribution: The rabbits were “mixed sex,” but the distribution per group was not provided. Sex-dependent differences may influence immune response.

4. Extract formulation: The exact ratio between the two combined extracts is not clearly stated.

5. Dose justification: The rationale for the selected doses is not mentioned

G) Bacterial characterization is insufficient

The isolate was identified only by API 20E and Congo red binding assay. However, the study repeatedly refers to EPEC, virulence, pathogenicity, attaching/effacing lesions.

No molecular confirmation of virulence genes was performed. The authors should avoid definitive classification as EPEC unless molecular virulence markers are confirmed,

or include PCR confirmation for relevant virulence genes.

H) PCA and multivariate analysis appear overinterpreted

The PCA and heatmap analyses are visually attractive, but the sample size is extremely small, the number of variables is very large, and no validation strategy is described. Statements such as: “biological reset” and “systemic recovery indistinguishable from healthy baseline” are exaggerated considering the exploratory nature of PCA.

The authors should moderate interpretation, clarify variance explained, and discuss limitations of multivariate modeling with small datasets.

I) Discussion parts after each results section need references and referral to the literature

The discussion following several results sections lacks sufficient referencing and critical comparison with the literature. The authors should discuss their findings in relation to previous studies, emphasizing agreements, discrepancies, possible explanations, and remaining research gaps. For example, Psidium species records indicate antidiarrheal activity through antimicrobial effects, not only for tannins (DOI: https://doi.org/10.1016/j.jep.2025.119670)

Minor Comments

1. Several sections contain overly promotional language inappropriate for scientific writing, including:

• “biological reset,”

• “high-activity extract,”

• “profound therapeutic trajectory,”

• “gold-standard treatment,”

• “extinguishing inflammatory cascades.”

The manuscript should include a more objective scientific tone.

2. The discussion is excessively long and repetitive. Several statements restate results instead of critically interpreting them (back to comment no. I in the major comments).

3. The authors should clarify whether ALT and AST units, cytokine units, and oxidative stress marker units, were standardized and validated for rabbit samples (ensure the kits are valid for rabbit samples).

4. Figure legends appear insufficiently detailed. They should be revised to be self-interpreted in some sections through including:

• magnification,

• stain details,

• scale bars,

• and statistical annotations.

5. The ARRIVE guideline adherence is mentioned, but several ARRIVE-required elements remain incomplete:

• sample size justification,

• exclusion criteria,

• blinding details.

6. The conclusion somewhat overstates translational applicability. The statement that the extract is “clinically equivalent to conventional aminoglycosides” is not fully supported without pharmacokinetics, toxicity studies, dose optimization, long-term safety, and field validation. Accordingly, the conclusion must be revised.

The work should not be accepted in its current form, but it may become suitable for publication after major methodological clarification, further specific analysis, and thorough revision of the interpretation and presentation of the data.

**********

what does this mean?). If published, this will include your full peer review and any attached files.

If you choose “no”, your identity will remain anonymous but your review may still be made public.

**Do you want your identity to be public for this peer review?**  For information about this choice, including consent withdrawal, please see our Privacy Policy

Reviewer #1: No

Reviewer #2: **Yes:** Dr. A. Vijaya Anand

Reviewer #3: No

**********

[NOTE: If reviewer comments were submitted as an attachment file, they will be attached to this email and accessible via the submission site. Please log into your account, locate the manuscript record, and check for the action link "View Attachments". If this link does not appear, there are no attachment files.]

To ensure your figures meet our technical requirements, please review our figure guidelines: https://journals.plos.org/plosone/s/figures

You may also use PLOS’s free figure tool, NAAS, to help you prepare publication quality figures: https://journals.plos.org/plosone/s/figures#loc-tools-for-figure-preparation.

NAAS will assess whether your figures meet our technical requirements by comparing each figure against our figure specifications.

Attachment Submitted filename: PONE-D-26-07694 Revised Dr Zeukoo.pdf

Attachment Submitted filename: PONE-D-26-07694.docx

# Author comment: Author response to Decision Letter 1

**DOI:** 10.1371/journal.pone.0357951.r002

20 Jul 2026

Response to Reviewers' Comments

Comments reviewers and editor: We thank the Editor and the three Reviewers for their constructive evaluation of our manuscript. Based on they inputs and recommendations, we have revised the entire manuscript, remade all figures and figure captions. Below, we tried to the best of our ability to respond point-by-point to every comment. Reviewer comments are reproduced in bold italics; our responses follow in red.

Slight change in the title: The original title was revised to align with the revised manuscript. The new title is: A Combined Aqueous Extract of Psidium guajava L. and Tithonia diversifolia (Hemsl.) A. Gray Improves Metabolic Homeostasis and Preserves Intestinal Integrity in Experimental Rabbit Colibacillosis

Manuscript ID: PONE-D-26-07694

Reviewer #1

The manuscript is interesting, but some corrections need to be addressed.

Comment 1: The volume of the bacterial suspension used for induction should be included.

Response 1: The disease was induced by administering 1 mL /100 g of body weight of bacterial suspension, adjusted to a 109 CFU/mL in 0.9% saline. This information has been added to the Materials and Methods section.

Comment 2: The software used for data analysis should also be included.

Response 2: Data were analysed using GraphPad Prism and OriginPro, and multivariate analysis was performed in the R environment. This information has been added to the corresponding Methods section (see also Reviewer #2, Comment 10, for the full software citation).

Comment 3: The term “polyherbal extract” should be replaced with “the combined extract of …”.

Response 3: The term “polyherbal extract” has been replaced throughout the manuscript with “the combined Psidium guajava and Tithonia diversifolia extracts,” as suggested.

Comment 4: The preparation of the combined extract should be described, and the rationale for the doses used in the experiment should be explained (the doses appear high).

Response 4: The preparation of the combined extract has been fully described in the Methods section. Briefly, the two extracts were combined in a 96.975% P. guajava : 3.025% T. diversifolia ratio (w/w), determined from the synergistic Fractional Inhibitory Concentration Index (FICI = 0.265) established in our prior in vitro work (Chouegouong et al., 2023; doi:10.1007/s13596-021-00615-1). Fresh stock solutions were prepared daily in sterile distilled water at 40 mg/mL and 80 mg/mL to deliver 400 and 800 mg/kg bw, respectively. The administration was done by oral gavage at a fixed volume of 10 mL/kg bw (i.e., 387.9 mg/kg P. guajava + 12.1 mg/kg T. diversifolia for the low dose, and 775.8 mg/kg P. guajava + 24.2 mg/kg T. diversifolia for the high dose). The selected doses (400 and 800 mg/kg bw) are consistent with standard pharmacological evaluations of crude phytotherapeutic agents in lagomorph models, as previously reported by Hirudkar et al. (2020); this rationale, together with the full preparation protocol, has been added to the Methods section.

Comment 5: Most sentences should be structured appropriately; sentences should not begin with “to” or “by”.

Response 5: Constructions beginning with “To” or “By” have been revised throughout the manuscript to improve phrasing and fluency.

Comment 6: The explanations of the results obtained do not accurately reflect the results or experiments performed; there is considerable overstatement.

Response 6: The results section has been rephrased to remove overstatement and to ensure that the interpretations presented are directly supported by the data obtained.

Comment 7: The conclusion should be rewritten to accurately reflect the work done, both in the abstract and throughout the manuscript.

Response 7: The conclusion has been rewritten in both the abstract and the main text to align precisely with the results obtained (see also Reviewer #2, Comment 12, and Reviewer #3, Minor Comment 6).

Comment 8: The manuscript should be rewritten to better reflect the results obtained; the figures are confusing for the reader.

Response 8: The manuscript has been revised to better reflect the data generated, all figures are now explicitly cited and described within the text, and every figure legend has been rewritten to be self-contained and self-interpretable (see the accompanying Revised Figure Legends document, and Reviewer #2, Minor Comment 4).

Comment 9: The manuscript contains some inconsistencies, which have been highlighted.

Response 9: Thank you to all reviewers for flagging these inconsistencies. We have revised the entire manuscript and checked all results individually against the raw data and corrected; the corresponding changes are marked in the revised manuscript.

Reviewer #2

Comment 1: Some grammatical, alignment, and typographical errors are noted in the manuscript; it should be thoroughly checked and corrected throughout.

Response 1: We thank the Reviewer for this observation. We have carefully re-read the entire manuscript and performed a thorough revision to correct all grammatical, alignment, and typographical errors identified.

Comment 2: The use of abbreviations in the abstract may distract readers who wish to quickly skim several publications before deciding to read one in full. It may help to write terms out in full in the abstract (for example, AST).

Response 2: We have revised the abstract to write out all abbreviated terms in full.

Comment 3: Abbreviations should be checked throughout the manuscript: the full term should be introduced at first mention in the abstract and again at first mention in the main text, after which only the abbreviation should be used (for example, IL-1β, IL-6, superoxide dismutase (SOD), etc.). Only terms repeated at least three times in the text should be abbreviated.

Response 3: We have thoroughly reviewed all abbreviations throughout the manuscript, introducing each full term at first mention before subsequent use of the abbreviation, and removing abbreviations for terms that did not meet the repetition threshold. The corresponding revisions have been incorporated into the updated manuscript and into the revised figure legends (see accompanying document).

Comment 4: The full species name should be given at first mention in both the abstract and the main text, followed thereafter by the abbreviated genus initial (for example, Psidium guajava / Tithonia diversifolia at first mention, followed by P. guajava / T. diversifolia); this should be checked for all species used in the manuscript.

Response 4: We have revised the entire manuscript and edited the full species of the two plants.

Comment 5: The time (season) of collection of the plant species should be added, since the collection period is known to affect phytochemical constituents; this should be given top priority.

Response 5: We completely agree with the reviewer that transparency in botanical processing is fundamental for reproducibility. We have now updated the "Ethnobotanical Selection and Extract Preparation" section to include the exact exact collection dates and season for both plant species, the starting fresh weights, resulting dry powder weights, and calculated fresh-to-dry ratios for both species.

Comment 6: The quantity of fresh plant material used and the quantity of powder obtained (i.e., the fresh-to-powder ratio) should be included under Materials and Methods.

Response 6: We have added the quantities of fresh plant material and the corresponding dry powder yield to the Materials and Methods section.

Comment 7: The standard diet used in the study should be described, including its composition and source.

Response 7: Information regarding the composition of the standard diet has been incorporated into the revised manuscript.

Comment 8: In the Materials and Methods, references should be cited for any standard protocols or manufacturer instructions used, and the corresponding citations should be added to the reference list.

Response 8: The Methods section has been updated to include references for the standard protocols used, and the corresponding citations have been added to the reference list.

Comment 9: The dose selected for the present study should be justified in the Materials and Methods, either based on the available literature or a previous study; if derived from another study, it should be supported with the appropriate reference.

Response 9: The dose justification, supported by the relevant reference (Chouegouong et al., 2023; Hirudkar et al., 2020), has been incorporated into the revised Materials and Methods section (see also Reviewer #1, Comment 4).

Comment 10: The authors should cite the software fully when referring to it. GraphPad Prism version 8.0.1 (GraphPad Software, San Diego, CA, USA).

Response 10: We have updated the Methods section to include this full software citation.

Comment 11: Necessary expansions should be given in the figure legends, for example for MDA, SOD, CAT, and GSH.

Response 11: We have rewritten every figure legend so that all abbreviations (including MDA, SOD, CAT and GSH) are expanded at first mention within each individual legend, in line with journal style for self-contained legends. Please see the accompanying Revised Figure Legends document.

Comment 12: The conclusion provides a comprehensive overview of the work but could be more realistic in relation to the evidence presented, to better convey the outcome of the investigation. The authors may also wish to include the limitations of the present findings.

Response 12: We have revised the conclusion to be more precise and directly grounded in the evidence generated by the present study, and we have added a dedicated statement on the limitations of the study and on future investigations, as recommended (see also Reviewer #3, Comments A–D, H, and Minor Comment 6).

Reviewer #3

Major Comments

Comment A — Rationale for the infection model and experimental design: The authors induced infection after administering both cyclophosphamide and streptomycin prior to E. coli inoculation. This creates a highly artificial disease model. Are the observed pathological changes truly due to E. coli infection, or are they partly due to cyclophosphamide-induced immunotoxicity and streptomycin-induced dysbiosis? The rationale for combining cyclophosphamide and streptomycin should be clearly explained.

Response A1: We thank the Reviewer for raising this critical point. We agree that pretreatment with cyclophosphamide and streptomycin adds complexity to the physiological baseline; however, this combination follows a well-established, highly reproducible methodological precedent for inducing an acute, standardised intestinal E. coli infection in adult rabbits (Nguiam et al., 2021), for two complementary reasons: (1) streptomycin transiently clears the facultative anaerobic Gram-negative gut microflora that would otherwise confer strong colonisation resistance, opening an ecological niche that allows the challenge strain to colonise and proliferate; and (2) cyclophosphamide induces transient immunosuppression that mimics the opportunistic susceptibility seen in young, weanling, or stressed animals in the field, ensuring a reliable, synchronised onset of acute clinical colibacillosis across cohorts. This rationale has been added to the revised Methods section.

Addressing Potential Confounding Pathological Changes:

To determine whether the pathological changes observed were attributable to the infection itself rather than to the pretreatment, we included a dedicated immunosuppression-only control group (Group T5), which received the same cyclophosphamide pretreatment but was not inoculated with E. coli (see Response A2 below for the exact T5 regimen). By Day 7 post-infection (9 days post-pretreatment), Group T5 showed no significant differences (P > 0.05) from the healthy, untreated control group (T0) in clinical scores, body weight gain, hematological profile, or tissue histopathology, whereas the infected, untreated group (T1) exhibited severe diarrhoea, weight loss, systemic inflammation, and marked histopathological damage. This parity between T5 and T0 indicates that the physiological and immunological perturbations caused by the pretreatment were transient and largely resolved by the study endpoint, supporting the interpretation that the pathology documented in the infected groups reflects the E. coli challenge.

We nonetheless agree that the reliance on chemically induced susceptibility is an inherent limitation of this model, and we have added this point explicitly to the new "Limitations of the Study" subsection of the Discussion.

Comment - The manuscript includes an “immunosuppression control” group (T5), without a sufficiently detailed description of the treatment regimen. The authors should clarify whether T5 received cyclophosphamide alone or both cyclophosphamide and streptomycin.

Response A2: We confirm that Group T5 (Immunosuppression Control) received cyclophosphamide alone and did not receive streptomycin, so as to isolate the biological effects of chemical immunosuppression on hematological profile, oxidative stress, and tissue architecture, independent of antibiotic-induced gut dysbiosis and of the E. coli challenge. We have expanded the Materials and Methods to state explicitly that animals in Group T5 received a single intraperitoneal injection of cyclophosphamide (30 mg/kg bw) 48 hours before the designated infection timeline, did not undergo streptomycin clearance or bacterial inoculation, and received daily oral gavage of vehicle (10 mL/kg bw sterile distilled water) for 7 consecutive days starting 24 hours post-induction, matching the handling stress and administration volume of the treated cohorts.

Comment- The authors need to discuss the limitations of this highly manipulated infection model, as cyclophosphamide itself can induce oxidative stress, haematological changes, and organ toxicity. Accordingly, several reported outcomes may not solely reflect colibacillosis.

Response A3: We agree that cyclophosphamide is a potent alkylating agent capable of inducing transient myelosuppression, systemic oxidative stress, and organ toxicity, and that this is an important limitation of chemically facilitated infection models. To isolate the contribution of cyclophosphamide from that of the infection, we compared Group T5 (cyclophosphamide alone, uninfected) with Group T0 (healthy control) and Group T1 (infected, untreated). By Day 7 post-infection, the hematological, biochemical, and tissue lipid-peroxidation parameters of Group T5 were statistically indistinguishable from those of Group T0 (P > 0.05), in clear contrast to the severe, persistent leukopenia, systemic inflammation (elevated TNF-α, IL-6, CRP), oxidative stress (elevated MDA; depleted SOD and catalase), and tissue damage observed in Group T1. This indicates that the acute toxicological effects of cyclophosphamide had largely resolved by the time of sample collection, although we agree that early-stage cyclophosphamide toxicity remains a confounding variable and a limitation of the model. A dedicated “Limitations of the Study” subsection has been added to the Discussion, and the T5-versus-T0/T1 comparison is now presented there explicitly to help the reader distinguish the relative contribution of cyclophosphamide from that of the infection.

Comment B — Lack of phytochemical characterisation and extract standardisation: The major weakness of the study is the absence of phytochemical profiling and standardisation of the two extracts used. The authors repeatedly attribute the biological effects to the presence of flavonoids, tannins, and sesquiterpene lactones, and to NF-κB modulation, without quantification or characterisation of these compounds (e.g., by HPLC, LC-MS/MS, total phenolic/flavonoid quantification, or chromatographic fingerprinting). Otherwise, the mechanistic claims should be substantially toned down.

Response B: Our previous study (Chouegouong et al., 2023) reported the chemical fingerprint of these same two extracts, confirming the presence of several chemical classes, including terpenes, flavonoids, and tannins; this reference has been cited in the revised manuscript. Moreover, we also performer an LC-MS analysis of crude extracts from Psidium guajava L. and Tithonia divers

Attachment Submitted filename: Response_to_Reviewers_200726 (1).docx

# Peer review: Decision Letter 1

**DOI:** 10.1371/journal.pone.0357951.r003

**Authors:** Seth Domfeh

7 Aug 2026

Dear Dr. Boyom,

Thank you for submitting your manuscript to PLOS ONE. After careful consideration, we feel that it has merit but does not fully meet PLOS ONE’s publication criteria as it currently stands. Therefore, we invite you to submit a revised version of the manuscript that addresses the points raised during the review process.

Please submit your revised manuscript by Sep 21 2026 11:59PM. If you will need more time than this to complete your revisions, please reply to this message or contact the journal office at plosone@plos.org. When you're ready to submit your revision, log on to https://www.editorialmanager.com/pone/ and select the 'Submissions Needing Revision' folder to locate your manuscript file.

- A letter that responds to each point raised by the academic editor and reviewer(s). You should upload this letter as a separate file labeled 'Response to Reviewers'.
- A marked-up copy of your manuscript that highlights changes made to the original version. You should upload this as a separate file labeled 'Revised Manuscript with Track Changes'.
- An unmarked version of your revised paper without tracked changes. You should upload this as a separate file labeled 'Manuscript'.

If you would like to make changes to your financial disclosure, please include your updated statement in your cover letter. Guidelines for resubmitting your figure files are available below the reviewer comments at the end of this letter.

If applicable, we recommend that you deposit your laboratory protocols in protocols.io to enhance the reproducibility of your results. Protocols.io assigns your protocol its own identifier (DOI) so that it can be cited independently in the future. For instructions see: https://journals.plos.org/plosone/s/submission-guidelines#loc-laboratory-protocols. Additionally, PLOS ONE offers an option for publishing peer-reviewed Lab Protocol articles, which describe protocols hosted on protocols.io. Read more information on sharing protocols at https://plos.org/protocols?utm_medium=editorial-email&utm_source=authorletters&utm_campaign=protocols.

As the corresponding author, your ORCID iD is verified in the submission system and will appear in the published article. PLOS supports the use of ORCID, and we encourage all coauthors to register for an ORCID iD and use it as well. Please encourage your coauthors to verify their ORCID iD within the submission system before final acceptance, as unverified ORCID iDs will not appear in the published article. *Only*  the individual author can complete the verification step; PLOS staff *cannot*  verify ORCID iDs on behalf of authors.

We look forward to receiving your revised manuscript.

Kind regards,

Seth Agyei Domfeh, PhD

Academic Editor

PLOS One

**Journal Requirements:**

1. If the reviewer comments include a recommendation to cite specific previously published works, please review and evaluate these publications to determine whether they are relevant and should be cited. There is no requirement to cite these works unless the editor has indicated otherwise.

2. Please review your reference list to ensure that it is complete and correct. If you have cited papers that have been retracted, please include the rationale for doing so in the manuscript text, or remove these references and replace them with relevant current references. Any changes to the reference list should be mentioned in the rebuttal letter that accompanies your revised manuscript. If you need to cite a retracted article, indicate the article’s retracted status in the References list and also include a citation and full reference for the retraction notice.

[Note: HTML markup is below. Please do not edit.]

Reviewers' comments:

Reviewer’s Responses to Questions

**Comments to the Author**

Reviewer #1: All comments have been addressed

Reviewer #2: All comments have been addressed

Reviewer #3: All comments have been addressed

**********

2. Is the manuscript technically sound, and do the data support the conclusions?

Reviewer #1: Yes

Reviewer #2: Partly

Reviewer #3: Yes

**********

3. Has the statistical analysis been performed appropriately and rigorously?

Reviewer #1: Yes

Reviewer #2: Yes

Reviewer #3: Yes

**********

4. Have the authors made all data underlying the findings in their manuscript fully available?

The PLOS Data policy

Reviewer #1: Yes

Reviewer #2: Yes

Reviewer #3: Yes

**********

5. Is the manuscript presented in an intelligible fashion and written in standard English?

Reviewer #1: Yes

Reviewer #2: Yes

Reviewer #3: Yes

**********

**Reviewer #1:**  The authors should include the statistical software used in the methods section of the abstract. Also, the sentences starting with to, for, and any, should be constructed appropriately. The graph axis should be checked and labelled.The limitations should be written in point form. The references should be checked and edited, font size checked too. The manuscript has been highlighted.

**Reviewer #2:** 1. This suggestion is not carried out properly and it should be rectified (Check the abbreviations throughout the manuscript and introduce the abbreviation when the full word appears first time in the abstract and the remaining for the text and then use only the abbreviation). For example, IL-1β, IL-6, C-reactive protein (CRP), superoxide dismutase (SOD), and these types of corrections need to be checked all other abbreviations used in the manuscript.

2. The technical terms (Latin Phrase) "in vitro" should be italic and it should be checked all over the manuscript.

**Reviewer #3:** The authors addressed all the requested comments and edits.

The manuscript is suitable to be accepted in PLOS ONE.

**********

what does this mean?). If published, this will include your full peer review and any attached files.

If you choose “no”, your identity will remain anonymous but your review may still be made public.

**Do you want your identity to be public for this peer review?**  For information about this choice, including consent withdrawal, please see our Privacy Policy

Reviewer #1: No

Reviewer #2: **Yes:** Dr. A. Vijaya Anand

Reviewer #3: No

**********

[NOTE: If reviewer comments were submitted as an attachment file, they will be attached to this email and accessible via the submission site. Please log into your account, locate the manuscript record, and check for the action link "View Attachments". If this link does not appear, there are no attachment files.]

To ensure your figures meet our technical requirements, please review our figure guidelines: https://journals.plos.org/plosone/s/figures

You may also use PLOS’s free figure tool, NAAS, to help you prepare publication quality figures: https://journals.plos.org/plosone/s/figures#loc-tools-for-figure-preparation.

NAAS will assess whether your figures meet our technical requirements by comparing each figure against our figure specifications.

Attachment Submitted filename: PONE-D-26-07694_R1 Revised Dr Zeukoo .pdf

# Author comment: Author response to Decision Letter 2

**DOI:** 10.1371/journal.pone.0357951.r004

11 Aug 2026

Response to Reviewers' Comments

Manuscript ID: PONE-D-26-07694R1

Reviewer #1:

Comment1: The authors should include the statistical software used in the methods section of the abstract.

Response 1: The names of the softwares employed for the data analysis have now been mentioned in the Methods section of the abstract.

Comment2: Also, the sentences starting with to, for, and any, should be constructed appropriately.

Response 2: The sentences starting with " to, for, and any " have been revised in the entire manuscript.

Comment 3: The graph axis should be checked and labelled. The limitations should be written in point form.

Response 3: The figures axis was reviewed and revised, and the limitations section written in point form.

Comment 4: The references should be checked and edited, font size checked too. The manuscript has been highlighted.

Response 4: The references and manuscript formatting, including font size, were carefully checked and corrected according to the journal’s guidelines.

Reviewer #2:

Comment 1. This suggestion is not carried out properly and it should be rectified (Check the abbreviations throughout the manuscript and introduce the abbreviation when the full word appears first time in the abstract and the remaining for the text and then use only the abbreviation). For example, IL-1β, IL-6, C-reactive protein (CRP), superoxide dismutase (SOD), and these types of corrections need to be checked all other abbreviations used in the manuscript.

Response 1: All abbreviations have been carefully checked and standardized throughout the manuscript.

Comment 2. The technical terms (Latin Phrase) "in vitro" should be italic and it should be checked all over the manuscript.

Response 2: The expression in vitro, as well as other Latin expressions such as in vivo, has been italicized consistently throughout the manuscript.

Attachment Submitted filename: Response to Reviewers Comments 10 08 26.doc

# Peer review: Decision Letter 2

**DOI:** 10.1371/journal.pone.0357951.r005

**Authors:** Seth Domfeh

25 Aug 2026

A Combined Aqueous Extract of Psidium guajava L. and Tithonia diversifolia (Hemsl.) A. Gray Improves Metabolic Homeostasis and Preserves Intestinal Integrity in Experimental Rabbit Colibacillosis

PONE-D-26-07694R2

Dear Dr. Boyom,

We’re pleased to inform you that your manuscript has been judged scientifically suitable for publication and will be formally accepted for publication once it meets all outstanding technical requirements.

Within one week, you’ll receive an e-mail detailing the required amendments. When these have been addressed, you’ll receive a formal acceptance letter and your manuscript will be scheduled for publication.

An invoice will be generated when your article is formally accepted. Please note, if your institution has a publishing partnership with PLOS and your article meets the relevant criteria, all or part of your publication costs will be covered. Please make sure your user information is up-to-date by logging into Editorial Manager at Editorial Manager® and clicking the ‘Update My Information' link at the top of the page. For questions related to billing, please contact billing support.

If your institution or institutions have a press office, please notify them about your upcoming paper to help maximize its impact. If they’ll be preparing press materials, please inform our press team as soon as possible -- no later than 48 hours after receiving the formal acceptance. Your manuscript will remain under strict press embargo until 2 pm Eastern Time on the date of publication. For more information, please contact onepress@plos.org.

Kind regards,

Seth Agyei Domfeh, PhD

Academic Editor

PLOS One

Additional Editor Comments (optional):

Reviewers' comments:

Reviewer’s Responses to Questions

**Comments to the Author**

Reviewer #1: All comments have been addressed

Reviewer #2: All comments have been addressed

**********

2. Is the manuscript technically sound, and do the data support the conclusions?

Reviewer #1: Yes

Reviewer #2: Yes

**********

3. Has the statistical analysis been performed appropriately and rigorously?

Reviewer #1: Yes

Reviewer #2: Yes

**********

4. Have the authors made all data underlying the findings in their manuscript fully available?

The PLOS Data policy

Reviewer #1: Yes

Reviewer #2: Yes

**********

5. Is the manuscript presented in an intelligible fashion and written in standard English?

Reviewer #1: Yes

Reviewer #2: Yes

**********

Reviewer #1: All the comments have been addressed, the manuscript is clear and easily understood and has been improved significantly. The manuscript can be accepted for publication.

Reviewer #2: 1. This suggestion is not carried out properly and it should be rectified (Check the abbreviations throughout the manuscript and introduce the abbreviation when the full word appears first time in the abstract and the remaining for the text and then use only the abbreviation). For example, in line numbers 75 and 76 both form is given for interleukin-1beta (IL-1β), interleukin-6 (IL-6) and the same is repeated in line number 230 and it should be replaced as IL-1β, IL-6, respctively, and these types of corrections need to be checked all other abbreviations used in the manuscript.

**********

what does this mean?). If published, this will include your full peer review and any attached files.

If you choose “no”, your identity will remain anonymous but your review may still be made public.

**Do you want your identity to be public for this peer review?**  For information about this choice, including consent withdrawal, please see our Privacy Policy

Reviewer #1: No

Reviewer #2: **Yes:** Dr. A. Vijaya Anand

**********

# Editor report: Acceptance letter

**DOI:** 10.1371/journal.pone.0357951.r006

**Authors:** Seth Domfeh

PONE-D-26-07694R2

PLOS One

Dear Dr. Boyom,

I'm pleased to inform you that your manuscript has been deemed suitable for publication in PLOS One. Congratulations! Your manuscript is now being handed over to our production team.

At this stage, our production department will prepare your paper for publication. This includes ensuring the following:

* All references, tables, and figures are properly cited

* All relevant supporting information is included in the manuscript submission,

* There are no issues that prevent the paper from being properly typeset

You will receive further instructions from the production team, including instructions on how to review your proof when it is ready. Please keep in mind that we are working through a large volume of accepted articles, so please give us a few days to review your paper and let you know the next and final steps.

Lastly, if your institution or institutions have a press office, please let them know about your upcoming paper now to help maximize its impact. If they'll be preparing press materials, please inform our press team within the next 48 hours. Your manuscript will remain under strict press embargo until 2 pm Eastern Time on the date of publication. For more information, please contact onepress@plos.org.

You will receive an invoice from PLOS for your publication fee after your manuscript has reached the completed accept phase. If you receive an email requesting payment before acceptance or for any other service, this may be a phishing scheme. Learn how to identify phishing emails and protect your accounts at https://explore.plos.org/phishing.

If we can help with anything else, please email us at customercare@plos.org.

Thank you for submitting your work to PLOS One and supporting open access.

Kind regards,

PLOS One Editorial Office Staff

on behalf of

Dr. Seth Agyei Domfeh

Academic Editor

PLOS One
